# Molecular Cloning, Characterization and Positively Selected Sites of the Glutathione *S*-Transferase Family from *Locusta migratoria*

**DOI:** 10.1371/journal.pone.0114776

**Published:** 2014-12-08

**Authors:** Xueyao Zhang, Jianxin Wang, Min Zhang, Guohua Qin, Daqi Li, Kun Yan Zhu, Enbo Ma, Jianzhen Zhang

**Affiliations:** 1 Research Institute of Applied Biology, Shanxi University, Taiyuan, Shanxi, China; 2 Department of Entomology, Kansas State University, Manhattan, Kansas, United States of America; University of Cincinnati, United States of America

## Abstract

Glutathione *S*-transferases (GSTs) are multifunctional enzymes that are involved in the metabolism of endogenous and exogenous compounds and are related to insecticide resistance. The purpose of this study was to provide new information on the molecular characteristics and the positive selection of locust GSTs. Based on the transcriptome database, we sequenced 28 cytosolic GSTs and 4 microsomal GSTs from the migratory locust (*Locusta migratoria*). We assigned the 28 cytosolic GSTs into 6 classes—sigma, epsilon, delta, theta, omega and zeta, and the 4 microsomal GSTs into 2 subclasses—insect and MGST3. The tissue- and stage-expression patterns of the GSTs differed at the mRNA level. Further, the substrate specificities and kinetic constants of the cytosolic GSTs differed markedly at the protein level. The results of likelihood ratio tests provided strong evidence for positive selection in the delta class. The result of Bayes Empirical Bayes analysis identified 4 amino acid sites in the delta class as positive selection sites. These sites were located on the protein surface. Our findings will facilitate the elucidation of the molecular characteristics and evolutionary aspects of insect GST superfamily.

## Introduction

The migratory locust (*Locusta migratoria*) is an important agricultural pest in East and South Asia and the Pacific Region. The locust has developed insecticide resistance because of frequent applications of insecticides. Increased glutathione *S*-transferase (GST, EC 2.5.1.18) activity has been reported to be one of the main reasons for the resistance in *L. migratoria* [1].

The GSTs constitute a superfamily of multifunctional proteins found in most aerobic prokaryotes and eukaryotes [2], [3]. GSTs catalyze the conjugation of glutathione (GSH) to electrophilic compounds, which are involved in detoxification of endogenous and xenobiotic compounds [4], [5]. Some GSTs also have GSH-dependent peroxidase, isomerase, thiol transferase, dehydroascorbate reductase, and non-catalytically binding activities [6]. They participate in endogenous metabolism or regulation of physiological function *in vivo*. In insects, the GST superfamily consists of 2 major classes, namely, cytosolic (soluble) and microsomal GSTs. The latter are also known as membrane-associated proteins in eicosanoid and glutathione metabolism (MAPEG).

Based on the sequence identity, substrate specificity, and immunological properties, insect cytosolic GSTs are assigned into at least 7 distinct classes, namely delta, epsilon, sigma, omega, theta, zeta, and unknown [7].The delta and epsilon classes are insect-specific GST classes, and are involved in detoxification of insecticides. The epsilon GSTs of *Anopheles gambiae* play a role in response to oxidative stress, by catalyzing the dehydrochlorination of DDT to yield DDE, thereby conferring resistance to DDT [8]. Enhanced transcription of delta GSTs from *Sarcoptes scabiei* is related to permethrin resistance [9]. Sigma GSTs are induced by oxidative stress and also participate in the detoxification and multi-bioprocesses, such as detoxification of epoxides, peroxides and 4-HNE, and response to carbaryl [10], mercury [11] and bacterial [12]. Sigma GSTs are also involved in isomerization of PGH_2_ to PGD_2_ [13]. Zeta GSTs catalyze the isomerization of maleylacetoacetate, and play important roles in catabolism of phenylalanine and tyrosine [14].

Microsomal GSTs are classified into 6 classes, namely class 1 (MGST1, insect, and PGES1 subclasses), class 2 (MGST2, LTC_4_S, and FLAP subclasses), class 3 (MGST3 subclass), *Synechocystis* class, *E. coli* class, and bacteria class [15], [16]. Microsomal GSTs play a major role in the biosynthesis of prostaglandins and leukotrienes, and are also involved in the detoxification of xenobiotics and protection against oxidative damage [16]. MGST1 is involved in the detoxification of toxic or carcinogenic substances and protection against oxidative stress [17], [18]. PGES1 functions downstream of cyclooxygenase, and catalyzes the isomerization of PGH_2_ to PGE_2_ under basal and inflammatory conditions [19], [20]. MGST3 catalyzes the conjugation of GSH and LTA_4_ to form LTC_4_, and the reduction of 5*S*-hydroperoxy-6*t*,8*c*,11*c*,14*c*-eicosatetraenoic acid (5-HPETE) to 5*S*-hydroxy- eicosatetraenoic acid (5-HETE) in the leukotriene synthesis pathway [21].

We previously identified 10 cytosolic GSTs from *L. migratoria* and assigned these GSTs into sigma, delta, theta, and unknown classes. We further showed that the sigma GSTs participated in carbaryl and malathion detoxification, whereas the delta GSTs were involved in the detoxification of carbaryl and chlorpyrifos [2]. However, to date, few systematic studies regarding the characterization of GSTs from *L. migratoria* have been performed. The goal of the study was to provide new information on the GSTs from *L. migratoria* and help researchers uncover the molecular characteristics and evolutionary aspects of insect GSTs.

In the present study, we sequenced and classified 32 full-length GST sequences from *L. migratoria*, and analyzed their tissue- and stage-dependent expression patterns by using real time quantitative PCR (RT-qPCR). We heterologously expressed 25 of these GST genes by using *E. coli* expression system We purified the GSTs by using NTA-Ni^2+^ affinity chromatography. Finally, we identified positive selection sites of the GSTs by using the branch model, site model, and Bayes empirical Bayes (BEB) methods, and labeled these sites by using 3-dimensional homology models. Our findings will facilitate the elucidation of the molecular characteristics and evolutionary history of insect GSTs.

## Materials and Methods

### Reagents and assay kits

RevertAid H minus reverse transcriptase was purchased from Fermentas (Waltham, MA, USA). Fastpfu DNA polymerase and the pEASY-Blunt zero cloning kit were obtained from Transgen Biotech Co. Ltd. (Beijing, China). The SMARTer RACE cDNA amplification kit and the His60 Ni Superflow resin were purchased from Clontech (Palo Alto, CA, USA). The substrates of GSTs, 2,4-dinitrochlorobenzene (CDNB), 2,4-dinitrofluorobenzene (FDNB), 3,4- dichloronitrobenzene (DCNB), 4-chloro-7-nitrobenzofurazan (NBD-Cl), 4-nitrobenzyl chloride (pNBC), 4-nitrophenethyl bromide (pNPB), and reduced glutathione (GSH) were from Sigma (St. Louis, MO). All other reagents used were of the highest grade commercially available.

### Molecular cloning and phylogenetic analysis

Total RNA was extracted by using TRIzol (TaKaRa, Japan), and mRNA was subsequently isolated by using the PolyATtract mRNA isolation systems. The cDNA was synthesized by using the SMARTer RACE cDNA amplification kit. RACE PCR was performed by using Fastpfu DNA polymerase, according to the manufacturer's instructions. The PCR product was subcloned into the pEASY-Blunt zero vector and sequenced by Sangon Biotech (Shanghai, China). The phylogenetic tree was constructed based on full-length DNA sequences, by using the neighbor-joining algorithm with mega 5.0 software.

### Analysis of tissue- and stage-dependent expression patterns

For the tissue-specific expression profile analysis, we dissected 12 different tissues, including the foregut, midgut, hindgut, gastric caecum, Malpighian tubules, cuticle, spermary, ovary, trachea, fat bodies, antenna, and muscles (from legs and back) from adult individuals of *L. migratoria*. For the stage-specific expression profile analysis, we collected whole bodies of *L. migratoria* individuals at 8 different developmental stages, including the egg (5 days and 10 days), and the 1st, 2nd, 3rd, 4th, and 5th-instar nymphs and adults. We extracted total RNA from these samples, and used 4 µg total RNA to synthesize cDNA, by using oligo-d(T) and RevertAid H minus reverse transcriptase. The primers used for RT-qPCR analysis are shown in S1 Table. The thermal cycling profile consisted of two steps, including 94°C for 5 s, 60°C for 31 s, followed by melting curve analysis. The fluorescence intensity was monitored during each annealing step. Relative transcript levels were determined by using the double standard curve method with *β-actin* as a reference gene. The experiment was repeated with 3 biological replicates, each with 2 technical replicates.All data were analyzed by the one-way ANOVA, LSD test.

### Protein expression and enzymatic activity assay

The open reading frames of the GSTs were subcloned into the pET28a vector, and the recombinant vectors were used to transform *E. coli* BL21 (DE3). The recombinant GSTs were induced by using 1 mM isopropyl-β-d-thiogalactopyranoside (IPTG) at 25°C, and purified by using NTA-Ni^2+^-affinity chromatography. The enzymatic activities were measured spectrophotometrically, as described previously [3]. The protein concentration was determined by using the BCA method with BSA as the standard protein.

### Homology structure modeling

The sequence of LmGSTD1 as a target sequence was submitted to BLASTP program (http://blast.ncbi.nlm.nih.gov/). Then the program searched against Protein Database (PDB) of known protein structures, and then the X-ray structure of Delta GST (3EIN) from *Drosophila melanogaster* were selected as template structure. Finally, the homology model of LmGSTD1 was generated by MODELLER 9v12 through comparative protein structure prediction [22].

### Positive selection site analysis

To investigate the selective pressure among phylogenies, we used the branch model of the PAML package [23]. The simplest model (one-ratio) assumes a single ω for the entire tree, whereas the most general model (free-ratio) assumes a ω for each branch. To determine whether positive selection had acted on specific amino acid sites of the GSTs, we explored 5 models of CODEML [24]—the one-ratio model (M0), nearly neutral model (M1a), positive-selection model (M2a), beta model (M7), and beta and v model (M8). We conducted two likelihood ratio tests (LRTs), which compared M1a with M2a, and M7 with M8, to verify whether the difference ratio ω differed significantly from 1 for each pairwise comparison. The LRT compared the likelihood scores of the pairwise models, with the constraint of ω = 1, and without such constraint: LR = 2 |ln1−ln2|. If the LRT was significant, we used the BEB [25] method to identify positive selection sites in the alignments. Finally, we labeled the positive selection sites on the homology structure of the corresponding GSTs.

## Results

### Identification of GST genes from *L. migratoria*

Based on the transcriptome of *L. migratoria* (S1 Data), we identified candidate GST gene fragments by keyword searching. We subsequently cloned 28 cytosolic GSTs and 4 microsomal GSTs from *L. migratoria*, including 10 known cytosolic GST genes of *L. migratoria* (S2 Data, Table 1), by using RACE PCR. The estimated numbers of GSTs may be lower than the actual numbers of GSTs in the locust genome, because of the limitations of transcriptome technology. However, our data are in good agreement with the number of GSTs in the genome of *L. migratoria* (28 cytosolic GSTs)[26], implying that almost all of the cytosolic GST genes of *L. migratoria* were detected based on the transcriptome, and were identified and sequenced by using RACE PCR.

**Table 1:** Summary of the character of GST gene from *L. migratoria*.

GSTs Genes	GenBank Accession No.	ORF(bp)	Deduced amino acid residues	Predicted MM(kD)	Theoretical p*I*
*LmGSTs1*	AEB91973.1	615	204	23.64	5.46
*LmGSTs2*	AEB91974.1	615	204	23.14	6.62
*LmGSTs3*	AEB91975.1	615	204	23.40	7.62
*LmGSTs4*	AEB91976.1	615	204	22.92	8.42
*LmGSTs5*	AEB91977.1	609	202	23.10	5.72
*LmGSTs6*	AEB91978.1	579	192	22.10	6.74
*LmGSTs7*	AEB91979.1	615	204	23.43	7.58
*LmGSTs8*	AHC08043.1	615	204	23.58	5.78
*LmGSTs9*	AHC08044.1	615	204	23.47	6.19
*LmGSTs10*	AHC08045.1	615	204	23.68	5.82
*LmGSTo1*	AHC08060.1	729	242	26.96	5.73
*LmGSTo2*	AFK10494.1	738	245	29.02	6.97
*LmGSTo3*	AHC08062.1	726	241	26.92	6.45
*LmGSTd1*	ADR30117.1	657	218	24.82	5.57
*LmGSTd2*	AHC08055.1	645	214	24.11	7.66
*LmGSTd3*	AHC08056.1	669	222	25.34	5.21
*LmGSTd4*	AHC08057.1	657	218	25.06	6.89
*LmGSTd5*	AHC08058.1	675	224	25.74	5.98
*LmGSTd6*	AEB91972.1	645	214	24.30	7.76
*LmGSTd7*	AHC08059.1	702	233	26.09	6.08
*LmGSTe1*	AHC08046.1	669	222	24.76	6.22
*LmGSTe2*	AHC08047.1	465	154	16.76	5.56
*LmGSTe3*	AHC08048.1	465	154	16.80	5.40
*LmGSTe4*	AHC08049.1	666	221	24.36	5.22
*LmGSTe5*	AHC08050.1	666	221	24.51	5.76
*LmGSTt1*	AEB91980.1	696	231	26.60	7.63
*LmGSTt2*	AHC08064.1	696	231	26.80	8.33
*LmGSTz1*	AHC08063.1	654	217	24.95	8.15
*LmGSTm1*	AHC08051.1	459	152	17.09	9.94
*LmGSTm2*	AHC08052.1	486	161	17.93	9.42
*LmGSTm3*	AHC08053.1	375	124	14.19	9.82
*LmGSTm4*	AHC08054.1	420	139	15.29	9.68

**Figure 1:**
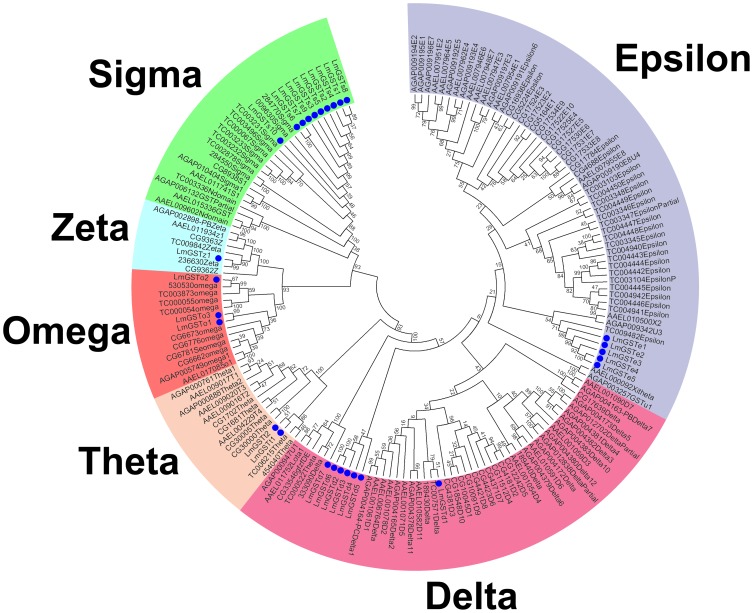
Phylogenetic analysis of cytosolic GSTs from *L. migtatoria* and representative insect species. The bootstrap neighbor-joining tree was generated using MEGA from ClustalW alignments. Branch numbers represent bootstrap values (1000 replicates). The 28 *L. migratoria* GSTs are marked with filled circles. The sequences used to reconstruct the NJ tree are available as S3 Data.

With the exception of *LmGSTe2* and *LmGSTe3*, the cytosolic GSTs of *L. migratoria* had an acidic theoretical pI, and a relatively high molecular mass (22.1–27.0 kDa) (Table 1). To enhance the accuracy of our phylogenetic tree, we retrieved>130 known GST sequences of the representative insects (S3 Data). Based on sequence similarity, the results of our phylogenetic analysis identified 28 cytosolic GSTs, belonging to 6 different cytosolic classes—sigma, epsilon, delta, omega, theta, and zeta (Fig. 1, S2 Table). It was shown that each class of GSTs from most insects clustered in a single clade with a high bootstrap value, thereby further supporting the classification of the *L. migratoria* GSTs. The sigma GSTs were most numerous (10 members), and were followed by the delta and epsilon GSTs (7 and 5 members, respectively), the omega and theta GSTs (3 and 2 members, respectively), and the zeta class (1 member). The former unclassified GST (*LmGSTu1*) was reassigned with high confidence into the delta class (*LmGSTd6*). Furthermore, 9 of the 10 sigma GSTs contained a class-specific Tyr9 residue in the G-site; the omega GSTs had an ancestral Cys9 residue; and the zeta, theta, and delta GSTs contained a conserved Ser in the G-site (S1 Figure).

**Figure 2:**
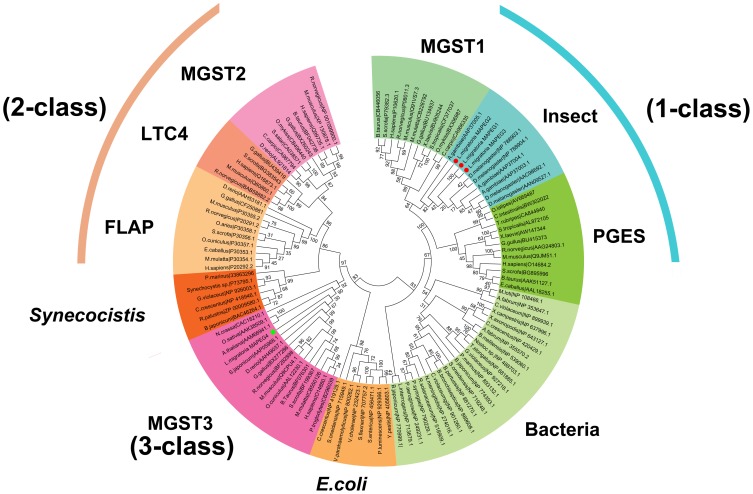
Phylogenetic analysis of microsomal GSTs from *L. migratoria* and representative insect species. The bootstrap neighbor-joining tree was generated using MEGA from ClustalW alignments. Branch numbers represent bootstrap values (1000 replicates). The four *L. migratoria* GSTs are marked with red circle and green square, respectively. The species names and amino acid sequences used to reconstruct the NJ tree are available as S4 Data.

On the other hand, the 4 microsomal GSTs had an alkaline theoretical pI and a relatively low molecular mass (14.2–17.9 kDa, Table 1). The secondary structure prediction indicated that the 4 microsomal GSTs consisted of 3 or 4 transmembrane regions (S2 Figure). To construct our phylogenetic tree, we retrieved>100 known microsomal GSTs from GenBank [15] (S4 Data). Based on high bootstrap support, we clearly identified 9 subclasses of microsomal GSTs (Fig. 2). Among these, 3 microsomal GSTs (*LmGSTm1*, *LmGSTm2*, and *LmGSTm3*) were classified into class 1 (insect subclass), and *LmGSTm4* was assigned into class 3 (MGST3 subclass). To the best of our knowledge, our study is the first to report an insect GST (namely, *LmGSTm4*) as a member of the MGST3 subclass.

### Analysis of tissue- and stage-dependent expression patterns

**Figure 3:**
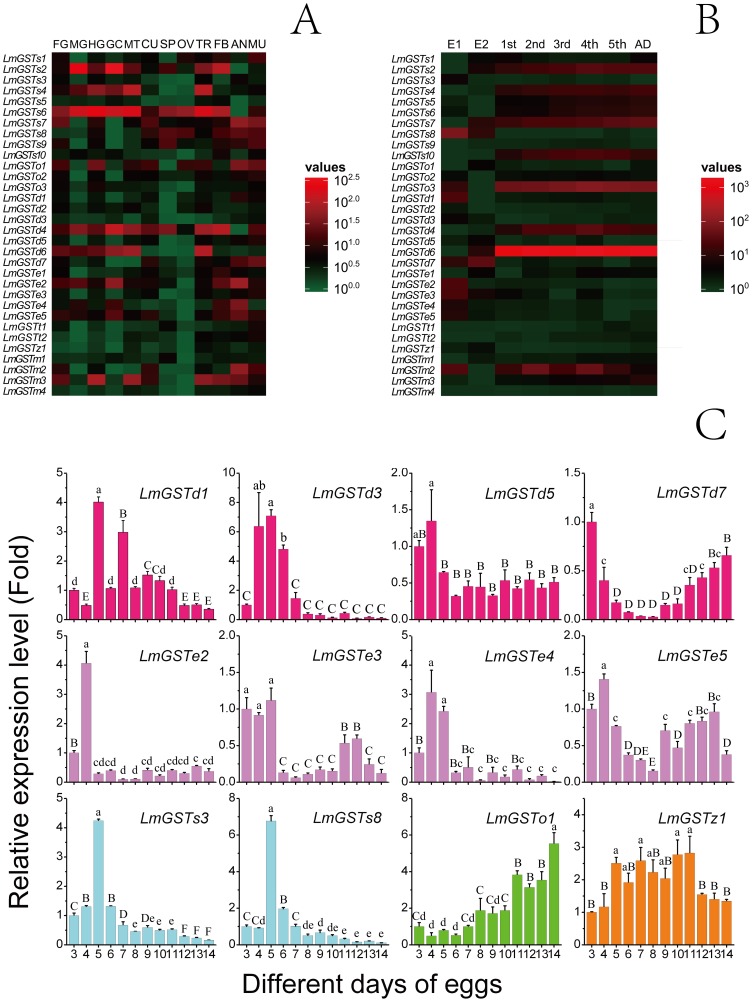
Expression patterns of GST genes from *L. migratoria*. (**A**) Tissue-specific expression patterns of GST genes as evaluated using RT-qPCR: foregut (FG), midgut (MG), hindgut (HG), gastric caecum (GC), Malpighian tubules (MT), cuticle (CU), spermary (SP), ovary (OV), trachea (TR), fat bodies (FB), anterior (AN) and muscles (MU). (**B**) Developmental stage-specific expression patterns of GST genes as evaluated using RT-qPCR: 5-day and 10-day eggs (E1 and E2), first-(1st), second-(2nd), third-(3rd), fourth-(4th) and fifth-(5th) instar nymphs and adults (AD). The β-actin gene was used as a reference gene. For each gene, relative expression levels are shown with the highest as red and the lowest as green. (**C**) Relative expression levels of GST genes in egg stage as determined by RT-qPCR. β-actin was used as a reference gene. Data are expressed as means±SE of three biological replications. Small letters and capital letters indicate significant differences at *p*<0.05 and *p*<0.01 level, according to ANOVA and LSD test, respectively.

Our RT-qPCR analyses showed that the tissue-expression patterns of GSTs could be divided into 3 types, including wide-expression, partial-expression, and specific-expression (Fig. 3A). We observed that 13 GST genes (*LmGSTs1*, *LmGSTs2*, *LmGSTs4*, *LmGSTs6*, *LmGSTs7*, *LmGSTs8*, *LmGSTs9*, *LmGSTo1*, *LmGST*o*2*, *LmGSTd4*, *LmGSTe2*, *LmGSTe5*, *and LmGSTm3*) were widely expressed in ≥6 tissues (P<0.05). In comparison, 12 GSTs (*LmGSTs3*, *LmGSTs10*, *LmGSTd1*, *LmGSTd2*, *LmGSTd5*, *LmGSTd6*, *LmGSTd7*, *LmGSTe1*, *LmGSTe3*, *LmGSTe4*, *LmGSTt2*, and *LmGSTm2*), had relatively narrow tissue-expression ranges (P<0.05). Moreover, *LmGSTt1* was specifically located in the muscles and spermary (P<0.05), whereas *LmGSTz1*, *LmGSTm1*, and *LmGSTm4* were mainly expressed in the trachea, spermary, and muscles, respectively (P<0.05).

The results of stage-dependent expression pattern analysis revealed that the GSTs could be classified into 2 groups, including the egg stage-expression group, and the nymph and adult stages-expression group (Fig. 3B). We observed that 12 GSTs (*LmGSTd1*, *LmGSTd3*, *LmGSTd5*, *LmGSTd7*, *LmGSTe2*, *LmGSTe3*, *LmGSTe4*, *LmGSTe5*, *LmGSTs3*, *LmGSTs8*, *LmGSTo1*, *and LmGSTz1*) showed relatively high expression in the egg stage (P<0.05). Among these, 7 GSTs (*LmGSTd1*, *LmGSTd3*, *LmGSTd5*, *LmGSTe2*, *LmGSTe4*, *LmGSTs3*, and *LmGSTs8*) showed a high expression level in the early egg stage (Fig. 3C) (P<0.05), 1 GST (*LmGSTo1*) was primarily expressed in the late egg stage, and 3 GSTs (*LmGSTd7, LmGSTe3, and LmGSTe5*) were expressed in the early and late egg stages (P<0.05). The remaining GSTs were mainly expressed in the nymphal and adult stages. The different mRNA expression patterns within each GST class may suggest different roles of these genes in different stages and tissues.

### Enzymatic properties of recombinant GSTs from *L. migratoria*

The locust GSTs were selected for protein expression and purification. Most of the GSTs were successfully purified by using a Ni^2+^-NTA column. The exceptions were 4 microsomal GSTs, 1 sigma GST (*LmGSTs6*) and 2 epsilon GSTs (*LmGSTe2* and *LmGSTe3*). However, the reason for the no-expression of these genes is unclear.

In order to explain the possible relationships between the catalytic activity and substrate diversity, we determined the substrate specificity of the recombinant locust GSTs toward 6 electrophilic substrates, including CDNB, FDNB, DCNB, NDB-Cl, pNPC, and pNPB (Table 2). We observed that all the 25 purified cytosolic GSTs showed activity toward CDNB, FDNB, and NDB-Cl; 19 of the purified cytosolic GSTs showed activity towards DCNB (the exceptions being the 3 omega and 1 zeta GSTs, and 2 of the sigma GSTs (LmGSTS10 and LmGSTS2); 8 GSTs were able to catalyze pNPB (the exceptions being the 9 sigma, 3 omega, 4 delta and 1 zeta GSTs); and 18 GSTs were capable of catalyzing pNPC, (the exceptions being the 3 omega and 1 zeta GSTs, and 3 sigma GSTs).

**Table 2:** Specific activity of the GSTs from *L. migratoria* toward six substrates.

Enzyme	CDNB (0.1 mM)	FNDB (1.0 mM)	DCNB (1.0 mM)	NDB-Cl (0.5 mM)	pNPB (0.25 mM)	pNPC (0.5 mM)
LmGSTD1	17.893±0.578	125.035±5.295	0.098±0.002	55.439±1.897	ND	0.611±0.033
LmGSTD2	0.534±0.174	110.065±3.135	0.286±0.007	48.627±0.662	ND	0.158±0.008
LmGSTD3	0.077±0.001	1.563±0.067	0.023±0.001	2.293±0.046	0.032±0.000	0.051±0.001
LmGSTD4	5.508±0.099	5.019±0.265	0.661±0.033	3.327±0.151	0.072±0.006	0.933±0.035
LmGSTD5	3.003±0.113	2.276±0.103	0.101±0.004	1.897±0.042	ND	0.152±0.014
LmGSTD6	8.530±0.153	76.387±1.913	0.776±0.016	22.999±1.055	ND	0.147±0.005
LmGSTD7	1.024±0.020	50.901±1.325	0.004±0.001	2.379±0.073	0.362±0.017	0.669±0.060
LmGSTE1	10.286±0.209	0.509±0.049	8.811±0.094	5.953±0.132	0.445±0.040	24.318±0.635
LmGSTE4	43.716±0.758	31.436±0.717	5.579±0.083	16.714±0.260	0.068±0.006	7.187±0.156
LmGSTE5	14.806±0.284	21.923±0.786	8.672±0.290	1.830±0.057	0.136±0.001	1.605±0.019
LmGSTS1	0.069±0.003	1.162±0.035	0.006±0.001	0.503±0.018	ND	0.210±0.003
LmGSTS2	0.007±0.000	1.640±0.069	ND	0.153±0.011	ND	ND
LmGSTS3	2.407±0.050	18.730±1.817	0.018±0.003	1.302±0.001	ND	ND
LmGSTS4	0.075±0.002	8.000±0.144	0.005±0.001	1.173±0.021	ND	0.180±0.050
LmGSTS5	4.230±0.128	15.044±1.018	0.022±0.001	3.901±0.315	ND	ND
LmGSTS7	0.360±0.007	0.417±0.026	0.042±0.001	0.061±0.005	ND	0.576±0.047
LmGSTS8	0.090±0.004	1.998±0.134	0.006±0.001	0.343±0.008	ND	0.094±0.009
LmGSTS9	0.829±0.039	14.627±0.561	0.101±0.003	1.096±0.015	ND	0.081±0.007
LmGSTS10	0.167±0.006	20.003±1.395	ND	0.563±0.019	ND	5.113±0.149
LmGSTT1	0.075±0.003	1.474±0.042	0.011±0.001	0.171±0.004	0.334±0.029	3.267±0.027
LmGSTT2	0.548±0.013	8.584±0.111	0.013±0.002	1.446±0.027	1.681±0.124	19.611±0.469
LmGSTO1	0.002±0.000	0.260±0.014	ND	0.339±0.010	ND	ND
LmGSTO2	0.012±0.001	0.493±0.064	ND	0.385±0.001	ND	ND
LmGSTO3	0.005±0.000	1.211±0.056	ND	0.202±0.011	ND	ND
LmGSTZ1	0.036±0.001	0.327±0.032	ND	0.206±0.003	ND	ND

The experiment was performed at least five times and the results shown are mean ± SD.

ND, No detectable activity.

We determined the kinetic constants of the 25 GSTs, by using CDNB and GSH as substrates (Table 3). The apparent *K*_m_^CDNB^ varied <70-fold among the GST classes (61.5-fold among the delta class, 28.1-fold among the sigma class, 14.6-fold among the epsilon class, and 3.9-fold among the omega class). By contrast, the catalytic efficiency (*k*_cat_/*K*_m_) of CDNB differed markedly among the GST classes (675.4-fold among the delta class, 103.3-fold among the sigma class, 12.6-fold among the omega class, and 6.0-fold among the epsilon class). On the other hand, the apparent *K*_m_^GSH^ varied <50-fold among the GST classes (45.5-fold among the sigma class, 30.2-fold among the theta class, 8.7-fold among the delta class, and 1.6-fold among the epsilon class). By contrast, the catalytic efficiency (*k*_cat_/*K*_m_) of GSH differed markedly among the GST classes (2482.4-fold among the delta class, 761.8-fold among the sigma class, 7.5-fold among the omega class, and 6.3-fold among the epsilon class).

**Table 3:** SteadystatekineticconstantsoftheGSTsfrom*L.migratoria*.

Enzyme	*K*_m_^CDNB^ (mM)	*K*_cat_^CDNB^ (s^−1^)	(*K*_cat_/*K*_m_)^CDNB^ (mM^−1^·s^−1^)	*K*_m_^GSH^ (mM)	*K*_cat_^GSH^ (S^−1^)		(*K*_cat_/*K*_m_)^GSH^ (mM^−1^·s^−1^)
LmGSTD1	0.139±0.008	9,842.92	70,740.17	0.507±0.025	42,664.26	84,142.21	
LmGSTD2	0.923±0.049	89,358.34	96,831.84	0.226±0.012	121,069.26	535,015.42	
LmGSTD3	0.102±0.010	99.08	976.10	1.977±0.042	426.19	215.52	
LmGSTD4	0.015±0.002	3,005.67	195,684.54	0.356±0.032	3,178.80	8936.70	
LmGSTD5	0.021±0.001	13,546.21	659,253.89	1.595±0.070	10,750.18	6,741.26	
LmGSTD6	0.528±0.301	12,675.08	24,023.99	1.031±0.069	35,147.86	34,096.99	
LmGSTD7	0.492±0.039	2,881.65	5,856.92	0.348±0.012	2,648.66	7,603.34	
LmGSTE1	0.007±0.001	13,741.51	1,937,102.02	1.404±0.099	41,709.13	29,702.53	
LmGSTE4	0.102±0.008	33,317.71	326,081.31	1.239±0.070	33,523.92	27,058.36	
LmGSTE5	0.026±0.002	8,356.89	324,916.19	1.961±0.058	9,271.29	4,728.43	
LmGSTS1	0.226±0.014	328.27	1,451.66	0.748±0.025	478.28	639.59	
LmGSTS2	1.047±0.092	1,124.79	1074.03	0.890±0.042	997.15	1,120.49	
LmGSTS3	0.217±0.023	2,030.00	9,339.88	0.569±0.020	3,818.57	6,711.74	
LmGSTS4	1.292±0.070	471.23	364.60	0.365±0.033	392.40	1,074.43	
LmGSTS5	0.253±0.026	6,673.54	26,333.24	0.555±0.046	14,499.79	26,138.66	
LmGSTS7	0.046±0.001	1,730.32	37,678.86	0.034±0.001	8,683.39	258,052.90	
LmGSTS8	0.100±0.006	84.84	850.60	0.742±0.028	251.30	338.75	
LmGSTS9	0.351±0.026	1,646.38	4,692.93	0.533±0.033	3,039.24	5,702.23	
LmGSTS10	0.311±0.018	2,228.91	7,172.99	1.547±0.076	6,555.24	4,236.67	
LmGSTT1	0.260±0.014	996.50	3,836.04	15.902±1.394	26286.68	1,653.10	
LmGSTT2	0.393±0.049	1,203.61	3,059.60	0.527±0.023	1,997.86	3,791.14	
LmGSTO1	0.228±0.045	32.65	143.01	5.186±0.476	114.43	27.85	
LmGSTO2	0.841±0.138	68.10	80.98	3.301±0.245	74.97	22.72	
LmGSTO3	0.216±0.004	221.38	1022.63	5.577±0.287	953.23	170.91	
LmGSTZ1	0.452±0.058	100.01	221.47	0.531±0.015	71.95	135.55	

The units are as follows: *V*_max_, µmol/min/mg; *k*_cat_, s^−1^; *K*_m_, mM; *k*_cat_/*K*_m_, mM^−1 s−1^.

The data are the means ± SD for at least three independent experiments.

The observed variations of enzyme catalytic properties not only reflect environmental changes in the GSTs active site, but also embody the diversity of the physiological substrates. This may partially explain why insects possess so many different GSTs.

### Positive selective sites of GSTs from *L. migratoria*

In order to detect the influence of selection in the delta GSTs, we used branch method to detect selective pressure [27]–[29]. Under the one-ratio model, the log-likelihood values were lnL = −4573.55, with estimates of ω = 0.10449 for the delta GSTs. Under the free-ratio model, the log-likelihood values were lnL = −4558.31 for the delta GSTs. The LRTs of the 2 assumptions indicated that the free-ratio model was significantly more likely (P<0.01) for the delta GSTs, implying that the selective pressure varied among branches. The estimated ω values in most branches of the delta GSTs were <1, indicating that the GST genes in the delta GST had been under purifying selection. The three tests, 0-A, 0-B, and 0-C, suggested that ω_A_, ω_B_ and ω_C_ were significantly greater than the background ratio ω_0_ (Table 4). While the other three tests, A-A′, B-B′, and C-C′, suggested that ω_A_, ω_B_ and ω_C_ were also significantly greater than one. These results suggested that the three major branches of delta class were under positive selection pressure.

**Table 4:** Likelihood ratio tests for delta GST genes under branch model.

	Model	-lnL	Model compared	χ2	P
0	One-ratio	4573.55			
1	Free-ratio	4558.31	0 and 1	30.48	<0.001^**^
	Two-ratio				
A	Branch A,	4570.59	0 and A	5.92	<0.05^*^
B	Branch B,	4566.61	0 and B	13.88	<0.001^**^
C	Branch C	4562.01	0 and C	23.08	<0.001^**^
A′	Branch A′, ω_A_ = 1	4585.05	A′ and A	28.92	<0.001^**^
B′	Branch B′, ω_B_ = 1	4631.91	B′ and B	130.60	<0.001^**^
C′	Branch C′, ω_C_ = 1	4637.80	C′ and C	151.58	<0.001^**^

Branch A (*LmGSTd7*), branch B (*LmGSTd1*and *LmGSTd5*), branch C (*LmGSTd2*, *LmGSTd3*, *LmGSTd4* and *LmGSTd6*). Phylogenetic analysis of delta GSTs from *L.migtatoria* are shown in S4 Figure. All data are present in S5 Data.

Our present results focused on whether the specific sites have undergone positive selection. Therefore, we chose 5 models of CODEML to detect positive selection sites. We conducted 2 LRTs, which compared M1a with M2a, and M7 with M8, to test whether each GST class had evolved under positive selection (Table 5 and S5 Data). The one-ratio model (M0) yielded estimated ω values of 10.449% for the delta GSTs; this result implied that, on average, negative selection played a dominant role during evolution. We further showed that the M8 model was considerably better than the M7 model for delta GSTs, and provided strong evidence for positive selection. Under the M8 model, 0.7% of delta sites were positively selected. Further, the results of BEB analysis revealed that 4 amino acid sites of the delta GSTs were identified as positive selection sites. Among the 4 candidate sites, 2 sites (I193 and T199) were located in the *C-*domain of GSTs, whereas the other 2 sites (V9 and G11) were located in the *N*-domain of GSTs.

**Table 5:** Parameter estimates and likelihood ratio tests for delta GST genes under site model.

Model	Estimates of parameters	-lnL	χ2	P	Positively Selected Sites	
M0	0.10449	4573.55				
M1a (nearlyneutral)	p_0_ = 0.81853, p_1_ = 0.18147	4530.57				
	ω_0_ = 0.06960, ω_1_ = 1.00000					
M2a (positiveselection)	p_0_ = 0.81853, p_1_ = 0.00226, p_2_ = 0.17920	4530.57	0	1.00		
	ω_0_ = 0.06960, ω_1_ = 1.00000, ω_2_ = 1.00000					
M7 (β)	P = 1.01633, *q* = 6.34930	4502.29			*N-*domain	V9
M8 (β&ω)	p_0_ = 0.97058, P = 1.12764, q = 8.41813	4499.13	6.32	<0.05^*^	*N-*domain	G11
	(p1 = 0.02942), ω = 105.37152				*C-*domain	I193
					*C-*domain	T199

Numbering is according to the amino sequence of LmGSTD1. All data are present in S5 Data.

**Figure 4:**
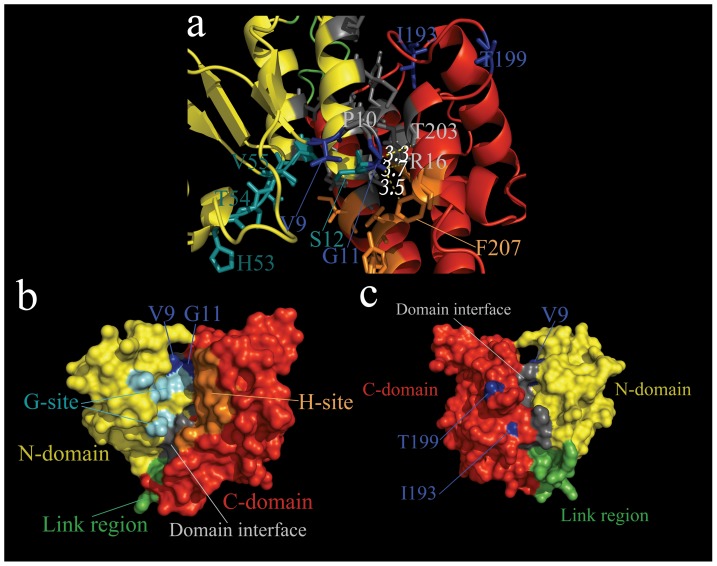
Homology structure of delta and sigma class GSTs from *L. migratoria*. **a**, Ribbon representation of the 3D-structures of LmGSTD1. **b**, **c** Surface representation of LmGSTD1 (**b** front, **c** back). *N*-domain, *C*-domain and link region are colored yellow, red and green, respectively. The glutathione binding site (G-site), the hydrophobic ligand binding site (H-site) and the positive selective sites are labeled with cyan, orange and blue, respectively. The residues located at the interface of the *N*- and *C*-domain are shown in gray. In order to conspicuously display structure details, the homology structure of LmGSTD1 were selected as representative structure of each class in ribbon and surface representation.

The results of protein 3-dimensional-structure modeling suggested that the overall structures of the delta GSTs, which are involved in positive selection pressure, were relatively conserved, especially regarding the β-sheet and α-helix (S3 Figure). Therefore, in order to clearly display detailed protein structures, we selected the homology structure of LmGSTD1 as a representative structure, and used the structure to label the positive selection sites (Fig. 4). We observed that most of the positive selection sites were situated either at the functional α-helix or at nearby essential sites. For instance, in delta GSTs, the V9 and G11 sites were close not only to the G-site, but also to the dimer interface. Further, the T199 site was close to the interface between the *N-* and *C*-domain residues.

## Discussion

In the present study, we identified and sequenced cDNAs putatively encoding 28 cytosolic GSTs and 4 microsomal GSTs based on the transcriptome database. Our phylogenetic analysis revealed that the 28 cytosolic GSTs belonging to 6 different cytosolic classes (sigma, epsilon, delta, omega, theta, and zeta), and the 4 microsomal GSTs belonging to 2 classes—class 1 (insect subclass) and class 3 (MGST3 subclass). The sigma class (10 members), delta class (7 members), epsilon class (5 members), and omega class (3 members) have apparently undergone gene expansion. However, it remains unclear whether these genes play different roles in different tissues and at different developmental stages. To explore these matters, we determined the gene expression patterns at the mRNA level, and the enzymatic properties at the protein level.

We observed 12 GSTs highly expressed during the egg stage. Among the GSTs that were highly expressed in the egg stage, some showed a high expression level in the early egg stage, others were primarily expressed in the late egg stage, and others were expressed in both the early and late stages (Fig. 3C). Because egg is a non-mobile and non-feeding stage, expression of these genes may imply their roles in metabolizing endogenous substances during the embryonic development. Other GSTs were mainly expressed in the larval and adult stages, and are therefore more likely responsible for detoxification in the larval and adult stages.

Tissue-specific expressed GSTs may play a vital part in the respective organs or tissues through their detoxification activity. For instance, the pi, rho, and omega GSTs of coho salmon (*Oncorhynchus kisutch*) are located in the olfactory rosettes and are critical to maintaining olfaction [30]. GSTM1 is situated in the mouse aorta, and regulates the proliferation and migration of vascular smooth muscle cells [31]. The expression level of omega GSTs in the human brain is associated with risk of Alzheimer's and Parkinson's disease [32]. The existence of differential gene expression profiles of locust GSTs suggests that their detoxification abilities are restricted within corresponding tissues or organs, according to physiological demands or regulation mechanisms *in vivo*. In the present study, we observed differences in the substrate specificities and steady kinetic constants of locust GSTs. The architecture of the functional region may have been altered by amino acid substitutions. The G-site residues of the *N*-terminal domain are known to be relatively conserved; on the other hand, the H-site residues of the *C*-terminal domain are relatively flexible, thereby contributing to adaptation to a wider spectrum of toxic reagents. Classic studies in the literature have paid more attention to the obvious functional changes caused by amino acid replacements in critical regions [33]. A single amino acid replacement located directly in the active site can markedly alter the substrate specificity of *N*-acetyl-l-ornithine transcarbamylase and *N*-succinyl-l-ornithine transcarbamylase [34]. Similarly, 6 substitutions located directly in the adenosine-binding pocket of *E. coli* isocitrate dehydrogenase systematically inverted coenzyme specificity [35]. The results of recent studies have shown that mutations located close to the active sites and other functional regions can alter the substrate preference or catalytic activity. In pine, tau GSTs contains positively selected residues located adjacent to the G-site or distant from the active center, and are related to the enzymatic activities [33]. Mutations of superoxide dismutase also indicated that residues located close to the active site can markedly alter the catalytic efficiency [36]. These observations imply that the positively selected residues of the delta GSTs, which are located close to essential catalytic residues, represent a mechanism for substrate diversity of the GSTs family [33]. The active sites of GSTs require specific amino acid residues and accurate orientation in protein structures, thereby resulting in greater functional and structural constraints. On the other hand, the protein surface region is generally relatively flexible. This may explain why the 4 positive selection sites of the delta class were spread on protein surface.

## Supporting Information

S1 Figure **Alignment of amino acid sequences of GSTs from** ***L. migratoria*** **.** The glutathione binding site (G-site), the hydrophobic ligand binding site (H-site) and the positive selective sites are labeled with green, red and blue square, respectively. The residues located at the interface of the N- and C-termianl domain and dimer interface are label with orange and pink square, respectively.
(PDF)

S2 Figure **Prediction of transmembrane segments of microsomal GSTs from** ***L. migratoria*** **.** **A**: *LmGSTm1*, **B**: *LmGSTm2,* **C**: *LmGSTm3,* **D**: *LmGSTm4*.
(TIF)

S3 Figure **Superimposed homology structure of delta class GSTs.** *N*-domain, *C*-domain and link region are colored yellow, red and green, respectively.
(TIF)

S4 Figure **Phylogenetic analysis of delta GSTs from** ***L.migtatoria*** **.** The bootstrap neighbor-joining tree was generated using MEGA from ClustalW alignments. Branch numbers represent bootstrap values (1000 replicates). Branch A, branch B and branch C are labeled with A, B, C
(TIF)

S1 Table **Primer sequence for Real-time PCR analysis.**
(DOC)

S2 Table **Comparison of GST gene number from** ***L. migratoria*** **and five insects.**
(DOC)

S1 Data **Analysis of** ***L. migratoria*** **transcriptome.**
(PDF)

S2 Data **Sequences of** ***L. migratoria*** **GSTs.**
(DOC)

S3 Data **Sequences of cytosolic GSTs used for phylogenetic analysis.**
(DOC)

S4 Data **Sequences of microsomal GSTs used for phylogenetic analysis.**
(DOC)

S5 Data **Output file from PAML.**
(RAR)

## References

[1] Yang ML, Zhang JZ, Zhu KY, Xuan T, Liu XJ, et al (2009) Mechanisms of organophosphate resistance in a field population of oriental migratory locust, *Locusta migratoria manilensis* (Meyen). Archives of Insect Biochemistry and Physiology 71:3–15. 18615705 10.1002/arch.20254

[2] Qin G, Jia M, Liu T, Zhang X, Guo Y, et al (2013) Characterization and Functional Analysis of Four Glutathione *S*-Transferases from the Migratory Locust, *Locusta migratoria* . PLoS ONE 8:e58410. 23505503 10.1371/journal.pone.0058410 PMC3591310

[3] Zhang X, Li T, Zhang J, Li D, Guo Y, et al (2012) Structural and catalytic role of two conserved tyrosines in delta-class glutathione *S*-transferase from *Locusta migratoria* . Archives of Insect Biochemistry and Physiology 80:77–91. 22581614 10.1002/arch.21025

[4] Qin G, Jia M, Liu T, Xuan T, Zhu K, et al (2011) Identification and characterisation of ten glutathione *S*-transferase genes from oriental migratory locust, *Locusta migratoria manilensis* (Meyen). Pest Management Science 67:697–704. 21413139 10.1002/ps.2110

[5] Li X, Zhang X, Zhang J, Zhang X, Starkey SR, et al (2009) Identification and characterization of eleven glutathione *S*-transferase genes from the aquatic midge *Chironomus tentans* (Diptera: Chironomidae). Insect Biochemistry and Molecular Biology 39:745–754. 19744561 10.1016/j.ibmb.2009.08.010

[6] Sheehan D, Meade G, Foley VM, Dowd CA (2001) Structure, function and evolution of glutathione transferases: implications for classification of non-mammalian members of an ancient enzyme superfamily. Biochem J 360:1–16. 11695986 10.1042/0264-6021:3600001 PMC1222196

[7] Yu Q, Lu C, Li B, Fang S, Zuo W, et al (2008) Identification, genomic organization and expression pattern of glutathione *S*-transferase in the silkworm, *Bombyx mori* . Insect Biochemistry and Molecular Biology 38:1158–1164. 19280710 10.1016/j.ibmb.2008.08.002

[8] Wang Y, Qiu L, Ranson H, Lumjuan N, Hemingway J, et al (2008) Structure of an insect epsilon class glutathione *S*-transferase from the malaria vector *Anopheles gambiae* provides an explanation for the high DDT-detoxifying activity. Journal of Structural Biology 164:228–235. 18778777 10.1016/j.jsb.2008.08.003

[9] Mounsey K, Pasay C, Arlian L, Morgan M, Holt D, et al (2010) Increased transcription of Glutathione *S*-transferases in acaricide exposed scabies mites. Parasites & Vectors 3:43. 20482766 10.1186/1756-3305-3-43 PMC2890653

[10] Qin G, Jia M, Liu T, Zhang X, Guo Y, et al (2012) Heterologous expression and characterization of a sigma glutathione *S*-transferase involved in carbaryl detoxification from oriental migratory locust, *Locusta migratoria manilensis* (Meyen). Journal of Insect Physiology 58:220–227. 22075389 10.1016/j.jinsphys.2011.10.011

[11] Yu X, Sun R, Yan H, Guo X, Xu B (2012) Characterization of a sigma class glutathione *S*-transferase gene in the larvae of the honeybee (*Apis cerana cerana*) on exposure to mercury. Comparative Biochemistry and Physiology Part B: Biochemistry and Molecular Biology 161:356–364. 10.1016/j.cbpb.2011.12.009 22248933

[12] Ren H, Xu D, Gopalakrishnan S, Qiao K, Huang W-B, et al (2009) Gene cloning of a sigma class glutathione *S*-transferase from abalone (*Haliotis diversicolor*) and expression analysis upon bacterial challenge Developmental & Comparative Immunology. 33:980–990. 10.1016/j.dci.2009.04.003 19414031

[13] Flanagan JU, Smythe ML (2011) Sigma-class glutathione transferases. Drug Metabolism Reviews 43:194–214. 21425928 10.3109/03602532.2011.560157

[14] Board PG, Anders MW (2011) Glutathione transferase zeta: discovery, polymorphic variants, catalysis, inactivation, and properties of Gstz1^-/-^ mice. Drug Metabolism Reviews 43:215–225. 21303221 10.3109/03602532.2010.549132

[15] Bresell A, Weinander R, Lundqvist G, Raza H, Shimoji M, et al (2005) Bioinformatic and enzymatic characterization of the MAPEG superfamily. FEBS Journal 272:1688–1703. 15794756 10.1111/j.1742-4658.2005.04596.x

[16] Frova C (2006) Glutathione transferases in the genomics era: New insights and perspectives. Biomolecular Engineering 23:149–169. 16839810 10.1016/j.bioeng.2006.05.020

[17] Siritantikorn A, Johansson K, Åhlen K, Rinaldi R, Suthiphongchai T, et al (2007) Protection of cells from oxidative stress by microsomal glutathione transferase 1. Biochemical and Biophysical Research Communications 355:592–596. 17306223 10.1016/j.bbrc.2007.02.018

[18] Johansson K, Järvliden J, Gogvadze V, Morgenstern R (2010) Multiple roles of microsomal glutathione transferase 1 in cellular protection: A mechanistic study. Free Radical Biology and Medicine 49:1638–1645. 20727966 10.1016/j.freeradbiomed.2010.08.013

[19] Kojima F, Kato S, Kawai S (2005) Prostaglandin E synthase in the pathophysiology of arthritis. Fundamental & Clinical Pharmacology 19:255–261. 15910650 10.1111/j.1472-8206.2005.00316.x

[20] Yamada T, Takusagawa F (2007) PGH2 Degradation Pathway Catalyzed by GSH−Heme Complex Bound Microsomal Prostaglandin E2 Synthase Type 2: The First Example of a Dual-Function Enzyme. Biochemistry 46:8414–8424. 17585783 10.1021/bi700605m

[21] Schröder O, Sjöström M, Qiu H, Stein J, Jakobsson P-J, et al (2003) Molecular and catalytic properties of three rat leukotriene C4 synthase homologs. Biochemical and Biophysical Research Communications 312:271–276. 14637132 10.1016/j.bbrc.2003.10.115

[22] Eswar N, Webb B, Marti-Renom MA, Madhusudhan MS, Eramian D, et al. (2001) Comparative Protein Structure Modeling Using MODELLER. Current Protocols in Protein Science. 10.1002/0471140864.ps0209s50 18429317

[23] Yang Z (2007) PAML 4: Phylogenetic Analysis by Maximum Likelihood. Molecular Biology and Evolution 24:1586–1591. 17483113 10.1093/molbev/msm088

[24] Yang Z, Nielsen R, Goldman N, Pedersen A-MK (2000) Codon-Substitution Models for Heterogeneous Selection Pressure at Amino Acid Sites. Genetics 155:431–449. 10790415 10.1093/genetics/155.1.431 PMC1461088

[25] Yang Z, Wong WSW, Nielsen R (2005) Bayes Empirical Bayes Inference of Amino Acid Sites Under Positive Selection. Molecular Biology and Evolution 22:1107–1118. 15689528 10.1093/molbev/msi097

[26] Wang X, Fang X, Yang P, Jiang X, Jiang F, et al (2014) The locust genome provides insight into swarm formation and long-distance flight. Nat Commun 5. 10.1038/ncomms3957 PMC3896762 24423660

[27] Shen T, Xu S, Wang X, Yu W, Zhou K, et al (2012) Adaptive evolution and functional constraint at TLR4 during the secondary aquatic adaptation and diversification of cetaceans. BMC Evolutionary Biology 12:39. 22443485 10.1186/1471-2148-12-39 PMC3384459

[28] Yang Z (1998) Likelihood ratio tests for detecting positive selection and application to primate lysozyme evolution. Molecular Biology and Evolution 15:568–573. 9580986 10.1093/oxfordjournals.molbev.a025957

[29] Osorio D, Antunes A, Ramos M (2007) Structural and functional implications of positive selection at the primate angiogenin gene. BMC Evolutionary Biology 7:167. 17883850 10.1186/1471-2148-7-167 PMC2194721

[30] Espinoza HM, Shireman LM, McClain V, Atkins W, Gallagher EP (2013) Cloning, expression and analysis of the olfactory glutathione *S*-transferases in coho salmon. Biochemical Pharmacology 85:839–848. 23261526 10.1016/j.bcp.2012.11.018 PMC3660137

[31] Yang Y, Parsons KK, Chi L, Malakauskas SM, Le TH (2009) Glutathione *S*-Transferase-µ1 Regulates Vascular Smooth Muscle Cell Proliferation, Migration, and Oxidative Stress. Hypertension 54:1360–1368. 19822795 10.1161/HYPERTENSIONAHA.109.139428 PMC2783903

[32] Mariet A, Fanggeng Z, High C, Curtis SY, Richard M, et al (2012) Glutathione *S*-transferase omega genes in Alzheimer and Parkinson disease risk, age-at-diagnosis and brain gene expression: an association study with mechanistic implications. Molecular Neurodegeneration 7:13–13. 22494505 10.1186/1750-1326-7-13 PMC3393625

[33] Lan T, Wang X, Zeng Q (2013) Structural and Functional Evolution of Positively Selected Sites in Pine Glutathione S-Transferase Enzyme Family. Journal of Biological Chemistry 288:24441–24451. 23846689 10.1074/jbc.M113.456863 PMC3750144

[34] Shi D, Yu X, Cabrera-Luque J, Chen TY, Roth L, et al (2007) A single mutation in the active site swaps the substrate specificity of *N*-acetyl-L-ornithine transcarbamylase and *N*-succinyl-L-ornithine transcarbamylase. Protein Science 16:1689–1699. 17600144 10.1110/ps.072919907 PMC2203365

[35] Chen R, Greer A, Dean AM (1995) A highly active decarboxylating dehydrogenase with rationally inverted coenzyme specificity. Proceedings of the National Academy of Sciences 92:11666–11670. 10.1073/pnas.92.25.11666 PMC40463 8524825

[36] Zhou H, Wong K, Vijayakumar M (1997) Design of fast enzymes by optimizing interaction potential in active site. Proceedings of the National Academy of Sciences 94:12372–12377. 10.1073/pnas.94.23.12372 PMC24950 9356456

