# Supplementary figures and images for: Molecular Cloning, Characterization and Positively Selected Sites of the Glutathione S-Transferase Family from Locusta migratoria
Source: PLoS One. 2014 Dec 8;9(12):e114776. doi: 10.1371/journal.pone.0114776 (PMC4259467; doi:10.1371/journal.pone.0114776)

A

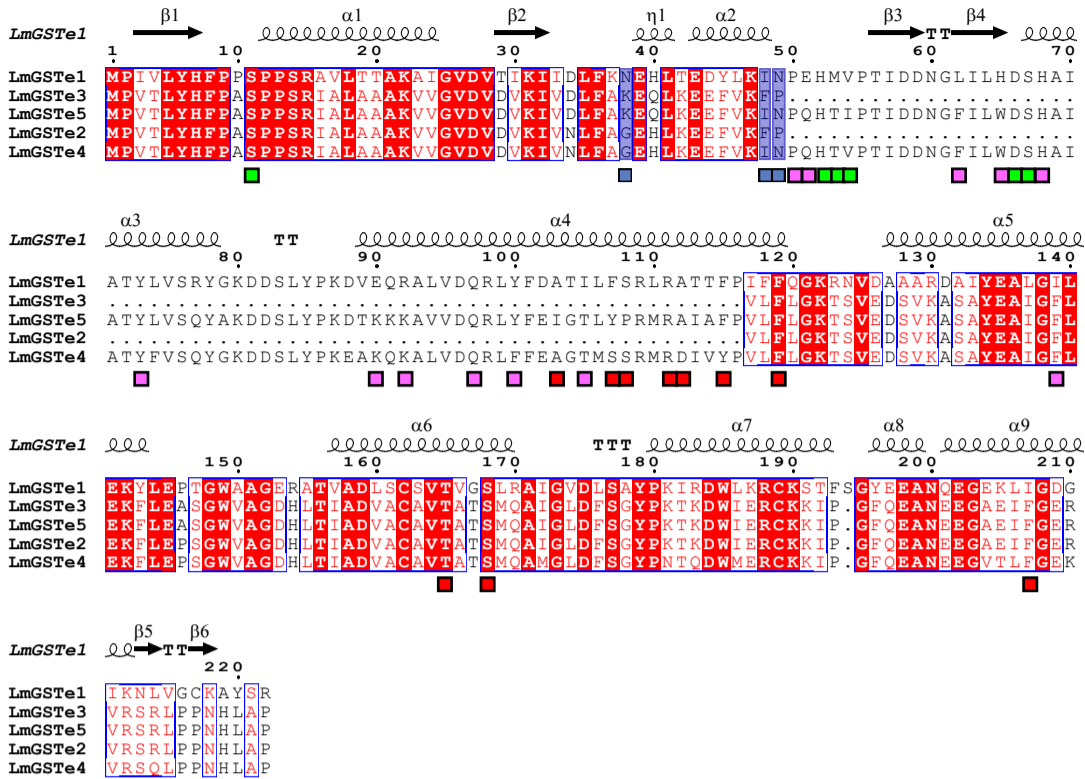

B

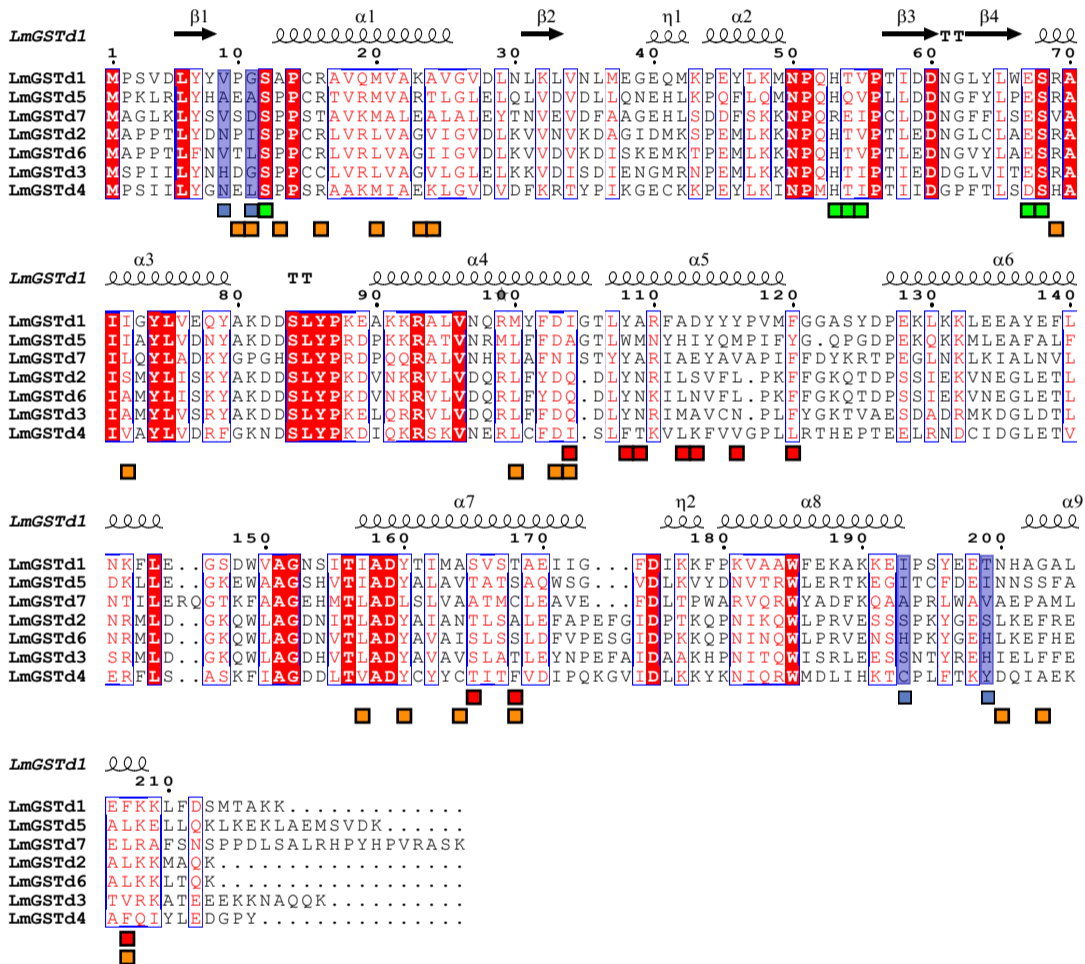

C

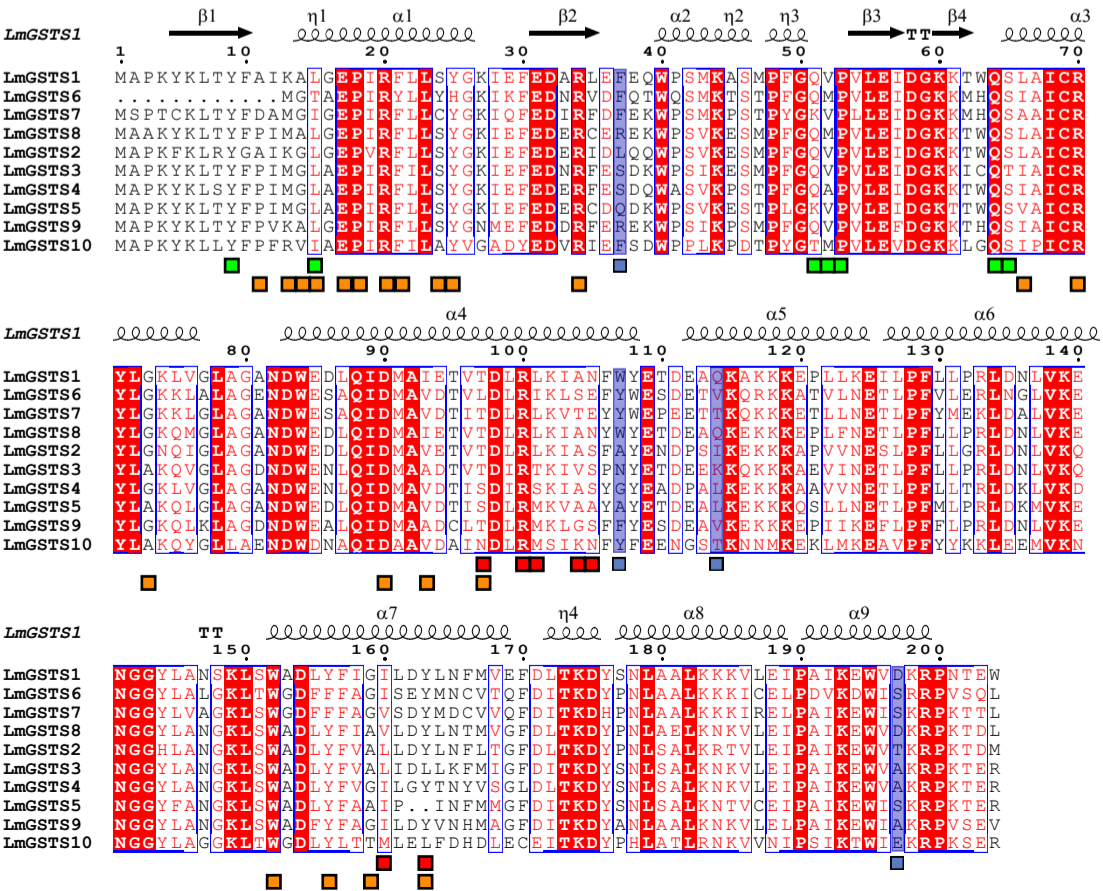

Supplement: S1 Figure — Alignment of amino acid sequences of GSTs from L. migratoria . The glutathione binding site (G-site), the hydrophobic ligand binding site (H-site) and the positive selective sites are labeled with green, red and blue square, respectively. The residues located at the interface of the N- and C-termianl domain and dimer interface are label with orange and pink square, respectively. (PDF) [file pone.0114776.s001.pdf]

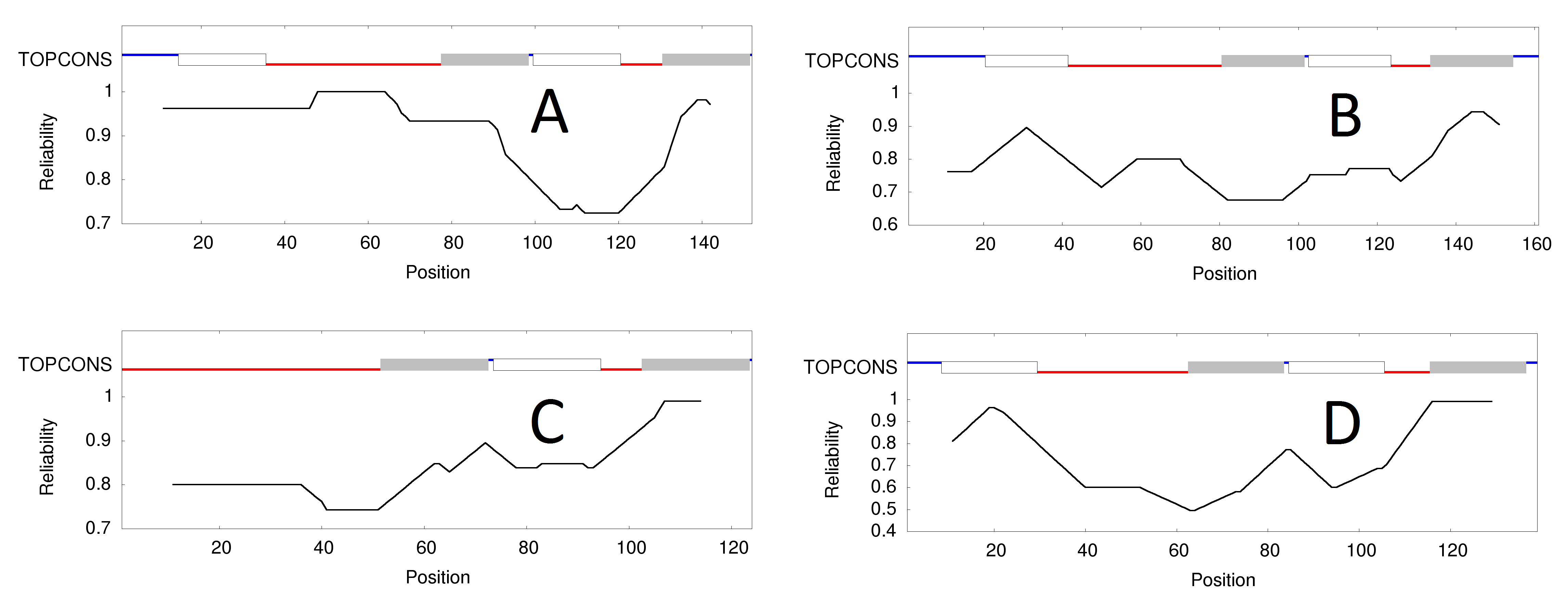

Supplement: S2 Figure — Prediction of transmembrane segments of microsomal GSTs from L. migratoria . A: LmGSTm1, B: LmGSTm2, C: LmGSTm3, D: LmGSTm4. (TIF) [file pone.0114776.s002.tif]

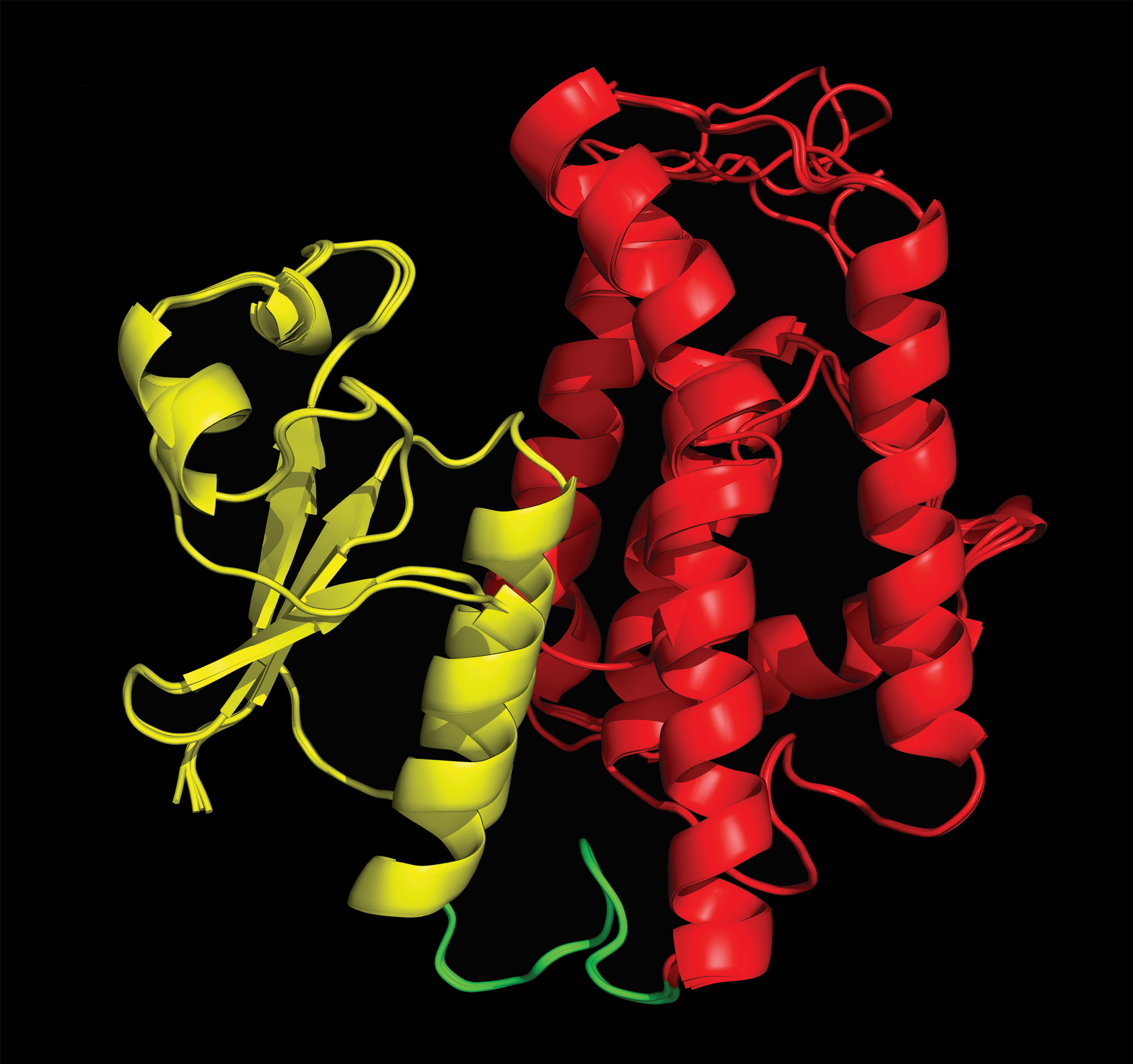

Supplement: S3 Figure — Superimposed homology structure of delta class GSTs. N-domain, C-domain and link region are colored yellow, red and green, respectively. (TIF) [file pone.0114776.s003.tif]

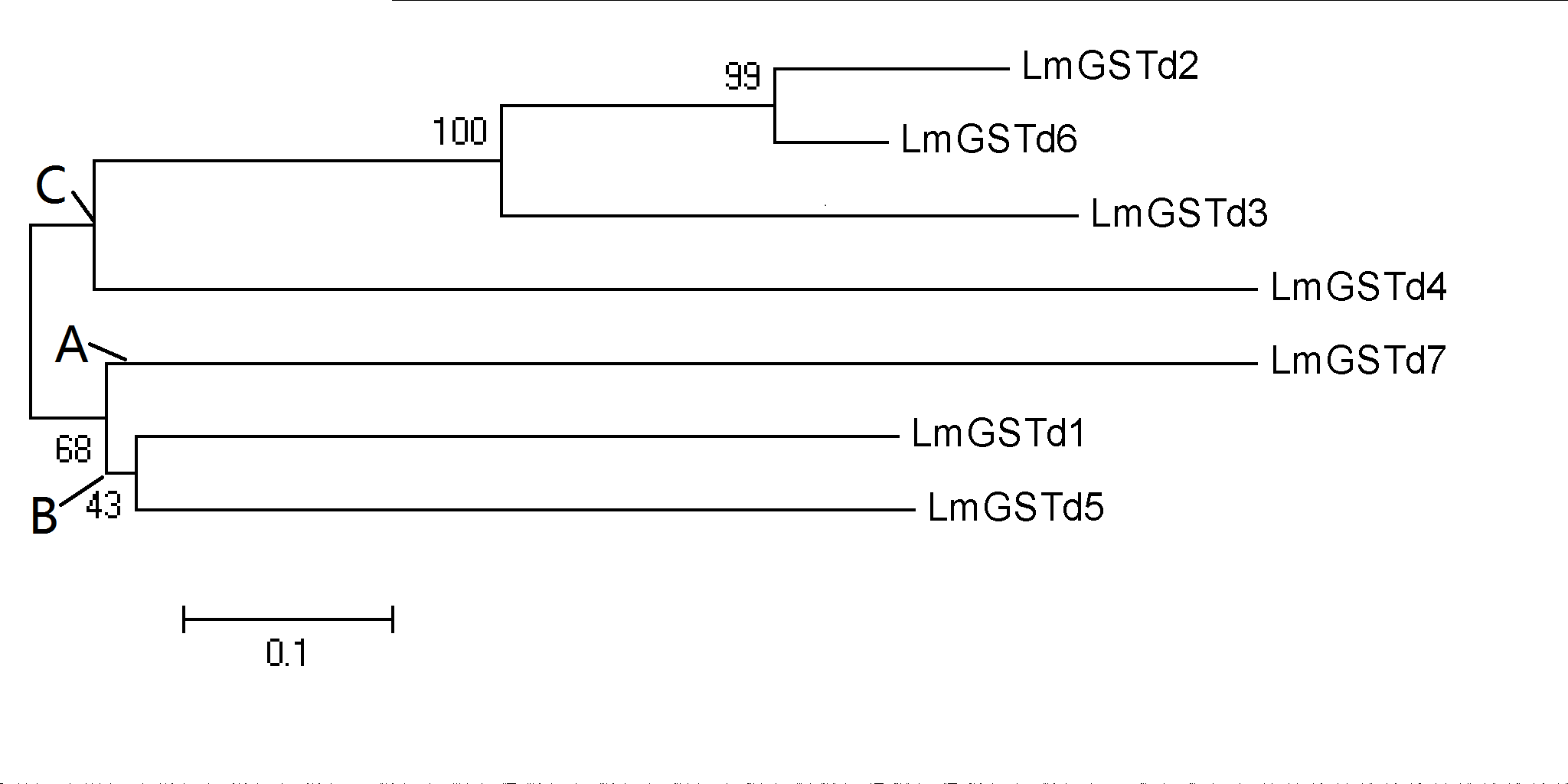

Supplement: S4 Figure — Phylogenetic analysis of delta GSTs from L.migtatoria . The bootstrap neighbor-joining tree was generated using MEGA from ClustalW alignments. Branch numbers represent bootstrap values (1000 replicates). Branch A, branch B and branch C are labeled with A, B, C (TIF) [file pone.0114776.s004.tif]
