# Supplementary material for: Molecular Cloning, Characterization and Positively Selected Sites of the Glutathione S-Transferase Family from Locusta migratoria
Source: PLoS One. 2014 Dec 8;9(12):e114776. doi: 10.1371/journal.pone.0114776 (PMC4259467; doi:10.1371/journal.pone.0114776)
Supplement: S1 Table — Primer sequence for Real-time PCR analysis. (DOC) [file pone.0114776.s005.doc]

**Table S1 Primer sequence for Real-time PCR analysis**

| Name | Primer sequence（5’-3’） |
| --- | --- |
| *LmGSTs1* | F/R: CCCGCGCTAGCAGCTTGATTT; ACCCAGAGCCTTAATAGCGAAGTATGTT |
| *LmGSTs2* | F/R: GTAAGCGAAACAGCCGAGAGC; CGTACTGGTTCACCCAGACCCT |
| *LmGSTs3* | F/R: GCACTGATTGATCTTCTCAAGTTCA; GTGAATGATTAAATACAGAATAATTCAGACAT |
| *LmGSTs4* | F/R: GCTACACCAACTACGTAAGTGGACTAGA; CTAGAAAGACATATATTTTGCACGAATC |
| *LmGSTs5* | F/R: AGACACTCCACACCGAGCTTTGCCT; GGCCCATTATAGGGAAGTAAGTT |
| *LmGSTs6* | F/R: TCTAGCAGCGCTAGGTCCAGCTAACT; CTGCCAAGTCTGGAAATCAACACGA |
| *LmGSTs7* | F/R: CCGCTAGTGGGCAAACTCAGAA; GTGACATCTTGTAACTGCTTTAATTTCTAGATAAA |
| *LmGSTs8* | F/R: CAGTGCTAGATTATCTAAACACCATG; TTAATCAGAACAGGGACACACAA |
| *LmGSTs9* | F/R: GCTGGAATATTGGACTATGTTAAT; ACAAATATGACAGATCATCTTGAAT |
| *LmGSTs10* | F/R: CTACTTTATTTTCCATTCAGAGTCA; CATAATCAGCTCCAACATATGCTAA |
| *LmGSTo1* | F/R: TGCCAAGGTCCCCAGCATCATC**;** CGCGGCGCCTAGACGTTGTCGTAGT |
| *LmGSTo2* | F/R: GGTGATACACTTTATGAAAGCCTAATAAT**;** TGTGTGGTAATAAACCTTTAGCATTG |
| *LmGSTo3* | F/R: GTACAGGCAGGGCACTCTGGACTT**;** TATATTCAATAGTTGACGTCTCTCAGTTAGG |
| *LmGSTd1* | F/R: GTGAACAGATGAAGCCAGAGTA; GTCGAAATACATTCTTTGGTTC |
| *LmGSTd2* | F/R: CCGCCACGCCCGACAGAAACTA; TGCCAGCATCCTTGACATTCACCACT |
| *LmGSTd3* | F/R: CAGAGCCGAAAATGTCACCAAT; CACCAGCTACCAGACGAACAAG |
| *LmGSTd4* | F/R: GAGTAGAGGTCCAACCACAAAT; AAAGGACATACAGAAACGCTCA |
| *LmGSTd5* | F/R: CGATTATAGCGTACCTGGTGGA; TGTTGACAGTTGCCCTCTTCTTA |
| *LmGSTd6* | F/R: ACGCCACATCTGAAACAGCAAT; ATGATACCGGCCACTAAACGGA |
| *LmGSTd7* | F/R: CTGAGTGAAAGTGTCGCCATCCT; ACCGCATACTCCGCTATTCGTG |
| *LmGSTe1* | F/R：TCTCCCGTCTAAGGGCCACAACT**;** CAGGGCCTCGTATATCGCATCT |
| *LmGSTe2* | F/R：ATCGTCAATCTATTCGCAGGGGAG**;** TCCCAGGAACAGCACCGGAAACTT |
| *LmGSTe3* | F/R：CGATGCCAGTCACCCTCTACCA**;** AACAGCACCGGGAACTTGACAAATT |
| *LmGSTe4* | F/R: GGACCAGAGATTGTTCTTCGAGGCG**;** TCCTCCACGCTCGTCTTTCCCA |
| *LmGSTe5* | F/R: CGATCAGAGACTGTACTTCGAGATAGGGACGC; GCCAGCCACCCAGCCCGAAGC |
| *LmGSTt1* | F/R: ATTTCAACTGAGGATCATTATTCTC**;** CATAAGGGATGTCATTTGCCAACAA |
| *LmGSTt2* | F/R: CATAATGACTGCCTGAACTGGAC**;** TGTAGTTTGTGCTTCTGTTTTGAAAT |
| *LmGSTz1* | F/R: TCTAACCAACCAGATTGCCCACC；ACATGAAGACTCTAATCCCTTAA |
| *LmGSTm1* | F/R: GTCAAGGACGCTGACGAGA; ATAACTCCGCAGCAGCCAT |
| *LmGSTm2* | F/R: CTGCCTCATTTAGCTTACC; AACCTTGATACCCTTCCAC |
| *LmGSTm3* | F/R: GCGACTACGCCTACTACAGC; ATTTTGCATCTTCAGGGTTCAG |
| *LmGSTm4* | F/R: TACTAACAAATCCACCTCCAG; ATAAGCCAACCAACAAAGCAA |
