# Supplementary material for: Molecular Cloning, Characterization and Positively Selected Sites of the Glutathione S-Transferase Family from Locusta migratoria
Source: PLoS One. 2014 Dec 8;9(12):e114776. doi: 10.1371/journal.pone.0114776 (PMC4259467; doi:10.1371/journal.pone.0114776)
Supplement: S2 Table — Comparison of GST gene number from L. migratoria and five insects. (DOC) [file pone.0114776.s006.doc]

**Table S2 Comparison of GST gene number from *L. migratoria*** and five insects

| GST | *T.castaneum* | *D.melanogaster* | *A.gambiae* | *A.mellifera* | *B. mori* | *L. migratoria* |
| --- | --- | --- | --- | --- | --- | --- |
| Delta | 3 | 16 | 11 | 17(12) | 5 | 7 |
| Epsilon | 19 | 1 | 14 | 8 | 8 | 5 |
| Omega | 3 | 2 | 4(5) | 1 | 4 | 3 |
| Sigma | 7 | 6 | 1 | 1 | 2 | 10 |
| Theta | 1 | 2 | 4 | 2 | 1 | 2 |
| Zeta | 1 | 0 | 2 | 1 | 2 | 1 |
| MAPEG | 5 | 2 | 3(1) | 3 | 0 | 4 |
| Unkonwn | 2 | 3 | 1(0) | 2(3) | 1 | 0 |
| Total | 41 | 32 | 40 | 35 | 23 | 32 |

The numbers of GSTs from the above five model insect are cited from the reference.

1. Shi H, Pei L, Gu S, Zhu S, Wang Y, Zhang Y, Li B**: Glutathion*e* S-transferase (GST) genes in the red flour beetle*, Tribolium castane*um, and comparative analysis with five additional insec**ts*. Genomic*s 2012**, 1**00(5):327-335.
