# Supplementary material for: Molecular Cloning, Characterization and Positively Selected Sites of the Glutathione S-Transferase Family from Locusta migratoria
Source: PLoS One. 2014 Dec 8;9(12):e114776. doi: 10.1371/journal.pone.0114776 (PMC4259467; doi:10.1371/journal.pone.0114776)
Supplement: S1 Data — Analysis of L. migratoria transcriptome. (PDF) [file pone.0114776.s007.pdf]

BGI Transcriptome Assembly Analysis Report

Output Statistics

The output of sequenced data is an important indicator of the contract. Clean reads in each sample must contain a total base number of no less than the contractually required output. The completion of this work is listed in the table below:

Output statistics of sequencing

Samples	Total Raw Reads	Total Clean Reads	Total Clean Nucleotides (nt)	Q20 percentage	N percentage	GC percentage
N2	59,129,922	53,559,770	4,820,379,300	97.42%	0.00%	46.44%

* Total Clean Nucleotides = Total Clean Reads1 x Read1 size + Total Clean Reads2 x Read2 size

Assembly Results

Statistics of assembly quality

Statistics of assembly quality

	Sample	Total Number	Total Length(nt)	Mean Length(nt)	N50	Total Consensus Sequences	Distinct Clusters	Distinct Singletons
Contig	N2	188,554	49,092,957	260	352	-	-	-
Unigene	N2	84,641	41,542,996	491	692	84,641	13,352	71,289

Contig

Data: N2-Contig.length.txt

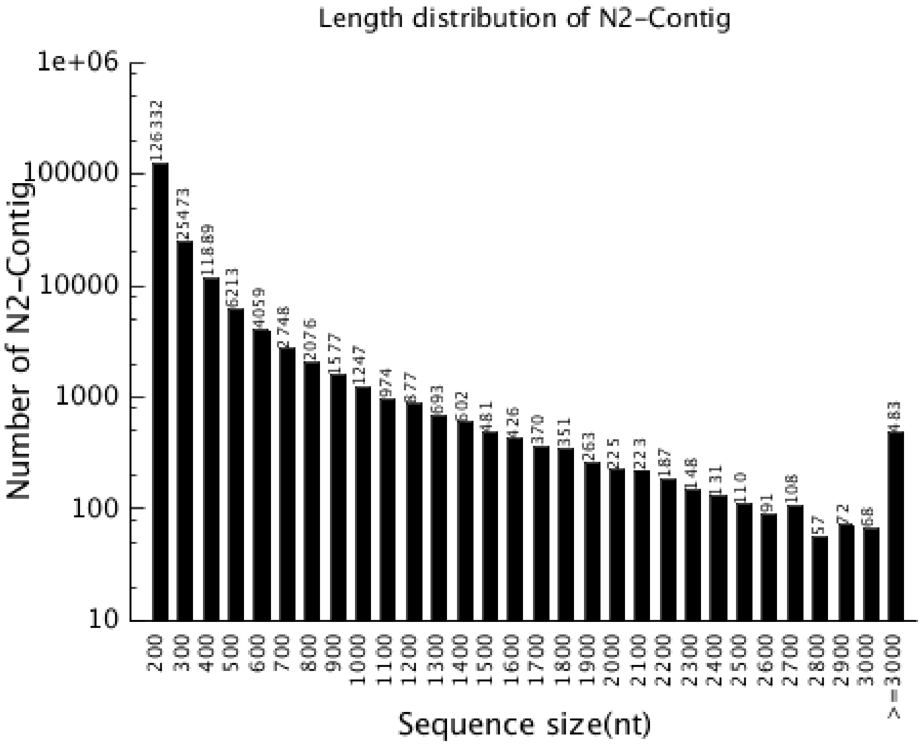

Unigene

Data: N2-Unigene.length.txt

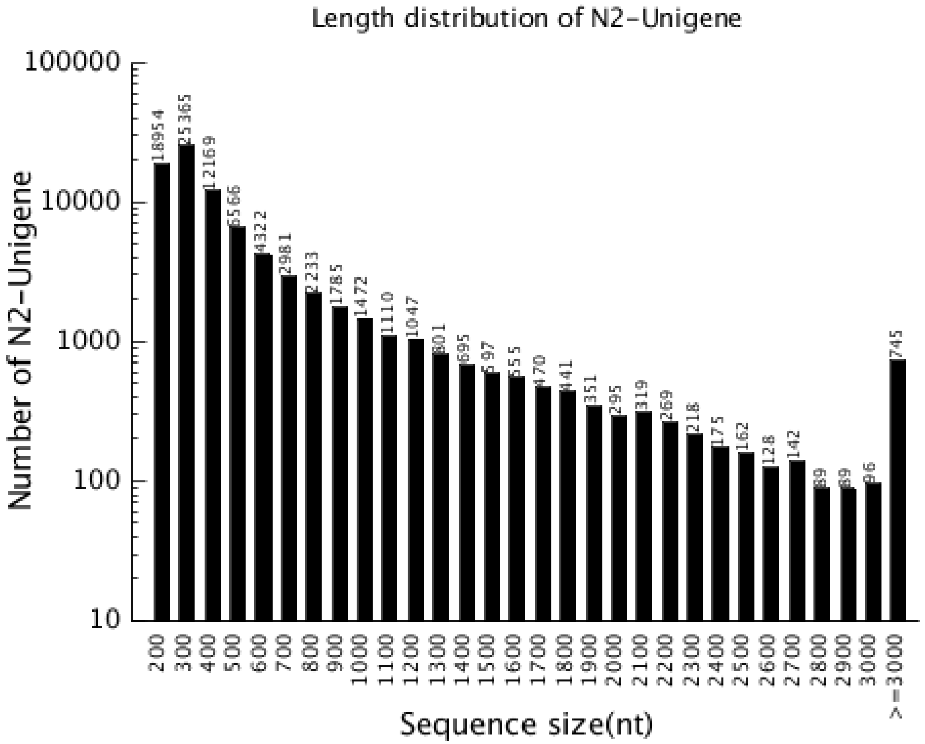

Sequence Files

Contig

1. N2

Unigene

1. N2
2. Unigene sequence from integrated samples

Unigene Function Annotation

Annotation analysis provides information of gene expression and functional annotation of All-Unigene in each sample. Functional annotation consists of protein functional annotaiton, Pathway annotation, COG functional annotation and Gene Ontology (GO) functional annotation.

Summary of annotation results

- annotation.xls

Unigenes were annotated with the databases of NR, NT, SwissProt, KEGG, COG and GO. Then counted the number of unigenes annotated with each database. The result is summaried as the follow table.

Statistics of annotation results

Sequence File	NR	NT	SwissProt	KEGG	COG	GO	ALL
N2-Unigene.fa	26,696	12,130	21,327	18,674	9,160	13,304	29,289

NR

Figure of NR classifacation

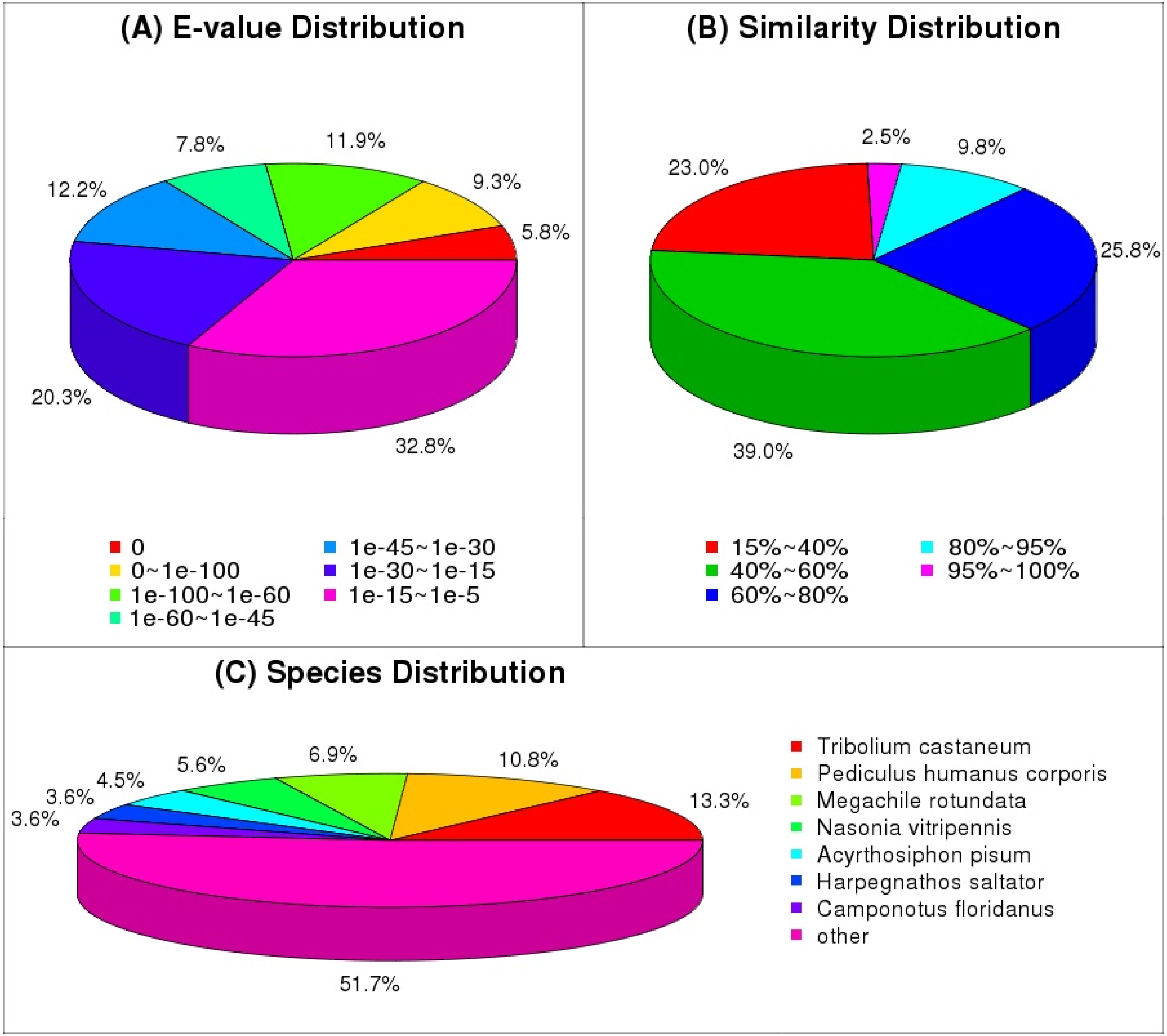
(A) E-value Distribution
7.8%
11.9%
12.2%
9.3%
5.8%
20.3%
32.8%
0
0~1e-100
1e-100~1e-60
1e-60~1e-45
1e-45~1e-30
1e-30~1e-15
1e-15~1e-5
(B) Similarity Distribution
2.5%
9.8%
23.0%
25.8%
39.0%
15%~40%
40%~60%
60%~80%
80%~95%
95%~100%
(C) Species Distribution
6.9%
10.8%
5.6%
4.5%
3.6%
3.6%
13.3%
51.7%
Tribolium castaneum
Pediculus humanus corporis
Megachile rotundata
Nasonia vitripennis
Acyrthosiphon pisum
Harpegnathos saltator
Camponotus floridanus
other

Figure of NR classifacation

- (A) Figure of E-value distribution: evalue_statistic.xls
- (B) Figure of identity distribution: similarity_statistic.xls
- (C) Figure of species distribution: species_statistic.xls

COG

Figure of COG classifacation

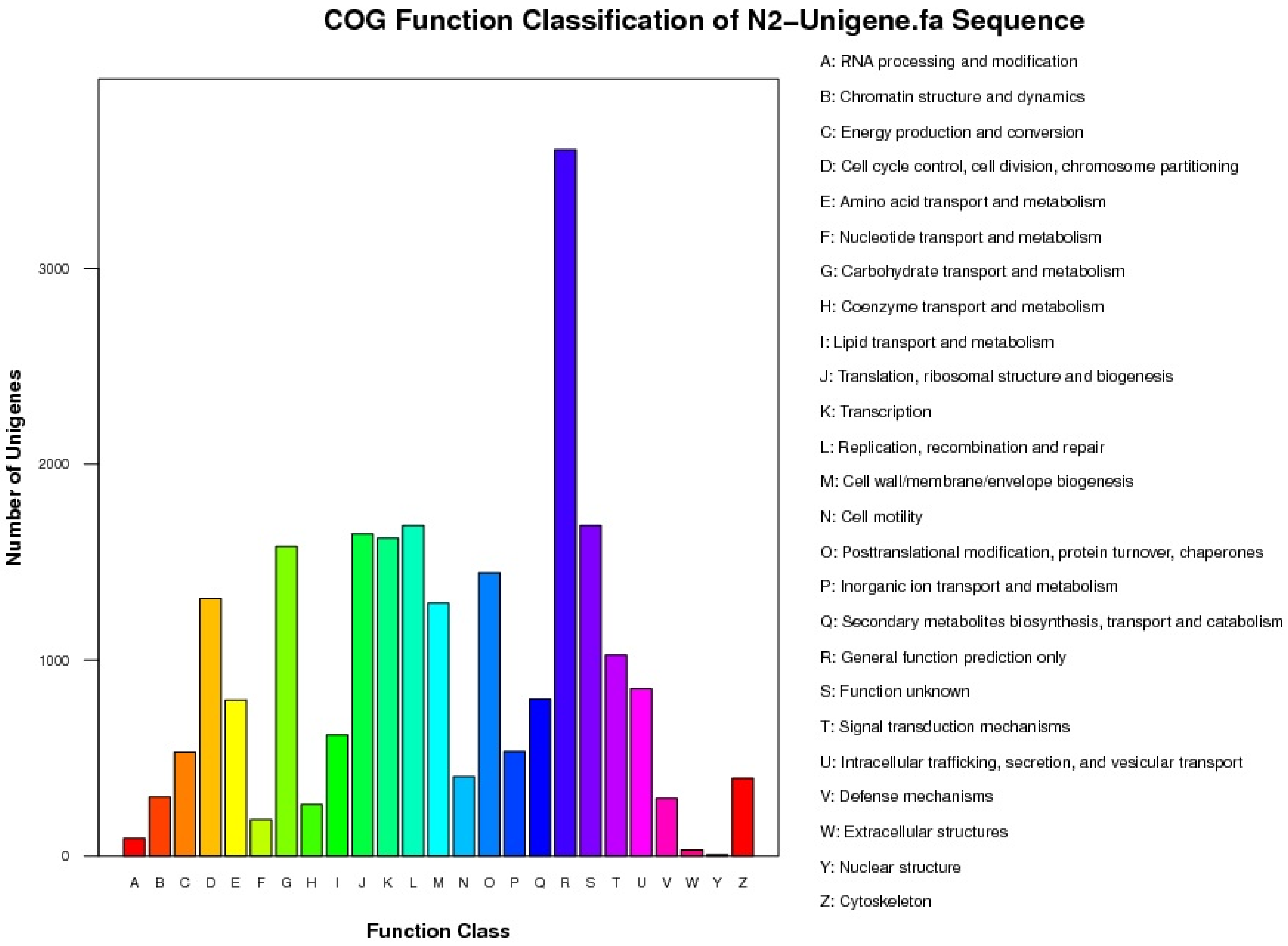
COG Function Classification of N2−Unigene.fa Sequence
A: RNA processing and modification
B: Chromatin structure and dynamics
C: Energy production and conversion
D: Cell cycle control, cell division, chromosome partitioning
E: Amino acid transport and metabolism
F: Nucleotide transport and metabolism
G: Carbohydrate transport and metabolism
H: Coenzyme transport and metabolism
I: Lipid transport and metabolism
J: Translation, ribosomal structure and biogenesis
K: Transcription
L: Replication, recombination and repair
M: Cell wall/membrane/envelope biogenesis
N: Cell motility
O: Posttranslational modification, protein turnover, chaperones
P: Inorganic ion transport and metabolism
Q: Secondary metabolites biosynthesis, transport and catabolism
R: General function prediction only
S: Function unknown
T: Signal transduction mechanisms
U: Intracellular trafficking, secretion, and vesicular transport
V: Defense mechanisms
W: Extracellular structures
Y: Nuclear structure
Z: Cytoskeleton
Number of Unigenes
3000
2000
1000
0
A B C D E F G H I J K L M N O P Q R S T U V W Y Z
Function Class

Data of figure of COG classifacation

- N2-Unigene.fa.cog.class.annot.xls

COG annotation file

- N2-Unigene.fa.cog.gene.annot.xls

Unigene GO Classification

Figure of GO classification

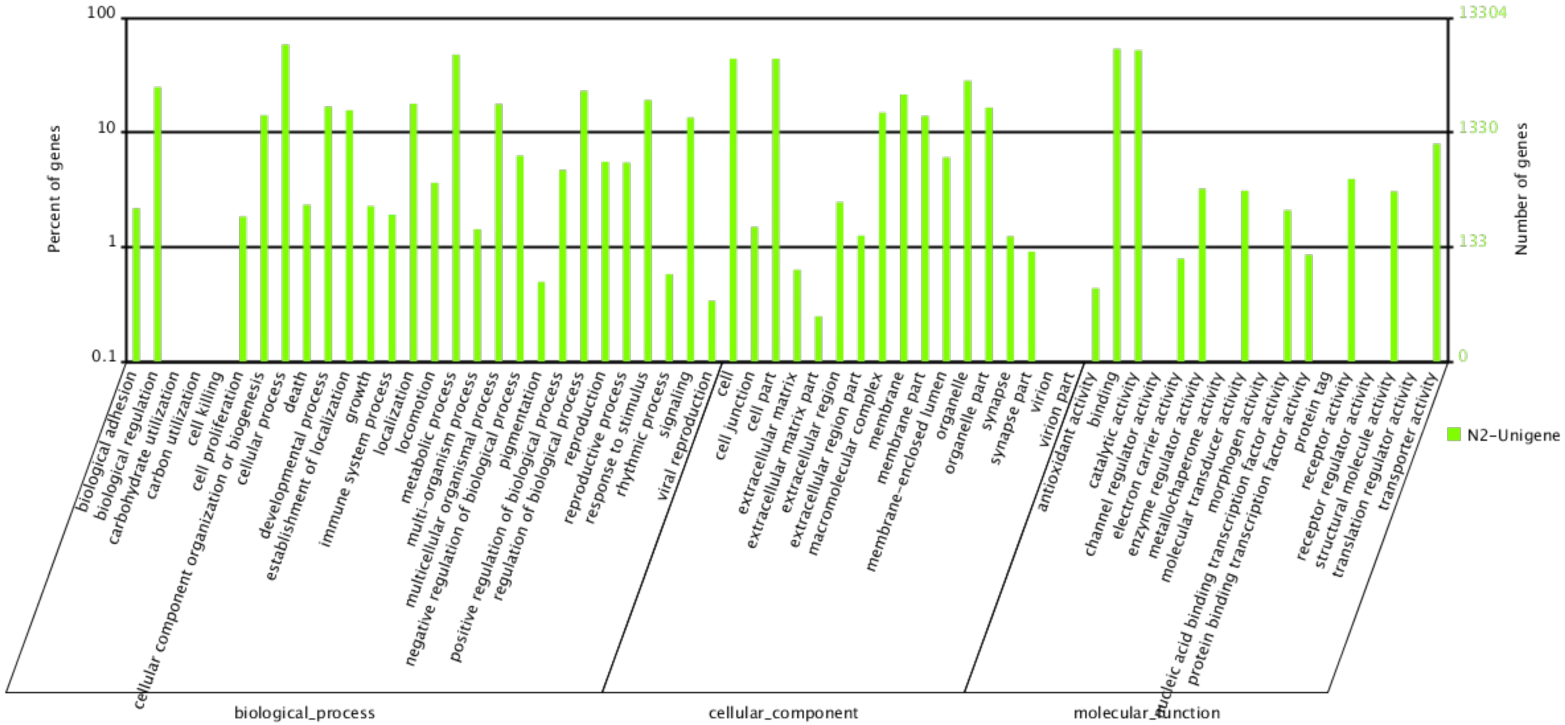

Data of figure of GO classifacation

- N2-Unigene.fa.GO2gene.xls

GO annotation file

- N2-Unigene.fa.gene2GO.xls

Unigene Metabolic Pathway Analysis

Pathway

- N2-Unigene.fa (File)

Protein Coding Region Prediction (CDS)

CDS nucleotide sequence

- N2-Unigene.blast

Data: N2-Unigene.blast.cds.fa.length.txt

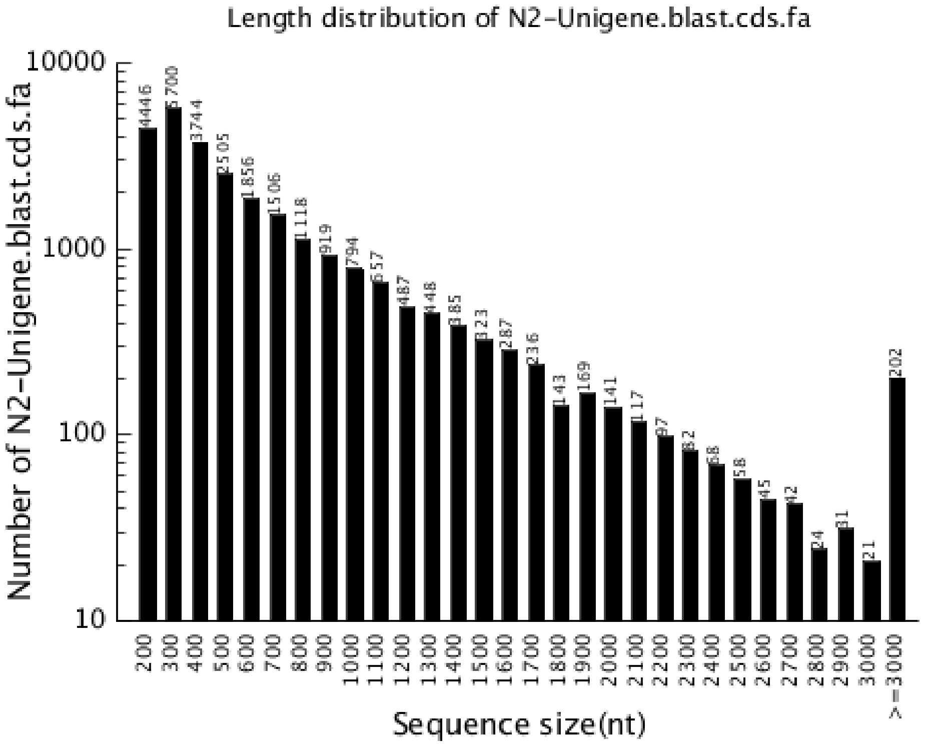

- N2-Unigene.ESTscan

Data: N2-Unigene.ESTscan.cds.fa.length.txt

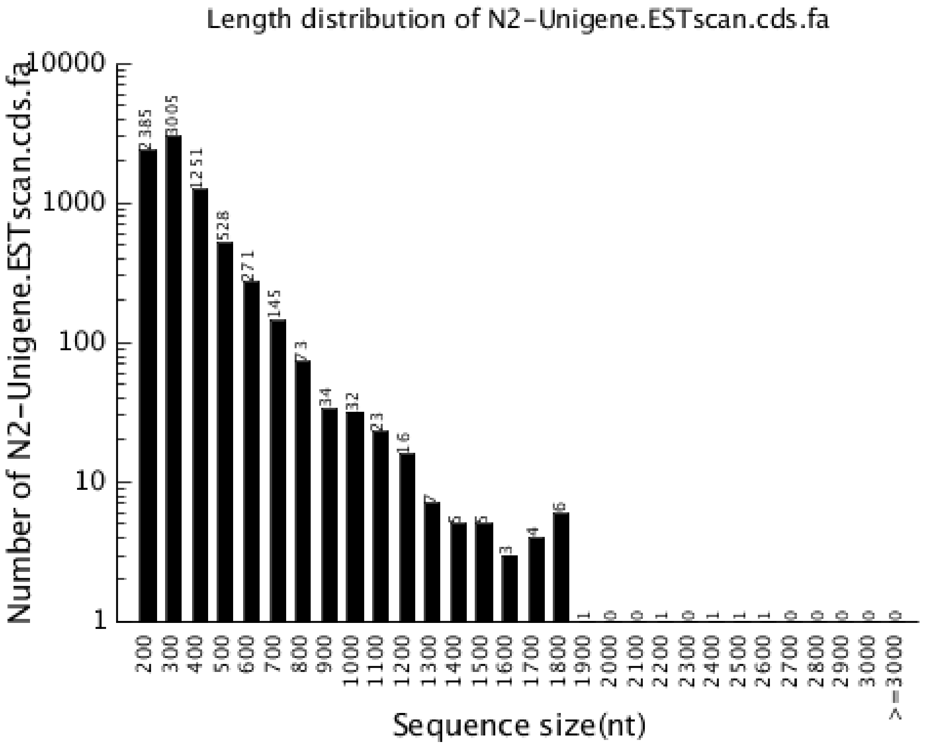

CDS protein sequence

- N2-Unigene.blast
- N2-Unigene.ESTscan
