# Supplementary material for: Molecular Cloning, Characterization and Positively Selected Sites of the Glutathione S-Transferase Family from Locusta migratoria
Source: PLoS One. 2014 Dec 8;9(12):e114776. doi: 10.1371/journal.pone.0114776 (PMC4259467; doi:10.1371/journal.pone.0114776)
Supplement: S2 Data — Sequences of L. migratoria GSTs. (DOC) [file pone.0114776.s008.doc]

>LmGSTo1

ATGAGCACCAAGCACCTCAAGAAGGGCGACCCGCAGCCGGCGGCCGCGGAGGGCGGCAAGCTGCGGCTGTACTCGATGGCGTTCTGCCCGTACGCGCACCGCGCGCGGCTGGCGCTGCAGGTGAAGGGCGCGCCCTTCGACATCGTCAACATCAACCTGCTGGAGAAGCCCGAGTGGCTGACCGCGGTGCACCCGCAGGGCAAGGTGCCGGCGCTGGACGCGGGCGACGGGCGCATCGTCGTAGAGTCGCTAGACATCGCGGACTTCCTGGACGGAAAGTTCCCCGAGCCCCCGCTCTGGCCCGCCGACGAGGACACCAAGGCCCGCCACAAGAAGCTCATCGACGACTTCGGAAAGGTGGCGAAGATCTTCTACGAGGCGGTGTACGGCAAGGAGAAGCGGCCGCTGGGAGACTACCTGGAGGACATCGTGGCCGCCCTGCAGCCCTTTGAAGACGAGCTGGTTGCTGGCGGGAACCTCTTCTTCGGAGGAAGCTGCCCAGGTATGCTGGATTACATGATCTGGCCGTGGGCTGAGCGTGCCAAGGTCCCCAGCATCATCAACAAGCAGGAGATGAACTTCCCCAAGGATAAGTTCCCTAAACTCCTGGCGTGGCGTGAGGCCATGAAGGAAGACGGTGCGGTGAAGGCGCAGGCGGTGGACACGGAGACGCACGCGCGATTCAGCCGCGCATACGTCGACGGCACGCTAGACTACGACAACGTCTAG

>LmGSTO1

MSTKHLKKGDPQPAAAEGGKLRLYSMAFCPYAHRARLALQVKGAPFDIVNINLLEKPEWLTAVHPQGKVPALDAGDGRIVVESLDIADFLDGKFPEPPLWPADEDTKARHKKLIDDFGKVAKIFYEAVYGKEKRPLGDYLEDIVAALQPFEDELVAGGNLFFGGSCPGMLDYMIWPWAERAKVPSIINKQEMNFPKDKFPKLLAWREAMKEDGAVKAQAVDTETHARFSRAYVDGTLDYDNV

>LmGSTO2

ATGAGCCAAAAACATTTAAGCAGAGGTTCCCGACTGCCACCGTTCCCTAAAGGAAAATTGCGGCTGTATAGCATGAGATTTTGCCCTTATGCACAGAGGGTCCACTTGGTTCTAGATGCGAAGAGAATTCCGTACGAGGTTGTCAATGTTGACTTGACGGAAAAGCCCGATTGGTTGTATGAGAAGAGCCCTTTTGGTAAAGTGCCAGCAATTGAGCTTGAAAGTGGTGATACACTTTATGAAAGCCTAATAATTTGTGATTTTCTGGACGAGAAATACCCTAGTAGATCGCTGTATTCAAGAGACCCTCTCAAAAAGGCAAAGGACAAGATAATGATCGATCACTTCAATAAGGTTATTCAGTCAATGCTAAAGGTTTATTACCACACAGCAAATTCCAATCTGAATGAAGATCAGCTGGAAGAATTCTTTCAGGGGCTTGATTTATACGAGAGGGAATTAGTTGAAAGAGGGAAATCATTTTTTGGAGGTGACAGACCTGGAATGCTTGATTACATGATATGGCCTTGGTGTGAACGGTCAGATATGATGAAAGTGTTGGGTGGCGACCAATTTCTACTACCCAAAGACAGGTTTAAGAGATTGATGGAATGGAGGAACCAGATGAAAGAAGATGATGCTGTGAAGGAAAGTTACTTGGAACCACAAGTACATGCAAAGTATTTTCAGAGCCGTAAAGCTGGATATCCTGATTATGATATGCTTGTTGGCAATTGA

>LmGSTO2

MSQKHLSRGSRLPPFPKGKLRLYSMRFCPYAQRVHLVLDAKRIPYEVVNVDLTEKPDWLYEKSPFGKVPAIELESGDTLYESLIICDFLDEKYPSRSLYSRDPLKKAKDKIMIDHFNKVIQSMLKVYYHTANSNLNEDQLEEFFQGLDLYERELVERGKSFFGGDRPGMLDYMIWPWCERSDMMKVLGGDQFLLPKDRFKRLMEWRNQMKEDDAVKESYLEPQVHAKYFQSRKAGYPDYDMLVGN

>LmGSTo3

ATGGGTGTGAAGTTCCTGAAGAAGGGAGACCCGCGGCCGCCGTCGCTGGAGGCGGGCCGGCTGCGGCTGTACTCCAACCTGTACTGCCCGTACGCGCAGCGCGCGCGCCTCGTGCTGGCCGCCAAGGGCGTGCCCTTCGAGACCACCGAGATCAACCTGCGCAACAAGCCCGAGTGGTACAGCGCCGTACACCCCGACGCCAAGGTGCCCGCGCTCGAGGTGGCAGCGGGCGAGGTCGTCGTCGAGTCTCTCGACATAGCCGACTACCTGGACCGCAAGTTCCCGGAACCGAAGCTGTGGTCAGACGACCAGCAGAAGAACCTTCACCACAAGCAGCTGCTCGAAGCATTCGGCAAGGTCTTACCGAATTACGTGAAGCTGTTATCGGGCAATGCCTCACAGCCAGTGGAAGAGGTCGTGTCCGCTGTTGCAGCTATCATTAAGCCCCTGGAGGATGAACTCGCAAAGACGGGATCCACGTACTTCGGAGGAGCGAGGCCCGGCATGCTGGACTACATGGTGTGGCCCTGGGCGGAGCGCCTGGAGGGCCTGCGGGCGCTGCTCCCCGACGTCCAGCTGCCCCTCAACGACTTCCCGAGACTCATGGCGTGGGGAGCCGCCATGAAAGAGGACCCAGCGGTGAAAGCTTCAGCCATGAGCTTGGACACGCACGTCGGTTTCATGAAGATGTACAGGCAGGGCACTCTGGACTTCGACAAGCTGTAG

>LmGSTO3

MGVKFLKKGDPRPPSLEAGRLRLYSNLYCPYAQRARLVLAAKGVPFETTEINLRNKPEWYSAVHPDAKVPALEVAAGEVVVESLDIADYLDRKFPEPKLWSDDQQKNLHHKQLLEAFGKVLPNYVKLLSGNASQPVEEVVSAVAAIIKPLEDELAKTGSTYFGGARPGMLDYMVWPWAERLEGLRALLPDVQLPLNDFPRLMAWGAAMKEDPAVKASAMSLDTHVGFMKMYRQGTLDFDKL

>LmGSTz1

ATGTCTGTTATTGGAAAGCCAGTTTTATATTCGTACTGGAGGAGCTCTTGTTCCTGGAGAGTTCGAATAGCACTAAATCTGAAAGAAATACCATATGATATTAAACCAGTAAGTTTAATTAAAGCTGGTGGTGAACAACATTGCAATGAGTATCGTGAAATAAATCCAATGGAGCAAGTTCCAGCCCTCCATATTGATGGACATACTTTGGTTGAATCTCTGAATATCATGCACTACTTAGAGGAGACCAGACCTCAGCGACCTCTTATGCCACAGGATGTTCATAAAAGAGCAAAGGTCCGAGAAATATGTGAAGTAATTGCATCAGGTATACAGCCGCTACAGAATCTCATTGTTTTGATTTATGTTGGGGAGGAAAAGAAAAAGGAATGGGCACAACACTGGATTAATAGAGGATTCCGAGCTGTGGAGAAGCTACTGTCAGCGAGTGCTGGGAAGTATTGTGTTGGAGATGAAATTTCCCTGGCAGACTGCTGCCTCATACCACAGGTGTTTAATGCTCGCAGGTTTCATGTAGATCTTCGCCCCTTTCCAATAATTCTTCGTATTGATCGTGAGCTCGAAAATCATCCAGCTTTTAGAGCAGCACACCCATCCAACCAACCAGATTGCCCACCTGAAGCTACCAAGTAA

>LmGSTZ1

MSVIGKPVLYSYWRSSCSWRVRIALNLKEIPYDIKPVSLIKAGGEQHCNEYREINPMEQVPALHIDGHTLVESLNIMHYLEETRPQRPLMPQDVHKRAKVREICEVIASGIQPLQNLIVLIYVGEEKKKEWAQHWINRGFRAVEKLLSASAGKYCVGDEISLADCCLIPQVFNARRFHVDLRPFPIILRIDRELENHPAFRAAHPSNQPDCPPEATK

>LmGSTt1

ATGTCACTGAAAGTTTACTACGATCTTCTTTCACAACCATCAAGAGCAGTTGTCTTATTTTTGTTGGCAAATGACATCCCTTATGAGGCACGGGAAATTAATGTTCTCCACGGTGAGCAGTTTAGTGAAGAATTTGCTAAGCTTAATCCAATGAAGAAGGTTCCTGTAATCAAAGATGGAGATTTCACATTGACTGAAAGTGTAGCTATTTTAAGATACCTGTGCCGAGAGAGAGATGTTCCTGACCATTGGTACCCAGCAGATAGTAAAAAACAGGCAAGAGTTGATGAATATCTAGAGTGGCAACATACAAACACGAGAAGTAACTGTGCACTTTATTTTTTAAATAAGTTTATGCTTCCAGCAATCAAAGGAACACAGCCTAATCCAGAAACAGTGGCTAGGCGGGAACGAAAAATGGTAGCAACATTGAATGAAGTGGAAGAGATCTGGCTGAGAAACAAAACATATTTGGCTGGAGACAAAATCAGTATTGCTGATCTCCTAGGGGCATGTGAGATTGAGCAAACTAGAATGGCAGGCTACAATCCTTGTGATGGACGTCCAAAACTCGCTGCCTGGTTAGAACGAGTTCGTTGTGATACACTTCCGCATTATGATACAGTTCATGCATTAGTCAGAAAAGTGACTGAGAAGTATGGAGGTGTCCCACCAGCTTCCTTCTCAAAGCTGTGA

>LmGSTT1

MSLKVYYDLLSQPSRAVVLFLLANDIPYEAREINVLHGEQFSEEFAKLNPMKKVPVIKDGDFTLTESVAILRYLCRERDVPDHWYPADSKKQARVDEYLEWQHTNTRSNCALYFLNKFMLPAIKGTQPNPETVARRERKMVATLNEVEEIWLRNKTYLAGDKISIADLLGACEIEQTRMAGYNPCDGRPKLAAWLERVRCDTLPHYDTVHALVRKVTEKYGGVPPASFSKL

>LmGSTt2

ATGTCGCTAAAATTTTACTACGATATTTTCTCGCAACCATCTCGAGCAGTACTTTTACTTCTAAAATTAAACAAGATACCATTTGAATCCAAGAAGATAAATTTATTGAAAGGAGAACACTTCAGTGAAGAATATGCCAAGATAAATCCATTTCAGAAAGTTCCAGTTATTGTCCATGGTGACTTTGCACTTTTTGAAAGTGTGGCCATCTTACGTTATCTCTGTCGAGAATTTGAAGTTGCTGACCACTGGTATCCAAAGGACAGCAAGGCACAGGCAAAAGTTGATGAATATGTTGAATGGCAGCACATTGAAATTAGAGCCAACCTTGTGAACTACTTCCGTACAAAGTTTCTGCTTCCTGTGGTAACAGGAAGGCCAGCAGATGGGAAGAAAGTAATAGTATACGAGAAGAAAGTAACTGATATCCTTAATAAACTTGAGGAGACATGGCTTACTGAAAGACAGTATTTAGGTGGTGACAAAGTGAGCATTGCTGACCTGTTAGGAGCTTGTGAACTTGAACAGCTCAGAATGGCAGGATATAATCCACGAGACAATCGTCCCAAGGTTTCAGCTTGGTTAAATAAGTTGAAGGAAGAATTAAACCCACATTATGAAGAGGTTCATTCTCTTGTCAGTCATATAGCAGAAAAACATAAGGGTGTCCCTCCTAGCTCACCTGATAAGTTATGA

>LmGSTT2

MSLKFYYDIFSQPSRAVLLLLKLNKIPFESKKINLLKGEHFSEEYAKINPFQKVPVIVHGDFALFESVAILRYLCREFEVADHWYPKDSKAQAKVDEYVEWQHIEIRANLVNYFRTKFLLPVVTGRPADGKKVIVYEKKVTDILNKLEETWLTERQYLGGDKVSIADLLGACELEQLRMAGYNPRDNRPKVSAWLNKLKEELNPHYEEVHSLVSHIAEKHKGVPPSSPDKL

>LmGSTs1

ATGGCACCAAAATATAAGTTAACATACTTCGCTATTAAGGCTCTGGGTGAGCCAATACGCTTTCTTTTATCCTACGGGAAAATTGAATTTGAAGACGCAAGATTAGAATTTGAACAATGGCCATCTATGAAAGCATCAATGCCCTTTGGGCAGGTCCCAGTTCTTGAAATCGATGGGAAGAAGACTTGGCAATCTCTTGCAATATGCCGTTACTTAGGCAAACTGGTGGGTCTGGCTGGGGCTAATGACTGGGAGGATCTCCAGATTGATATGGCTATTGAGACTGTAACAGACCTGAGACTGAAAATAGCGAACTTTTGGTATGAGACTGATGAAGCCCAGAAGGCTAAAAAGAAAGAACCACTCTTGAAAGAAATTCTGCCATTTTTGTTGCCAAGACTTGATAACCTGGTGAAGGAAAATGGAGGTTATCTTGCAAATAGCAAGCTCTCATGGGCAGATCTGTACTTCATTGGAATACTAGACTATCTAAATTTCATGGTCGAATTTGACCTAACAAAAGATTATTCAAACCTTGCTGCACTCAAGAAGAAAGTACTTGAAATACCAGCTATTAAAGAGTGGGTTGACAAGAGACCAAACACTGAATGGTGA

>LmGSTS1

MAPKYKLTYFAIKALGEPIRFLLSYGKIEFEDARLEFEQWPSMKASMPFGQVPVLEIDGKKTWQSLAICRYLGKLVGLAGANDWEDLQIDMAIETVTDLRLKIANFWYETDEAQKAKKKEPLLKEILPFLLPRLDNLVKENGGYLANSKLSWADLYFIGILDYLNFMVEFDLTKDYSNLAALKKKVLEIPAIKEWVDKRPNTEW

>LmGSTs2

ATGGCGCCAAAATTCAAGTTACGTTACGGCGCTATAAAGGGTCTGGGTGAACCAGTACGTTTTCTACTCTCATACGGAAAGATTGAATTTGAAGATGAACGAATAGATCTACAGCAGTGGCCTTCTGTGAAAGAATCAATGCCATTTGGGCAGGTACCAGTTCTTGAAATTGACGGGAAGAAGACATGGCAGTCTCTTGCAATATGCCGTTACTTAGGCAACCAGATAGGCCTGGCTGGAGCTAATGATTGGGAGGATCTCCAGATTGATATGGCTGTTGAGACTGTAACAGATCTAAGACTGAAAATAGCGAGTTTTGCATATGAGAACGACCCATCCATAAAGGAAAAGAAGAAAGCACCAGTCGTTAATGAAAGTTTACCATTTTTGTTGGGAAGACTTGATAATCTTGTGAAGCAAAATGGGGGCCATCTTGCAAATGGAAAGCTTTCGTGGGCAGACCTCTATTTTGTTGCGCTGCTCGACTACCTCAACTTCTTGACTGGATTTGACCTAACAAAAGACTATCCTAACCTTTCTGCACTCAAACGTACTGTACTTGAAATACCAGCTATTAAAGAATGGGTTACCAAGAGACCAAAAACTGACATGTGA

>LmGSTS2

MAPKFKLRYGAIKGLGEPVRFLLSYGKIEFEDERIDLQQWPSVKESMPFGQVPVLEIDGKKTWQSLAICRYLGNQIGLAGANDWEDLQIDMAVETVTDLRLKIASFAYENDPSIKEKKKAPVVNESLPFLLGRLDNLVKQNGGHLANGKLSWADLYFVALLDYLNFLTGFDLTKDYPNLSALKRTVLEIPAIKEWVTKRPKTDM

>LmGSTs3

ATGGCACCAAAATACAAGTTAACGTACTTCCCTATAATGGGCCTGGCTGAGCCGATACGGTTTATACTCTCGTATGGCAAGATTGAATTTGAAGATAATCGGTTTGAGAGTGACAAATGGCCATCTATTAAAGAATCAATGCCCTTTGGGCAGGTACCAGTTCTTGAAATTGATGGAAAGAAGATATGCCAGACTATCGCTATATGTCGTTACTTAGCAAAGCAAGTTGGCCTGGCTGGGGATAACGATTGGGAAAATCTACAGATTGACATGGCTGCTGACACAGTAACTGACATAAGGACAAAAATTGTAAGTCCTAACTATGAGACTGACGAGGAAAAGAAGCAAAAGAAGAAAGCAGAAGTCATAAATGAAACCCTTCCATTTTTGTTGCCAAGACTTGATAATTTGGTGAAGCAAAATGGAGGATATCTTGCAAATGGCAAGCTCTCGTGGGCGGACCTCTACTTTGTAGCACTGATTGATCTTCTCAAGTTCATGATTGGATTTGACATAACAAAGGATTACTCAAACCTTTCCGCACTGAAGAATAAAGTACTTGAAATTCCAGCTATTAAGGAATGGGTTGCAAAGAGACCAAAAACTGAGAGATGA

>LmGSTS3

MAPKYKLTYFPIMGLAEPIRFILSYGKIEFEDNRFESDKWPSIKESMPFGQVPVLEIDGKKICQTIAICRYLAKQVGLAGDNDWENLQIDMAADTVTDIRTKIVSPNYETDEEKKQKKKAEVINETLPFLLPRLDNLVKQNGGYLANGKLSWADLYFVALIDLLKFMIGFDITKDYSNLSALKNKVLEIPAIKEWVAKRPKTER

>LmGSTs4

ATGGCACCAAAGTATAAGCTATCTTACTTCCCTATAATGGGTCTCGCTGAGCCAATACGTTTTCTGCTCTCATACGGCAAGATTGAATTTGAAGATGAACGGTTTGAGAGTGACCAATGGGCATCTGTGAAACCATCAACGCCCTTCGGGCAGGCTCCAGTCCTTGAGATTGACGGCAAGAAGACATGGCAGTCTATTGCTATTTGCCGTTACTTAGGAAAGCTAGTGGGTCTGGCTGGAGCTAATGATTGGGAAAATCTCCAGATCGATATGGCTGTCGACACAATATCAGACATAAGATCGAAAATAGCGAGTTATGGATATGAGGCCGATCCAGCCTTAAAGGAAAAGAAGAAAGCAGCAGTTGTAAATGAAACTCTACCATTTTTGCTGACAAGGCTTGATAAGCTTGTGAAGGATAATGGAGGTTATCTTGCAAATGGCAAGCTTTCATGGGCAGACCTCTACTTTGTAGGAATACTTGGCTACACCAACTACGTAAGTGGACTAGACCTAACGAAAGATTATTCAAATCTTTCTGCTCTGAAGAACAAAGTTCTTGAAATACCAGCTATCAAAGAGTGGGTTGCCAAGAGACCTAAAACTGAGAGATGA

>LmGSTS4

MAPKYKLSYFPIMGLAEPIRFLLSYGKIEFEDERFESDQWASVKPSTPFGQAPVLEIDGKKTWQSIAICRYLGKLVGLAGANDWENLQIDMAVDTISDIRSKIASYGYEADPALKEKKKAAVVNETLPFLLTRLDKLVKDNGGYLANGKLSWADLYFVGILGYTNYVSGLDLTKDYSNLSALKNKVLEIPAIKEWVAKRPKTER

>LmGSTs5

ATGGCACCAAAATACAAGTTAACTTACTTCCCTATAATGGGCCTGGCTGAGCCGATACGTTTTCTTCTCTCGTACGGCAAGATTGAATTTGAAGACGAACGGTGTGACCAGGACAAATGGCCATCTGTGAAAGAATCTACACCCTTGGGGAAGGTTCCGGTCCTTGAAATTGATGGGAAGACGACGTGGCAGTCTGTTGCAATATGCCGCTACTTAGCCAAGCAGCTGGGCCTGGCTGGAGCCAATGACTGGGAAGATCTGCAGATTGACATGGCAGTTGACACAATATCAGACTTAAGAATGAAAGTAGCAGCTTATGCCTATGAGACTGATGAAGCCTTAAAGGAAAAGAAGAAACAATCGCTTTTGAATGAAACACTACCATTCATGTTGCCAAGGCTTGATAAGATGGTGAAGGAAAATGGAGGATATTTTGCAAATGGAAAGCTTTCGTGGGCAGACCTCTATTTTGCAGCTATACCTATCAATTTCATGATGGGATTCGATATAACAAAAGACTATTCAAACCTTTCTGCACTCAAGAATACCGTATGTGAAATACCAGCTATTAAGGAGTGGATTAGCAAGAGACCAAAAACTGAGAGATGA

>LmGSTS5

MAPKYKLTYFPIMGLAEPIRFLLSYGKIEFEDERCDQDKWPSVKESTPLGKVPVLEIDGKTTWQSVAICRYLAKQLGLAGANDWEDLQIDMAVDTISDLRMKVAAYAYETDEALKEKKKQSLLNETLPFMLPRLDKMVKENGGYFANGKLSWADLYFAAIPINFMMGFDITKDYSNLSALKNTVCEIPAIKEWISKRPKTER

>LmGSTs6

ATGGGGACTGCTGAACCAATACGTTATCTTTTGTACCATGGTAAGATAAAGTTTGAGGATAATCGTGTTGATTTCCAGACTTGGCAGTCAATGAAAACATCTACACCATTTGGACAGATGCCCGTTCTTGAAATTGATGGAAAGAAAATGCACCAATCCATTGCCATTTGTCGCTATTTAGGCAAGAAGCTTGCACTGGCAGGTGAAAATGATTGGGAATCAGCTCAGATTGATATGGCTGTCGACACAGTACTAGACTTAAGAATAAAATTATCCGAGTTTTACTGGGAGTCTGACGAAACCGTAAAGCAGAGGAAAAAAGCAACCGTTTTAAATGAGACATTACCTTTCGTTCTTGAAAGACTTAATGGATTGGTGAAGGAAAATGGAGGTTATCTTGCACTAGGAAAGTTGACTTGGGGAGACTTCTTCTTTGCTGGAATCTCTGAGTATATGAACTGTGTTACTCAGTTTGATATTACAAAAGATTATCCAAATCTAGCTGCTCTTAAGAAAAAAATATGTGAACTGCCAGATGTAAAAGACTGGATCAGCAGGAGACCAGTATCACAACTTTGA

>LmGSTS6

MGTAEPIRYLLYHGKIKFEDNRVDFQTWQSMKTSTPFGQMPVLEIDGKKMHQSIAICRYLGKKLALAGENDWESAQIDMAVDTVLDLRIKLSEFYWESDETVKQRKKATVLNETLPFVLERLNGLVKENGGYLALGKLTWGDFFFAGISEYMNCVTQFDITKDYPNLAALKKKICELPDVKDWISRRPVSQL

>LmGSTs7

ATGTCACCAACATGTAAATTAACTTACTTTGATGCTATGGGGATTGGTGAACCAATACGTTTCCTTTTGTGCTATGGAAAGATACAGTTTGAAGATATTCGTTTTGATTTTGAAAAATGGCCATCAATGAAACCATCTACACCATATGGAAAGGTACCTCTTCTTGAAATTGATGGAAAGAAGATGCATCAGTCCGCTGCCATATGCCGTTATTTAGGCAAGAAGCTGGGACTGGCAGGTGCAAATGATTGGGAATCTGCTCAGATTGATATGGCTGTTGATACAATAACGGACTTAAGATTGAAGGTAACGGAGTATTACTGGGAACCCGAGGAAACTACAAAACAGAAGAAGAAAGAAACTCTGTTAAATGAAACACTTCCCTTCTATATGGAAAAGCTTGATGCCCTGGTGAAAGAAAATGGAGGTTATCTTGTAGCAGGGAAGTTGTCTTGGGGAGACTTTTTCTTTGCTGGAGTCTCTGATTACATGGATTGTGTAGTTCAATTTGATATTACTAAAGATCATCCAAATCTAGCTGCTCTCAAGAAGAAGATACGTGAACTGCCAGCCATAAAAGAATGGATCAGCAAGAGACCAAAGACAACCCTTTGA

>LmGSTS7

MSPTCKLTYFDAMGIGEPIRFLLCYGKIQFEDIRFDFEKWPSMKPSTPYGKVPLLEIDGKKMHQSAAICRYLGKKLGLAGANDWESAQIDMAVDTITDLRLKVTEYYWEPEETTKQKKKETLLNETLPFYMEKLDALVKENGGYLVAGKLSWGDFFFAGVSDYMDCVVQFDITKDHPNLAALKKKIRELPAIKEWISKRPKTTL

>LmGSTs8 ATGGCAGCAAAATATAAGTTAACTTACTTTCCTATAATGGCTCTGGGTGAGCCAATACGTTTTCTTCTATCATATGGAAAGATTGAATTTGAAGATGAACGATGTGAGAGGGAGAAGTGGCCATCTGTAAAAGAATCAATGCCCTTTGGTCAGATGCCAGTTCTTGAAATTGATGGGAAGAAGACATGGCAGTCTCTTGCAATATGCCGTTACTTAGGCAAGCAGATGGGCCTGGCTGGGGCTAATGACTGGGAGGATCTCCAGATTGATATGGCTATTGAGACAGTAACAGACCTGAGACTGAAAATTGCAAATTATTGGTATGAGACTGATGAAGCCCAGAAAGAGAAAAAGAAGGAACCCCTCTTCAATGAAACTCTGCCATTTTTGTTGCCAAGACTCGATAACCTGGTGAAGGAAAATGGAGGTTATCTTGCAAATGGCAAGCTCTCGTGGGCAGACCTGTATTTCATAGCAGTGCTAGATTATCTAAACACCATGGTTGGATTTGACCTAACAAAAGATTATCCAAACCTTGCAGAACTCAAGAATAAAGTCCTTGAAATACCAGCTATTAAAGAGTGGGTTGACAAGAGACCAAAAACTGATTTGTGA

>LmGSTS8

MAAKYKLTYFPIMALGEPIRFLLSYGKIEFEDERCEREKWPSVKESMPFGQMPVLEIDGKKTWQSLAICRYLGKQMGLAGANDWEDLQIDMAIETVTDLRLKIANYWYETDEAQKEKKKEPLFNETLPFLLPRLDNLVKENGGYLANGKLSWADLYFIAVLDYLNTMVGFDLTKDYPNLAELKNKVLEIPAIKEWVDKRPKTDL

>LmGSTs9

ATGGCGCCGAAGTATAAGCTGACTTATTTCCCTGTCAAGGCTTTGGGTGAACCTATCAGATTTCTGTTGTCATATGGTAATATGGAGTTTGAAGATGATCGTTTCGAAAGGGAGAAGTGGCCGTCAATTAAACCATCGATGCCATTTGGACAAGTTCCTGTCCTAGAATTTGATGGAAAGAAGACACATCAGTCAATTGCTATTTGTCGGTACTTGGGAAAGCAGTTGAAATTAGCTGGTGATAATGATTGGGAAGCTCTCCAGATTGATATGGCTGCTGACTGCTTAACAGATTTAAGAATGAAGCTGGGAAGTTTTTTCTATGAATCAGACGAAGCTGTGAAGGAGAAAAAGAAAGAACCAATTATAAAGGAGTTTCTGCCGTTCTTTTTGCCTAGACTGGATAACCTGGTAAAGGAGAATGGAGGCTATCTAGCAAATGGAAAGCTCTCGTGGGCAGATTTCTATTTTGCTGGAATATTGGACTATGTTAATCATATGGCTGGGTTTGACATTACTAAGGATTATGCTAACCTTGCTGCACTGAAGAACAAGGTCCTTGAACTACCAGCTATTAAGGAATGGATTGCCAAAAGGCCAGTTTCAGAAGTGTGA

>LmGSTS9

MAPKYKLTYFPVKALGEPIRFLLSYGNMEFEDDRFEREKWPSIKPSMPFGQVPVLEFDGKKTHQSIAICRYLGKQLKLAGDNDWEALQIDMAADCLTDLRMKLGSFFYESDEAVKEKKKEPIIKEFLPFFLPRLDNLVKENGGYLANGKLSWADFYFAGILDYVNHMAGFDITKDYANLAALKNKVLELPAIKEWIAKRPVSEV

>LmGSTs10

ATGGCACCAAAATATAAACTACTTTATTTTCCATTCAGAGTCATTGCTGAGCCAATTCGATTCATTTTAGCATATGTTGGAGCTGATTATGAAGATGTCAGAATTGAATTTAGTGATTGGCCACCTCTTAAACCAGATACACCATATGGAACAATGCCTGTCCTTGAAGTTGATGGAAAGAAACTTGGTCAGTCCATCCCCATATGTCGTTACCTAGCAAAGCAATATGGATTATTGGCTGAAAATGACTGGGATAATGCTCAGATTGATGCAGCAGTTGATGCCATCAATGACTTAAGAATGTCTATAAAGAATTTCTACTTTGAGGAAAATGGATCTACAAAAAATAATATGAAGGAAAAACTAATGAAGGAAGCAGTACCATTTTATTACAAAAAATTGGAGGAAATGGTGAAAAATAATGGTGGCTATCTTGCTGGTGGGAAGCTCACTTGGGGTGACCTGTACCTCACAACTATGCTGGAACTTTTTGATCATGATCTTGAGTGTGAAATAACCAAAGATTATCCACATCTTGCAACATTACGCAACAAAGTTGTGAATATACCTTCTATTAAAACATGGATTGAGAAACGACCAAAGTCAGAGAGATGA

>LmGSTS10

MAPKYKLLYFPFRVIAEPIRFILAYVGADYEDVRIEFSDWPPLKPDTPYGTMPVLEVDGKKLGQSIPICRYLAKQYGLLAENDWDNAQIDAAVDAINDLRMSIKNFYFEENGSTKNNMKEKLMKEAVPFYYKKLEEMVKNNGGYLAGGKLTWGDLYLTTMLELFDHDLECEITKDYPHLATLRNKVVNIPSIKTWIEKRPKSER

>LmGSTd1

ATGCCATCAGTGGACTTGTACTACGTTCCGGGCAGTGCTCCTTGCAGAGCAGTCCAGATGGTGGCCAAGGCTGTGGGTGTTGACCTCAACTTGAAACTTGTGAACCTCATGGAAGGTGAACAGATGAAGCCAGAGTATCTGAAGATGAATCCTCAGCACACAGTTCCTACAATTGATGACAATGGCCTTTACCTTTGGGAGAGCCGTGCCATCATTGGTTACCTGGTAGAGCAGTATGCCAAAGACGACTCACTTTACCCTAAGGAGGCAAAGAAGAGAGCATTGGTGAACCAAAGAATGTATTTCGACATTGGAACACTATATGCCAGATTTGCTGATTATTATTATCCTGTAATGTTTGGTGGAGCAAGTTATGATCCAGAAAAATTAAAGAAACTGGAAGAAGCATATGAATTTCTGAACAAATTCTTAGAAGGTAGTGACTGGGTTGCTGGCAACTCCATCACAATTGCAGATTACACAATAATGGCATCAGTCAGTACTGCAGAGATTATTGGCTTTGATATCAAAAAGTTCCCAAAAGTTGCAGCTTGGTTTGAGAAAGCAAAGAAAGAAATTCCATCATATGAAGAAACTAATCACGCAGGAGCATTAGAATTTAAGAAACTGTTTGATTCAATGACTGCCAAGAAGTAA

>LmGSTD1

MPSVDLYYVPGSAPCRAVQMVAKAVGVDLNLKLVNLMEGEQMKPEYLKMNPQHTVPTIDDNGLYLWESRAIIGYLVEQYAKDDSLYPKEAKKRALVNQRMYFDIGTLYARFADYYYPVMFGGASYDPEKLKKLEEAYEFLNKFLEGSDWVAGNSITIADYTIMASVSTAEIIGFDIKKFPKVAAWFEKAKKEIPSYEETNHAGALEFKKLFDSMTAKK

>LmGSTd2

ATGGCACCCCCAACTCTTTACGACAACCCTATCAGTCCTCCGTGTCGTCTGGTACGGCTGGTTGCTGGCGTCATTGGAGTTGACCTGAAAGTGGTGAACGTCAAGGATGCTGGCATTGACATGAAGTCGCCCGAAATGTTGAAGAAAAATCCTCAACATACTGTTCCCACGCTGGAAGACAACGGCCTGTGTCTTGCTGAAAGCCGTGCCATTTCCATGTACCTCATTTCGAAGTACGCGAAGGACGACTCTCTCTACCCTAAGGATGTAAACAAACGTGTCCTCGTCGACCAGAGGCTATTTTATGACCAAGATTTGTACAACAGAATTTTGAGTGTTTTCTTGCCAAAATTCTTCGGCAAGCAGACGGATCCCAGCAGCATCGAGAAAGTGAACGAAGGGCTGGAAACTCTGAACCGAATGCTCGATGGCAAGCAGTGGCTGGCCGGGGACAATATTACACTGGCAGACTACGCCATCGCTAATACTCTATCAGCGCTCGAGTTCGCTCCAGAATTTGGGATTGATCCTACGAAGCAGCCCAACATCAAGCAGTGGCTTCCACGAGTTGAAAGCTCCAGTCCAAAATATGGAGAGAGTCTGAAGGAATTCCGCGAGGCTTTGAAGAAGATGGCGCAGAAGTAA

>LmGSTD2

MAPPTLYDNPISPPCRLVRLVAGVIGVDLKVVNVKDAGIDMKSPEMLKKNPQHTVPTLEDNGLCLAESRAISMYLISKYAKDDSLYPKDVNKRVLVDQRLFYDQDLYNRILSVFLPKFFGKQTDPSSIEKVNEGLETLNRMLDGKQWLAGDNITLADYAIANTLSALEFAPEFGIDPTKQPNIKQWLPRVESSSPKYGESLKEFREALKKMAQK

>LmGSTd3

ATGTCACCAATAATTCTGTACAACCACGACGGTAGTCCTCCATGTTGTCTTGTTCGTCTGGTAGCTGGTGTGCTTGGACTTGAACTCAAGAAAGTGGATATAAGTGACATAGAAAATGGAATGAGAAATCCTGAAATGCTAAAGAAAAACCCACAGCACACTATTCCTACAATAGAAGACGATGGTTTGGTCATCACTGAAAGCCGGGCGATAGCAATGTACCTCGTGTCCAGATACGCCAAGGACGATTCTCTGTATCCAAAGGAACTGCAGAGGCGTGTTCTCGTCGACCAGAGACTGTTCTTTGACCAAGATTTGTACAACAGAATTATGGCTGTTTGTAACCCATTATTCTATGGGAAAACTGTGGCTGAAAGCGACGCCGACAGGATGAAAGACGGCCTTGATACCTTAAGCAGAATGTTGGATGGCAAACAGTGGCTAGCAGGGGACCACGTAACGCTTGCCGATTATGCAGTCGCTGTATCTCTGGCCACACTAGAGTACAATCCAGAATTTGCCATTGACGCTGCCAAACACCCTAATATCACCCAATGGATATCTCGTCTTGAAGAATCTTCTAACACATACAGAGAGCACATTGAACTGTTTTTTGAGACCGTAAGAAAAGCAACCGAAGAAGAAAAGAAGAATGCTCAACAGAAATAA

>LmGSTD3

MSPIILYNHDGSPPCCLVRLVAGVLGLELKKVDISDIENGMRNPEMLKKNPQHTIPTIEDDGLVITESRAIAMYLVSRYAKDDSLYPKELQRRVLVDQRLFFDQDLYNRIMAVCNPLFYGKTVAESDADRMKDGLDTLSRMLDGKQWLAGDHVTLADYAVAVSLATLEYNPEFAIDAAKHPNITQWISRLEESSNTYREHIELFFETVRKATEEEKKNAQQK

>LmGSTd4

ATGCCGTCCATCATCCTCTATGGAAACGAGCTGAGCCCACCGTCACGAGCAGCCAAGATGATCGCTGAAAAACTGGGAGTCGACGTCGACTTCAAGAGGACCTACCCCATCAAAGGGGAGTGCAAGAAACCTGAATACCTAAAGATTAACCCTATGCACACTATTCCAACTATTATAGACGGCCCATTCACATTGTCTGACAGTCATGCAATTGTGGCGTATTTAGTAGACAGATTTGGAAAGAATGACAGCCTCTATCCAAAGGACATACAGAAACGCTCAAAAGTCAATGAGAGACTGTGTTTCGACATATCGTTGTTCACTAAAGTTTTGAAATTTGTGGTTGGACCTCTACTCAGGACACATGAGCCGACTGAAGAATTGAGGAACGACTGTATAGACGGCCTGGAGACGGTGGAGCGCTTCCTCAGTGCCAGCAAGTTCATCGCCGGGGACGACTTGACCGTCGCCGACTACTGCTACTACTGCACCATCACGTTTGTTGACATTCCACAGAAAGGTGTAATAGACCTAAAAAAGTACAAAAATATCCAAAGGTGGATGGACCTAATCCACAAGACATGTCCCTTGTTTACGAAATATGACCAGATCGCTGAAAAGGCGTTTCAGATATACTTGGAAGATGGGCCGTATTAG

>LmGSTD4

MPSIILYGNELSPPSRAAKMIAEKLGVDVDFKRTYPIKGECKKPEYLKINPMHTIPTIIDGPFTLSDSHAIVAYLVDRFGKNDSLYPKDIQKRSKVNERLCFDISLFTKVLKFVVGPLLRTHEPTEELRNDCIDGLETVERFLSASKFIAGDDLTVADYCYYCTITFVDIPQKGVIDLKKYKNIQRWMDLIHKTCPLFTKYDQIAEKAFQIYLEDGPY

>LmGSTd5

ATGCCGAAGCTGCGCCTGTACCACGCCGAGGCGAGTCCCCCGTGCCGGACCGTGCGCATGGTGGCGCGCACGCTGGGGCTGGAGCTGCAGCTGGTCGATGTGGACTTGTTGCAGAACGAGCACCTCAAGCCCCAGTTCCTACAGATGAATCCACAACATCAGGTACCACTACTCGATGACAATGGATTTTACTTACCAGAGAGTCGAGCGATTATAGCGTACCTGGTGGATAATTACGCAAAGGACGACTCATTATACCCGAGAGATCCTAAGAAGAGGGCAACTGTCAACAGGATGTTGTTCTTCGATGCTGGAACACTCTGGATGAATTACCACATTTATCAGATGCCGATTTTCTACGGTCAGCCAGGGGATCCAGAGAAGCAGAAGAAAATGCTGGAAGCTTTCGCCTTGTTCGACAAGCTGCTGGAGGGAAAGGAGTGGGCCGCGGGCAGCCACGTCACCATCGCAGACTACGCCCTCGCCGTCACCGCCACCAGTGCCCAGTGGTCAGGAGTGGACCTAAAGGTGTACGATAACGTTACTAGATGGCTGGAACGCACGAAAGAGGGTATTACCTGCTTCGACGAGATCAACAATTCATCCTTTGCTGCTCTAAAGGAACTACTACAGAAGTTGAAAGAGAAGCTCGCAGAGATGTCAGTCGACAAATAA

>LmGSTD5

MPKLRLYHAEASPPCRTVRMVARTLGLELQLVDVDLLQNEHLKPQFLQMNPQHQVPLLDDNGFYLPESRAIIAYLVDNYAKDDSLYPRDPKKRATVNRMLFFDAGTLWMNYHIYQMPIFYGQPGDPEKQKKMLEAFALFDKLLEGKEWAAGSHVTIADYALAVTATSAQWSGVDLKVYDNVTRWLERTKEGITCFDEINNSSFAALKELLQKLKEKLAEMSVDK

>LmGSTd6

ATGGCTCCCCCGACTCTTTTTAACGTTACCCTGAGTCCTCCATGCCGCCTGGTCCGTTTAGTGGCCGGTATCATTGGAGTTGACCTCAAAGTAGTGGATGTGAAGGACATTAGTAAAGAGATGAAGACACCTGAAATGTTGAAGAAAAATCCTCAACATACCGTTCCCACACTGGAAGACAATGGCGTGTATCTAGCTGAAAGCCGTGCCATTGCCATGTACCTCATTTCGAAGTACGCGAAGGACGATTCGCTCTACCCGAAGGATGTAAACAAACGTGTCCTCGTCGACCAGAGGCTATTTTATGACCAAGATTTGTACAACAAGATTTTGAATGTTTTCTTGCCAAAATTCTTCGGCAAGCAGACGGATCCCAGCAGCATCGAGAAAGTGAACGAAGGGCTGGAAACTCTGAACCGAATGCTCGATGGTAAGCAGTGGCTGGCCGGGGACAACGTAACATTGGCCGACTACGCCGTCGCTATCTCCCTGTCATCGCTCGATTTCGTTCCAGAAAGTGGCATTGACCCTAAGAAGCAGCCTAACATCAACCAATGGCTTCCACGTGTCGAAAATTCTCACCCAAAATACGGAGAGCACCTCAAGGAATTCCATGAAGCATTAAAGAAATTGACGCAGAAGTAG

>LmGSTD6

MAPPTLFNVTLSPPCRLVRLVAGIIGVDLKVVDVKDISKEMKTPEMLKKNPQHTVPTLEDNGVYLAESRAIAMYLISKYAKDDSLYPKDVNKRVLVDQRLFYDQDLYNKILNVFLPKFFGKQTDPSSIEKVNEGLETLNRMLDGKQWLAGDNVTLADYAVAISLSSLDFVPESGIDPKKQPNINQWLPRVENSHPKYGEHLKEFHEALKKLTQK

>LmGSTd7

ATGGCGGGCCTGAAGCTGTACTCGGTCAGTGACAGTCCTCCGTCGACGGCCGTCAAGATGGCGCTCGAGGCCCTGGCACTCGAGTACACCAACGTGGAGGTCGACTTCGCTGCCGGCGAACACCTCAGCGACGACTTCAGCAAGAAAAATCCGCAACGAGAAATTCCCTGCCTGGACGACAATGGTTTTTTTCTGAGTGAAAGTGTCGCCATCCTGCAGTACCTGGCAGACAAGTACGGCCCCGGACACAGCCTGTACCCGCGGGACCCCCAGCAGAGAGCGCTGGTCAACCACAGGCTCGCCTTCAACATTTCCACCTACTACGCACGAATAGCGGAGTATGCGGTAGCACCAATATTCTTCGATTACAAGAGAACTCCAGAGGGCCTGAACAAATTGAAGATAGCTCTGAACGTCTTAAACACCATTTTAGAAAGACAAGGAACCAAATTTGCAGCAGGAGAGCACATGACGCTGGCGGACCTGTCGCTGGTAGCAGCCACCATGTGCCTGGAGGCGGTGGAGTTTGACCTGACCCCTTGGGCGCGCGTGCAGCGCTGGTACGCAGACTTCAAGCAGGCGGCGCCGCGCCTCTGGGCGGTCGCCGAGCCCGCCATGCTGGAGCTCAGGGCCTTCAGCAACAGCCCGCCCGACCTGTCGGCGCTCCGGCACCCCTACCACCCCGTCAGGGCCAGCAAGTAG

>LmGSTD7

MAGLKLYSVSDSPPSTAVKMALEALALEYTNVEVDFAAGEHLSDDFSKKNPQREIPCLDDNGFFLSESVAILQYLADKYGPGHSLYPRDPQQRALVNHRLAFNISTYYARIAEYAVAPIFFDYKRTPEGLNKLKIALNVLNTILERQGTKFAAGEHMTLADLSLVAATMCLEAVEFDLTPWARVQRWYADFKQAAPRLWAVAEPAMLELRAFSNSPPDLSALRHPYHPVRASK

>LmGSTe1

ATGCCTATTGTTTTGTATCACTTTCCTCCCAGTCCACCATCGAGAGCAGTTTTGACTACAGCAAAAGCCATTGGAGTGGATGTCACCATCAAAATCATCGATCTATTTAAGAATGAACACTTGACAGAAGACTATCTTAAGATAAATCCAGAGCACATGGTTCCAACCATCGACGACAACGGTCTTATTCTTCACGACAGCCACGCGATCGCCACGTACCTGGTGTCTCGCTATGGGAAGGATGACTCCCTCTACCCGAAGGATGTGGAGCAGAGGGCCCTGGTAGACCAAAGGCTGTACTTCGACGCGACTATACTCTTCTCCCGTCTAAGGGCCACAACTTTCCCGATTTTCTTCCAAGGGAAAAGAAATGTGGACGCGGCCGCGAGAGATGCGATATACGAGGCCCTGGGCATCCTGGAGAAGTACCTGGAGCCCACTGGATGGGCGGCTGGAGAACGCGCCACCGTGGCCGACCTCTCGTGCTCGGTGACAGTTGGCAGTCTGCGGGCTATAGGAGTGGACCTAAGTGCTTATCCGAAGATTAGGGACTGGTTGAAACGATGCAAAAGCACATTTAGCGGCTACGAGGAGGCTAATCAAGAAGGCGAGAAACTTATTGGGGATGGCATCAAGAACCTTGTGGGATGCAAGGCTTATTCAAGATAG

>LmGSTE1

MPIVLYHFPPSPPSRAVLTTAKAIGVDVTIKIIDLFKNEHLTEDYLKINPEHMVPTIDDNGLILHDSHAIATYLVSRYGKDDSLYPKDVEQRALVDQRLYFDATILFSRLRATTFPIFFQGKRNVDAAARDAIYEALGILEKYLEPTGWAAGERATVADLSCSVTVGSLRAIGVDLSAYPKIRDWLKRCKSTFSGYEEANQEGEKLIGDGIKNLVGCKAYSR

>LmGSTe2

ATGCCGGTCACCCTCTACCATTTCCCGGCCAGTCCGCCTTCCAGAATAGCGCTGGCGGCGGCTAAGGTGGTCGGCGTCGATGTGGACGTCAAAATCGTCAATCTATTCGCAGGGGAGCATCTCAAGGAGGAATTTGTCAAGTTTCCGGTGCTGTTCCTGGGAAAGACGAGTGTGGAGGACTCCGTGAAGGCGTCGGCGTACGAGGCCATCGGCTTCCTGGAGAAGTTCCTGGAGCCTTCTGGCTGGGTGGCCGGCGACCACCTCACCATAGCCGACGTGGCCTGCGCCGTCACCGCCACCAGCATGCAGGCGATTGGCTTGGATTTCAGTGGCTATCCGAAGACAAAAGACTGGATTGAGCGTTGCAAGAAGATCCCGGGATTCCAGGAGGCCAACGAAGAAGGCGCAGAGATTTTCGGAGAGAGAGTCCGAAGCCGCTTACCGCCGAATCACCTGGCTCCGTAG

>LmGSTE2

MPVTLYHFPASPPSRIALAAAKVVGVDVDVKIVDLFAKEQLKEEFVKFPVLFLGKTSVEDSVKASAYEAIGFLEKFLEASGWVAGDHLTIADVACAVTATSMQAIGLDFSGYPKTKDWIERCKKIPGFQEANEEGAEIFGERVRSRLPPNHLAP

>LmGSTe3

ATGCCAGTCACCCTCTACCATTTCCCGGCCAGTCCGCCTTCCAGAATAGCGCTGGCAGCAGCTAAGGTTGTCGGCGTCGATGTAGACGTCAAAATCGTCGATCTATTCGCAAAAGAACAACTCAAAGAAGAATTTGTCAAGTTCCCGGTGCTGTTTCTGGGAAAGACGAGTGTGGAGGATTCCGTGAAGGCATCGGCGTACGAGGCCATCGGCTTCCTGGAGAAGTTCCTGGAGGCTTCGGGCTGGGTGGCTGGCGACCACCTCACCATAGCCGACGTGGCCTGCGCCGTCACCGCCACCAGCATGCAGGCGATTGGCTTGGATTTCAGTGGCTATCCGAAGACAAAAGACTGGATTGAGCGTTGCAAGAAGATCCCGGGATTCCAGGAGGCCAACGAAGAAGGCGCAGAGATTTTCGGAGAGAGAGTCCGAAGCCGCTTACCGCCGAATCACCTGGCTCCGTAG

>LmGSTE3

MPVTLYHFPASPPSRIALAAAKVVGVDVDVKIVDLFAKEQLKEEFVKFPVLFLGKTSVEDSVKASAYEAIGFLEKFLEASGWVAGDHLTIADVACAVTATSMQAIGLDFSGYPKTKDWIERCKKIPGFQEANEEGAEIFGERVRSRLPPNHLAP

>LmGSTe4

ATGCCAGTCACCCTCTACCATTTTCCAGCCAGTCCGCCTTCCAGAATAGCGCTGGCGGCAGCTAAGGTGGTCGGCGTCGATGTGGACGTCAAAATCGTCAATCTATTCGCAGGGGAGCATCTCAAAGAGGAATTTGTAAAGATAAATCCACAGCACACGGTACCGACTATTGATGACAACGGCTTCATCTTGTGGGACAGCCACGCCATCGCCACGTACTTCGTTTCGCAGTACGGGAAGGATGACTCCCTGTATCCAAAGGAGGCAAAGCAGAAGGCGTTGGTGGACCAGAGATTGTTCTTCGAGGCGGGGACCATGTCCTCCCGCATGAGGGACATTGTGTATCCGGTGCTGTTTCTGGGAAAGACGAGCGTGGAGGATTCCGTGAAGGCGTCGGCGTACGAGGCCATCGGCTTCCTGGAGAAGTTCCTGGAGCCTTCTGGCTGGGTGGCCGGCGACCACCTCACCATAGCAGACGTGGCGTGCGCCGTCACCGCCACCAGCATGCAGGCGATGGGCTTGGACTTCAGTGGCTACCCGAATACACAAGACTGGATGGAGCGTTGCAAGAAGATCCCGGGATTCCAGGAGGCCAACGAAGAAGGCGTGACGCTCTTCGGGGAGAAAGTCAGAAGCCAACTACCGCCAAACCATCTGGCTCCGTAG

>LmGSTE4

MPVTLYHFPASPPSRIALAAAKVVGVDVDVKIVNLFAGEHLKEEFVKINPQHTVPTIDDNGFILWDSHAIATYFVSQYGKDDSLYPKEAKQKALVDQRLFFEAGTMSSRMRDIVYPVLFLGKTSVEDSVKASAYEAIGFLEKFLEPSGWVAGDHLTIADVACAVTATSMQAMGLDFSGYPNTQDWMERCKKIPGFQEANEEGVTLFGEKVRSQLPPNHLAP

>LmGSTe5

ATGCCAGTCACCCTCTACCATTTCCCGGCCAGTCCGCCTTCCAGAATAGCGCTGGCAGCAGCTAAGGTTGTCGGCGTCGATGTAGACGTCAAAATCGTCGATCTATTCGCAAAAGAACAACTCAAAGAAGAATTTGTCAAGATAAATCCACAGCACACGATACCGACTATTGACGACAACGGCTTCATCTTGTGGGACAGCCACGCAATCGCGACGTACCTCGTGTCGCAGTACGCGAAGGATGACTCCCTGTATCCAAAGGACACAAAGAAGAAGGCGGTGGTCGATCAGAGACTGTACTTCGAGATAGGGACGCTGTACCCTCGCATGAGGGCAATTGCCTTCCCGGTGCTGTTTCTGGGAAAGACGAGTGTGGAGGATTCCGTGAAGGCATCGGCGTACGAGGCCATCGGCTTCCTGGAGAAGTTCCTGGAGGCTTCGGGCTGGGTGGCTGGCGACCACCTCACCATAGCCGACGTGGCCTGCGCCGTCACCGCCACCAGCATGCAGGCGATTGGCTTGGATTTCAGTGGCTATCCGAAGACAAAAGACTGGATTGAGCGTTGCAAGAAGATCCCGGGATTCCAGGAGGCCAACGAAGAAGGCGCAGAGATTTTCGGAGAGAGAGTCCGAAGCCGCTTACCGCCGAATCACCTGGCTCCGTAG

>LmGSTE5

MPVTLYHFPASPPSRIALAAAKVVGVDVDVKIVDLFAKEQLKEEFVKINPQHTIPTIDDNGFILWDSHAIATYLVSQYAKDDSLYPKDTKKKAVVDQRLYFEIGTLYPRMRAIAFPVLFLGKTSVEDSVKASAYEAIGFLEKFLEASGWVAGDHLTIADVACAVTATSMQAIGLDFSGYPKTKDWIERCKKIPGFQEANEEGAEIFGERVRSRLPPNHLAP

>MAPEG1

ATGGCTGCTGCGGAGTTATTACTAACCACAAGTAATCCTGTGTTTAAGGCATACCTCTTACATGTTGCAGTACTTGGACTAAAGATGCTTTTGATGTCTCCGCTCACAGCACGACAACGATTTAAAAATAAGATTTTTGCAAGTCCTGAAGACACCGTATCAATGAAAGGTGCCAAAGTTAAATATGATCATCCTGATATTGAACGTGTTAGGAGGGGTCATCTGAACGATTTGGAGAATATCACAGTGTTCTTTATTGTGGCACTAGCATATCTACTAACAAATCCATCTCCAGGTCTTGCCATCAATCTGTTCAGAGCATATACTATAGCCCGAATTGGACACACAATTGTGTACTGTGTCATACCATTACCACAACCATCACGGTTCTTATTTTGGATTGTTGGATGGGGTATTACAGTGTTCATGACAGGCAGTGTGATACTTAAATTCATGTGA

>MAPEG1

MAAAELLLTTSNPVFKAYLLHVAVLGLKMLLMSPLTARQRFKNKIFASPEDTVSMKGAKVKYDHPDIERVRRGHLNDLENITVFFIVALAYLLTNPSPGLAINLFRAYTIARIGHTIVYCVIPLPQPSRFLFWIVGWGITVFMTGSVILKFM

>MAPEG2

ATGTCGCTCGAGTCAGAGCTGCGCGGCGCCGCCACGGCCGCCACCACGGCGCTGGTGGCCGCCAACCCCGTGCTGCGCGACTACGCCTACTACAGCGCGCTGCTCGGCCTCAAGGTGCTGGCCATGGGGCCGCTCACCGCCAGGCAGCGCTTCGCCAAGAAGGTGTTCCTGAACCCTGAAGATGCAAAATTTTTTAGTGGCTCTGAAAAGCTGGATGATCCAGATGTGGAAAGAGTTCGCAGGGCCCATCGCAATGACCTAGAAAATATCCCCGTTTTTATGTTGATTGGTGGTTTGTATACACTGACTAATCCAGATCCTAAATTGGCACTGAATCTGTACCGTGGATACTTTGCTTTCCGACTAGCACATACTGTTGTGTATGCAGTGTACCCAGTTCCACAACCAGCAAGAGTACTTTGTCATTTGGGTGCATTAACAATTTGTGTCTATATGGGAGTCAAGGTGGTAAAATTTTTCTTTTAA

>MAPEG2

MSLESELRGAATAATTALVAANPVLRDYAYYSALLGLKVLAMGPLTARQRFAKKVFLNPEDAKFFSGSEKLDDPDVERVRRAHRNDLENIPVFMLIGGLYTLTNPDPKLALNLYRGYFAFRLAHTVVYAVYPVPQPARVLCHLGALTICVYMGVKVVKFFF

>MAPEG3

ATGCTCCTCATGTCTCCGCTCACGGCACGGCAGCGTTTTAAAAATAAGAGTTTCTCAAATCCTGAAGATACTATGTTTGTGAAAGGTAGCAAGGTGAGGTATGATCATCCGGATGTTGAACGAGTTAGGAGGGCTCATTTAAATGACTTGGAGAATATCTCAGTGTTCTTTATTGTGGCACTAGCATATGTACTAACAAATCCACCTCCAGTTCTTGCGATCAACCTCTTCCGAGCATTTACTGTAGCCCGAATTGGCCACACAATTGTATACTGCATCTTACCAATTCCACAACCTGCTCGGTTCTTATTTTGCTTTGTTGGTTGGCTTATCACAATTTTCATGGCAGGTAGTGTGATACTTTATTCCCTTTGA

>MAPEG3

MLLMSPLTARQRFKNKSFSNPEDTMFVKGSKVRYDHPDVERVRRAHLNDLENISVFFIVALAYVLTNPPPVLAINLFRAFTVARIGHTIVYCILPIPQPARFLFCFVGWLITIFMAGSVILYSL

>MAPEG4

ATGATTACTCTCCAGGTTCCATCACAGTATGGCTACTGTGTTCTCGTTGCAGTGGGCTCAATCTTTGTTCTTATGTGGAAGGGTATCAAGGTTGGGGTTGCGCGAAAGAAGTTTAACATACCATATCCAACAATGTATAGCAAGGACAATGATCAATTCAACTGTATTCAGAGGGCACACCAGAATACACTGGAGAATTACCCACAGTTCCTAACTCTTTTGTTACTGGGGGGTCTTGAGCACCCTGTTGTATCTGCTGCCGCAGGTTGTGTGTGGCTGGCTGGCCGCATTGCTTATGCAAAGGGCTATTACACTGGCAATCCAGCAAAGAGAATGCAAGGTGGCTTTGCATACTTGGGGCTACTGGTACTACTTGGAACGTCGGTGAAGTTTTCTCTTCGTCTCTTGGGAGTAGTCTAA

>MAPEG4

MITLQVPSQYGYCVLVAVGSIFVLMWKGIKVGVARKKFNIPYPTMYSKDNDQFNCIQRAHQNTLENYPQFLTLLLLGGLEHPVVSAAAGCVWLAGRIAYAKGYYTGNPAKRMQGGFAYLGLLVLLGTSVKFSLRLLGVV
