# Supplementary material for: Molecular Cloning, Characterization and Positively Selected Sites of the Glutathione S-Transferase Family from Locusta migratoria
Source: PLoS One. 2014 Dec 8;9(12):e114776. doi: 10.1371/journal.pone.0114776 (PMC4259467; doi:10.1371/journal.pone.0114776)
Supplement: S3 Data — Sequences of cytosolic GSTs used for phylogenetic analysis. (DOC) [file pone.0114776.s009.doc]

>AAEL000092Xitheta

ATGCCCATGAGTTTGTATTACAGTAAGATGAGCCCTCCGGCTCGGTCCGTACTGCTGCTCATCCAGGAACTGGGACTGACTGGGATTCAGTTGAAAGAAGTGGACGTCCAGGGCGGTGGAACTCGCACCGAAGAGTTTCTCAAGATGAACCCGGAGCATACAATCCCGACTTTGGACGACAACGGTTTCTATCTCTGGGAATCCCGTGCCATTCTGACGTACTTGGTGGATGCCTACCGTCCAGGACATGACCTTTATCCAAACATTCCCCGGGAAAAGGCCCAAATCAACCGAGTTCTTCACCACGAGCTATCGGCATTCCATCCGAAAACTTTAGGCCAAATGGGTGCAATCTACCGGCGTGAAACGTCAGTTGTCACCGACGAAATGAAGGCCAAGATCAACGAAGCATACACCAATTTGGAACTATTCCTGGTTCGGAATGACTGGTTCGCCGGGGAGAACGTTACCGTGGCGGATCTTTGCCTGTTGCCAACGATTTCTACGATGGTGCACGTCGGATTCGACCTGTCCAAGCATCCACGGTTGGCCGCCTGGTACGAAAACTGCAAGGTTCTTAAAGGATACGAGGAGGACCAGGCGGTTTCCCAACAGATTGGACAGTTGTTCAAGGAACTCGTGACCGAAGGAATGTAA

>AAEL004229T4

ATGCCCCGTCCAGTGAAGTTCTTCTACGATCTGCTGTCGACGCACTCGAGAGCCCTCTACATGTTCTTTGAGGCCACCAAAATCCCGTACGATCCGATTCCGGTTTGCGCCACCAAAGGCGAGCATCTGACCGACGAGTACCGCGAGTGCGTCAACCGCTTCCAGCAAGTACCCAGCATCATCGACGATGGCTTCAAATTATCCAACGGCGTCACCATTCTCAAGTACCTGATCCGTGAGAAACTAATCCCGGAACACTGGTACCCTCGCGATAGCCAACTGCGAGCCAAGATCGATGAATATCTCGAGTGGCAGCACGACAATAGTTCAAAGGTGTGCAATGCCTTTGTCCAGGAAAAGTGGAGCCTCCTGAACATCGAAGACGAACGCTCCAGCGAACAGAAAGTCGAGGAATACCGCCGCCAGATGGAGCAGAACCTGAATCAGCTGGAACGGGAATGGTTGGTGCCCGGACGGTTCATCATTGGCGATCGGATTACCATCGCGGACATTCTGGCCGCTTGCGAGATCGAGCAGCCTAAGATTGTCGGCATGGATCCGTTCCAGGGAAGACCAAAGCTAGCGGCCTGGCTCGAAAAGGTGCGTTACACCATGACGCCCTACTACCAGGAAGCACACCAGGACTTTTACAAGTTCACCGAGAAAGCCTCGGTGAAGAATTGA

>AAEL001054D4

ATGGATCTGTATTACATGCCGATTTCTCCGCCATGTTGGTCGATCCTGCTACTCGGACGTCAACTTGATCTTACCTTCAATTTGAAGGAAATCGATTTTAAAGCCGAAGAGCACAAGAAGCCGGAATTTTTGAAGATCAATCCAGCACATACGGTTCCCACACTGGCAGTAGGCGACGGCTACGCTCTCAGCGAATCGCGTGCCATCTTGGTCTATCTGGTGGAGTCGTTGAAAACTGAAGGCCAGGAAAACTCGCTGTACCCTCGGGATGCCAAGACCCGTGGACTCATCCACAACCGGCTGGATTTCGATCTGGGAACGCTGTACCAGCGGATCATCGCCTACTGTTCACCCCAGTGGAAGAGCGGCTCCATGGGCACGGAGGAAAATCGCACCAAGGTTCAGGATGCGTTCGAATTGTTGGAAGTTTTCCTGAGTAAGACGAAGTACGTGGCTGCCGATCAGCTGACGATTGCGGACATTTCGCTGTTCGTCAGTGTGAGCTTGCTGGATTTGTGCTACTTTGATCGGTCCGGGTACGGGAAGGTCGCCGCTTGGCACGACGTTCTCAAGAAGGAGCTCGTTGGCTACGAAGACGTTATCGCGAAGGGATTCCCCGAGTGGAGGAAGCACGTCACGAAACCCGAGACGAATTAG

>AAEL001059D3

ATGGATCTCTACTATCATATTATTCCACCGCCAAGTCGCGCCGTCCTTGTATTGGCGAAAAAACTCAACATAACGCTGAACCTCATTTCAATCGACACCAGAGATGCCAACGAAATGGCCATCCTGACCGAGGTGAACCCATTGCAATCGCTCCCGACGTTGATCGACGATGGGCAGGTCATAGGCGAATCGCACACCGTTCTGATCCATTTGACCAGCTTGTTCGACAAGGAGGGAATGCTCTACCCTGCCGACCTCAAAATACGTTCGGCCATCAACGAGTTGCTCTTCTTCGACACCAACATGTACAAGTGCTTCGTGCTGTTCGCCATGCCAACGGTCATCAAGCGCCAGGATCCGAACCATGACATGCTGGAGAAGCTGCTCGTGTGCGTTAAGGCGCTGGATAACTATCTACGAGCACGCGTCTACGCTGCTGGGGACCACTTCACCCTTGCCGATCTATCCTTGGCTCATACCATTTCGTCGCTGGATGTGATCAAGGTCAAGCTGAGCGACTATCCCAATGTGGAACGCTGGATGACGAAAGTGCTGCCGGAGATGCCGCAGTTTGAGGAGTTTCAGGTCCGTGCCGAGGAAGCGCTGTCTACATTCCTGGCCAAGCAGTACGGGGCCAAGTGCATTTGA

>AAEL001071D5

ATGGAGTTGTACTATTCGCATGCATCGGCTCCCTGTAGGGCCGTCCAGATGACCGCTTTGGCCCTCGGAGTTCAGCTTAACCTGAAGGAGATCCATTTGATGAATGGGAAGGATCATCAACGTCCAGATTATGGCCGGATCACGCCTCAACACAGCATTCCAACGCTGAAGGACAAGGATCTCATCCTGTGGGAAAGCAGAGCCATTCAGATGTATTTGGTCCAACAGTACGGCAAGGACGATAGCCTCTACCCGAAGGACCCCTCAAAGCAAGCCAAAGTCAACGAAAGACTGTTCTTCGACGCGTGCATTTTGTACCACAGATTCACCGAATACTATCACGAGCAGGTCTACGGAGGGCTGGAGGGAGACGACAAGAAGCTGGCGGCGCTGGAAGATGCGGTGAAAATGCTGGATCTATTTTTAGAAGGACAGCCCTACGTAACGGGGGAGGCCATGACGATTGTCGACCTGAGCATGCTGGCAACGGTTGCCACGATGAACTGCCTCGGCTTCGAGCTGAAGCCTTATCACAACGTGTTCGAATGGTACAAACACATGAAGGATGTGGCCCCGGGGTCGAAATTCAACGAAACCGGCGCGAAGGAGTTTGCAGCATTCAAATGA

>AAEL001078D2

ATGGATTTCTACTACTTACCGGGATCGGCTCCATGTCGTGCCGTGCAAATGACTGCGGCCACCGTTGGCGTTGAGTTGAATCCGAAACTCGTTAATCTGATGAACGGCGATCAGCTGAAGCCCGAATTTCTCAAGCTGAATCCTCAACACTGTCTACCGACGCTGGTGGATGGAGATTTCGTCTTGTGGGAGTCACGTGCCATCGCGATATATCTGGTGGAACAGTACGGAGATAGTGATCAACTGTATTCGAAGCAGCCCCGTCAACGAGCCGTGGTGAATCAACGGCTGTTTTTCGACGCTACGGTACTGTATCCACGATTTGCGGAGGCATTTTACCCGGAGATGAAACTTCCGGGAGAGTCGGAGCGGGAGAAATTGGACCAAGCGGTGGAGATGTTGGACAAGTTTTTGGAGGGGAAGCAGTTCGTAGCAGGCGGGGATGGATTGACGGTTGCCGACATTAGTATTTTGGCTACTATGACGACGTTCGACGTGGCAGGTTATGATCTGGGGAAGTATCGGAATGTTGGCGAATGGTACAAGCGGGTCAGCGCTGTGACTCCCGGATGGAAGGAGAACAGAAGAGGCGCTCTGCAAGCTATTTTGTATAGGGATTTCTTCCAGGTGTATTACCCGAAGTATTTTTCTGGAGAGATGCTCGGTTGTAATGAAATGGATTCTGCGGAACTACTCAAGTTCTTAGACCAGTTTCTAGACGGTATGATGTTTTTGATAGGTGAAGCAATGTCCTACATAGATCAACAAGTCATTAGTATTGTTGATAAGATTGAGGAGACGGGAGTTAATATGGGCTTATATCCTAACGTTCTAAGTTGGACGACAAGAATCAAAAGCTACGCTTTGAAATAA

>AAEL001061D1

ATGGATTTCTACTACCTGCCAGGATCTGCCCCGTGCCGTGCCGTCCAGATGACCGCGGCCGCCGTTGGCGTGGAGCTGAATCTTAAGCTCACCAACCTGATGGCCGGTGAACACATGAAGCCCGAGTTCCTCAAGCTGAACCCGCAGCACTGCGTTCCGACGCTGGTCGACGGCGACTTTTCGTTGTGGGAGTCTCGAGCGATCATGATCTACCTAGTGGATCGGTATGCAAAGGGGGAGGTTGGCGAGAAACTGTACCCGAAAGATCCGCAGAAACGAGCAGTGGTCAATCAGCGGTTGTACTTCGATATGGGAACACTGTATCAACGGTTCGGCGATTACTACTATCCGCAGATATTCGAAGGTGCTGCTGCAAATCCGGAAAACTACCGGAAGATCGGAGAAGCGTTGGAGTTTTTAGAAGTATTTTTGCACGATCAACAGTTCGTGGCTGGAGGGAACTGCCTAACGCTAGCGGATTTGAGTGTACTGGCAACACTTACAACGTTTGAAGTGGCGGGATATGACTTTTCGGGGTACACAAGTGTTTATGAACGGTATGGAAGGATTCAGAAGGTGGCTCCGGGTGCCGACATCAATCGAGAGTGGGCAGAAGCTGCTCGACCGTTTTTCGAAAAGGTTAAAGCCCCTGCGCAGTAG

>AAEL001090D7

ATGACTCCGGTGCTATATTTCTTGCCTGCTTCGCCACCATGTCGCGCGGTCATGCTTCTGGCCAAGATGATCGGAGTAGACCTGGAGTACAAAACGCTGAATGTTATGGAAGGGGAGCAGTTGAGGCCGGAGTTTGTCGAATTGAATCCCCAGCACACAATTCCGACCTTGGATGACCACGGCCTGGTGCTGTGGGAAAGCCGCGTCATCCTCTCCTACCTGGTCTCGGCGTACGGCAAGGACGAAAGCCTGTACCCGAAAGATTTCCGCTCGCGTGCCATGGTGGACCAGCGACTGCACTTTGACCTCGGCACCCTCTACCAACGCGTCGTGGACTACTACTTCCCCACCATCATGGTAGGCGCCCACCTGGACCAGACCAAGAAGGCCAAACTGGCGGAGGCGCTCGGGTGGTTCGACGCCATGCTCAAACAGTACCAATGGGCGGCGGCGAACCACTTCACGATAGCGGATGTTACCCTGTGCGTGACCGTCTCGCAGATAGAGGCCTTCGAGTTCGATCTGCATCCGTATCCGAAGGTGCGCGCGTGGCTGGCCAAGTGCAAGGAGGAACTGGAACCGCACGGATATAAGGATATCAATCAAACCGGAGCGGAAGCTTTGGCTGGTCTGTTCCGGGCCAAACTCAAGCAATAG

>AAEL006764Delta

ACCATGGACTTCTATTATCTGCCGGGATCTGCCCCTTGCCGTGCCGTCCAGATGACCGCGGCTGCCGTTGGCGTGGAGTTGAACCTCAAGCTCACCAACCTGATGGCCGGTGAACACATGAAGCCCGAGTTCCTCAAGCTGAACACACTGGTGGACAATGGCTTCTCTCTGAGGGAATTCCGCGCCATCATCGCCTATCCGGTCGAAAAGTACGGCAAGGACGACAAACTTTACCTGAAGGACCGTGAACCAGCGGCTGTACTTCGACAAGGGTACCAGCGCTTCGTCGACTACCCGCAGGTCTTCGCTAAGCAGTCCGTTCTGGACAACGAGAAAAAGATGCTGGACGCGCTGGAGTTTCTATACAAGTTCCTTAAGGACTCGAAACACGTGGCCGGTGATAAGCTGACCATTGCCGATCTGAGCATCTTGGCGACGGTCTCCTACGAAGGGGTCAAGGTTGACCTGAGCAAGTACCCGAATGTGGCCAGCTGGTACGACCGGCTGCGCAAGGAAACCCCGGGAGCGGCCATCAACGAAGCCGGCTGCAAGGAGTTCTACAGGATATATCAGACATCTTTGTCAGCATGCACAACAGAAAAT

>AAEL007955E8

ATGTCCAAACCGGTGCTGTATTACGACGACATCAGCCCGCCAGTTCGCGGAGTGCTTCTCACCGTGGCCGCCTTGGGAATCAAAGACCAAGTCGAACTCAAACTGGTTCGCCTCTTCGAGAGAGAACATCTTTTGGAAGATTTTGTTAAACTGAATCCTCTGCACGCGGTACCTGTGCTGAAACACGACGATCTGGTCCTGACCGATAGTCATGCAATTATCATGTATTTGTGCGACATCTTCGGACAAGATGGTGATTTTTCGTTGAAAGATCCTAAACAGCGAGCCCGCGTCCACAATCGATTGTGTTTCAACAATGCCGTTCTGTTCCAAAGAGAGTCGATCGTAATGCGAGGCTTGATCAACAGATCGATAGTGACACTCGAAGACCATCACTTAAAACCCGTTCAGGAAGCGTACGACTGTTTGGAAGTTTATCTGACGAATTCGAAGTTTGTTGCTTGTGATCAACTTACAGTTGCAGATTTCCCAATCGTGGCATGCATGAGCACAGTTGGAATGGTCTGTCCGCTATCAACCAGTCGATGGCCTAAGACTGCCGCGTGGTTTGAAACCATGAAACAGTTACCCTACTATCAACAGGCAAACCAGGTTGGGGTAGACAAGCTCAAAGAGAGACTTCATGCTGTAATGAAAAAATAG

>AAEL007954E1

ATGGGCAAAATCAAACTCTACAGTTTCCTGCTGAGTCCACCTGGTCGAACGATTCAGCTAACCGCGAAAGCCCTCGACCTTGAGCTCGAATTTCAGATGAACCCACAACACACCATCCCGGTAATCGACGATGATGGGTTTGTGCTGTACGACAGTCATGCGATTGCCATCTACTTGGTATCGAAGTATGCCCCTGGAAATCGGCTCTATCCTACGAAGGACTTCAAGCAGCAGGCCCGTATCAACGCGATCTTGCACTTCGAGTCTGGAGTAATGTTCGCCAGGCTCCGATTCGTTGGAGATGCCATACAGAAGGCCAGTCACCAAGGTGAAGTTCCACAGGATCGCGTGGAGTACGCACTGGAAGCCGTTGAGCTGCTGGAAGCCCTGCTGAGGGATGGACAGTATCTAGCTGGGGACCACGTGACATTGGGCGATATCAGTTGCGTGACTTCATTCTCGTTTCTGGATGCTATGCTACCAGTGGAACGTGCCAAGTATCCGAAGGTTTACGCTTGGTATGAGCGGATGAAACACATCGAGGGATATGATGAGATCAACCAAAAAGCGGTGGATCAGTTGAACGGGGTCATTCAGGGGATTTTTGAGGGAAATAAGAGCAAATAG

>AAEL007962E4

ATGGGAAAAGTTCAACTCTACACGGCCAAGCTGAGCCCTCCGGGACGGGCAGTTGAGCTGACGGCCAAGGCTATTGGTTTGGACCTGGATGTTCACCCGATCAATCTGATCGCGGGTGATCATCTGAAGCCAGAGTTTGTCAAGATGAATCCCCAACATACGATTCCGTTGATCGTCGACGAGGATGGGACGATCGTTTACGATAGCCACGCCATCATCATCTATCTGGTGAGCAAGTACGCTAAGGACGATTCGCTGTATCCGAAGGACATTGCAACACGTGCCAAGATCAACGCTGCCCTACATTTCGATTCAGGAGTGCTTTTCGCCCGTTTGCGGTTCTACCTGGAACCCATCTTGTACTACGGAAGCCCGGACACCCCGCAGGACAAGATTGACTACGCGTGCAAGGCGTACCAGCTGTTGAACGATACGCTCGTGGATGAGTACATCGTTGGCAACCGGATGACCCTGGCGGACCTTAGCTGTATTGCTAGCATTGCATCGTATCATGCCATTTTCCCGATCGATGCGGCCAAGTATCCCAAACTGGCCGCATGGGTTCAACGCCTAGAGAAGCTACCCTATTACAAGGGAACCAACCAGGAGGGCGCCGAAGAACTGGCCGCAGTCTATCGCGATCGGCTCGCCCAGAATCGGGCCGGGAAGAAGTAA

>AAEL007951E2

ATGACGAAGCTCATTTTGTACACGCTCCATGTGAGCCCACCCTGTCGGGCAGTGGAACTCTGTGCCAAAGCGCTGGGCCTTGAATTGGAACAAAAAACGGTCAATTTGTTGACCAAAGAGCATCTTACACCGGAATTTATGAAGATGAACCCTCAACACACCGTGCCGGTGCTAGACGATAACGGTACCATCGTTTGTGAAAGTCATGCAATTATGATTTATCTGGTGTCAAAGTACGGCAAAGATGACAGTCTCTACTCGAAAGAACTGGTTAAGCAAGCCAAACTGAATGCTGCTCTTCACTTCGAGAGCGGTGTCCTGTTCGCTCGTTTGCGGTTTGTGTGTGAACCAATCCTTTTCGCCGGAGGGTCTGAGATTCCAGCGGATCGTGCCGAATATGTGCAAAAGGCTTACCAACTGTTGGAGGATACCCTGGTGGATGACTATATCGTGGGAAATTCGCTGACAATCGCGGATTTCAGTTGCGTTTCGAGCGTTTCGTCGATTATGGGAGTAATTCCGATGGATAAGGAGAAGTTCCCGAAGATCTACGGCTGGCTGGACCGCTTGAAGGCGCTGCCCTACTACGAGGCAGCCAACGGAAGTGGAGCCGAGCAGGTGGCGCAGTTTGTCCTGTCGCAGAAGGAGAAGAATGCTCAAAAGGCATAA

>AAEL007964E5

ATGACCAAACCAATTGTATACACGCTCTACTTAAGTCCACCTTCGCGAGCTGTGGATCTCTGTGCGGTAGCGCTGGGAATCGAATTGGAACGCAAAGTTATGAATCTGCTAGAGAGGGAACATTTGGACCCGAAATTTCTCAAGATGAATCCTCAACATACGATTCCGGTGCTGGATGATGGTGGGATAATTGTTCGTGATAGCCATGCCATCATGATCTATTTGGTATCCAAGTATGGGAAGGACGATAGTTTGTACCCGAAGGATTTGGCTGAACAGGCGAAAGTTAATGCTGCCTTATACTTTGATTGTGGAGTCCTGTTCGCTCGTTTGCGGTTCATTACTGAACAAATTCTGATGGGAGGAAGTGAAATTCCGGCGGAAAAGGCTGCTTACGTGGAGTCGGCTTACCAACTGCTGGAAGATGCCCTGACCGATGACTTCATCGCAGGAAACTCTCTGACTATCGCAGATCTTAGCTGCGGGTCCACTGTCTCCACCGCGATGGGATTAATTCCGATGGACCGGGACAAATATCCGAAGATCTACGCTTGGTTGAACCGCTTGAAGGCACTGCCCTACTTCGAAGAATTGAACGACCAAGGGGCTGTGGAGTTACCGGCTATTATGAAAAACCTCATGGAGACCAATGCTCGCAAGGCTTGA

>AAEL007948E7

ATGAGTTCCAAAATAGTGCTGCATACAACCCGTCGCACCCCCGGAGGACGAGCTGTTCAAATCTTGTCCCACATTTTGGGGCTGGATCTCGATCTCAAGTTTGTCGATCTGTCCAAAAAGGAACAGATGAGCGAGGAGTTTCTCAAGTTAAACCCATTCCACACGATACCGACCATTGACGACGATGGTGTTCCGGTGTACGACAGCCATGCCATTCTCGTCTATCTGGTGTCCAAGTACGCCAAGGATCGGGATCTCTTTCCGGAGGATCCCGTCATCCAAGCTCGCATCAACGCTTGGTTCCACTTCGACTCCGGAGTTTTGTTTCCGAGACTGCGTGGTGCCGTTGAACCAGTGTTCTACTTTGGTCTGAAGAAGATTCCCCGGGATCGAATGGAAGCAATTGAGGCGGCTTACGATCTATTCGAAGGAGCGCTGAAGGGCGATTTCCTGGTAGGAGATTCGTTGACGCTGGCGGATATAAGCGTAACCACTTGTTTGGTGTCCTTGAATGGAGTTTGCCCTATGGAGGAGTCTAAATATCCGAAAAGCTGCGCTTTTCTTAAACGGATGGAACAGTCAATGCCGTGCTACAAGGAGTTCAACGCTGAAGTGTTGGAAGAGACCAAGGTATTTCTAAAGCAGAAACTGGATGAAAACAATAAGAAATAA

>AAEL007946E6

ATGGCCACCAACAGTCCCCGAATCAAGCTGTACACCAATCCCATCAGTCCTCCGGGACGATCGGTAGAACTGACGGCCAAAGCAATTGACCTTGACATTGAAGTCATCGCGATTGATCTGCTGGGAAATGATACCCTAAAACCGGACTATCTCCTCAAGAACCCACAGCACACGATTCCAATGATCGACGATGGCGGTAAGTTCATTTGGGACAGCCAGGCGATCAACGTGTACCTCACAACCGTCTACTCACGCAACGAAGATCTATATCCGAACGATCCTTTCGTGCGCGCCAAGGTGAACGCCGGTCTGCATTTCAACTCCGGAGTGCTCTTCAGCCGTCTGAAACTGTTGATCTCCCCGGTGATTCGTGGATTCAAGCAGGACCTGGACCCGGAAAAGGTGGAATACTTTAACATTGGGCTGCAGCTGCTCGAGGATACGCTGCACGCTGATTACTACATTGGCAATCGGATGACCTTGGCCGATTTGAGTTGCGTTTCGTCGGTGTCCAGCTTCGATGCGGTTCTGCCGCTTGATAATTCTAGATTTCCAAAAACCGTCGACTGGTTGAGACGGATGGAACAATTGCCGTACTATGGGGAAGCGAATGGAGAAGGTGCAAAGAAATTGGCGAAGGTTGTACAGAGCTTTCTGAAATAA

>AAEL007947E3

ATGTCCCCCATTACGTTGTACACTACTCGGCGCACACCGGCCGGCAGAGCTGTGGAAATCACCGCTAAGCTCATTGGCCTGGAACTGGACGTTAAGTTTATTGACTTGTCCAAGAAGGAACATCTGACTGAGGAGTTTCTGAAGTTGAACCCACAACACACAGTGCCAACGATCGTTGATAATGGAGTCGCTCTGTACGATAGTCACGCAATCATCGTCTACCTGGTACAGAAATACGGCAAAGATGACGCCCTGTATCCGAAAGATCTGGTGACGCAAGCTCGTGTCAACGCATTGCTGCATTTCGAGTCGGGCATTTTGTTTGCTCGATTGAGAGGTACCTTGGAACCCATCTTCTACCATGGGTTCCCGGAGGTTCCCCAGGAAAAGCTAACCGCCATCCACGGAGCGTACGATCTGCTAGAGGCCACCCTGAAATCAGACTATCTGGTTGGCGACTCGTTGACCCTTGCCGATGTCAGCTGCAGCACATCGCTGTCCACTCTGAATGCCCTGTTCCCAATCGACGCCGAAAAATGCCCAAAATTGGTTGCGTATCTTCAGCGCTTAGAAGCGAACATGCCGAACTACAAGGAGCTGAACTCGGATCGTGCTGCCGAAGCGGTAGCATTCTTCAAAGTCAAGCTGGAAGAAAACAAGAAGAAATGA

>AAEL009020T3

ATGGCCAGCCGTGCTCTTAAATACTACTACGATCTGCTATCGCAGCCATCCCGCGCCCTGTACATCCTGTTGGAACAGACGAAGATTCCGTTCGAAAAGTGTCCGGTAGCGTTGAGGAAATTTGAAAACCGTTCGTCAGAATTCGTCCAGAACGTGAACCGCTTTGGGAAGCTGCCCTGCATAATCCATGGCGATTTCAAGCTGGCGGAGAGTATTGCAATCTTTCGATATCTGTCGCGGGAGTTTCAGCTTGAAGATCGATGGTATCCGAAAGAGGGCGCGGATCGTGCCCGGGTAGATGAGTATCTGGAGTGGCAACATGCCAACATCCGGGCCCAGTGTGCGCAGTTCTTCATCTACAGCTGGATCACTCCGCTGCTGGGGATGGAAGTCAATCAGGCGAAGGTCGATAGGCTGCGAGCGAACATGATCGAGTGTTTGGACGTTTTCGAACGGGAGTGGCTGGATGAGGGCCGGAAGCCGTTCGTGGCTGGGAAGGAACTGAGCTTTGCCGATGTGGTGGCGGCTTGCGAGATTGAGCAACCGAAGCTGGCTGGGTTCGATCCCAGAGTGGGGCGACCGCATTTGGCGGCTTGGATGGAACGGGTCAGGGTGGCCACTAATCCGCACTACGATGAGGCGCACAAGATTTTGTATAAGTTCACACCGAAGGAGATCGAAACTCCAAAGGTGCAATAA

>AAEL009017T1

ATGTCGAAGCTACGGTACTTTTACGACCTTATGTCACAGCCCAGTCGGATGCTGTATATCTTTCTGGAGTCAACGAAGATCCCCTACGAACGGTGTCTAGTGAATCTGGGAAAAGGCGAGCATTTGACCGACAAGTTCAAGGCGATCAATCGCTTCCAAAAGGTCCCGTGCATCGTCGACAAAAACGACCTGCATCTGGCGGAAAGTGTTGCCATCGTGCGGTATTTGGCCCGGGAGTATCCATTCTCGGATCATTGGTACCCCAAGGACAGCCAGAAGCGAGCCCGGATCGATGAGTATCTGGAATGGCAGCAGCACAATACGCGGGCCGTTTGTGCCACCTATTTCCAGTACGTGTGGCTGCGGCCAAAGTTGATGGGAACCAAGGTCAATCCGGAACGGGCCGAGGAGTACAAGCAGAAGATGGAGGACTGCTTGGATTTCATCGAGAGTGATTATCTGGGCGGAGGCAATCCCTTCCTAGTGGGCAATGAGATTTCCGTTGCGGATCTGTTTGCGGCGTGCGAGATTGAGCAACCAAAAATGGCTGGCTTCGATCCGTGCGTAGGGCGACCGAAAATGACTGCTTGGATGGCCCGGGTTCGGGAGGCAACCAATCCGCACTACGATGAAGCGCACAAATTGGTTTACAGGATTGCTCCGGATAGCGTTCCGAAGCCGAAGTTGTAG

>AAEL009016T2

ATGGCAAACGGTCGCAGCATTCGCTTCTACTACGACCTGATATCGCAACCTTGCCGAGCTTTGTACATTTTCCTTGAGCAGAACAAAATCCACTATCAAAAGTGTCCCATTGCGCTGCGGAAATGGGAGCACACAACGCCCGAATATCTGCAAAATGTAAACCGTTTCGGTAAGGTGCCCGCCATTGTGGACGGAAAGAACTTCAAATTGGCGGAAAGTATCGCCATTCTGCGATATTTGGCACGAGAGTTTACCGTGCCGGACCATTGGTATCCGAGGGATAGCCGTAGGAGAGCCAGGGTTGACGAGTACTTGGAGTGGCAGCATTCGAACACCCGGTTGCACTGCGCTGGGTATGTGAGGTACGTGTGGCGAGGACCTCTGCGAGGGGAAACCATGGACCCGAGGGTTGCCAAACGGTTGAAAGCGGAGATGGTCGGTTGCTTGGATTTCATCGAGACGAACGTTTTACAGCGGGACGTGCATTTCATAGCAGGCGATGAAATCTCGATCGCTGATCTGGTGGCGGCTTGCGAGATCGAACAGCCAAAACTAGCCGGGTACGATGCACGTGTCGGTCGACCGAAGCTGACAGCGTGGATGCAAAGGGTAAAGGAAACCACCCAACCAGACTACGATGAGGCGCATAAGGTTTTGAATAAATTTGCGCCTACTGCGACGTGA

>AAEL010500X2

ATGGCTCCAATTGTGCTGTATCATTTCCCCATGTCGCCACCATCCCGGTCGGCTCTGCTGGTGGCCCGCAATCTCGGATTGGATGTAGAGGTCAAGATTTTGAACTTGATGGCCGGCGAGCACATGCAGGAGGAATTCGTCAAGATTAATCCCCAACACACGGTCCCGACTGTCGTCGACGACGACTACGTCCTGTGGGAATCGAAAGCCATCGCCACCTACCTGGTGGAGCAGCACCAGCCGGACAGCACATTGTACCCGGCGGATCCGAAACAGCGAGGCATCATCAACCAACGGTTGTACTTTGATTCGACTGTTCTGTTTGCCCGCGCTTATGCTGCTGTCGCTCCGTTAATGCGCCAAGGTGCCACCTCGATCCCACAGGACAAAAAGGACGCCATCCTGGAAGCCCTAGGAACGTTGAACGGATACCTCGATGGACAGGATTGGGTCGCCGGCGAAAATACCACCGTGGCCGATTTGTGCCTTCTGGCAACCGTGTCATCTCTAGAGAAACTTGGTGTTGACCTGAGTGATCTGCCGAACATCACTGCCTGGCTGGAACGCTGCAAATCTCTGCCGGGATTTGAGGAAAACGAAGAGGGTGCCAGCATGTTCGGAAATGGACTGAAATCCAAGCTGGAGGAACCTTTCTAA

>AAEL010582D11

ATGGCCCTGAATCTGTACTATATGCCGCTTTCGGCACCCTGTCAATCGATTCGCCTACTGGCGAAAGCCATGAATCTGCACATAAACCTGACCTACTTGGATCTCATGAAGGGCGAGCACATGAAGCCGGAGTTCCTGAAGATAAACCCGCAACACGTCATCCCAACGCTGGTGGACAACGATTTCGTCCTGTGGGAGTCGCGTGCCATATTGATCTATCTGGTCGAGAAATACGGCAAAAACGATAAGTTCTACCCGCGTGACCCGAAGAAGCGAGCCGTTGTCAATCAGCGGTTGTACTTCGACATGGGCACGCTGTATGCTCGGTTCGCCGAATACTACTACCCAGCCATATTCGAAAACAAGAAGTTCGATGAAGAAAAGTTCAAGAAATTGGAAGAGGCCTTCGAATTTCTGGAAGTCTACCTCGGAAAGACCACGTACGTTGCCGGCGATAAGCTGACCGTGGCGGATTTCTGCATTTTGGCTTCGATCACCACGATGAAGGTGGCAGCGGAGATCGATTTCTCCAAGTATCCCAACATCGAACGTTGGTATGCCCAACTCAGCGAAGAAGTGTCCGGCCATGATGAAGTGTGCGTCCAAGGAGCTCAGGAGATGAAACCATTCTTCGAAGCTGCCCGAAAGGCGGCTGCCGAGGAGCAATAG

>AAEL010591D6

ATGCCGCTGGATCTGTACTGCCACATTGTGGCACCGTTCTGTCGCTCCGTTATCCTGTTGGCCGATGCCCTTGAAGTGGAACTGAATTTCATCGAAGTAAACGTGTTGAAGAAGGAACAATTCAAGCCAGAATTCATAGCGATGAATCCTCAGCACTGTATTCCGACGCTGGTGGATGGTGACGTCGTAGTGTGGGAATCGAACGCGATACTGATTTACCTAGCTGAGAAATACGGCAAAGTAAGCAAACGGTTCTACCCAACGGACATTGCAGAACGCGCCAAAGTAAATCGAATATTATTCTTCCAATTGGGGACATTGCACCGAGCGCTGTCCACCTACTACTACCCAATTCTGGCTGGGCTCGGCGAAGGAAAGCCGGAAGACTTCCGAAAGATACAGGATGCGGTTGGTGTGATGGACAAACTCCTCGATGGAAACAAATGGCTTGCGGGAGAAGACCTATCAATAGCGGATTTTAGTGTGGTTATAAGCGTTGCCTCCCTCGAAGGGGTAATCAAATTCGATCTGACCGTGTACAAAAACGTGTACCGTTGGTATCAGCAGTGCAAGAAAGAGTTTCGAAAATTCGAAGAACTCACACAGGAAGCCAATGACAAATCCCAGGAAATGATTGCAGCCTTACGTCAGTATAAGTTGGAGGAAATAAATTCCGCTAAAGAGCCGTGCTGTAGTGCACCCCCGGGAGCGAAAACTCCCCCGAAGCCACCCTGCCCGGACTCATCTTGA

>AAEL011741S1

ATGCCGGATTACAAGGTCTACTACTTCAACGTCAAGGCCTTGGGCGAGCCTCTGCGGTTCCTGCTGTCCTACGGCAATCTCCCGTTCGATGATATCAGAATCACCCGCGAAGAATGGCCAGCTCTGAAACCAAGTATGCCCATGGGACAGATGCCGGTGCTGTCGGTGGACGGCAAGAAGGTGCACCAATCGGTGGCCATGTCCCGCTATCTGGCCAAGCAGGTCGGCCTGGCCGGTGCCGACGATTGGGAAAATCTCATGATCGACACCGTCGTCGACACCATCAACGATTTCCGTCTGAAAATCGCCGTTGTGTCCTACGAGCCCGATGATGACGTGAAGGAGAAGAAGCTGGTCACCCTGAACAGTGAGGTCATTCCATTCTACCTGGAAAAGCTGGACGACATCGCCCGTGATAACAACGGCCACATGGCAAATGGCAAACTCACATGGGCCGATATGTACTTTGTCGCCATCCTGGACTACCTGAACTACATGACCAAGTCCGACCTCGTGGCCAATCACCCGAACTTGCAGAGAGTGGTCGACAATGTCACGAGCATCGATTCGATCAAGGCCTGGATCGATAAGCGACCACAAACTGAGATCTAA

>AAEL011752Lota1

ATGAAAATCTATGCCGTATCGGATGGACCTCCCTCGTTGGCGGTCCGTATGGCCCTCAAAGCGCTAGACATTGCTCACGAACATGTCCCCGTCGACTACGGCAAGGGAGAGCACATGACGGAGGACTATGCCAAGATGAATCCACAAAAGGAAATCCCGGTCTTAGATGACGATGGGTTCTTCTTGAGTGAAAGCAATGCCATTCTACAGTATCTATGCGACAAGTACGCTCCGGACAGTCCCCTGTATCCTAAGGATCCCAAGGAGCGTGCCCTGGTCAATCATCGCCTTTGCTTCAATTTGTCGTTTCTGTACCCGCAGATATCGGCTTATGTGATGGCACCGATTTTCTTCGATTACGAGCGGACTCCGATGGGGCTGAAGAAGCTGCACATTGCTTTGGCCGCTTTTGAGACCTACATGAGCCGCTTGGGAAGTAAGTTTGCCGCTGGAGATCATCTCACGATTGCGGATTTCCCATTGGTCACATCTGTGATGTGCTTGGAAGGAATCAATTTCAACATCGATCAATACCCGCTGGTGAAGGCATGGTATGCCAACTTTAAGCAACAATATCCCGAGCTATGGGCCATTTCCGCTGTGGGAATGGCGGAAATTACTGAATTCGAGAAGAATCCTCCAGATCTATCAGGCATGGAGCACCCAATTCATCCCATCAAAAAAGTTAAGAAATGA

>AAEL011934z1

ATGTCGTCCGCAAATGATAATGTTGTTGTGTCCGATGTTTCTTCCCTGGCGGAAAACGCCCTGCAGCCGATACTGTACTCGTACTGGCGCAGTTCCTGCTCGTGGCGGGTGCGGATAGCGCTCAATCTGAAGGAGATTCCCTACGACATCAAGCCGATCAGTTTGATCAAATCGGGCGGCGAGCAGCACTGCAACGAGTACCGCGAGGTGAACCCGATGGAGCAGGTACCGGCGCTGCAGATCGATGGCCATACACTGGTGGAATCGTTGGCGATCATGCACTACCTGGAAGAGACGCGTCCCCAGCGTCCTCTTCTGCCGCAGGACGTCCTGAAGCGTGCCAAGGTTCGGGAGATATGCGAGGTCATCGCCTCCGGCGTCCAACCACTTCAGAACCTGATCGTGCTGATCCACGTCGGCGAGGAGAAGAAGAAGGAATGGGCCCAGCATTGGATCACCCGAGGATTCCGCGCCATCGAGAAACTGCTCTCGACCTCGGCCGGGAAGTTCTGCGTCGGTGACGAGATCACCCTGGCCGATTGCTGCTTGGTACCGCAGGTGTTCAACGCTCGCCGCTTCCACGTGGATCTACGCCCGTATCCGATCATCCTCCGCATCGATCGGGAGCTGGAAGGACATCCGGCATTCCGCGCGGCACATCCCTCCAATCAACCGGACTGTCCACCGGAAGCGGCCAAGTAG

>AAEL015336GST

ATGCCATTGATTGAACTAATATTGATTTTTTCGCAGGTTTTGTGGGCATTACACGAAAAAGATATAAAATTCACAAAATATGAAATTGACGTCGCAAACGATGAGCACTTTTCTGAATGGTTCCTAGAGCTGAATCCCCGGGGAGAGTTGCCAGTACTGCAAAAAGGCCTTCTTATCGTGCCAGGTTCTACCCGGATTTTGGACTATTTGGAAGAAAACTATCCGAACAACAAACCTTTAAAGTTACGTTGGTGCGAGAATGTGAAATTACAAAATCTCAAAAATTCGCTGGATAAGTTACCAATAGGTGTTATAACAATGGGTTCGTTCCTCCATCCAAGGACAGTAGTAGCTCCCAAATCGCCCTTTGTTCAACCAGTTCGATACACCATTCTCGAGCGGGACGAATCAGTCAGCAATCGCTTGAGAGGCTACGCAAAGGCATTTCCAGCCTTCAGTGAGGTACTGCAGAAGAAGGCAGAGTTTCACGATCGAAAGCGCCAAATGCTGGCCAGCGAGCAGTACTTTTTACAATTGTTGGACGTTTTGGATCAAGTTCTGGCAGATGCCGAGGCGGAACTGACAAAATCGGGCGACGATAAGATTTGGATCGCGGGAGCAGACATCAGTCTGCTAGATATTTCATTGGGATGTCTACTGTATCGTTTGTACGTTCTTGGCTTGGAGGATCGTTTCTGGACAGTCGGGAAGAAACCACAGCTGGAGAAGTACTTCAATCGAATTATGGCCTCGGAAAATTTCCAGCATACCCTGCCTACGAAGACATCCCTGCTCAAAACGATTTGGTTGAACACGCCATCGACGTACAAAGCCGGTATTGCAGCATTTTCCTTTTCCTCGATGATCATAGGATCGACCCTGCTCAAGAGATAG

>AAEL017085o1

ATGAGCAACGGAAAGCATCTCGCCAAAGGTTCCAGCCCACCATCCCTCCCGGACGATGGTAAGCTGCGCCTGTACTCGATGCGCTTCTGCCCGTACGCCCAGCGCGTCCACCTGATGCTGGACGCGAAGAAGATCCCGTACCACGCGATCTACATCAACCTGTCCGAGAAGCCGGAATGGTACCTGGAGAAGAACCCGCTCGGCAAGGTGCCGGCACTGGAGATTCCCGGCAAGGAGGGCGTCACCCTGTACGAGTCGCTCGTCCTGTCCGACTACATCGAGGAGGCGTACTCGGCCCAGCAGCGCAAGCTGTACCCGGCCGACCCGTTCAGCAAGGCGCAGGATCGCATCCTGATCGAGCGGTTCGCCGGCTCGGTCATTGGGCCGTACTATCGCATCCTGTTCGCGGCCGACGGCATCCCGCCCGGTGCCATCACCGAGTTCGGCGCGGGGCTGGACATTTTCGAGAAGGAGCTGAAGGCGCGGGGCACCCCGTACTTCGGGGGCGATAAGCCGGGCATGATCGACTACATGATCTGGCCGTGGTGCGAGCGGGTCGATCTGCTCAAGTTTGCGCTCGGCGATAAGTACGAGCTGGACAAGGAGCGGTTCGGTAAACTGCTGCAGTGGCGAGAGCTGATGGAAAAGGACGACGCCGTAAAGCAGTCGTTCATTTCGACCGAGGACCACACGAAGTTCCTGCAGAGCCGCAAGAACGGCGAAAACAACTACGATATCTTGGCGTAA

>AAEL009602Ndomain

ATGGTGGATGAACCCAAATTTAAACGCCCAGAAGTCGTCAATGATGATTCATTGGTTCTATATTGCAACCAATACAGTTACTACTGTCAGAAGATTTTGTGGGCATTACACGAGAAAGATATAAAATTTACAAAATATGAAATTGACGTAGCAAACGATGAACACTTTTCCGAATGGTTCCTAGAGCTGAATCCCCGTGGCGAGTTGCCAGTACTGCAAAAAGGCCTCCTTATCGTGCCAGGTTCCACCCGGATTTTGGACTATTTGGAAGAAAACTATCCGAACAACAAACCTTTAAAGTTACGTTGGTGCGAGAATGTGAAATTACAAAATCTCAAAAATTCGCTGGATAAATTACCAATAGGTGTTATAACCATGGGTTCGTTCCTCCATCCAAGGACAGTAGTAGCTCCAAAATCGCCCTTTGTTCAACCAGTTCGATACACCATTCTTGAGCGGGACGAATCAGTCAGCAATCGCTTGAGAGGCTACGCAAAGGCATTTCCAGCCTTCAGTGAGGTACTGCAGAAGAAGGCAGAGTTTCACGATCGAAAGCGCCAAATGCTGGCCAGCGAGCAGTACTTTTTACAATTGTTGGACGTTTTGGATCAAGTTCTGGCAGATGCGGAGGCGGAACTGACAAAATCAGGCGACGATAAGATTTGGATCGCGGGAGCAGACATCAGTCTGCTAGATATTTCATTGGGATGTCTACTGTATCGTTTGTACGTTCTTGGCTTGGAGGATCGTTTCTGGACAGCCGGGAAGAAACCACAGCTGGAGAAGTACTTCAATCGAATTATGGCCTCGGAAAATTTCCAGCATACCCTGCCTACGAAGACATCCCTGCTCAAAACGATTTGGCTGAACACGCCATCGACGTACAAAGCCGGTATTGCAGCATTTTCCTTTTCCTCGATGATCATAGGATCTACCCTGCTCAAGAGATAG

>009630Sigma

ATGACAGAAAAATATAAATTGATTTATTTTAATGTAACGGGATTAGCAGAACCGATTCGTTATTTGTTTGCTTATGGTAAAATTCCCTACGAAGATTGTAGATTAGAATACGAAGAATGGCTACGTTACAAACCAGAAACGCCATTCGGACAAATTCCTGTTTTAGAAATAAATGATAAACCGATTAATCAATCCGTTGCCATTTGTCGTTACCTCGGTAAACGTTTAAATTTAACCGGAAGTTCCGATTGGGATTCTTTTCTTATTGATTCCGTCGTCGACACTATAACGGATTTTCGACTAAAGGAAAAAGAATCAAAGTTGAATGAAATTAAAAATGTACACAATCCATATTATTTAGAACGTTTGGAAAAAATAGCATTGAAAAATGGCGGATATTTTGTTAACAATCAATTAACATGGGCAGACATATTTTTCTCAGGACTTTCTGATCTTTGGATCAAACTAATTAAAGAACCGATTTTAATGTCTTATCCAACACTTAAATCATTAAAAATGAAAGTTGAATCCGAACCAAATATAAAAACTTACCTCGATAAAAGACCTGAAACCGTTTACTGA

>097960DeltaPartial

ATGACAATAACACTCTATCATTTTCCGCCAAGTGCTCCATCCAGAGCAGCCTTACTGACTCTTAGAGTACTAAATCTTGATTTTAATATTGTAGAAATAAATCTTTTCAAAAAAGAACAACTTCAACCGTCANNNNNAAGCAGAGCAATAGCAAGTTACTTAGTAAGAAAATATGGAAAGGATGATTCTTTATACCCTAAAAATCCACAATTAAAAGCTGTTGTTGATCAAAGATTATATTTTGATGCCACAGTTCTTTTCCCTCGAATTAGAGCAATATGTTTTCCAGTAATTTTTTTGGGTGAGACAATGATTACTGATGAAAAAAAGAAATCTTTGACTGAAGCTTTAGGATTTCTCGAAAGATTTCTTCATGGAAGGAAGTGGCTAACTGGAGATACTTGCACCATTGCTGACTTATCAACATTCGCATCATTAAGCAGTGCATTGTCTATTGGATGGGATGTAAGTCCTTATTCGAATATTACATCTTGGATCGAAAGATGCAAAACACTCCCAGGTGCTGATGAAAATGAAAGTGGTGCTAAAATTTTTGGCAATGCAGTGCTCAAAAACTTGAAACCTGGTCAGTTATAA

>189430Delta

ATGCCCATCGATATTTACTACCTTCCCGGAAGTCCACCGTGCAGGGCCGTACTTCTTACCGCCAAAGCATTGGGACTCGAAATGAATCTTAAAAGACTTGAACTCATGAAAGGTGAACATTTGAAACCCGAATTTTTAAAAATAAATCCTCAACACACGATCCCAACGATAATCGACAACGGTTTTTGCATGTGGGAAAGTCGTGCCATAATGGCTTACCTCGTCGATCAATACGGAAAAGACGACAAACTTTATCCGAAAGATATTAAACAAAGAGCACTCGTCGATCAGAGATTGTTTTACGATGCCACTAACCTTTACCCGAGATTAGGAGATTATTACTACCCCTACATGTTCGGAAGAGGTCCCATGGAACCTGCGAAAAAGGAAAAACTCGATCAATCTTTCGAAGTTTTAAACAATTTTCTCGACGGACAAAAATGGGCTGCGGGTCCGAACATGACCATCGCGGATTTGACTTTAGCCGCGAGCATATCCACGTGCGATGCTTTAGGATACCCGATTGAAAAATATCCCAACGTTCAATCTTGGTATACGAGAATTCAAAAGGAAGCACCCGGTTACGAAGTTAACGCGTCCGGTACCGAAGCATTCAAACAATTGTACATCACTTTGACATCTAAAAAATAA

>189440Delta

ATGGAATTGTATTATTACGAAATGAGTTCACCGTGTCGAGCCGTTTTACTTTTCGTTAAAGAATTAAATTTAAATCCGGTAATGAAAAAAATAGATTTGTTAAAAGGTGAAAATTTATCTCCGGATTATCTCAAGTTGAATCCTCAACACACGATACCTTTGATAATTGACGATGGATACGTACTTTCGGAAAGTCGAGCCATTCTCGTTTATTTGGCCGAACGATACGATCGAGATTCGATTTATTATCCAAACGATGCGAAAACTCGAGGAGTCATCAGTCAAAGGCTCATGTTCGACATAGGAACTCTGTCTTCACGTTTTTACGACACATATTCGGAATTTTTTATTCCGAACGGTTCGAATAGAATCGATTTGAAAAAAATAAATAAATTGGGAGAGGCTTTCGAATTTCTGGAAGCTTTCATGGGTGATTCTTTGTTCATGGCGGGAAACGTCATGACGATTGCGGATTATTCGATCGTGGCAACGGTTTCGACGATCGAAGCTTGCGGATTCGATTTTTCGAAATACGAAAGGGTTTCGGCTTGGTTCGAAAGATGCAAAACCGAAATGTTGGATTACGAGGAAATAAATCAAAAGGGAGCCGATTTAATCGGAAACATGGTGCAACCTTATTTAGAAAAGAGTCAGGCAATGAATTACGAATAA

>236630Zeta

ATGTCAGTTATAGGAAAGCCAGTATTATATTCATATTGGAGAAGTTCATGCTCATGGAGAGTAAGAATAGCATTAAATTTAAAAGAAATTCCCTATGATATTAAGCCAGTATCTCTTATTAAATCTGGTGGAGAACAGCACTCCAATGAATATAGAGAAATTAATCCAATGGAGCAAGTGCCAGCTCTGCAGATTGATGGCCACACTTTTGTAGAATCATTAAGTATTATGCATTACCTTGAAGAAACAAGACCAGGTCGTCCGTTAATGCCAGCTGATGTTGCTAAACGTGCCAAAGTTCGAGAAATATGTGAAGTAATTGCATCCGGAATACAACCTCTACAGAATTTGATTGTTCTTATTTATGTAGGAGAGGAAAAGAAAAAAGAATGGGCTCAGCATTGGATCAATAGGGGATTTAGGGCTGTTGAAAAACTCCTTTCTGCCAGTGCTGGAAAATATTGTGTTGGAGATGAAGTTACTCTTGCTGATTGCTGTCTTGTTCCACAGGTCTTCAATGCCAGGAGATTTCATGTTGATTTGCGACCATTTCCAATAATTCTTAGAATTGACAGGGAATTAGAAAATCATCCTGCATTTCGTGCTGCTCATCCTTCAAATCAACCAGATTGTCCTCCTGAAGCTACAAAATAG

>284550Sigma

ATGAATCCAGAATATAAACTTATTTATTTCAATGCAAGAGGTAGAGCTGAACATATCAGATTTATATTTGCATATGCAGGAGTAGATTATGTGGATGAAAGAATATCACAAGGAGATTGGCCAGAATATAAAAAAAAAATGCCATTTGGAACGGTTCCCGTCCTTGAAGTGAATGGAAAAACAATTGCTCAAAGCAATGCCATAGCAAGATATTTGGCTAAAAAATATAATTTAGCCGGTAAAAACGATTGGGAATCATTGGAATGTGATGTTTTGATAGATTCATTGTCCGATATAAAACAAGTTTTAATGCAATATAGAAACGAATGCGATCCGTTGAAAAAAGAAGAGAAAAAAATTATTTTGATGAAAGAAACAATACCATTTTACATGAATAAATTTGAAGATATTATAAGTAAAAATAATGGATTTTCCGTTGGTGAATCGGTGACTTGGGCCGATTTTGTATTTGCGTCTTCCCTTGAAAATTTCGAATGTATTTTTGGTAAAAATGCATTGGATCGTTATCCGGCACTTAAAGCATTAAAAGAAAAAATTTTTAATATACCTAAAATAAAAGAATGGGTACAAAAAAGACCGCAAACGGACTTTTAA

>284560SigmaPartial

ATGGCGCCTAAATACAAGCTTACATATTTTAACTTCACTGGTTTAGGAGAGCCTATTCGTTACATGTTGGCTTACGGTAATCAAGATTTCGAAGACAACCGGATCGAAATGGCCGATTGGCCAAAATTGAAACCAAACTATTCGGCCTACTTCAGAGAACCAACCGAAGAGGGTAAAGCTAAAAAATTAGAAGACGTAAGAAACGTTCACAATCCAAATTTTCTTTCGAAATTCGAAGAAAGAGTCAAAAATAACGGCGGTCATTTCGTTAACGGCCAACTTACTTGGGCAGATCTTTATTTCTCGGCTGTCGTTGACCTCATGGTAAACGTATTGAAAGAACCAATTTTGGACAAATATCCAAACTTGAAAGCTCTCAAAGAGAAAGTTGATTCTCTTCCCAGCATCAAAGCTTATCGTGAAAAGAGACCAAAAACACTTTTTTAA

>284770Sigma

ATGAGTCCGAAATACAAGCTTATTTATTTTCCTGTCACCGCATTGGGTGAACCCATAAGATTTTTATTATCCTATGGAAATTTAGAATTTGAAGATTATCGTTTTAAACGAGAAGATTGGCCGAAAATCAAACCTTCCATGCCCTTTGGTAAAACTCCCGTCCTTGAAATTGACGGTAAGCAAACCCACCAATCTGCCGCCATTTGCCGGTATTTGGCGAGACAACTTAAACTGACCGGTAAGGACGAATGGGATGCATTCAACGTTGATATGGTAATTGATACGATAACCGATCTTAGACAACAATTGGCCAATTATTTTTACGATACGAATCCGACAACCAAAGCACCCAAATTGGAACCATTACAAAAAGAAACTGTGCCTTATTATTTGAAACGTTTGGATAAAATTGTCGAAGAAAATGGCGGTTATTTTGTTGGCGGACAGCTTACCTGGGCTGATTTGTATTTCGTTGCCGTTTCGGACTATTTGCAAAAAATGGCAGAATTAGATTTTTTCTCAGATTATCCAAATCTTCAAAGTCTCAAGAATAAAGTTGTCAACATTCCGCAGATAAAAAAATGGATAGAAAAACGTCCGGCAGATAATTTGTAA

>333090Delta

ATGGTGCTTAAATTGTATTCCGTTTCTGATGGACCACCTTCATTGGCTGTGAGAATGGCATTAAAATATTTAGGTAAAGAATACGAATTGATAAACGTTGATTTTTTATCTGGTTTTCATACGACGGAAAATTATGCCAACATGAATCCGCAAAAAGAAATTCCAGTTTTGGATGACGATGAATATGGTATGCATTCTTTCACACCTTTCATTTGTTTTTCCAGCAATGCCATACTCCAGTATTTGGCAGATCGTTATCCAAAAGATGATACACTATATCCTCAAGACCCGAAAAAACGTGCCTTGGTCAATCATAGGCTGGCATTTAATTTATCTACGTACTACAGTAATATAGGTCAGCATGTGATGTTGCCAATATTTTACAAATATAAAAGAACTCCATTAACGTTAAAAAAAGTTAACATGGCTCTTGATGTTTTTAACACTTATCTGAAGAAACTGAACAAAAAATATTCTGCCGATGCCATCAATTTTGATTTTTCTCCATATACCAAGGTATATAACCCACGTGATCAAATGTTAATAAACGTTTGGGTCGTCGAATGGTATTCCAGATTTAAAAAAGAATATCCAGAACTTTGGGAAATTGCAGAAAAAGGAATGAAAGAAATTGAAAATTTCAATAAAAATCCTCCCGATTTGTCTCATTTAAATCATCCAATTCATCATTTTCAAAATTCGTAA

>454040Theta

ATGGTTTTAAAACTTTACGTTGATTTATTGTCACAACCGGCAAGAGCTTTGGCTCTTTTTGTTAAAAAAG

CAAATATTCCTCACGAATTGAAAATTGTTTCTATAATGAAAGGTGAGCACAAAACTCAAGAATATATTGACAATGTTCATCCTTTTGGAAAAATCCCTGCAATCGATGACAATGGTTTTAAAATGATTGAAAGTATAGCCATCATAAGGTACCTTGCCAGAAAGTATAATGTAGATGATCATTGGTATCCAAAGGATATTGAAAAACAAGCTAGAGTTGATGAATTTCTCGAGTGGCAACACATGTCGGAAAGAATACCGCTTTCATTGTTTTTTCTGAACACATATTTGAAACCAATGTTAACTGGGAAAAAACTTGATGACAATCGTTTACAAAAATACAAAAGTCAAATGGAAGATTCATTAAACGATATTGAAAATGTTTGGTTGAAAGATAGTCAATATTTAGTCGGCAATGAAATAAGCGTTGCAGACCTTGTTGGAATATCTGAAATCGAACAACCCAGAATTTCAAATTATGATGTAACTGCTGGAAGACCGAGAATAGCAGCATGGATGAAAAGAGTTCAAAATGATCTACAACCTCATTACGATGATGTGTTTAAAATTTTAAACAAATTGGCGAGTAAAAATGCATCAAAATTGTAA

>530530omega

ATGGGGGATAGTCAACCTCCGTTAAAAGATGCCTGCCTTAGATTATACAGCATGAGATTTTGTCCTTATGCTCAAAGAGTGCATTTAGTTTTGGATTCTAAAAATATCAAGTATGAAGTAGTCAACATTAATTTATCTGACAAACCGGATTGGTTTTATGACAAATCACCGGAAGGAAAAGTTCCAGCTCTTGAATTTCCCAATGGAGAAACATTGTACGAAAGTCTCATCATTGCCGATTACTTGGATGAAAAATATCCAGAACGTCCATTGTATCCGAAAGATCCATTGGCAAAAGCAAAGGATAAACTTTTGATGGAAAAATTTAACAGAGTTATAAGAGGGATATATAAAATGTTGCTTCACGAAGGTTTCAGTCCGGAAATAATGGATGACGTCAAAGCAGGTTTAGATTTATTCGAAAAGGAAATGTGCATTCGAAAAACTAAATTTTTAGGAGGCGAGTATCGAAAGACAAAAAAAAAAATCTTATTCGGTTCCAAACCCGGCATGTTAGATTTCATGATATGGCCGTGGTGCGAAAGAGCGGATATACTTAAAATCGTCGGCGGAGATTTGTACAAACTTCCAAAGGACAGATTTCCAAAAATTCTCGAATGGAGAGTCAACATGATTGCCGATGAAGGAGTCAGAGTGAGTTATTTGGATCCGGAAATCCATGCTAAATATTTCATGAGTAGACAAGCAGGAACTCCCGACTATGATCTTCTTCACAAAATGAAACATTAA

>CG17524E3

ATGGGAAAACTTACGCTTTACGGAATCGACGGTAGTCCTCCGGTCCGTTCGGTGCTGCTCACGCTGAGAGCCTTGAATTTGGACTTCGACTACAAGATCGTCAATCTGATGGAGAAGGAGCACCTGAAGCCAGAGTTCCTCAAGATCAATCCCCTGCACACCGTGCCCGCTCTGGATGATAATGGTTTCTATTTGGCCGACAGCCATGCTATTAACTCCTACCTGGTGAGCAAGTACGGCCGGAATGATTCTCTATACCCCAAGGATCTGAAGAAGCGAGCCATTGTCGATCAGCGTCTGCACTACGACTCCAGCGTGGTGACCTCGACAGGAAGGGCTATCACCTTTCCGCTCTTCTGGGAGAACAAGACGGAGATTCCACAGGCGAGGATCGATGCCCTGGAGGGTGTCTACAAGTCCCTCAATCTGTTTCTGGAGAATGGCAATTATCTGGCCGGCGACAACCTGACCATTGCCGATTTCCACGTCATCGCCGGCTTGACAGGATTTTTCGTGTTCCTGCCTGTAGATGCCACTAAGTATCCCGAGCTGGCTGCCTGGATCAAGCGCATCAAGGAGCTGCCATACTACGAGGAAGCGAATGGCTCTCGTGCTGCCCAAATCATCGAGTTCATCAAGAGCAAAAAGTTCACTATTGTTTAA

>CG6781Seomega

ATGAGTAACGGCAGGCATTTGGCAAAAGGCTCACCCATGCCGGATGTTCCCGAAGATGGTATCCTTCGCCTGTACTCGATGCGCTTCTGCCCATTTGCCCAACGGGTGCATCTGGTCCTGGACGCCAAGCAGATCCCGTATCACAGCATCTACATTAATCTCACAGACAAGCCGGAGTGGCTGCTGGAGAAGAATCCACAGGGCAAGGTGCCGGCTCTAGAAATCGTGCGAGAACCTGGACCACCTGTGCTCACAGAGTCGCTACTGATTTGTGAATATCTGGACGAGCAGTATCCATTGCGACCACTCTATCCACGTGATCCGCTGAAGAAAGTGCAGGACAAGTTACTAATCGAGCGATTTAGAGCGGTGTTAGGTGCCTTCTTCAAGGCATCCGATGGCGGTGATCTGGAGCCCTTCTGGAGCGGCCTGGACATCTACGAAAGGGAGCTGGCTCGACGTGGTACGGAATTCTTTGGTGGCGAGCAGACGGGCATTCTGGACTATATGATTTGGCCCTGGTGTGAGCGCCTCGAGCTCCTTAAGTTGCAGCGTGGAGAGGATTATAACTACGATCAGAGTCGCTTCCCCCAGCTGACCCTTTGGCTGGAACGCATGAAACGAGATCCGGCTGTGATGGCCTTCTACATGGAGGCCGAGGTTCAGGCGGAGTTCCTGCGTACACGGAGCCTGGGTCGACCCAATTACAATCTGCTGGTCAAGGATGCCTGA

>CG6776omega

ATGAGTTCTGGTAAACATTTGGCCAAAGGTTCCCCCAAGCCTGTACTCCCTGACGATGGAGTCCTTCGCCTGTACTCCATGAGGTTCTGTCCCTACGCTCAGCGGGCTCATCTCGTCCTGAACGCCAAGAATGTGCCCTATCACAGTGTCTACATCAATCTCACCGAGAAACCAGAGTGGCTGGTGGAAGTGAGTCCGTTGCTTAAGGTGCCTGCCCTGCAGCTGGTGGCGGAGAAGGGTGAGCCCTCGCTCATCGAGTCGCTGATTATTGCCGAGTATCTGGACGATAAGTACCCGGAGAATCCGTTGCTGCCCAAGGACCCATTGAAGCGGGCACAGGACAAGATTCTGCTGGAACGTTTCAGCAGCATCACCAGTGCCTTCATAAACATCCTTGTGCAGGGCACCGGTCTGGAGGATTACTGGACGGCACTTGATATCTTTGAGGAGGAGTTGACCAAGCGGGGCACCCCATATTTTGGCGGCAACAAGCCGGGATTCGTGGACTACATGATCTGGCCCTGGTTCGAACGCCTCTCTGTAATCGAGTTGAAGTTGCAGAAGGAGTACAACTTCAACGAGAGTCGTTTCCCGAAGATCACCAAGTGGATTGCCCTCCTGAAGGCGGACTCGGTGGTGCAGTCCTTCTATGCCACTCCCGAGCAGCACAACGAGTTCTGGCGCACCCGCAAGGCCGGCAATGCAAACTACGATCTGCTGGCTTAG

>CG4688Epsilon

ATGTCTCAGCCCAAGCCGATTTTGTATTACGACGAGCGCAGTCCGCCGGTCCGCAGTTGCCTTATGCTAATCAAATTGCTCGATATAGATGTGGAGCTCAGGTTTGTGAATCTCTTCAAGGGCGAGCAATTCCAAAAAGATTTCTTAGCGTTAAATCCCCAGCACAGTGTTCCCACCCTAGTCCACGGTGATCTGGTGCTGACGGACAGTCATGCTATACTCATTCACCTGGCGGAGAAGTTCGATGAGGGCGGTAGTTTGTGGCCGCAGGAGCACGCAGAACGGATGAAGGTTCTGAACCTCCTGCTCTTCGAGTGCTCCTTTTTGTTCCGACGTGACAGTGATTTTATGTCGGCGACTGTCCGCCAGGGATTCGCCAATGTCGATGTGGCACATCATGAACGCAAGCTGACCGAGGCGTATATTATCATGGAGCGTTACCTGGAAAATAGCGATTTTATGGCCGGGCCACAGCTGACGCTCGCCGACTTATCCATCGTGACCACATTGAGCACCGTCAATCTCATGTTTCCCCTGTCGCAGTTCCCACGTCTGCGGCGCTGGTTCACCGCGATGCAGCAGCTGGATGCCTACGAGGCCAACTGCAGTGGCTTGGAGAAGCTCCGCCAAACGATGGAGAGCGTCGGTAGCTTTCAGTTCCCATCGTCATCAGCGGTAGTCACCGAGAAGGTGGAGTAG

>CG4181D2

ATGGACTTTTACTACATGCCAGGTGGTGGAGGATGCCGCACGGTCATCATGGTGGCCAAGGCTCTCGGCCTGGAGCTGAACAAGAAGCTACTGAACACCATGGAGGGTGAACAATTGAAGCCGGAGTTTGTTAAGCTCAATCCACAGCACACCATTCCCACGCTGGTGGACAACGGATTCTCCATCTGGGAGTCCCGGGCCATCGCCGTCTATCTGGTGGAGAAGTACGGCAAGGATGACTATCTGTTGCCCAACGATCCCAAGAAGCGTGCCGTGATCAACCAGCGTCTGTACTTCGACATGGGAACTCTGTACGAAAGCTTTGCCAAATACTACTATCCCCTTTTCCGCACTGGAAAGCCCGGATCGGATGAGGACTTGAAGAGAATCGAAACCGCGTTTGGATTTCTCGACACCTTCCTGGAGGGCCAGGAGTATGTGGCTGGCGACCAGCTCACCGTGGCGGACATTGCCATCCTGTCCACTGTCTCCACGTTCGAAGTTAGTGAGTTCGACTTCAGCAAGTACTCCAATGTCTCCAGGTGGTACGACAATGCCAAGAAGGTGACTCCAGGATGGGATGAGAACTGGGAGGGCCTCATGGCGATGAAGGCGTTGTTCGATGCCCGTAAATTGGCGGCTAAGTGA

>CG17527E5

ATGGTCAAGTTAACTCTATATGGTGTGAATCCCAGTCCACCAGTTCGTGCCGTCAAACTCACTCTGGCTGCCCTCCAGCTGCCCTATGAGTTTGTAAACGTTAATATTTCGGGTCAGGAGCAGCTTTCTGAGGAATATCTAAAAAAGAATCCAGAGCACACGGTGCCAACACTGGAGGATGATGGTAACTACATTTGGGACTCGCATGCCATTATCGCGTATCTGGTGTCCAAATATGCAGATTCGGATGCCCTGTATCCCAGAGATCTGCTCCAGCGAGCTGTGGTGGATCAACGACTGCACTTCGAGACAGGAGTGGTTTTCGCCAATGGCATTAAGGCCATTACCAAGCCGTTATTCTTTAATGGCCTGAACAGGATTCCCAAGGAACGCTACGATGCCATTGTCGAGATCTATGACTTTGTGGAGACCTTCCTCGCTGGACATGATTACATTGCTGGGGATCAGTTGACCATTGCCGATTTTAGCCTGATCTCGTCGATTACCTCGCTGGTGGCGTTCGTGGAGATCGATAGGTTGAAATATCCCAGGATCATCGAATGGGTCAGGCGTTTGGAGAAGCTGCCTTACTACGAGGAGGCCAATGCGAAGGGAGCCCGGGAGTTGGAGACCATTCTAAAGTCCACTAATTTCACCTTTGCAACCTAG

>CG8938S1

ATGGCCGATGAAGCACAAGCACCACCAGCTGAGGGAGCACCACCAGCCGAGGGAGAGGCGCCACCACCCGCCGAGGGAGCCGAGGGTGCCGTCGAGGGCGGAGAGGCCGCTCCACCAGCAGAGCCCGCCGAGCCCATCAAGCACAGCTACACGCTCTTCTACTTCAACGTGAAGGCGTTGGCCGAGCCCCTGCGCTACCTGTTCGCCTACGGCAACCAGGAGTACGAGGATGTGCGCGTCACCCGCGACGAGTGGCCAGCCCTGAAGCCCACCATGCCCATGGGACAGATGCCCGTGCTGGAGGTGGACGGCAAGCGTGTGCACCAGAGCATTTCGATGGCTCGATTCCTGGCCAAGACTGTCGGCCTGTGCGGCGCTACTCCGTGGGAGGATCTGCAGATCGACATTGTCGTTGACACCATCAATGACTTCCGTCTAAAAATTGCAGTCGTCTCGTACGAGCCGGAGGACGAGATTAAAGAGAAGAAGTTGGTCACCCTGAATGCGGAGGTCATTCCATTCTACCTGGAGAAGCTCGAGCAGACCGTCAAGGACAACGATGGTCACCTGGCTCTGGGCAAGCTGACCTGGGCCGACGTCTACTTCGCAGGCATCACCGACTACATGAACTACATGGTCAAGCGCGATCTGTTGGAACCCTACCCAGCTCTGCGCGGAGTCGTGGATGCCGTCAACGCTTTGGAACCGATCAAGGCCTGGATCGAGAAGCGCCCCGTCACCGAGGTCTAA

>CG9362Z

ATGGCTTCTGCTACCCAATTGACCCACAGAGGTATCCATTTGGCCGGACTGTACAGATCTTCTTGGTCTAAGCCCTTGTTCCGACATTTGGCAACAAAGCCTATACTGTATTCCTATTGGCCTAGTTCGTGTTCTTGGAGAGTTCGCGTTGCGCTGGCAATAAAGAAGATTGATTATGACATTAAGCCGACTTCTTTGCTAAAAACAGTTAGCGGACATGCCTATACGGATGAATATAGGGAAGTCAATCCGATGCAAAAGGTTCCCTCGTTGAAGATTGATGGCCATACCTTGTGCGACTCGGTGGCTATTATCCACTACCTGGAGGAAACTCGACCTCAGCCAGCACTTCTGCCCCAAGATCCGGTTAAAAGAGCAAAGATCCGTGAAATTGTGGAGCTCATTTGCTCGGGCATCCAGCCACTGCAAAATGTATCTGTCCTAGATCATATAGGCAAGGATCAGAGTCTGCAATGGGCACAACACTGGATTTCTCGAGGATTTCAGGGTCTCGAAAAGGTTCTTTCCCATTCGGCGGGCAAATTCTGTGTGGGAGATGAGCTCAGCATGGCTGATATTTGCCTGGTACCTCAGGTGCGCAATGCCAGGAGATACAAAGCTGACCTGACCCCATATCCCACCATAGTACGCTTGAATCAGGAACTACAAGAACTTGATGTCTTCAAAGCCACTCATCCCAGTACCCAGCCAGATTGTCCACCTGAATTCGCAAAAAAATAA

>CG11512D4

ATGGATTTCTACTACTCCCCTCGAAGCAGTGGATCCCGCACCATTATCATGGTTGCCAAAGCTCTTGGACTGGAACTGAACAAAAAGCAACTGCGTATCACAGAAGGGGAACACCTCAAGCCAGAATTCCTTAAGCTCAATCCCCAGCACACCATTCCCACGCTGGTGGACAATGGATTCGCCATTTGGGAGTCCCGTGCCATAGCCGTCTATCTGGTGGAAAAGTACGGCAAGGACGACTCCCTTTTTCCCAATGATCCCCAGAAACGCGCATTGATCAATCAGCGATTGTACTTCGATATGGGAACCCTGCATGACTCCTTTATGAAGTACTATTACCCATTCATCCGTACTGGTCAGCTTGGGAATGCCGAGAATTATAAGAAGGTCGAAGCTGCCTTTGAATTCCTGGACATTTTCTTGGAGGGCCAGGACTACGTGGCTGGTAGCCAGCTAACTGTGGCCGACATCGCCATCCTCTCCAGCGTTTCCACTTTCGAAGTGGTTGAGTTTGACATCAGCAAGTATCCAAATGTGGCACGTTGGTACGCCAATGCCAAAAAGATCACACCCGGATGGGATGAAAACTGGAAGGGTCTGCTACAAATGAAAACAATGTACGAAGCCCAAAAGGCCTCATTAAAGTAA

>CG10045D1

ATGGTTGACTTCTACTACCTGCCCGGCTCCTCCCCCTGCCGCTCCGTGATCATGACCGCCAAGGCCGTGGGCGTCGAGCTGAACAAGAAGCTGCTCAACCTGCAGGCCGGTGAGCACCTGAAGCCGGAGTTCCTGAAGATCAATCCCCAGCACACCATTCCCACGCTGGTGGACAACGGATTCGCGCTGTGGGAGTCCCGCGCCATCCAGGTGTATTTGGTGGAGAAGTACGGCAAGACCGACTCCCTGTACCCTAAGTGCCCCAAGAAGCGCGCCGTGATCAATCAGCGCCTGTACTTCGACATGGGAACGCTGTACCAGAGCTTCGCCAACTACTACTACCCACAGGTGTTCGCCAAGGCGCCCGCCGATCCAGAGGCCTTCAAGAAGATCGAGGCCGCCTTCGAGTTCCTGAACACCTTCCTGGAGGGACAGGACTACGCCGCCGGTGACTCCCTTACCGTAGCCGACATTGCCCTGGTGGCAACCGTGTCCACATTCGAGGTGGCCAAATTCGAGATCAGCAAGTACGCCAATGTGAACAGGTGGTACGAGAACGCCAAGAAGGTGACTCCCGGATGGGAGGAGAACTGGGCCGGATGCCTGGAGTTCAAGAAGTACTTCGAATAA

>CG5164E1

ATGTCGAGCTCTGGAATTGTACTCTATGGGACAGATCTCAGTCCCTGTGTGAGGACCGTCAAACTTACCCTAAAGGTCCTGAATCTGGACTACGAGTACAAGGAGGTGAATCTTCAGGCGGGCGAGCACCTGAGCGAGGAATATGTGAAGAAGAATCCCCAACACACGGTACCGATGCTCGATGATAATGGCACTTTCATCTGGGACTCCCATGCCATTGCCGCCTACTTGGTGGACAAGTATGCCAAGTCGGATGAGCTGTATCCCAAGGATCTGGCCAAGCGTGCGATCGTCAATCAGCGTCTCTTCTTCGATGCCAGTGTAATCTATGCCAGTATAGCCAATGTCAGCCGCCCGTTTTGGATAAACGGTGTTACCGAAGTGCCCCAGGAAAAACTGGACGCCGTACACCAGGGTCTGAAGCTGCTGGAGACGTTCCTGGGCAACAGCCCCTACCTGGCCGGCGATTCGCTAACCCTAGCCGATCTGTCCACCGGACCCACTGTAAGCGCCGTGCCCGCTGCCGTGGACATAGATCCTGCTACATATCCCAAGGTCACCGCCTGGTTGGATCGCCTCAATAAGCTGCCCTACTACAAGGAGATCAACGAAGCTCCGGCCCAGAGCTACGTCGCCTTCCTGCGCAGCAAGTGGACCAAGCTGGGCGACAAGTGA

>CG30000Theta

ATGTCGAAGGCTATTAAGTATTATTACGATTTCCTATCGCAGCCATCCCGCGCCCTCTGGATTGCTATGAAATTGGGCAAGACCCCATTTGAAGACTGCCCGGTTGCCCTGCGAAAACAGGAGCAATTGACCGACGAATATCGCAGTATAAATCGGTTCCAAAAGGTGCCGGCCATCGTGGATGGTAAATTTCAGTTGGGCGAGAGTGTCTCCATAGTGCGATATCTCGCGGACAAGGGAGTCTTCAGTGAACAGCTGTACCCCAAAACCTTGGAGGAACGCGCGCGGGTGGACGAGTTCCTCGAGTGGCAGCATTTCAATGTCCGCTTGGTCTGCTCCCTGTTCTTCCGTCAGGTGTGGCTTCTTCCGGCAAAGGGACTGGCGCCGGCTCCCAAGCCAGAGTCTGTCAAGAAGCTAATCAAGGATGTGGAGAGTAATTTGGGTCTACTGGAACGCCTCTGGCTGGAAAAGGACTTCTTAGTCGGCGACAAACTTACCGTGGCAGACATCTTCGGGTCTTCAGAAATTAATCAGATGAAGCTCTGCCAGTACAACGTAAATGAGAAGCAATTCCCCAAGGTGGCCAAGTGGATGGAACGCGTTCGTGATGCTACCAATCCCTACTACGATGAGGCCCACAGCTTCGTCTACAAGACCTCTCAGCAGGCAGTCAAGGCCAAAAACTGA

>CG6673omega

ATGGCCCTGCCGCAAAAGCACTTCAAGAGGGGTTCCACCAAGCCCGAGCTGCCAGAAGATGGAGTTCCTCGTTTCTTTTCCATGGCATTCTGCCCCTTCAGCCACCGAGTTCGTCTGATGCTGGCCGCCAAACATATTGAACATCATAAGATCTACGTCGATTTGATTGAGAAGCCTGAATGGTACAAGGATTTCAGTCCTCTGGGCAAAGTGCCAGCCCTGCAGCTTACTGGGGTGAAGGACCAACCGACCCTCGTGGAGTCGCTCATCATAGCAGAGTACTTGGACCAGCAATATCCCCAGACGCGACTCTTTCCCACGGATCCGCTTCAGAAGGCGCTGGACAAGATTCTAATCGAACGTTTTGCTCCAGTGGTCAGTGCCATTTATCCTGTGCTGACTTGTAATCCCAACGCTCCGAAAGATGCCATACCGAACTTCGAGAATGCCTTGGATGTTTTTGAAGTGGAGTTGGGCAAAAGAGGAACGCCCTACTTTGCCGGCCAGCACATTGGAATCGTGGACTACATGATTTGGCCTTGGTTCGAACGTTTTCCCAGCATGAAGATCAATACGGAGCAAAAGTATGAGCTGGATACAAAACGATTTGAGAAGCTGCTCAAGTGGCGCGATTTGATGACCCAGGATGAGGTTGTGCAGAAGACAGCTTTGGATGTCCAGTTGCATGCTGAGTTCCAGAAATCGAAGACCCTTGGAAATCCCCAGTACGACATTGCCTTCAAGGGTACACCATAG

>CG18548D10

ATGGATTTATACTATAGACCCGGATCTGCTCCCTGCCGCTCTGTTCTGATGACAGCCAAGGCACTGGGTGTGGAGTTCGATAAGAAGACCATTATCAACACCCGAGCTAGGGAGCAATTCACGCCGGAATACCTGAAAATCAATCCGCAGCACACGATCCCCACGCTGCACGACCATGGATTTGCTTTGTGGGAGTCGCGGGCGATTATGGTTTATCTGGTGGAGAAGTACGGCAAGGACGACAAGCTCTTCCCCAAGGATGTGCAAAAGCAGGCGTTGATCAATCAGCGCCTGTACTTCGACATGGGTACGCTGTATAAGAGCTTCTCCGAGTACTATTATCCGCAGATTTTCCTAAAGAAGCCCGCCAATGAGGAGAACTACAAGAAGATCGAAGTGGCCTTCGAATTCCTAAACACATTCCTGGAGGGGCAGACCTACAGCGCTGGAGGGGATTATAGCTTGGCGGATATTGCCTTTCTGGCCACCGTTTCCACTTTCGATGTGGCTGGCTTCGATTTCAAGCGGTATGCCAATGTGGCACGTTGGTACGAGAATGCCAAGAAACTGACTCCCGGTTGGGAGGAAAACTGGGCTGGTTGCCAGGAGTTCCGCAAATACTTCGATAACTGA

>CG17522E10

ATGGCTAACCTGATTCTATATGGCACTGAGTCCAGTCCGCCGGTCCGTGCCGTTTTGTTGACCCTTCGTGCCCTCCAGCTGGACCATGAATTCCATACGCTGGACATGCAGGCCGGCGATCATCTGAAGCCGGATATGTTACGCAAGAATCCCCAGCACACGGTACCCATGCTGGAGGATGGTGAGTCATGCATTTGGGACTCACACGCCATCATCGGCTATCTGGTGAACAAGTACGCCCAGTCGGATGAGCTCTATCCAAAGGATCCGCTGAAGCGAGCTGTGGTGGATCAGCGGCTGCATTTCGAGACCGGTGTCCTTTTTCACGGCATCTTCAAGCAATTGCAGAGAGCTCTGTTCAAAGAGAATGCCACTGAGGTGCCCAAGGATCGTTTGGCTGAGTTGAAGGATGCCTACGCCTTGCTGGAGCAATTTCTGGCGGAGAATCCCTATGTGGCCGGTCCTCAGCTGACCATCGCCGATTTTAGCATTGTGGCCACCGTGAGCACTCTGCACCTGAGCTACTGTCCCGTGGATGCGACCAAATACCCCAAATTATCCGCCTGGTTGGCACGTATCTCCGCATTGCCCTTCTATGAGGAGGACAACTTGAGAGGAGCCCGCTTGTTGGCCGATAAGATTCGCTCCAAGCTGCCCAAGCAGTTTGACAAGCTGTGGCAAAAGGCCTTTGAGGACATCAAGAGCGGAGCTGGAAAACAGTGA

>CG4371D7

ATGCCGAACTTGGATCTCTACAATTTCCCCATGGCGCCGGCCAGTCGCGCCATCCAGATGGTGGCCAAGGCTTTGGGTCTGGAGCTGAACTCCAAGTTGATCAACACGATGGAGGGTGACCAACTGAAGCCAGAGTTCGTGAGGATTAACCCACAGCACACCATTCCCACGCTGGTGGACAATGGATTTGTCATCTGGGAGTCGCGTGCCATCGCCGTCTATCTGGTGGAGAAGTACGGCAAACCCGATTCCCCACTCTATCCCAACGATCCCCAGAAGCGGGCTTTGATCAACCAGAGGCTTTACTTCGATATGGGCACCCTGTACGACGCCCTGACCAAATACTTCTTCCTAATCTTCCGCACTGGCAAATTCGGAGATCAGGAAGCTCTGGACAAGGTTAACTCCGCCTTTGGATTCCTCAACACCTTCCTGGAGGGTCAGGACTTCGTGGCCGGTAGCCAACTGACCGTGGCTGATATCGTCATCCTGGCCACCGTATCCACCGTAGAATGGTTTTCGTTTGACCTAAGCAAGTTCCCCAACGTGGAGAGGTGGCTTAAGAATGCCCCAAAAGTAACTCCTGGATGGGAGCAAAATCTTGAGAGTCTGCAGCAGGGAAAGAAGTTCCTGCAGGACCTTCAAGCGGCAAAGGAAAAGGAAGTAAAGGCCTAA

>CG9363Z

ATGTCTACTAATCTCTGTCCCAATGCTTCCTCCTCCGACATACAGCCAATACTCTACTCGTATTGGCGCAGCTCGTGCTCCTGGCGCGTGCGCATTGCGATGAACCTGAAGGAGATACCCTACGACATCAAGCCGATCAGCCTGATCAAATCCGGTGGCGAGCAGCACTGCAATGAGTACCGCGAGGTGAATCCAATGGAGCAGGTGCCCGCCCTACAGATTGATGGACACACCCTCATCGAATCGGTAGCCATAATGCACTACCTGGAGGAAACACGTCCCCAGCGACCACTCCTGCCACAGGACGTCCACAAGCGGGCCAAGGTGCGCGAAATAGTCGAGATCATTTGCTCTGGCATCCAGCCCCTGCAGAACCTCATCGTGCTCATCCATGTGGGCGAGGAGAAGAAGAAGGAGTGGGCCCAGCACTGGATTACACGAGGCTTCCGGGCGGTTGAGAAGGCGCTGTCCACTTCGGCCGGCAAATATTGCGTGGGCGATGAGATCTCCATGGCGGACTGCTGCCTCGTACCTCAGGTGTTCAATGCCCGAAGATTCCACGTCGACTTGCGACCGTATCCCATAATTCTGCGCATCGATCGCGAACTGGAGAGCAATCCGGCATTCCGGGCGGCCCATCCCTCCAATCAACCGGACTGTCCGCCGGAGCTGCCCAACAAATAG

>CG4421D8

ATGGACTTTTACTACCATCCTTGCTCGGCTCCTTGCCGCTCCGTTATAATGACAGCCAAGGCCCTTGGAGTTGACCTGAATATGAAGCTATTGAAGGTCATGGACGGGGAGCAACTGAAGCCGGAGTTTGTGAAGCTCAATCCACAGCACTGCATTCCCACCCTGGTGGACGATGGCTTCTCCATCTGGGAATCCCGTGCCATTTTGATTTACTTGGTGGAGAAGTACGGCGCTGATGACTCGCTGTATCCCAGCGATCCCCAGAAGAAGGCTGTGGTCAATCAGAGGCTCTACTTCGACATGGGCACCCTGTTTCAGAGTTTCGTCGAGGCCATCTATCCACAGATAAGGAATAATCATCCCGCCGATCCAGAGGCCATGCAGAAAGTGGACAGCGCCTTTGGCCACCTGGACACCTTCCTGGAGGACCAGGAGTATGTGGCTGGCGACTGCCTCACCATTGCCGACATTGCCCTGTTGGCCTCCGTTTCCACCTTTGAGGTGGTGGACTTCGATATAGCCCAGTATCCAAATGTGGCCAGGTGGTACGAGAATGCCAAGGAAGTGACTCCCGGTTGGGAAGAGAACTGGGACGGTGTACAGCTAATCAAGAAACTTGTCCAGGAGAGGAATGAATGA

>CG6662omega

ATGAGCAATACTCAGCACTTAACTATTGGCTCGCCAAAGCCCGTATTTCCGGATGATGGGATCTTAAAGCTGTATTCGATGCGCTTTTGCCCCTATGCACACCGTGTGCACCTGGTCCTGGATGCCAAAAAGATTCCCTACCACGCTATCTACATCAATCTTCGCGACAAACCCGAGTGGTTCTCCCTGGTGAGCAGCTCCACAAAGGTGCCGGCACTGGAGCTGGTCAAGGAACAGGGAAATCCTGTGCTGATCGAGTCGCTCATTATTTGTGACTACTTGGACGAAAAGTATCCGGAGGTGCCATTGTATCCCAAGGATCTGCTTAAAAAAGCCCAGGAGAAGATTTTAATCGAACGTTTCGGACAGTTCATCAATGCCTTCTACTACCTGTTGCTGCACGACAATCCCGAGCAGCTGGTTGACACCGATCACTATGCCGGATTGGTCGTTTATGAGGAGGAACTGAAGCGACGTTGTACCAAGTTCTTTGGTGGCGACAGCCCAGGCATGCTTGACTACATGATGTGGCCCTGGTGCGAGCGCTTTGACTCTCTGAAATACACTTTTGAACAAAAATTCGAATTGAGTCCGGAACGTTTTCCCACTTTGATTAAGTGGCGCGACTTGATGATCCAGGATCGTGCTGTCAAGTGTTTCTATCTGGATGGACAGACCCATGCCAAATACATGAACTCCCGGCGATCGGGCCAGGCCGATTATAATATGCTATACAATGAGGCCAAACGTGTCAAATTGGGGTAG

>CG1681Theta

ATGTCGCAGCCACTCAAGTTCTACTTCGATTTCCTGAACCAATCGAGCCGGGCGCTCTACATCCTCCTGGAGGCCTCCAAGATCCCCTTCGAGGCCATACCCATATCGATGCTCAAAGGCGAGCACCTGACTGGCGAGTTCCGGGACAATGTGAACCGGTTCCGCAAGCTGCCGGCGATCACGGATCACGGCTACCAGCTGTCCGAGAATGTGGCCATCTTCCGGCATCTGGCACGCGAAAAGTTGGTGCCGGAGCACTGGTATCCGCGACGACATCTCGGCCGTAGTCGCATCGATGAGTACCTGGCCTGGCAGCAGACCAACATGGGTGTGGCCACCACGGAGTATTTCCAGCAAAAGTGGCTGGTGCCGTATCTGCAAAAGACCCGGCCAGCCGATAATGCGGTGAATCTCGCCAGCAAGCAGCTGGAGCACACGCTCAACGAGTTCGAACAGCTATTCCTCAACTCCCGCAAGTTCATGATGGGCGACAACATTTCGTATGCGGATCTCAGCGCCATCTGCGAAATCGATCAGCCGAAATCCATTGGCTACAATGCGTTCCAGAATCGCAACAAGCTGGCGCGTTGGTATGAAACGGTGCGCGAGGAGCTGGGTCCCCACTACAAGGAGGTGCTGGGCGAGTTCGAGGCCAAGCTGAAGGGCAGTGGCAGTGGTCAGCAGCAGGGCGTCGCCCAGGCGGTGAAGCAATAG

>CG17531E7

ATGCCCAAATTGATACTGTACGGCTTGGAGGCAAGTCCACCAGTTCGTGCCGTCAAATTGACCTTGGCTGCCCTGGAGGTTCCCTACGAATTCGTGGAGGTAAACACTCGGGCCAAGGAAAACTTCTCTGAGGAGTTTCTGAAGAAGAATCCACAGCACACGGTGCCCACGTTGGAGGACGATGGACATTATATCTGGGACTCACATGCCATTATTGCCTATCTGGTGTCCAAATACGGCAAAACGGACAGTCTCTATCCGAAAGATCTCCTCCAGCGTGCTGTCGTGGATCAGCGATTGCATTTCGAGTCCGGAGTGATCTTCGCTAATGCACTGAGAAGCATTACCAAGCCACTTTTCGCCGGTAAGCAAACGATGATTCCCAAGGAGCGTTACGATGCGATTATTGAGGTCTATGACTTCCTGGAGAAATTCCTTGCTGGAAATGACTACGTCGCCGGCAATCAGCTTACGATTGCCGACTTTAGTATCATATCAACTGTGTCCTCGTTGGAGGTCTTCGTAAAGGTGGACACGACCAAATATCCTCGGATAGCTGCATGGTTCAAGAGACTCCAAAAGCTGCCCTACTACGAGGAGGCCAACGGCAATGGTGCTCGTACATTTGAGTCCTTCATCAGAGAGTATAATTTCACTTTCGCATCGAATTAA

>CG30005Theta

ATGTCCAAGCCAATTAGATTCTATTACGATTTGTTGTCCCCGATCGCCCGTGGTCTATGGATTGGTTTAAAATTCAGCAACTCACCCGTTGAGTATTGCCCGATTGCCCTGCGAAAATTTGAGCAATTGACCGACGAGTACAAGAAGATCAATCGATTCCAGAAGGTGCCCGCCATTGTGGGTGGGGACTTCCATTTGTCTGAGACCATAGCGATTATAAGATATCTCGCAGACAAAGGTCAGTTCGATGAGAAGCTCTACCCAAAGACGCTGGAGAACCGTGCCCGAGTGGATGAGTTCCTTGAGTGGCAACACCTGAACATTCGTCTTGCATGTTCCATGTACTTTCGAGATGCCTGGCTGTTTCCCATGAACGGGATAGCGCCCAAGCCCAAGCCAGAGCAGATTCAAGCACTGATTGAGGGAGTGGAAAACAATCTGGGTTTACTGGAGCGCCTGTGGCTGGAAAATGATTTTTTGGTTGGGAAAAACCTGACAATGGCTGATATCCTTGGCTCTTCCGAAATCAACCAGCTGAGGCTCTGCCAATACAGAGTGGACGAGAAAAAGTTCCCAAAGGTGGTCAAATGGTTGGAACGCGTAAGGGTATCCGCAAATCCCTACCATGACGAGGGACTGACTTTTATTGACCGAAAATCTAAGCAATCGACGGCGGCAAAGTTGTAA

>CG16936Epsilon

ATGTCAAAGCCAGCTCTGTATTATGCCACCCTAAGTCCCCCATCGCGCGCCGTCCTCCTCACGGCTAAGGCGATCGGACTCGACCTTGAACTACGGCCAATTAACCTGCTGAAGGGAGAGCATCTGACTCCGGAATTCCTCAAGCTGAACCCCCAGCACACCATCCCGACCCTGATCGACGGCGAGGCCACTATCATTGACTCGCACGCCATCTGCGCCTACCTGGTGGAGAAGTATGGCCAGAAGGAGCAGCAGCTCTATCCGAAGGAATTGGTGCAGCGCGCCAACGTGGATGCTCGGCTCCATCTGGACTCCGGCCACCTCTTCGCGCGCCTGCGCTTCCTTTACGAGCCCATCCTGTATTATGGATCGACGGACTGCTCCATCGACAAGATCGCATACATCCAGAAGTGCTGGGAGATCCTAGAGGGATTCCTCAAGGATCAGCCGTATTTGTGTGGTTCTGATCTAACCATCGCAGACTTTTGCGCCGTGGCCACCGTAACCTCGGTGAACGACACCGCTCCCATCGATGAATTTAAGTTTCCCAAGATGCACGCCTGGCTGAAGCGTCTGGCAGAGCTACCCTACTACCAGGAGGTAAACGGCGACGGCGCTGACGAGCTTAAGAGCATCTTCAAGGCCAAGCTGGCAGAAAACCGTGGCAAGTAG

>CG17525E4

ATGGGTAAGATATCGCTATACGGCCTGGACGCAAGTCCGCCAACACGGGCATGTCTGCTCACCCTGAAGGCACTGGATCTGCCCTTCGAATTTGTGTTCGTCAATCTGTTCGAGAAGGAGAACTTTAGCGAGGACTTCTCGAAGAAGAATCCACAGCACACGGTGCCACTGCTGCAGGACGATGATGCCTGCATCTGGGACTCCCATGCCATCATGGCGTATCTGGTGGAAAAGTACGCGCCAAGCGATGAGCTCTATCCCAAGGATCTGCTGCAGCGTGCCAAAGTGGACCAATTGATGCACTTCGAGTCGGGTGTCATCTTCGAGTCTGCCCTAAGGAGACTCACCCGTCCGGTGCTCTTCTTCGGCGAGCCCACCTTGCCCCGCAATCAGGTGGATCACATCCTTCAGGTCTATGATTTCGTAGAGACCTTTCTCGATGATCACGACTTCGTGGCCGGCGATCAGTTGACCATAGCCGATTTCAGCATCGTATCGACCATCACCTCGATTGGTGTTTTCCTGGAGCTGGATCCGGCCAAGTACCCTAAGATTGCCGCCTGGCTGGAGAGGCTTAAGGAGCTGCCCTACTACGAGGAGGCCAATGGCAAAGGAGCTGCCCAGTTCGTGGAGCTCTTGAGGTCCAAAAACTTCACCATAGTTTCGTAA

>CG1702Theta

ATGTCCGCTCCCATTCGCTACTACTATGACCTGATGTCGCAGCCCTCGAGGGCGTTGTTCATTATCTTCCGGCTGAGCAACATGCCCTTCGAAGACTGCGTGGTTGCCCTGCGCAATGGCGAGCACTTGACCGAAGACTTCAAGAAGGAGATTAACCGCTTCCAGCGTGTGCCCTGCATCCACGACAATGGCTACAAGCTAGCGGAGAGCGTGGCCATCCTGCGCTATTTGAGCGCCAAGGGCAAGATACCGGAGCACCTCTATCCCAAGTACTTCGTTGACCAGAGCCGCGTCGACGAGTTCCTCGAATGGCAGCACATGTCCCTGCGGCTCACCTGCGCCATGTACTTCCGCACCGTGTGGCTGGAGCCGCTTCTGACAGGACGCACACCCTCCGAAGCCAAAATCGAGACGTTCCGCATGCAGATGGAGCGCAACCTCGACGTAGTCGAGGAGGTCTGGCTGGAGGGCAAGGACTTCCTCACCGGATCCTCCCTCACCGTTGCGGACATCTTTGCAGCCTGTGAAATCGAACAGACACGAATGGCCGACTACGATGTCAGGATCAAGTACCCCAAGATCAGGGCGTGGCTGAAGAGAGTGCGCCAGAGCTGCAATCCGTACTACGATGTGGCCCACGAGTTCGTCTACAAAATCTCCGGAACGGGTCCACAGGCCAAGCTATAA

>CG17639Delta

ATGTCCGACACGAAGCGTATTGTAACTGAATTCGTGTATTGCCTACTCCTCAGAGGTGGCAAGATGTCGCCGCCCGTGCTGTACTACCTGCCGCCCAGTCCGCCCTGCCGCAGCATTCTGCTGCTGGCCAAGATGCTGGACATCGACTTCGAGCTGAAGATCGTCAACATTTTGGAGGGGGAGCAGCTGAAACCGGACTTTGTGGCCATGAATCCGCAGCACTGTGTGCCGACTATGAACGACGAGGGTCTGGTTTTGTGGGAAAGTCGTGCCATACTCTCCTATCTGGTGGCTGCCTACGGCAAGAGTGACCAACTGTATCCCACGGACATAAGGGTGAGGGCTTTGGTGGACCAACGCCTCCAGTTCGATTTGGGCACCCTATACATGCGACTCACGGACTACTATTTCCCCACAATGTTTATTGGTGCGCCCCTGGACGAAGGAAAGCGTGCCAAATTGGCGGAGGCTGTGGGCTGGCTAAACACAATCTTGGAGGGCAGACAGTTTTCCGCCGCCGATCACTTCACCATCGCGGATCTCACGCTTTTGGTGACCGTTTCCCAGCTGGAGGCCTTCGAATTTGAACTTCGACCCTATAAACATATCCGCCAATGGCTCGATCGCTGCAAGGATCACATGGCACCGTTTGACTACGAGGAACTCAATGCCAACAAGGCCAATATGTTGGCCGATATGTTCAAGGCCAAGATGAATCAATCGGCGGGCTAA

>CG10091D9

ATGTTGGACTTCTACTATATGCTCTACTCGGCACCTTGCCGTTCCATCCTGATGACGGCCCGTGCCCTGGGATTGGAGCTGAACAAGAAGCAGGTGGATCTGGATGCCGGCGAGCATCTTAAGCCGGAATTTGTAAAGATCAATCCTCAGCATACGATTCCCACGCTGGTTGACGATGGTTTCGCCATCTGGGAGTCGAGGGCTATACTGATTTATCTGGCCGAGAAGTACGATAAAGATGGCTCCCTTTATCCCAAGGATCCCCAGCAGAGAGCCGTGATCAATCAGCGCCTGTTTTTCGATCTGAGTACTCTGTACCAGAGCTACGTGTACTACTACTATCCCCAGTTGTTCGAGGATGTGAAGAAGCCAGCTGATCCCGATAACCTCAAGAAGATCGATGATGCTTTCGCTATGTTCAATACTCTGTTGAAGGGTCAGCAGTACGCCGCCCTCAACAAGTTGACTCTGGCCGATTTTGCGCTTCTGGCCACCGTTTCCACTTTTGAAATATCGGAATATGATTTTGGTAAATATCCGGAAGTGGTTAGGTGGTACGACAATGCCAAGAAAGTGATACCCGGCTGGGAGGAGAACTGGGAGGGCTGCGAGTACTACAAGAAATTGTATCTGGGTGCGATTTTGAACAAACAATGA

>CG4381D3

ATGGTGGGCAAGGCCCTGGGTCTAGAATTTAATAAGAAGATAATCAATACGCTGAAAGGGGAGCAAATGAATCCCGATTTCATCAAGATCAACCCGCAGCACTCGATTCCCACGCTGGTAGACAATGGTTTCACCATTTGGGAGTCGCGTGCCATTCTCGTTTACCTGGTGGAAAAGTACGGAAAGGATGACGCGCTGTACCCCAAGGATATTCAGAAGCAGGCGGTGATCAATCAACGCCTTTACTTCGACATGGCGTTGATGTATCCCACCCTGGCCAACTACTACTACAAAGCATTTACCACCGGTCAGTTTGGCAGCGAGGAGGACTACAAAAAGGTCCAGGAGACTTTCGATTTCCTAAACACATTCCTGGAGGGTCAGGACTACGTGGCTGGGGACCAGTATACCGTCGCCGACATTGCCATTCTCGCCAATGTCTCCAATTTCGATGTTGTGGGATTCGACATTAGCAAATATCCGAATGTGGCCCGATGGTACGACCATGTCAAGAAGATTACCCCTGGATGGGAAGAAAACTGGGCAGGAGCTCTGGATGTAAAGAAGAGGATCGAGGAGAAACAGAATGCTGCTAAATAA

>CG33546gfzfDE

ATGAAACTCTACGCCGTATCCGATGGTCCGCCTTCCCTGGCCGTTCGCATGACCCTGAAGGCCTTGGATATACAATATCAACTCATCAATGTGGACTTTTGCGCCATGGAACATCGCTCTGAGGAGTACTCGAAGATGAATCCGCAAAAGGAGATACCCGTGCTGGACGACGACGGTTTCTATCTATCGGAGAGCATTGCCATTATGCAATACCTCTGCGACAAGTACGCGCCGGATTCAACGCTATATCCGCAGGACGTCAATGTGAGAGCAGTAATCAATCAGCGGCTATGCTTTAACATGGGATTCTACTATGCCCCCATATCCGCCCACAGCATGGCGCCCATTTTCTTCGACTACAAACGTACGCCCATGTCGCTAAAGAAGGTGCAGAACGCACTTGATGTGTTCGAAACCTATTTGCAGCGACTGGGCACTAAGTACGCGGCAGGCGAGAACATAACCATTGCAGACTTCGCTCTCATTTCGGCCACGATCTGCTTGGAGGCAATTAACTTTGACTTGCACCAGTTTACGTTGGTGAACAAGTGGTACGAAACCTTCAAGGTGGAATACCCGCAGCTGTGGGAGATCGCCAACAGCGGCATGCAGGAGATAAGTGCGTTTGAGCAGAACCCACCGGATATGTCACACATGGAACACCCATTCCATCCGACGCGCAAGTCTATGGGCCTGAAGTTGTAG

>CG5224Epsilon

ATGTCGGCCAAACCCATCCTCTATTACGCTCCCCGTAGTCCCCCCTGTCGTGCTGTTCTGCTGACGGCCGCCGCCCTCGGTTTGGAGTTGGACTTGCGACTGGTCAACGTAAAGGCCGGAGAGCACAAATCCGCCGAGTTTCTCAAGTTGAATGCGCAGCACACGATCCCCGTGCTCGATGATAACGGCACCATCGTGAGCGATTCGCACATTATCTGCAGCTATCTGGCAGATAAGTACGCACCGGAGGGCGATGATTCCCTGTATCCAAAGGATCCGGAGAAGCGGCGCCTGGTGGATGCCCGTTTGTACTACGATTGCGGTCATCTATTCCCGCGAATCCGTTTCATTGTCGAGCCGGTGATCTATTTCGGAGCTGGCGAGGTGCCCAGCGATCGAGTGGCCTACCTTCAGAAGGCCTATGATGGCTTGGAGCACTGTCTGGCTGAAGGTGATTACTTGGTGGGCGACAAGCTGACCATCGCCGATCTCAGCTGCATCGCATCGGTGTCCACGGCCGAGGCTTTTGCGCCAATCGAGCCGGATCAGTTTCCACGCCTGGTACAGTGGGTCAAGCGCATTCAGGCCCTTCCATACTACCAGAAAAACAATCAGGAAGGTCTGGATATGTTGGTGGGACTGGTTAAGGGACTTTTGGCTGAGCGACAGCAGAAGTAA

>CG4423D6

ATGGATCTCTATAACATGTCTGGTTCCCCGAGCACCCGGGCCGTTATGATGACCGCCAAAGCTGTGGGAGTCGAGTTCAACTCGATACAAGTTAATACCTTTGTGGGGGAACAACTGGAGCCATGGTTTGTAAAGATAAACCCTCAGCACACGATTCCAACCCTGGTGGACAATTTATTCGTTATTTGGGAAACTCGGGCCATTGTTGTCTACCTAGTGGAGCAGTACGGTAAGGATGATTCTCTGTACCCGAAGGATCCCCAGAAGCAAGCGCTGATCAACCAGCGTCTGTACTTCGATATGGGCACCCTGTACGATGGCATTGCCAAGTACTTTTTCCCACTGCTTCGGACGGGCAAACCCGGAACTCAGGAGAATCTTGAGAAACTGAACGCTGCCTTCGATCTTCTCAACAACTTCCTGGATGGCCAGGATTACGTGGCCGGCAATCAGCTTTCAGTGGCAGATATCGTTATATTGGCCACCGTTTCCACAACCGAAATGGTTGACTTCGATTTAAAGAAGTTTCCCAACGTGGACAGATGGTATAAGAACGCCCAAAAAGTTACGCCGGGTTGGGACGAGAACTTGGCAAGAATTCAAAGCGCCAAAAAATTTCTAGCCGAAAACTTAATAGAAAAGTTATAA

>CG17533E8

ATGTCCAAGCTGATTTTGTACGGGACAGAGGCTAGTCCTCCAGTTCGGGCCGCCAAATTGACCTTGGCCGCCCTTGGCATTCCCTACGAATACGTCAAAATAAACACCCTGGCCAAGGAGACCCTTTCGCCCGAGTTCTTGCGGAAGAATCCCCAGCATACGGTGCCCACTTTAGAGGACGATGGCCATTTCATCTGGGACTCACACGCAATCAGCGCATATCTGGTGTCCAAGTATGGCCAGAGTGATACTCTTTATCCGAAAGATCTCCTCCAACGCGCTGTGGTGGACCAGCGATTGCACTTCGAGTCCGGCGTGGTCTTTGTCAACGGACTGAGAGGCATCACCAAGCCGCTCTTTGCGACCGGTCAGACGACGATTCCCAAGGAGCGTTACGATGCCGTCATAGAGATTTACGACTTTGTAGAGACTTTTCTCACCGGACACGATTTCATTGCCGGTGATCAGTTGACCATCGCGGATTTTAGCCTAATCACATCGATTACCGCACTGGCGGTATTCGTGGTCATCGATACAGTAAAATACGCCAATATAACTGCGTGGATCAAGAGGATTGAGGAACTACCCTATTACGAGGAGGCATGTGGCAAAGGTGCCCGGGATTTGGTGACCCTCCTCAAGAAATTCAATTTTACCTTCTCAACTTAA

>CG17530E6

ATGGTGAAATTGACTTTATACGGTTTGGACCCCAGTCCCCCAGTTCGCGCTGTTAAGCTTACTTTGGCCGCTCTAAACCTAACCTACGAATATGTAAACGTTGACATTGTGGCTCGTGCCCAACTTTCACCGGAATATCTGGAGAAGAATCCACAGCATACGGTGCCCACCCTGGAGGATGACGGTCACTACATCTGGGATTCGCATGCCATTATTGCCTATTTGGTCTCGAAATATGCCGATTCCGATGCCCTATACCCGAAAGATCCTCTCAAGCGGGCTGTTGTGGATCAGCGGCTGCACTTTGAATCCGGAGTGGTCTTTGCCAATGGCATAAGGAGCATATCGAAGTCAGTGCTCTTCCAGGGACAGACGAAAGTACCCAAGGAGCGATACGATGCCATTATCGAGATCTACGATTTTGTGGAAACTTTTCTCAAGGGACAGGATTACATTGCTGGCAATCAACTGACCATTGCGGATTTCAGTCTCGTTTCATCGGTGGCCTCCCTTGAGGCCTTCGTGGCCTTGGATACGACTAAGTATCCCAGGATCGGTGCTTGGATCAAAAAGCTGGAACAGCTTCCATACTACGAGGAAGCCAATGGCAAGGGCGTCCGCCAGTTGGTGGCCATTTTCAAGAAGACCAATTTCACATTCGAAGCATGA

>CG17534E9

ATGGGAAAATTAGTACTGTACGGCGTAGAGGCTAGTCCGCCGGTGCGAGCATGCAAACTGACCCTCGACGCCCTGGGCCTTCAGTATGAGTATAGGCTGGTGAACCTGCTGGCCGGTGAGCACAAGACGAAGGAGTTCAGCCTGAAGAATCCGCAGCACACGGTACCCGTGCTGGAGGACGATGGCAAGTTCATCTGGGAGAGTCACGCCATTTGCGCTTATCTGGTTAGACGCTATGCCAAGAGTGATGACCTGTATCCCAAGGATTACTTCAAACGCGCACTCGTTGATCAGCGCCTGCACTTTGAGTCGGGTGTGTTATTCCAGGGCTGCATCCGGAACATAGCCATTCCGTTGTTCTACAAGAACATAACTGAGGTGCCGCGTTCCCAGATTGATGCCATCTACGAGGCCTATGACTTTCTGGAAGCGTTCATCGGTAATCAGGCTTACCTCTGCGGACCGGTCATAACCATCGCCGACTACAGTGTAGTTTCCTCGGTCTCCAGCCTAGTGGGATTGGCCGCTATCGATGCCAAGCGCTATCCCAAGTTAAACGGCTGGCTAGACAGAATGGCCGCACAGCCCAATTACCAGTCGCTCAATGGCAATGGGGCACAGATGTTGATCGACATGTTCAGTTCGAAGATCACAAAGATTGTGTAA

>CG17523E2

ATGTCGGATAAATTGGTTTTGTATGGCATGGATATTAGTCCTCCTGTTCGCGCTTGCAAGCTGACCTTGCGGGCCTTAAACTTGGACTACGAATACAAGGAAATGGATCTACTGGCAGGAGATCACTTTAAGGATGCGTTCCTCAAAAAGAACCCGCAGCACACCGTACCACTCCTCGAAGATAATGGTGCCCTTATCTGGGATTCACATGCTATTGTCTGCTACCTGGTGGACAAGTATGCCAATTCGGATGAGCTATATCCCAGGGATCTGGTGTTGCGCGCCCAGGTGGATCAGCGTTTGTTCTTTGATGCCAGCATTCTGTTTATGTCGCTGCGAAATGTCAGTATACCCTATTTTCTTCGCCAAGTAAGCCTGGTACCCAAGGAGAAGGTGGACAACATTAAAGATGCATATGGCCATTTGGAGAACTTTCTAGGGGATAATCCCTATTTGACCGGGTCGCAACTGACCATAGCTGATTTATGCTGCGGAGCTACTGCATCCTCGCTGGCCGCTGTTCTTGATCTGGATGAGTTAAAGTATCCAAAGGTGGCTGCTTGGTTCGAACGACTCTCTAAGTTGCCCCACTATGAGGAAGACAATCTGCGGGGCTTGAAGAAGTATATCAATTTATTGAAACCCGTATTAAATCTGGAGCAATAG

>CG12242D5

ATGGATTTCTATTACTCGCCCCGTGGAAGTGGATGTCGCACCGTGATCATGGTGGCCAAGGCTCTCGGCGTGAAGCTGAACATGAAGCTACTGAACACCTTGGAGAAGGATCAGTTGAAGCCCGAGTTCGTCAAGCTCAATCCATAGCACACCATTCCCACGCTGGTGGACAACGGATTCTCCATCTGGGAGTCCCGCGCCATTGCCGTCTATCTGGTGGAGAAGTACGGCAAGGATGACACCCTCTTCCCCAAGGATCCCAAGAAGCAGGCTTTAGTCAACCAACGCCTCTACTTCGACATGGGAACTCTGTACGACAGCTTCGCCAAATACTACTATCCCCTTTTCCACACTGGAAAGCCCGGATCTGATGAGGACTTTAAGAAGATCGAGAGCTCCTTCGAGTATCTGAATATCTTCCTGGAGGGCCAGAACTACGTGGCCGGTGACCACCTCACAGTGGCTGATATTGCCATCCTCTCCACCGTTTCCACTTTCGAAATCTTTGATTTCGACCTCAACAAGTACCCGAATGTGGCCAGGTGGTATGCCAACGCCAAGAAGGTGACTCCCGGATGGGAAGAGAACTGGAAAGGTGCCGTGGAACTGAAGGGAGTATTTGATGCCCGTCAGGCGGCGGCGAAGCAATAA

>CG11784Epsilon

ATGTCGAAGCCAACGCTGTACTACGCCCTTTTTAGTCCTCCTGCCAGGGCATGCATCCTGGTGGCCAAACTTATTGGACTGGACCTGGAGCTCAAACCCGTTGACTTCGCCAAGAAGGAACACCTGAGCGAGGAATTTGTCAAGCTAAACCCCCAGCACCAGATCCCGGTGTTTGTGGACAGCGATGGCGAGGTCTACGTGGACAGTCACGCCATCGTGTGCTTCCTGGTAGCCAAGTACGCCGGGAATGACCAACTCTATCCGCGGGATTTGAAAAGGAGAGCCCACATCGACCATCGCATGCACTACGAGAACGGAGTGCTATTTCAGGTGGTAAAGGACATTGTGGCTCGGAACATTTACGGTGGCGAGGGAGAATACAACCCCCGATCGCTGACCCTCTGCCACAATGCGTACTCCGACTTGGAACACTTTCTGCAGCAAGGAAGTTTCGTGGTGGGCAACGAACTGAGCGTTGCCGACGTGTCCATCCACACCACTCTGGTGACCTTGGATCTGCTCATACCAGTGGAACGGGAAAAGTACCCGCAGACTAAGCAATGGATGGAACGCATGGATAAGTTGTTGCCCGACAACGAGGAGATCAACCTCAAGGGTGCACGGGCTCTGCAGACCCGCATCCTGAGCTGCATGGCCGAGAACAAAGCGAAGAGCCAGTAG

>TC004450Epsilon

ATGGCCAAGCTCTACTACAATGACATGAGCCCCCAGAGCAGGGCCGTGCTGATGACGGCGGGCGCCATCGGGGCGCGACTCCACCTCCAGGAGGTCCACCTCTACGGCAAGGACCCCGACCCGCAGCTCCTCCAGATAAACCCCCATCACACGGTGCCCACGTTGCTCGATGGTGACTTTACCATCTGGGACAGCCACGCGATTGTGGGCTATCTCGTCGGACAGCACTCCTTCGATGAGCTCTACCCCAAAGAGACGAAACCGAGAGCTCTAGTTGATCAGTTTTTACATTTCGATTCAGGCGTCTTGTTTCCTAGAGTCAATAGCATTATCGATTCCATCGTAGTGGAGAACGATAAAGAAATATCGACGAAAAGAATAAGTGCAATTACGGAATCTTACAAATTTTTAGATAAATTTCTACAAAACAAGAACTACTTAATTGGTCCAAACCTAACGGTGGCTGACTTATGTTGCGTTGCAACGATCAGTACAGCCACCATAATAACCCCCATATCAACGGAAAAGTACCCCCATCTTTCCACGTGGTACCGAACCTGCAAGAATCTTCCCTTCTACGAAGAAACAAACGGCGTGGGTCTCAACAAACTCGACGCTTTGGTCGAACTCAAGCTAGGACGACCTCGAACCAAAGACTTCTGTGAATAA

>TC004449Epsilon

ATGTCCCTAAAACTGTACTACACATCAGTAAGCCCCCCATGCCGGGCGTCCCTTTTGACAATTAACGCCTTGGGCATTGACGTCGAACTTGTCCCAATAAATCTAAGCGCACAAGAACATCTCACTACCGAGTTTCTTCAGCTTAATCCCTTTCACACCGTGCCGACGCTTCAAGATGGATCTTTCACACTTTGTGACAGTCATGCAATTAATGCGTATCTTGTCGAAAAGTACGCAACGGATGATTCTCTCTACCCCAAAGACTTGCAACAAAAGGCCATCGTCAGCCAAAGGCTGCACTTTGATTCAAACGTACTGAGCGCAAGGCTTTCGGCAATTACGGGCCCCATTTTGCGAGGCGGTGCCAAAACTGTGGCCAAAGACAAGGCCGACGCTCTGCTCCAGGGCTTAACCCTCCTGGAGACGATCCTCGAAACGAACAAATACGCCTGTGGCGATAAGTTAACCATTGCCGATTTCAGTCTTGTCACCACTGTCAGTAGTGCAAACGCAGTCCTTCCTTTAGCCTCCAACAGGTTTCCGAAAATTTTCGACTGGTGGAACCGACTTGAAGCTCTACCCTTCTACAAAGAAGCGAACCAGGAGGGTCTAGATGCGTTCACAAATCTCATTAAAAGCAAAAACATTGTGCGACAAAATTCGACAAGATAA

>TC004448Epsilon

ATGGCGCCCGTACTTTATGTCACACATCTGACGCCGCCAGTCCGAGCTGTTCTAATGACCGCCAAAACAATAGATCTAGATCTCGAACTCAAAAAATTAAATGTGGAAAAACGTGAACACAAAAACTCCGAATTTCTCAAGTTAAATCCTCAACACACCGTCCCCACTTTAGTCGACAATGATTTTGTCCTATGGGACAGTCACGCAATTATGGCTTATCTCGTCTCCAAATACGCCAAAGACGACTCTCTTTACCCGAACGACTTGAAACAGAGAGCCATCGTGAACCAAAGAATGCATTTTGAAAATGGGGTCGCATTTCCCGAGTTGTTAAAAATTTTGTATCCAATAATCCATGACGGGAAAAAAACAATCACACAAGAAGACGAAATCGCAGCTGATGAAGTTTATTCCTTCCTGGAGGCTTTTCTCGACGGAAAACAGTGGATAACGGGCGATTCTGTCACGATTGCTGACTATAGTTTAATAACTACAATCACAGCCTTGAATGTTTTGGTAAAAATTGACCATGTTGTGTTCCCAAACCTGAACACTTGGATGAAAAAACTCGAACAACTGCCGGTTTATGAGGCAAATAGGAAAGGACTGGACAGTTACACCACTCACGTTAAATTGTTGTTACGCTGA

>TC004447Epsilon

ATGGCGCCCACACTGTATATGATGCCTCCAAGTCCCCCAGTACGAGCTGTTCTCATAACTGCAAAATCAATCGGTCTTGACCTAGAGCTCAAACAATTAAACCTTCGAAGAGGTGACCACAAAACACCTGAATATCTTAAGCTAAACCCCCAACACACCGTCCCCACTTTGGTCGATGATGATGGTTTTGTCCTTTGGGACAGCCATGCAATTATGGTTTATCTCCTCTCAAAATACGCCAAAGACGATTCTCTGTACCCACAAGACTTGAGAAAGAGAGCGGTTATTGATCAAAGGATGCATTTTGACAGTGGTGTTGCTTTTGCCGTGTTCTTAAGAATTTTGTATCCACTAATCTATGGTGAGAAGAAAACAATCACTTCTGAAGATACGAAAGCAGCTGAGGAAGTTTATTCCTTCTTGGAAGCTTTTCTTGAAGGAAGGCAATGGTTAACTGGCGATTTTGTCACAATTGCTGATTACAGTTTGATTGCCACGACTAGTTCTCTTAACGTTTTGGTGAAAATTGATCCTGTTAAGTACCCAAAGCTAACCGCTTGGGTGAAAAATGTTGAAAAACTGCCGGCCTATGAGGCGAATAAGCCAGGACTTGAAAAATTCATTGGCTACGTTAAGTCAATATTAGGATAA

>TC004940Epsilon

ATGGCTATCACCTTGTACATGGTCGCTCCAAGCCCAGCGGTACGTGCCGTTCAAATCACAGCCAAGGCTCTAGGAATAGAGCTGAAGGAAAAACCTTTGAACTTTCTGGAAGGCGAACATCTAAAACCCGAATATCTCAAGATCAACCCCCAACACACTGTACCCACTATTGTCGAAGACGACGGGTTCACTCTTTGGGACAGCAACGCCATTAACGCCTACCTTGTCTCAAAATATGGCAAAAACGACTCCCTCTACCCCAAAGACCTGAAGAAGAGGGCACTTGTCGACCAAAGACTTCACTTCAATAACGGAGTTGCGTTCGCCACCGGTTTAAAAATTATCGGTGCTATTCTTCGCGCTGGCAAAACCACAATCGATGATAAAGACCAGGAAGACCTGAACCGAGTTTACGCATTTATTGAAGCTTTCCTGGAGGGCAAGCAGTGGATTGCTGGCGATTGTGTGACCATTGCTGACTACAACTTGTACGCCACTGTTAGTGCCATGAATGTTCTGGTACCGATTGATGGCAAGAAGTACCCGAAAGTGGCAGCTTGGTACAAGAAAGTCGATGCTCTGCCCGAAGTTGAAGTGAGCAAGAAAGGCTTGGGCATGTTCGAAGCCATGATTAAAGGCAAACTTAAGTAA

>TC004941Epsilon

ATGGCACCTACTTTGTACATGCTTCCTGCAAGTCCTCCTTGCCGAGCTGTGGTTATGACAGCTAAAGCCCTTGGCGTGGAATTTGTAGAAAAAGGTATTTACTTCTTTCGAGACGATAATGTGAAGAAGGAATTTTGCAAAATAAACCCACAACACACTATTCCAACCCTTGTTGATGAAGACGGATCGGTATTTTGGGACAGTCATGCAATTATGGCTTATCTGGTAGCCAAATATGCAAAAAATGACTCGCTCTATCCTCAAGATATTAAAAAACGAGCCATTGTTAACCAACGATTGTTTTTCGAATCTAGTGTTATATTTTTTCATATGAAAAATATTGCTTGTTCAATTCTTCTAGACGGCAAAAATTTTATAGAGTCGGAAGAGAAGGAACCTCTACTTGAAAGTTTAAAAGTTTTGGAAAAATTCCTAGAAGACAGTGAATGGATGGCTGGAAATTCACTTACCATAGCTGATTATAGCCTAGTTTCGTCTATCGCTAGTGTTAATAACGTGATACCAATTGATCCCGAAAAGTACCCAAAGTTAATTGCTTGGTTTCAAAAATGTAAGGAGCTTCCTGAATATGAAGTCACGAGGAAAGGGCAAGAAGAAGGTGCTGCTATGCTTAACGCTAGATTAAATATGTCATTCGGGAAAAAATACCAATAA

>TC004942Epsilon

ATGGCACCTACACTGCACATGCTTTATGCAAGTCCTCCGGCAAGAGCTGTCATGATGACGGCCAAAGCCATCGGACTTGAACTCAATCTGAAAGAAGTTGATTTCATGAACGAGGAACATTTAAAACCCGAATATGTGAAAATGAACCCTCAACACACAATTCCAACACTTGTCGATGACGATGGTTTCATAATTTGGGACAGTCATGCCATCATGATCTACCTGGTCTCCAAATACGCCAAAGACGACGCACTTTACCCCAAAGATATCAAAAAACGGGCAGTTATTGACCAAAGACTCCACTTTGAATCGGGCGTCGTATTCGCACTTTTGAGAAGAATTGCGAGACCAATTGTTATCGGAGGTCAGGATTTTATTGAAGAGAAAAATCAAAAAGGTGTTATTGAAAGTTACGCTTTCTTGGACCAGTTTCTGGACGGGAGAAAATGGGTTGCTGGTGATTTTAAAAGCATTGCAGATTATAGTCTGCTTTCATCGATCAGTACACTAAACAAGGTGATTCCTGTCGATCCTGAAAAGTATCCAAGAGTCATCGCTTGGCTGAAAAAATGCGAGGAACTACCGGAATACGAGGCCAACCGCAAAGGAGTGCAAGAGATGGCTGACATGATCAACAGCAAGTTGGCTAAAAAATGA

>TC004446Epsilon

ATGGCTCCGACTTTATACATGATTCAAGCGAGCCCTCCAGTGCGAGCAGTTTTGATTACGGCCAAAGCCATCGGGCTTGATTTGAACCAAAAAGACATCAATTTTTTTCAAGACGAACACTTGAAACCACATTTTCTTAAATTGAACCCTCAACACACTATCCCCACGTTGGAAGATGAGGACGGTTTCGCAGTTTGGGACAGTCATGCAATTATGACTTATCTCGTTTCAAAATATGCAAAGGACGATTCTGTTTATCCACAAGATATTAAAAAGCGAGCGGTTGTAAACCAGCGACTGTTTTTTGAATCCGGAGTAATTTTTTTCCACATGAGGAATATTGCTCGATCGCTTCTTGTTCATTGCCAAAACTTTATTAACGAGGATGACAAGGATGGCATGATTGAGGGTTTTGGTTTATTGGAAAAGCTTCTAGAAGGGAAAAAATGGGCCGCTGGTGATTTTGTCAGTCTTGCTGACTATAGTTTAATTTCGTCAGTTGGTACCGCTGTAACTATTATACCAGTTGACTCGGAGGAATACCCTAATTTGACTGCTTGGATGAAAAGGTGTGAGGAATTGCCAGAGTATGCAGCCAATGCCAAAGGCTTGAAAGAAGCCGCCGAAATGCTTAAACTTAGACTAACCAGAAAGTGA

>TC004445Epsilon

ATGGCCCCAACCCTGTACATGTTCCAAGCAAGCTCTAATGTACGAGCTGTCCTTATTACAGCCAAAGCTATTGATTTGAAATTCAACGAAAAGGAAATTGATTTTTTGCACCAAGACCACTTAAAACCGGAGTATCTTCAATTGAACCCACAACATACAATCCCAACCCTTGTCGACGACGATTTTATTCTGTGGGACAGCCATGCCATTATGATTTATCTGGTTTCAAAATATGCCAAAAACGACGCTCTTTATCCCGCCGATTTGAAAAAACGGGCGGTTATTCACCAAAGACTTCATTTCGAGTCGGGAGTTTTAGCGGTTCAAATGAGAAATTTTGCTTTTGCAGTTCTTTACGAAGACAAAACCACGATTGATCAGAAAGATAAGGACGCTATTCAGGAGAGTTACGCAATTATGGAAACTTTTCTAGAAGGGAAAAATTGGATGGCTGGTGATTCTGTTACAATTGCCGACTACAGTTTAGTCGCAACTGTTAGTACCCTCAACGCATTCGTCTCAATTGACACGGAAAAGTACCAAAAACTGGCAAAGTGGGTGCAACGGTGCGAAAGCCTCCCCGAGTATCAGGTTAACAGGAAAGGATTAATCGAAGTTTACAACATACTGAAAAATAAATTGTCAATATAA

>TC004444Epsilon

ATGGCTCCAACAGTTTACGTCACACATGTAAGTCCTCCAGTTCGTGCTGTTTTGATAACTGCTAAAGCCATCGGTTTGGCTGTGGCAGAAAAAGAAGTGAATTTATTTGCTGGCGAACATCTAAAACCCGAATACCTCAAGTTGAACCCTCAACACACTGTTCCAACACTTGTTGACGATGACGGCTTTACAATTTGGGATAGTCATGCAATTATCACTTATTTGGTGTCCAAATATGCAAAAAACGACGCTTTATATCCGAAAGACTTGAAAAAACGTGCGGTGGTTGATCAGCGGTTGCATTTTGAATCGGGGTTTGTTACCCCTAGGTTAAAGGCAGTTGTTCTCCCAGTTTACTTAGAAGGTAAGAAGACAATCACCCAACAAGATAAGGAGAGAATTTGTGAGGCTTACGCATTTTTGGAAACTTTTCTAAACGGACACCAGTGGGTAGCTGGTGATTTTATCAGTGTAGCTGATTACAGTTTAGTCTCAATTATAAGTAGTTTGCACTATATTTTGGTGCCGATTGACGCGGAAAAGTATCCTAATTTGCAAGCCTGGTTAAAACGGATGGAAGGTCGGCCGGAGTATGAAGCCAATGTGAAAGGATTGGAAGACTACCGCCAATTATTAAAAAGCAAAATGACGAATTTACCTTGTTGA

>TC004443Epsilon

ATGGCTCCAACTGTTTATCTCATCTATGCAAGTCCTCCAGTTCGGGCTGTTCTAATGACCGCTAAAGCCATCGGATTGACTCTAACAGAAAAGCAAGTGAATTTGTTAGCCGGCGATCATCTCAAACCCGAATACCTCAAGTTGAACCCTCAACACACCGTTCCAACACTTGTTGACGACGACGGTTTTACAATTTGGGACAGTCACGTAATCATCACTTATTTAGTGTCCAAATATGCAATAAACGATGCTTTGTATCCCAAAGACTTGAAAAAACGTGCGGTGGTTGACCAACGTTTGCATTTTGAATCGGGGTTTGTTTTCCCTCGATTAGCGGCGATTTCACGCCCGATTTTCTTTGAAGGTAAGAAGACAATAAATCAAAAAGACAAGGAGAGTGTTTGTGAGGCTTACGGGTTTTTGGAAACGTTCCTGAACGGACACCAATGGGTTGCTGGTGATTTTATCAGCGTCGCTGACTACAGTTTGGTTTCAGTTATTAGTAGTTTGAATTATATTATGGTGCCCATTGATGCGGGGAAGTATCCTAATTTGCATGCGTGGCTAAAGAGGATGGAAGAGCGGCCGGAATATGCAGCCAATGTGAAAGGACTGGAAGACTACAGCAAATTATTGAAAAGCAAAATGGATAGTGCTTGA

>TC004442Epsilon

ATGGCTCCTACTTTATATCTTCATCATGCAAGTCCTCCAGTTCGGGCTGTTCTAATAACTACTAAAGCTATCGGATTGCCGATAACTGAAAAAGAAGTAAACTTCGCTACTAGTGAAAACCTCAGTCCTGAATATCTGAAGATGAACCCTCAACACACTGTTCCAACACTTGTTGACGACGACGGATTTACAATTTGGGACAGTCATGCAATCATCACTTATTTAGTGTCCAAATACGCAAAAAACGACGCTTTATATCCCAAAGACTTGAAAAAACGTGCGGTGGTTGACCAAAGTTTGCATTTTGAATCGGGGGTTGTTTTTCCCCGATTAGCGGCAATTTCATGGCCGATTATCCGTGGTGGTAAAAAGTCACTAACTCAACAAGAAATGGACGGCATTTATGAGGCTTATGGATTGTTGGAAACTTTTCTAGACGGACATGAATGGGTTGCTGGTGATTTTATCAGTGTTGCTGATTATAGTTTGGTTTCAATTATCAGTAGTTTGAATTATATTTTGGTGCCCATTGATTCTTTAAAGTATCCTAACTTGGACGCGTGGTTAAAAAGGATGAAAGGTCGGCCAGAATATGCCGCTAATTTAAAAGGATTGGAAGACTACAGCATTATACTAAAAAGCAGAATGGCTAGTGCATAA

>TC000522Theta

ATGCCGATAACACTTTATTCCGTTTCCGATGGACCCCCATCGCTGGCAGTAAGACAATGTTTGAAAATGTTAAACGTGGAATTTAATCTCGTAAATGTTGATTTTGGCTTAGGAGAGCACATGACTGAAGAATACGCAAAGAAAAATCCACAAAAAGAAATTCCAGTGCTAGATGACAACGGGTTTTACCTTGGAGAGAGCAATGCCATTCTTCAATATCTGGCCGATAAATACGGAAAGGACGATAAGCTTTACCCTAAAGATTTGCAGACAAGAGCCATCGTGAATCACCGCTTGTGTTTCAATCTTTCCACCTACTATCGCTACATTTCCGAGCATGTGATGGCCCCAATATTTTTCGATTACGCCCGAACTCCCCTAACTCTTAAAAAGGTCCACATCGCTTTGGACAACTTCAACACGTATTTGCAGCGACGTGGGACGAAATATGCAGCTGCAGATCACATAACCATCGCCGATTTCCAACTGGTGACGGCAACAATGTGCCTGGAAGCCATCAATTTTGATTTCTCCTCATACCCCCTGGTGACCAAATGGTATGCCACCTACAAGAAGGAGTACCCCGAGCTGTGGGCCATTGTGGAAGGGGGAATGAAGGAAATAAGCACGTTTGAGAAAAATCCACCGGATTTGAGTCACATGAACCATCCGATTCACCCAATCAGGAGGCATTAA

>TC003231Sigma

ATGTCCCCTTTATATAAACTGACCTATTTTGCTACCCCAGGTAGGGCAGAAGCCATCCGTTTTCTGTTCAGTTATGCAGCTGTCGAATTCGAAGATGTGAGAATTGCTTATGAAGACTGGCCAGCATTAAAAAACCAAACACCCTTTGGTTTTCTCCCTATGTTAGAACATGAAGGCAAAAAAGCACATCAAAGCGTGGCAATTATGAGATACGTAGCCAAACAGGTGAAACTTGCAGGAAACGATGACTGGGAAGATTTAGAGATTGATGCAACCGTAGACACACTCAGAGATTGTTCTTCAAAATTTCATCCACTTCGCCTTGAGACTGACGAAGAGAAAAAGAAAGCTTTACTAGAACAACTTTTTAAAGAAACAGTTCCGTATTTTATGAGGCGTTTTGAGGCTTTAGTTCAGAAAAATAACGGTTATCTGGCGCTAGGACGACTGACTTGGGCTGATTTATACTTTGTGGCATCGATAGCAGCAGGTGTAAAACAATATACCGACATCGATATAATAAAAGAATATAAAACACTGGCAGAGCTGCGCACTAAAGTTTTGGAAAATCCTCGTATTAAAAAGTATTTGGAAAATCAGCCTAAACTTTAA

>TC003232Sigma

ATGGCACCAGCTTACAAATTGACTTATTTCGAGTACACAGGCCTTGCCGAAGTCTCCCGTTTCCTTATGAAATTCGGTGGAATTGATTTTGAAGACTGTCGGATCAAAATGGAAGAGTGGCCTCAACTGAAAGCCAAATTTCCATTCGGCCAAGTGCCTGTGTTGGAGTATAAGGGAAAAATTGCGTGTCAAAGCCTTGCAATTGCACGATATTTAGCCAAACAAGTAAAATTGAGTGGAAACGATGATTGGGAAAATCTCGAAATCGATGCTACCATTGACACCATCAACGATCTTCGCATGAAGCTTGCAACTTGGTGGTTTGAGGCAGACGAGGCGAAAAAGAAGCTCATTGTTGAGAATTTCAAAAAAGACAATTTGTCCTACTATTTACCCCGTTTAGAGGCAATCGTTAAAAAGAATAAAGGCTTTTTGGCAGTTGGACGACTCACTTGGGCCGATTTTTACTGGGCGACAATTTCACCTGTTTTTGATATGGTCACAGGGATTGACGCACTTGCTGATTATCCAGAATTAAAAGCTGCCAGAGAAAGAGTTAACGCTTTACCAGCTGTCAAGAAATGGATTGATGAGCGACCCAACAACTGA

>TC003233Sigma

ATGGCACCGGCATACAAATTGACTTATTTCGACGGAAGAGGTCTTGCTGAAACCTCTCGTTTCCTCATGAAATACGGAGGAATTGATTTCGAAGATTGCCGGATCAAAAGAGAAGAATGGCCTCAATGGAAACCGAAATTCCCCTTCGGTCAAGTGCCTGTCCTGGAGCACAAAGGCAAAGTTGTTGGTCAGAGCATCGCAATCGCCAGATATTTGGCCAAGCAAGTCAAATTAGTGGGAAATGACGATTGGGAAAATCTCGAAATCGATGCAATCGTTGACACCATCAACGATCTTCGAATGAAACATGCGGCTTGGTTCTACGAGCCTGACGAAGCGAAAAAGAAGGTCATTATTGAGAATATCAAAAAGGACACTTTGCCCTACTATTTGCCCCGTTTGGAGGAAATCGTCAAGAAGAATAAAGGATTTTTGGCAGTTGGACGGCTCACTTGGGCCGATTTTTACTGGGCGACCGTTTCACAAGTTTTCGATGTGGTTAACGGGATTGACACTCTTGCCAATTATCCGGAACTGAAGGCCGCAAGAGAAAGAGTTAATTCTTTGCCAGCCATCAAAAAATGGATCGAGGTGCGACCCAAGACTGATTTCTAA

>TC003104EpsilonP

ATGGCGCCTACTCTGCATCTAATTTACGCTAGTCCCCCTGTACGAGCTGTTTTAATGACGGCTAAAGCCATCGGATTGACTTTAAAAGAAAATGAAATTAATCTTTTTGGTGGCGACCACATGAAGCCGGAATTTTTGAAGCTTAATCCCCAACACACTGTGCCAACACTTGTCGATGATGATGGTTTTGCAATTTGGGACAGCCATGCAATTATCACTTACTTGGTTTCAAAATATGCCAAAAATGATGCTTTGTACCCTCAAGATGTAAAAAAGAGGGCAGTGGTTGACCAAAGGTTGCATTTTGAGTCGGGTACTGTCTTTGTCCGGCTGTTAAAAATCACTAGGCCGATACTTTTCGAAGGGAAGACCACAATTGATCCAAAAGATAGAGACAATATTTTGGAAGCGTATGGATTTTTGGATGTTTTTCTAAATGGGAAGCAATGGGTTGCTGGCGATTTTATCAGCGTAGCTGATTACAGTTTAGTGTCTTCTATCAGCAGTTTAAATGTACTAATACCCATTGATGCGCAAAAATATCCCAATGTGGTTGCGTGGCTGAAAAGAATTGAAGCACACTACTAA

>TC003345Epsilon

ATGGCCCCAACTTTGTACATGGTGTACCCAAGTCCGCCGGTTCGAGCTGTTTTGATCACAGCAAAGGCCATTGGACTTGAATTAGAACTCAAAGAACTGAACTTGACCGAAGGCGAGCATCTAAAACCGGAATTTCTCAAGATCAATCCTCAACACACTGTACCGACTCTGGTTGAAGAGGACGGTTCCGTTATTTGGGACAGCCATGCGATTATGACATATCTTGTATCAAAATACGGTCAACACAAGGAATCCCTTTATCCCAAAGAACTCCTCAAGCGTGCTATCGTTGACCAAAGACTACATTTCGAGTCCGGGATCGCTTTCCCAAGAATGCTTCAAATTGCGGGCCCAATATTGCGCAAAGGAAAAACGAGTATAGAGCCTGAGGATGTGAAATTAGCCCATGAGGTTTACGCCTTTTTGGAAAAATTCCTCGATGGGAAAAAATGGGTAGCCGGTGGTCACGTAACAGTGGCTGATTATAGTCTAATTAGCACTATTAGTACGCTAGACCTGTTTGTAAGTGTGGAGGAGGATATATTTCCCAAGGTGAAAACGTGGATGAAGAAAATGGAATCTTTGGGAGAGTATGAAGCGAACAGAAAAGGACTGCAAATGTCCAAGGAGACTGTGGAAAGCAAACTGACAAAAAAATGA

>TC003346Epsilon

ATGGCTCCAAAACTGTACATGATTCCATACAGCCAGCCAGTTCGGGCAACTTTAATGACAATTAAGGCTTTGAATCTTGATGTAGAACTAGTTGAAGTAAATCTATTAGACAGAGCGCAGTTTTCGTCAAATTATACCAAGTTGAATCCCCAACACTCTGTCCCAACATTAGTCGAAGAAGACGGTTTTATATTATGGGACAGTCATGCCATTATGGCCTATCTGGTCGACAAATACGGCAAAGACGATTCTCTCTACCCCAAAGACTTGCAAACGCGGGCCATAGTCACCCAAAGGTTGCATTTCGAAAATGGAATTCTTTTTCCCAGGACCATGGACCGCCCTGTTATTTACCAAAGCGTCAAGACTGTAACTTCAGCACAAAAAGACGCAATCAAGGAAAGTTACAATTTTTTGGAGAAGTTTTTAGAGGGTAACGATTGGGTTGCCGGAAAATCCGTCACTATCGCAGACTTTAGCATAGTTTCGTCAATTACCACAATGGATATTGTGGTTCCGATTGATGCAAAGACGTACCCGAATATCACTTCTTGGATCAAACGGTGCCAGAAATTGCCTTATTACCATCTCAACCAGAAGGGTTGTGACGAAATCAGCAATATTTTTATCTCCGTCATGGCCGTGATTAGGTGCTTCAGTTCTTCCTTCGCCATGGCGCCCATTCTCTACATGCAGCACTTCTCGGGACCTGTAAGGTCAGTATTGTTGACGGCCGCTGCTTTAGGACTGAAACTGCAGCACAAGATCGTCGATCTGTCGAAGCAGGAACACCTAACCGAAAATTTTCTTAAGCTGAACCCGCAACACACTATACCCACTCTGGAAGACGATGGTGTTGTTATTTGGGACAGTCATGCCATTAATGCTTATTTAGTGGCGAAATACGGGAAAGATGACTCCCTCTACCCTAAGGACCTGGCCAAGAGAGCCATGGTGGACCAAAGAATGCACTTTGACAGTGGACTAATTTTCAGCTGGCTCAGGAACATTGCTCGAGGGGTGAAATACAAGGGGCGGAAGGCACTAACTGAGGACCAAATCGAAGGACTAGAACACGGGTACGAACATTTGAACACGTTCCTGAAGGACAGCAAATGGGTGACTGGTAATGCAGTCACCATAGCAGACTTCAGCTTGATTGCCAATGTTACTACCCTTAATATTATATATCCCGTGGACAAGTCCCGTTATGGGAACATCAGCAGGTGGCTGAAGGACTCGGAAGCCCTTCCCTACTATGACGTAAACCGGGAGGGTCTGGACCACTTCAAAGCGACAGTGAAACCCTTGCTGTCATAA

>TC003347EpsilonPartial

ATGCGCCCCAAGTTGTACATGAGCGAGATATGTCCCTCAGCTAGAGCCGTCGTGTTGACAGCCAAGGTCCTCGAACTCACCCTCGAGCTGAAGGAGGTTTCTTGCGACAAAAAGTTGAATCAACAATTCTCCATCCCTACGCTGGAGGACAGCGGCTACGTGATTTGGGACAGCCATGCCATTATTGCCTTCCTGGTCGGGAAGTACGGCAAAGACGACTCCTTGTACCCCAGAGACAACCCCCGGAGGGCCATTATAGACGAACGATTGCGGTTTGACTCGGGGGTCGTGTCTTTTTTCACCAAAACGATTCTGAACTCGATACCATACGAAGATAATGAAAAAGCCGTCAATGAAATTTATTCCTTGGTTGAGGAATTCTTCGATGGTAACAACCCATGGATCGCTGGGGATGCTTTAAGCATCGCAGACTTAAGTCTAATTCCTTCGATCACGTCGCTGGATGTTGTGGTCCCTATTGACCCCAAGCGGTTCCCTAAGTTAGCAAGATGGGTGAAAAGAGCCGAAACAATGCCCTTTTTTGAGGCAAATAAGACCGGGCTCTGTAAACTCCGAAGGATTCTAAACCGGTGTTAA

>TC003348Epsilon

ATGGCCCCCAGAGTATTCGTGACCATCGTGAGTCCGAACGTCCGATCCACCCTCCTGACCACACACGCCCTAGGAATTAACATCGAACTGGAAGAGATCGATTTGAACAACAAGGAACAGTTCAAGCCCAGCTTCATCAAGCTCAATCCGCAACATACCGTACCTACTTTGCAAGACGATGATGATTTCGTCGTCTGGGACAGCCACGTGATCAATGGGTACTTGGTGGACAAATACGGAGGAATCGACGATTCTCTTTACCCCACCGACATGCAGGAAAGAGCCAAAGTCAACCAAAAATTGCACTTTGATACTGAATTGTCATTGCTCGCCTCCAGAATCATGAAAGGGATCCTCCATGGGGGTAAGAAGAGCGCCCCCCAGGAGCAAGTGGACGAAATCCTGGAACGTTATGACTTTTTGGAGAAGTTCCTGGCAATGAACACCTTCGTAGCGCTGGGACACATGACCATCGCTGACTTCAGCCTAATCGCCACAGTGTCGACCATCGACATTTTCGTGCCGCTTGACGCGAAAAAGTACCCAAAAATCGCCGCCTGGATGAAGAAGATGCAAGCCCTCCCCTATTACGCCGCCAACAAGAACGGTCTCGACAAAATCCGAAACCAGATCGCGGCGGCTTTGGAGGGCTAG

>TC003103Epsilon

ATGGCACCAAAGCTCTACATCATGCAGCTGAGTCCTCCCTGCAGGGCTGTGCTGATGGTGGCCAAGGCCATCGGTCTTGAACTTGACATCGAGGAGGTCGAGCGCGAGGCCCTAAAGACGCCCGAGATGCTGGAGCTCAACCCCCAGCACACCGTGCCTATTCTCGTGGACGGCGATTTCGTCGTCTGGGACAGTCATGCGATCGCTGGGTATCTGGTAGGTCAGTACGCCGAGGACGACACCCTTTATCCCAAGGACGATATCCGCAAACGGGCGATTATTGATCAGAGGCTGCACTTCGAAAACGGGGTGCTCTACGAGAGATGTCGCGCAGTTGCCCAAATTCTTTTTTCTGGTATTGGCGACATCTCGGAAGACGACCGCGACAAACTGCTTGAAGCTTACGGCTTCCTGGAAGAGTTCCTCAACGGCCACCCCTGGCTCGCTGGCGACGAAATGACAGTGGCTGACCTCAGCGTCCTTGCGACTTTAGCCTCAGCCGACCTCTTTATCCCTGTGGATTCAGATCGCTTTCCCCAACTCTCCGACTGGTTAAAAACCGGAAAAGAGCTGCCTTACTTCGACGAATGCAATGCCGAAGGCTTGGCAAAGTTCAAGGAGATGCTCCCAACTCCCGAATCGTAA

>TC003496Sigma

ATGGCTCCTGCATATAAATTAACCTACTTCCCAGTGGAGGCCCTCGCCGAACCCATCAGATTCCTCCTCAACTACGGCGGAATCGAATTTGAGGACCACCGCTTCGACCGCGAAAACTGGCCCCAACTCAAACCCAACATGCCCTTTGGCCAAGTCCCCATTTTGGAATACAACGGGAAGGTGGCACACCAAAGTGTTGCCATGGCTAGATTTTTCGCCAAAAAGGTCAAACTAGTCGGCAATGACGATTGGGAAGATCTGGAAATCGACGCCATCGTGGACACCATCAGCGATTTGCGCCAAAAAATCGCCCTTTACCATTACGAGCAAAACGAAGCTGTTAAGGAATCTAGGAAAGAGCCCCTATTTAAGGAGACCATCCCTTATTACTTGCAACGTCTGGATGCCATTGTCAAGGCTAACAATGGACATCTTGCCGTTGGAAAGCTAACATGGGCCGATTTATTCTTTGTTGCCCTGTTGAAATATCTGTGTTTTATGTGCGGTAGCGACATAATCGCCGACTATCCGAACCTTGTTGCTTTGAAAAAAAGCGTTTTGGAAATACCAGCAATCAAGAACTGGGTAGAGAAGCGTCCGAAGTCTGACATGTAA

>TC002878Sigma

ATGTCTCCTAACTATAAGCTGATTTACTTCAATGCTAGAGGGCGAGCCGAGCATATCCGTTTTATATTCGCTTATGCCGGCGTGGAATACGAGGACGAGAGGATCCCTCGAGAAAAATGGCCAGAAATCAAAAAAAGAACGCCGTTTGGAATGCTCCCAGTCCTTGAAATAGATGGAAAAGCGGTGGCTCAGAGCAACGCTGTGGCAAGATATCTGGCGAGACAATATGGCTTAGCAGGACGAAATGAATGGGAAGCTTTGCAATGTGACGTCTTGGTTGACACTCTAGGAGATCTCAAACAGGTTCTTGCACAATTTCGAATGGAACAGGATCCGATCAAGAAGGAAGAAAAGAAGGCCCGCTTGATGAAGGAAACAATACCTTTTTATTTATCCAAATTCGAGAAAATTCTCTCGGAAAATAACGGATTTTCAGTGGGATCTGAGATAACCTGGTGCGATTTTGTGTTCGCTGTGTCTCTCGAAAACTTCGAGCACATCTTTGGCAAAGCCGCTCTGGATCAGTATCCGGCCCTGAAAGCTCTAAAAATAAAAGTCTATAGCATTCCTAGTATAAACGCATGGGTGTCGAAGCGACCCGCCACTGAATCGTAA

>TC000067Sigma

ATGGCACCAGCTTACAAACTCACTTACTTCGACGGCAGAGGCCTTGCAGAAACCTCCCGTTTCATCATGAAGTACGGAGGAATCGACTTTGAAGACTGCCGAATTAAACGAGAAGACTGGCCCCAAATTAAATCAAAATATCCCTTCGGCCAATTGCCAGTTCTTGAACACAACGGCAAAACAGTGAACCAAAGTCATTCGATTGCCCGATATTTGGCCAAACAAGTCAAACTTGCAGGAAACGATGACTGGGAAAACCTCGAAATTGATGCCATTGTGGACACCTTCAACGATTTGCGCTTGAAAATTGTGGCCTATTTCTACGAACAAGACGAAGAAAAAAAGAAAACTATTCTGGAAAATCTCAACAAGGACGTTTTCCCCCAATATTTGACACGTTTTGAAGAAATTGTTAAAAAGAACAAAGGATATTTCGCTTTGGGACGGCTGACTTGGGCTGATTTTTGCTGGGCAACTGTGTCTCCAGGTTTCGATATGATTACAAAAGTCGACACGATTGCGAATTATCCTGAATTGAAGGCAGTTAGAGACAAAGTTAATTCATTGCCGGCCATCAAAAAATGGATTGAACAACGACCCAAGACTGACTTTTAA

>TC000055omega

ATGCCTCAACCACATCTGACAACCGGGTCACCACAGCCCCCCAAAATCGAGGGAAAATTGCGCCTTTATTCAATGGAATATTGTCCGTATGCGCATCGAGTTCGGTTGGTTTTAAATGCTAAAAATATTCCGCATGACATTGTCAACATTAACCTGATTAATCAACCGGAATGGTATTTCAAAATACATCCTCAAGGCTACGTTCCCGCGTTAGACACCGGTTCCCAAATCGTCATCGAAAGCCTGCACATTTGCGACTTTTTGGACGAAAAATACCCTTCTCCTCCACTTTTCCCTCAAGACCCCGCCTCAAAACAACGCGACAAAGACTTACTCAAAAAAATCCAACCAATGCATGGTGTTTTCTTGCGTTGCATTTCTCTAAACGAGAATAAATCACTCGAAGAGTGGGCCACTGAATTTGTCCCTCATTTGGAAACATTTGAAACGGAATTATCCAATAGGGGGACTACGTTTTTCGGGGGTGAAAAGCCAGGAATGGTGGATTACATGTTGTGGCCTTGGGGTGAGAGGGTTGGTACTATTGTGATCGCTCACGGCCAACAATTGCCATTCGCTTCCGATCAATTTCCGCTTTTGAGGAAATGGAGGAAGGCCATGCGGGGGGATCCCGTGTGTGACGGGTTGTATTACGGACCGGAAAAATACTGGAAAATTATACAAATTAAATTTAGGAAAGCGCCCCCCGAATACACTGATTGTTTGATTGATTCAGGGTCACAACAACCACTCAAAGTGGATGGAAAACTACGACTTTATTCGGCCGAATTTTGCCCATATGCCCAACGAGTTCGATTGGTTTTAAAAGCTAAAAATATACCGCACGATATTGTTAATATTAGTCTCTCGCATAAGCCGGAATGGTACTCAAAAATCCACCCTGAAGAAAAAGTACCAGCACTTGACACCGGCACAAAAATCATCATTGAAAGCCTTGACATTGTGGAGTTTCTTGACGAGCAGTACCCCAAAAACCCCCTATACCCCCTTGAGCCTGAAGCTAAAAAACGTGACCAAGAACTGGTCAAAAAACTATCACCCCTTAGCGACGCACTTTTCAAATGTGTGTTGAGCCACAAAGTCAAAACTTTGGGGCAGTGTATGGCCGAACTCGTGCCCCAATTTGAGATATTTGAGACGGAGTTAGCAGCGAGAGGGAGCCCCTTTTTCGGGGGGCGCACACCTGGAATGGTTGATTACCTGTTGTGGCCCACTTGCGAACGTCTGGGTGTGTTAGCGATTGCTTACGGCGAACAATTACCTTTCGACGAAAATCAGCTTAAATTTTTGAAGAAATGGAACAAGGCAATGTTTGAAAATCCAATTTGCCAGGAAACTTACCACGAACCGGAAGAACATTGGATTGTTGTCCAACAAAAATTGAATTTTTTAAAAACGAAATAA

>TC000054omega

ATGGCTTCACCTCATCTAACAACCGGGTCACAACAGCCCCCCAAAATCGAGGGCAAACTCCGCCTCTACTCGATGCAGTTTTGCCCTTATGCCCAGCGAGCCCGTTTGGTTTTAAAAGCAAAAAACATCCCCCACGACATAGTCAACATCAATTTGATCAACAAACCCGAATGGTACACCAAAGTGCACCCTGAAGGTAAAGTGCCGGCTCTCGACACTGGCTCAAAAATAGTGGTCGAAAGCCTGGATATTGCTGACTTTCTTGACGCCGAATACCCCAACAACAACCCCTTATACTCCTCGGACAAAAACCGGGACAAAGAATTGATCAAGAAAATAGCCCCCATAACCGATCTTTTTTACAAATGCGTGGCCAAAACCGAGAACAAATCACTAGAAGAGTGGGCCAAAGCCTTCGTCCCCCATTTGGAAGTCTTCGAGAGAGATTTAGCCGCAAGGGGGACCACGTTTTTCGGAGGGGACAAGCCCGGAATGGTTGATTACATGTTGTGGCCTTGGGGTGAGAGGGCTGGTACTATTGCGATAGCTCACGGGGCGCAACTGCCTTTCGGAAGTGATCAATTTCCGTGTTTGAGGAAGTGGAGGAAAGCGATGCGCGAGGATAAAATCTGCAGCGAGATCTACAACGGGCCGGAAAAATTCTGGAAGTGTGTAGAGATGAAATTGAAGAATTTGCCACCGGATTATGATAGTATTTAA

>TC003873omega

ATGAATTCCAAACATCTTACCAAAGGATCAAGAAAACCATCTCCTGTTCGAAGGGGCAAACTCCGGTTATACAGTAATCGATTTTGTCCTTATTGCCAACGTGTTATCCTAGTCCTGGACGCAAAAAGGATACCATACGAAGTGGTTAACATAAATCTGGTCAATAAACCCGACTGGATGTTCGATAAGTCACCAATGGGTAAAGTACCGGCCCTTGAACTTGATAACGGTGAAGTCCTCTATGAATCTTTAATTATCGCTGATTATTTAGACGATAAATACAACGCCCACCGGTTACATGCAAAGGACCCTTTGCAAAAAGCGAAAGACCAACTTTTACTGAAGCAATTCACCAAGATTATTAACGCTTTACTGAAAACGGTCTCCTTGGGTCGAATTGAACACGACGAAGCACAAGTCATTTCCGAAGGACTTGCAATATTTGAAAGGGAATTAACTGACAGACCTGGGCCATTTTTTGGTGGTAGTAGGCCGGGAATGTTGGACTACATGATATGGCCATGGTGTGAACGTTCCGATGTGCTGAAGATTTATGGCAAGGACTACATTCTCAAAAAAGAAAAATACAAACAACTTATGGAATGGCGTAAGGTTATGGTGGAAGATGAGGCTGTGAAAAAGAGTTGGTGTAGTGCTAACACCCACATAAAATATTTGCAGTCATCCAGGGCTGGAGTACCCAACTATGACTTACTCATATAA

>TC007571Delta

ATGGGGAATTGTATGGAAAATGAAAGTGTTGTTCCTGTTAATAACCCTACGTTTGATCCAAAAACCATCAATCATACCATTGATGTTTATTTCTATCCGTTAAGCCCCCCGTCAAGAGCAGCTTTAATGCTTATTAAAGCCCTTGGAATTAAACATAATGTGAAAATCGTCAATATAATGGCTGGAGAACAAATGACACCTGAATTTTTAAAAATGAATCCCATGCATACTGTCCCTACGATAAATGACGGAGGGTTTATTTTATGGGACAGTCATGTTATAATGAAATATCTGGTTGAACAATACGCAAAAGATGATAGTCTTTACCCGAAAGATCCTAAAAAAGGGGCCATTGTAAACCAACGGTTGCATTTCAACACGACAACTTTGTTTCCTAAATTGTTGGATTACTGTGTGCCTGTGCTTTTTAATAATGAGGAGCCAGACCCTAACAAAGCGACAAAATTTGAAGAACTCTTGAATATCTTGGACGGATTTTTAAAGAACCAGTCATGGGTCGCTGGAGATAATTTAACAATTGCGGATTTTGCAATAATTACAGTCGTTGCCACAGCAGAGATCAAAATGCCCATCGACCTGTACTATCTTCCTGGAAGTGCCCCATGCAGGGCCGTCCTTTTGGCGGCGAAAGCCGTCGGTGTGGAGCTCAATTTAAAACTCACAGACCTCATGAAAGGCGAACACCTAACGCCTGAATTTATTAAGATCAACCCGCAACATACGATCCCGACCATGGTCGACAACGGCTTTGCCCTGTGGGAGAGCAGGGCCATAATGACCTACCTGGCCGACCAATACGGCAAAAACGATGCGCTTTACCCGAAAGACCCGAAAAAACGCGCCCTGGTTGACCAGAGATTGTACTTCGACATTGGCACTTTGTACGCCAGATTTGCCGATTATTACTACCCGGTGATTTTTGGAGGCGCTGAATACGAGCCCGCCAAACTGGAAAAAATCAAAGACGCATTCAAGTTCCTGGAGATTTTCCTTGAGGGTCAGGACTTTGTTGCGGGAAATCAGCTCACTTTGGCTGATTTGTCGCTTCTTGCAACTGTTACGACATTCGAAGCAGTGAATTTTGACCTTTCGCCGTACAAAAATGTGGTTAATTGGTTGGCGAGAGCCAAAGCTGCGGCGCCTGGGTATGAGGAGGCGAATGGGAAGGGGGCTGTGATCTTTAAGCAGATGGTCGAAAATTTAACGAAAAAATAA

>TC009482Epsilon

ATGGCTTTAACTCTGTACCATTTTCCGCCAAGTGCGCCCTCAAGAGCGGCCCTCCTGAGTGCCAAAGCCGTTGGTGTGAAAGTCGACGTCCAAATTGTCGATTTGTTCGCAAAAGAACAACTCAAACCCGACTTTGTCAAGGTTAACCCACAACACACCGTTCCCACGTTAGTAGATGGGGACTTCACAGTGTGGGACAGCCATGCCATTGGGCCTTATCTCGCAAAAACCCACGGAAAAGACGACACACTTTACCCCACAGACCCCAAAGAGAAAGCATTAGTTGACCAACGGCTGTACTTCGACTGTGGCACTTTGTACCCAAGAATTCGCCAAATTTGTTTCCCAGTGTTGTTTTTGGGTGAAGATGAGATCCTGGATGAGCACAAAACCGCCCTTGATGAAGCTCTGGGATTCCTCGATATTTTTCTCGAAGGAAATAGTTTTGTTGCAGGCGATAAGCTTACAGTGGCCGATTGTTCGTTAGTAGCTTCAGTTTCCAGCATTGTTGCTGTTGGTTGGGACATTACACCATATTCAAATGTGGCTTCATGGCTTGCTCGATGTGCCTTAACTATCCCTGATTATGGCGAGGCAAACCAGGCAGGAGCCGAGCAATTTGGCAAAGCGGTCCGAAGCAAATTAGCACCAGGACAAATATAA

>TC009842Zeta

ATGTCTGGTAAGCCTCTTTTGTATTCATATTGGCGCAGTTCGTGCTCGTGGAGGGTCCGGATAGCCCTAAATTTAAAAGAAATCCCCTACGACATCAAACCCGTGTCTCTCATCAAAACCGGGGGTGAGCAGCACACAAACGAGTTCAGAGAGGTCAACCCGATGGAACAAGTGCCTGCCCTCCACATCGACGGAGTCACCTTGGTGGAGTCTTTGAGTATATTAGCTTATCTGGAAGAAACGAGGCCCCAAAGGCCGCTGCTTCCGCACGACGTGGTCAAACGTGCAAAGGTCCGCGAAATTTGCGAAGTAATTGCGTCAGGAATCCAGCCTTTGCAAAACCTCGTCGTCCTCATTCACGTCGGGGAGGAAAAAAAGAACGAATGGGCTCAACACTGGATCAACCGGGGCTTCAGAGCCGTGGAGAAGCTGCTCTCGGCCAGCGCGGGAAAGTACTGCGTCGGGGACGAAATCACTCTAGCGGATTGTTGTTTAATACCACAAGTTTTCAACGCGAGGAGATTTCACGTGGACCTGCGACCGTTTCCAATTATTTTGCGGATTGATAGGGAATTGGAGAATCATCCGGCGTTCCGAGCGGCTCATCCGAGCAATCAGCCAGACTGTCCCCCCGAAATTGCCAAGTAA

>TC006215Theta

ATGACTCTCAAATTGTATCTCGATTTTTTATCACAGCCCTCAAGGGCTTTGTATATTTTTTTCAAAATTAATAAAGTGCCCTTTGAGCTGTCCCAGGTCGCACTGAGAAAAGGTGAACATTTGAGTGAGGAATTCAAAACAAACTTGAATCGATTTCAAAAAGTGCCTTTCATACACGATGGCGATTTTAGACTCACCGAAAGTGTGGCTATCATTAGATACGTTTCAAAAGTGCACAACATTGACAACAACTGGTACCCCAAAGAGACCAAAGCCCAAGCCCGAGTCGATGAGTACCTCGAGTGGCAACACAACAACACTAGAGCCTTCTGTGCCCTCTATTTCCAAAGAAAATGGCTTTTCCCACTGTTGACCGGACGGCAAACGAGTCCTGAAACTATGCAAAAATACGAAGACAACATGCTTGCTTGTTTGGACCAAATTGAAAATATTTGGTTAGCTGACACGCCCTACTTGTGTGGGGACAGGATCTCGGTTGCTGATATTTTTGCCGCTTGCGAAATTGAGCAACCACGGGTTGCCGGGTTTGATCCGATTAAAGGACGGCCCGTTTTAAGCGCTTGGATGAATCGGGTGCGAAGCGAGGCAAGCCCTTTTTATGAAGAGGCGCACGCTGTTTTAAATAAACTGGCAGAAAAGGGCGGAAAGGCCAAACTGTGA

>TC003336Ndomain

ATGTTTTATCGACGATTAAAGCGTATCCTGCCAGTAGTGTTTGCGCTGCGTCAACGACACTTGGCGTTTTTGGTTTGTGCAGCCGCGAACGAGGTTAGCGAGAGTTGTGATCAGCGCGGCGATAGTCTTGTCAAGTGCACCGCCAAAAATCAGTTTGTCAACTTGGGAAATATGCGGAAAGGCGCCGAGGGGTTCAAGCTTGAGGTCTCCGATGGCTTGCTGTTGTACTACGATCGCTTCTCGTTTTATTCCCAAAAGGTGGTTATGGCTCTCCACGAGAAGAACCTCGCCTTTGATAGCAAAATAGTAAACTTGATCAAAAACGAGCAATATCAGCCTTGGTACCTCTTCCTGAACCCTCGAGGCGAAGTCCCTGTCCTTCAGGACACTGGCAAGATAATTCCAGACTCGGCCCGCATTATCGACTACTTGGAGGACAATTTCAGCAATGGGGACACTCCCAGACTTATCCCCATGGATCAGGGAGCTGAAGTCAGGCAGAGGGTCACCCATTTCAGACACATCCTGCAGAAAGCGCCTGCAGGAATGCTAACAACTGGCGCTATGCTCAACCAACACCTTTTAAAAAATCCGAAAAGTCCGTTTTTTGCACCGATTCGAAAGGCACTTGCCAATGCTGAAAAGAACAGTGGGAAGCATTTGCGGGAGTATGCGGAGAAGAATCCAGAGGCCAAAGAAATACTGTTGAAGAAAGCCAGTGTTAGGGAAGAACAGTACAAACAACTGTCTGATGAGAAGAATTTCAGGGACGTCCTAAATCAAGTACATACGATTCTGGATGAGATTGAAGCAGAGTTGGAGAAGCACAAGGGTGATAAGGAAGACTGGTGGCTGTGTTCTGATAGATTTACAATAGCCGATGTTGAGTTGACGATTTTATTGATCAGGATCAGTCAGTTAGGGCTTGAACACATCTTTTGGAGTTCGGGAAAGAGGCCTTGTATTGAAAAATACTACGAGAGGGTCAAGGCAAGGGATTCGTTCAAGAAGACAGTTCCAGGGACCTTAGTGTTGCTAAAAACAATACTCATGTCACAAGCACCGATCGTTTTCGGGGTTGTGGCTGCTGTGGCGTTGGTCGTTGGAGGGGCGATCATCGCAAAGAAGTACATTGCTTAG

>AGAP005749omega1

ATGAGCAACGGAAAGCATCTCGCCAAAGGTTCCAGCCCACCATCCCTCCCGGACGATGGTAAGCTGCGCCTGTACTCGATGCGCTTCTGCCCGTACGCCCAGCGCGTCCACCTGATGCTGGACGCGAAGAAGATCCCGTACCACGCGATCTACATCAACCTGTCCGAGAAGCCGGAATGGTACCTGGAGAAGAACCCGCTCGGCAAGGTGCCGGCACTGGAGATTCCCGGCAAGGAGGGCGTCACCCTGTACGAGTCGCTCGTCCTGTCCGACTACATCGAGGAGGCGTACTCGGCCCAGCAGCGCAAGCTGTACCCGGCCGACCCGTTCAGCAAGGCGCAGGATCGCATCCTGATCGAGCGGTTCGCCGGCTCGGTCATTGGGCCGTACTATCGCATCCTGTTCGCGGCCGACGGCATCCCGCCCGGTGCCATCACCGAGTTCGGCGCGGGGCTGGACATTTTCGAGAAGGAGCTGAAGGCGCGGGGCACCCCGTACTTCGGGGGCGATAAGCCGGGCATGATCGACTACATGATCTGGCCGTGGTGCGAGCGGGTCGATCTGCTCAAGTTTGCGCTCGGCGATAAGTACGAGCTGGACAAGGAGCGGTTCGGTAAACTGCTGCAGTGGCGAGAGCTGATGGAAAAGGACGACGCCGTAAAGCAGTCGTTCATTTCGACCGAGGACCACACGAAGTTCCTGCAGAGCCGCAAGAACGGCGAAAACAACTACGATATCTTGGCGTAA

>AGAP006132GSTPartial

TTCCCTCTCCCGCAGGTTCTACAGGCACTGCACGAGAAAGGGATCCGGTTCACCAAGTATGAGATCGACGTCACGAACGATGAACATTTCTCCGAATGGTTTCTGGAGCTGAATCCACGTGCCGAGCTACCGGTGCTGCAAAACGGTTTGCTCATCGTACCCGGCTCAAACCGCATACTGGACTATCTGGAGGAAAACTACCCCAAAAACAAATCGCTACGCATGCCGATCGAAACGGATAAGCTGGTCGTTGGCTTTCGGCAAACGATCGAACGCCTGCCGATCGGGGTGATTACGATCGGATCCTTCCTGCATTCGCAGCACACGAGCAGTCCAAAGTTCCCGTTCGTGCTGCCCGTGCGTCAAACGATCCTAGCGCGGGACGAAACGCTTGCTCACCGGTTACGAGCGTACGCGACGGCATATCCTGCGTTTGCGGAGGTTTTGCTAAAGAAGGCGGACTTTCACGATCGCAAGCGCGCCATCATCGCTAGTGAGGAGTACTTTTGCAAGCTGCTAACGGCACTGGACGAATTTCTGTCCAGCGTCGAGCAGTATCTCGCGACGATCGACGTCGACCGCTGCTGGCTGGCAGGGACGGAGGACACGTTCACGATGGTGGATATCAGTCTCGGGACGCTGCTGCATCGCCTGTACGTGCTCGGGCTGGAGGATCGTTTCTGGGGCGAGGGCAAACGGCCCCAAGTGGCGCGGTACTTTGCGAAGAGTTGCCAACGCGAATCGTTCCAGTGTGTGCTACCGTCGAAGGTGTCGATCCTGCGCACGGTGTGGATCAATACGCCGCCCGTGTACAAGGCAGGGCTGGCGGCTGTCTCATCCGTTTTAATTAGTTCTACTCTTTTGAAACGATAA

>AGAP002898-PBZeta

ATGCCGGCAATTTTTGCGTGCTTGTCGAAAAAGTATGTGCTAGGCGCGGCTACTGCAGGCCTTAACAAGTTTCCACTGCTGCAGTCGTTTACCAACAGCTCCAAAATTAATTACTCCAAGTTCCATGGCTCGATGTCGCTCTCGGCGATGTCGAAGCCCATCCTGTACTCGTACTGGCGCAGTTCCTGCTCCTGGCGCGTTCGCATTGCGCTGAACCTGAAGGAAATCCCGTACGATATCAAACCGATCAGCTTGATCAAGTCGGGCGGCGAGCAGCACTGCAACGAGTACCGAGAGGTGAACCCAATGGAGCAAGTTCCCGCACTGCAAATCGATGGCCATACGCTCATTGAATCGGTTTCGATTATGTACTATTTGGAAGAAACGCGCCCTCAGCGTCCACTGATGCCCCAGGATGTTCTGAAGCGTGCCAAAGTGCGGGAAATCTGTGAGGTGATTGCATCCGGTGTTCAGCCACTGCAGAATCTGATAGTGCTGATCCACGTCGGTGAGGAGAAGAAGAAGGAATGGGCCCAGCATTGGATAACGCGCGGATTCCGTGCGATCGAGAAGCTGCTCTCCACGTCGGCGGGCAAATTCTGTGTGGGCGATGAGATCACCCTAGCCGACTGTTGCCTCGTGCCGCAGGTGTTCAATGCGCGCCGTTTCCACGTGGATCTGCGCCCGTATCCGATCATTCTGCGTATCGATCGCGAGCTCGAGGGTCATCCCGCATTCCGAGCGGCTCACCCATCCAACCAGCCCGACTGTCCACCGGAAGCTGCCAAGTAA

>AGAP003257GSTu1

ATGCCTGCCCCAACGCTGTACTACTTTCCGATGAGCCCACCGGCCCGTGCGGTGTTGCTGCTGATGAAGGAGCTTGAACTGCCGATGAATCTAAAAGAAGTGAACCCACTCGCCGGTGAAACGCGCACCGAGGAATTCATGCGCATGAACCCGGAGCACACGATCCCAACGCTGGACGACAATGGCTTCTACCTGGGCGAATCACGTGCCATTTTGTCGTACCTAATCGATGCCTACCGTCCCGGGCACACGCTCTACCCGAACATTCCGAAGGAAAAGGCACTCATTAATCGGGTGCTGCATCACGATTTGGGCTCTTTCTATCCCAAGTTCTTCGGCACCATCGGTGCACTGTTTTCTGGTGCAGCGACGGAAATTTCAGACGAAATGAAAACCACCACCCAGAAGGCGCTCACCGACTTGGAGCACTATCTGACACGGAACGATTACTTTGCCGGGGAAAATCTCACCATTGCAGACCTTTCCCTGGTGCCGACGATTGCTTCCGCCGTCCACTGCGGTTTGGATTTGACCAACTATCCACGACTAAACGCGTGGTATGAGAGCTGCCGGGTGCTGAAAGGATTCGAGGATGATCAGGAAGCTGCCCGCCAGGTTGGTGAATATTTGCGCTCCAAGTTTCCCACCGGTCTGGAGGCGTTGAATTGA

>AGAP004163-PBDelta7

ATGACTCCAGTGCTGTATTATCTGCCGCCATCGCCACCGTGCCGATCGGTGCTGCTGCTGGCCAAGATGATCGGCGTCGAGCTGGAGCTGAAAGCGCTGAACGTGATGGAGGGCGAGCAATTGAAACCGGACTTTGTCGAGCTCAACCCACAGCACTGCATACCGACACTGGACGATCACGGGCTGGTGCTGTGGGAGAGCCGAGTGATTCTTGCGTATCTGGTATCAGCTTACGGGAAAGATGAAAACCTCTATCCGAAAGATTTTCGCTCCCGCGCCATCGTCGATCAGCGGCTGCATTTCGATCTTGGTACGCTTTACCAGCGTGTCGTCGATTACTATTTTCCAACCATCCAGCTTGGCGCTCACCTAGACCAAACGAAGAAGGCAAAGCTGGCCGAAGCGCTCGGTTGGTTCGAGGCAATGCTGAAACAGTACCAATGGTCAGCGGCCAACCACTTCACGATAGCGGACATTGCGCTGTGCGTCACGGTCTCACAAATCGAAGCATTCCAGTTCGACCTCCACCCGTACCCGCGGGTACGTGCCTGGTTGCAAAAGTGCAAAGATGAGCTACAAGGACACGGCTACAAAGAGATCAACGAAACTGGAGCAGAAACGTTAGCGGGATTGTTCCGGTCAAAGTTGAAGCAGTAG

>AGAP004164-PCDelta1

ATGGATTTCTATTACCTACCCGGATCTGCCCCGTGCCGTGCCGTCCAGATGACGGCGGCCGCCGTTGGCGTTGAGCTGAACCTGAAGCTCACCGACCTGATGAAGGGCGAGCACATGAAGCCGGAGTTCCTGAAGCTGAATCCACAGCACTGTGTGCCGACGCTCGTGGACAATGGATTTGCTCTGTGGGAGTCTCGAGCGATCATGTGCTATCTGGTGGAGAAGTACGGGAAACCGTGCAACAACGACTCTCTGTATCCGACCGATCCACAAAAACGAGCCATCGTCAACCAGCGCTTGTATTTCGACATGGGAACGCTGTATCAGCGCTTCGGCGACTACTATTATCCACAAATCTTCGAGGGAGCACCAGCTAATGAGACAAACTTTGCAAAAATTGGGGAAGCTTTAGCGTTTCTCGATACTTTTCTCGAGGGTGAACGGTTTGTGGCGGGAGGAAATGGTTATTCGTTAGCTGATATTAGCCTGTACGCGACACTTACTACGTTCGAGGTGGCAGGGTATGACTTCAGTGCGTATGTAAATGTTCTGCGATGGTATAAGAGCATGCCCGAATTGATTCCCGCTTCGGACACGAACCGGAGCTGGGCTGAAGCAGCTAGGCCCTTTTTCGATAAAGTAAAACATTAA

>AGAP004165Delta2

ATGTTGGATTTTTACTATCTTCCCGGGTCGGCACCGTGTCGGGCGGTACAAATGGTGGCAGAAGCAGTTCACGTGAAGCTAAACCTTAAGTATCTCGATCTCATGGCTGGTGCTCATCGATCACCGCAGTTTACCAAGCTTAACCCTCAGCGTACGATCCCCACGCTGGTCGACGGTTCATTGATACTGTCCGAGTCGCGTGCAGCACTAATATATCTCTGCGATCAGTATGGCGACGAAGATAACGATTGGTATCCCAGAGACACTATCCAGCGAGCCATCGTCAATCAACGGTTATTTTTCGATGCCTGCGTGCTGTATCCAAGGTTTGCTGATTTTTACCACCCGCAAGTATTTGGCAACGCTGCTCCTGATGGCAGGAAGAGGCTAGCCTTTGAAAAAGCAGTGGAGCTACTCAACATCTTTCTTAGTGAGCACGAGTTTGTGGCCGGATCGAAGATGACCATAGCTGATATAAGTCTGTTTGCCACGTTAGCCACAGCATGTACGTTGGGATTCATTCTGCGACCGTACGTCCATGTCGATCGATGGTACGTCACAATGGTGGCCTCGTGTCCAGGAGCACAGGCGAACGTTTCGGGTGCGAAGGAGTTTTTAACATATAAATAA

>AGAP004171D8ATGGATTTTTACTATCATCCCGCCTCGCCCTACTGCCGTTCGGTGATGCTGGTAGCAAAGGCTCTGAAGCTGAACCTGAACCTGCAATTCGTTGATCTAATGAAGGATGAACAACTGCGACCCACGTTTACTGTGCTTAATCCATTCCATTGCGTACCGACGCTTGTGGACAACGATTTAACGATGTGGGAGTCACGTGCCATACTGGTGTACCTGGTCGACAAGTACGGACGCACCAACAGTCGTCTGTATCCGAAGGATGCCAAGACACGGGCCATCATTAATCAGCGATTGTTCTTCGATCACGGCACGCTTGGTACTCGGTTGGAGGATTACTACTATCCGTTATACTTTGAGGGTGCCACGCCCGGCGGCGAGAAGCTTGAAAAGTTGGAAGAAGCACTGGCAGTGCTGAATGGATATCTTATCAATAATCCGTACGCCGCCGGACCGAACATTACGCTGGCGGACTACAGTCTGGTGTCGACGGTGACGTCCCTCGAGGTGGTCCAGCATGACCTGAGCAAGTATCCGGCAATTTCGGCGTGGTACGAGGGCTGCAAGGCAACCATGGCGGACTTTCAGGAGATTAACGAGAGTGGAATGCAGCAGTACCGGGCTTACTTCGAGTCTGGTCCCACACCTCCAGCTACTGCATATGCCGTTGCGGAATAG

>AGAP004172Delta

ATGGATCTGTATTACAACATCCTGTCACCGCCATCGCGTGCCATACTGTTGCTCGGTGAGGCACTGCAGCTAAAGTTCAACCTGATCAGTCTGGACGTGCACCGGAAGGATTATGTGAACCCAGCGTTCAAGAAGATCAACCCACAGCACACGGTACCGACGCTGGTCGTCGACGGTGTGGCCATCTGCGAGCCAGGTGCCATTCTGATCTACCTGGCAGAGCAGTACGCACCGGCCGGAACCACCTACTATCCGCCTGATCCGCTGCGTCGGGCCATCGTCAACCAGCGATTGCTGTTCGAGTGCGGCACACTGTACAAGTGTATCTTTGTGTACTACAGCCCGGTCGTGCTGGAACGGGCCACACCGGTAGAGACCGATCGCCAAAAGCTGATCGAAGCGGTTGCCGTGCTGGATGGCATTCTGCAGCACAGTGCCTTCGTGGCCGGGGATTGTCTTACTGTGGCGGACTATTCGCTCGTGTGCACCGTTTCCATGCTGGTGGTGCTGAAGTTTGAGCTCGCGCCATATGCCGCTGTTCGGCGGTGGTACGAGCGGTGCAAGGAAGTGATTGCGGGTTACACCGACCTGACACAGCGTGCCGTTACAATGTTCCAAAAATGGATGGAGCAGGAAAACAGCAAAGGCTAA

>AGAP004173Delta5

ATGGAGCTCTACAGCGATATCGTTTCACCGCCCTGTCAGAATGTGCTCCTGGTGGCTAAGAAGTTGGGCATTGCGCTTAACATCAAGAAGACAAACATTATGGATGCAGCGGACGTAGCTGAGTTGACAAAGGTCAATCCGCAACATTTGATCCCAACGTTCGTGGAGGACGACGGACATGTGATCTGGGAGTCGTACGCAATTGCCATTTACCTGGTGGAAAAGTACGGCCAAGATGATGCCCTCTATCCAAAAGATCCCAAAGTGCGATCCATTGTAAATCAGCGTCTGTTTTTTGATATCGGAACGCTGTACAAAAACATTCTAGCCAACATTGACGTCCTGATCGAGAAGCAGCAACCTTCGGCCGAATTACGCGGGAAGCTGGAGCAAGCGCTTGATCTTACGGAGAAGTTTGTTACCGAGTGTCGTTTTGTTGCTACCGATCATCTTACGCTAGCTGACATCTTTATGCTTGGTAGCATTACTGCCCTGGAATGGTTTAGGTACGATTTGGAGCGTTATCCCGGCATTCGGGGATGGGTTGAGAGAGTAACGGCCCAATTCCCCGATTATTCCGACTTTCACAAGGAGATAAGGGAAGCCACTAAGCAGTACGTTGCAACGCACTGTCCGCATTTGGAATATTAG

>AGAP004378Delta11

ATGGATTTCTATCACCTTCCCCTGTCGGCCCCGTGCCAATCGATCCGTCTGTTGGCCAAAGCGCTCGGTCTACATTTGAACCTAAAGGAGGTCGACCTGTTGAAAGGTGAACATCTTAAACCGGAGTTCCTGAAGATCAACCCACAACACACCGTACCGACGCTGGTCGACAATGACTTTGTGCTGTGGGAATCGAGAGCGATCCTGACGTACCTGTGCGAAAAGTACGGCAAAAACGACGGCCTCTATCCGAAGGATCCGAAAAAGCGTGCCGTCGTGAACCAGCGCCTGTACTTCGACATGGGCACGCTGTACCAGCGGTTCTCGCAAGCCTTCTACCCGGTCATGATGGAGGGCAAGGAGCTGAACCCCGAACTGGTGGTGAAGCTAGACGAAGCGCTCGAGTTTTTGGAAAGCTTCCTCGACAAAACACCCTTCGCTGCGGGCGATAAGCTAACGGTTGCCGATTTCAGTCTGCTGACGAGCATCACTACCATCGACGTGACGGCCGGGCACGATCTCAGCAAGTACGCCAACATTCAGCGTTGGTACAGCCAGCTGAAGGAGTCGGTGGCGGGACATCAGGACATTTGCGTCGAAGGTGCCATCCAGTTCCGTGACTCGTTCAACCCGATCAACAAGTAA

>AGAP004379Delta6

ATGCCTCGACTCGATCTGTACTATAACATCATATCGCCCCCGTGTCGAGTGGTGCTGTTGTTTGCGAAATGGTTGAAGCTGGAGCTGAACCTCATCGAGCTGGACGTGTTGAAGCGCGACCACTACAAGCCCGAATTTCTCAAGCACTACATTCCCACGCTGGTCGACGCCGATGGTGACGTGGTGGTGTGGGAATCGAGCGCCATCCTGATCTATCTGGCCGAACGGTACGGTGCAGCAGACGACGATACGCTCTACCCGAAGGATATTGCACTGCGCGCGAAAGTCAACCAGCGGCTGTTTTACGACATCGGTACGCTGATGCGCAGCGTGACCACCTACTACCACCCCATCCTGATGGGGGGCGAGGGCAAGCTGGAAGACTTTAAGAAGGTGCAGGATGCGGTCGGCGTGCTGGACAGCTTTCTGAGCGCGAGCCGCTGGACGGCGGGCGATCACATAACGGTGGCGGATTTCGCGATCGCTGTGACGGTGGCCGCCCTGGACGGATTGCTCAACTTTGACTTTAGCGTGTATCCCAACGTGCACCGGTGGTACGAGCAGTGCAAGCGCGAGCTCGTCGGCTACACGGACATTACGAAGGAGGCGGCCCAACGGACGCAAGCCTTCCTGGAGCGATTTCGCGCTATGCGGGCCGCCGACCAGCAGCTACTGTGCGAACAGCAACGAACTGTGCGGCAAAGGCAAAAGGAGGATGGGACTGATGATCAACAGCAGCAGCAGCAGCAGCAGCAGCAGCAGCAACAACAGTACACCGAACTGCAGACGCAGCCACGATCCGGGCGGACACGCGACACCGCGCCGGACTACGCAAGAAAGCCGGATGAGTCGACAAGAATGGCCACGGAAGATTAG

>AGAP004380Delta12

ATGGATCTGTATTACCACATTCGCTCACCACCATGTCAGCCGGTTGTATTTCTGGCCCGCCATTTGGGTCTGGAGTTTAACCACATAGTGACGAGCATCTACGATCCGGCGGACTTTGAAGTGCTCAAGAAAGTGAATCCACAACACACCATCCCCACGCTGGTGGACAATGGCCATATTCTGTGGGAGTCGTACGCCATCCTGATCTATCTGGCGGAGAAGTACGCACTGGACGATAGTCTCTATCCGAAGGATGTTTGCGAACGATCGATCGTACATCAGCGGCTCTTCTTCGATAGTGGAATGTTCCAGAACACCACACTGCAGGCGGTATTGTCGCACTTGCGCAACAACCCAATCACGGACGAGCATCTGGCTAAGGTGAAGCGCGGTGTGGAGATAGTGGAGATGTATCTCACGGACAGCCCGTACGTTGCTGGCCAGAAGCTGACGATTGCCGATTTCTCGATCTTTGTAAGCTTCTGCTCGCTCGACATGATGAAGTACGACCTAACGGCGTACCCGAACGTGCAACGATGGTTCGCAAAGATGGGCACGCACATTCCCGACCTGGAGCCAACGCGCAAAACGATCGAGGAGGAGCTCCGGGCTCTGCTGCAATCGATGAACAAGTAA

>AGAP004381Delta4

ATGGATTACTATTGCAACTTCGTTTCACCGCCCAGTCAGAGCGTCATACTGGTTGCGAAGAAGCTGGGCATAAAGCTAAATCTCAGGAAAATGAACATATACGATCCCGTTGCAATGGATACCCTGTCCAAGCTCAATCCACATCACATCCTGCCGATGCTGGTAGACAACGGCACTGTTGTATTCGAACCGTGCGCGATCGTCCTGTATCTGGTGGAAATGTACGCCAAGAACGATGCGCTCTATCCGAAGGATGCGCTAGTGCGGTGCGTTGTGAATCAGCGGCTCTTCTTCGACGTTGGCACACTGTACAAACAGATCTACGAAAACGTGCACGTTCAAATGCGGAACAGTCAGCCCAGCGAGAAGCAGGTGCAAAGGTTGCAGAAGGCAGTCGACGTGCTGGAAAGCTTCCTTTACGAACGATCGTACACGGCCGCAGATCAGCTGACGGTGGCGGACATTTGTTTGCTAGTTACGGTCAATGCGCTCACGCTTTGGTTGGGCTACGAGCTTGCACCTTATCCACGGATCAGAGATTGGCTCGGGAGAGTGGTGGCAGAGCTTCCGGGTTGTGTTGAGTTTCAAAGGGAGGTAGAGGACGCCACCAGGGCGTACGTAGTCAATCGAAAGATTTGA

>AGAP004382Delta3

ATGGATTACTATTACAGCCTCATTTCACCGCCCTGCCAGAGCGCAATCCTGGTTGCCAAGAAGCTGGGCATTACGCTGAACCTGAAGAAGACGAACGTGCACGATCCGGTTGAACGTGATGCATTGACTAAGCTTAATCCGCAACATACCATCCCCACGCTGGTGGACAATGGGCACGTCGTGTGGGAATCGTACGCGATCGTGACGTATCTGGTGGAGGTGTACGGCAAGGATGACACGCTTTACCCGAAGGACCCGAAGGTGCGGTCCGTCGTGAACCAGCGGCTCTTCTTCGACATTGGCACGCTGTACAAGCAAATCATCGACATTATCCATCTGGTGGTGAAGAAGGAGCAGCCGACCGACGAGCAGATGGAGAAGCTGAAGAAAGCGATGGATCTGCTGGAGCACTTCCTTACCGAACGATCGTACGCGGCCGCCGATCACCTGACGGTGGCGGACATCTGTTTGCTCGGCAGTGTGACCGCACTGAACTGGCTGAAGTACGATCTCGAACCGTTCCCGCACATTAAGGGATGGGTGGCGCGTGTGACTGGGGAAATTCCGGACTACGCCGAGTTCCGGAAGGACGTGGAGGAAGCTACCAAAGCGTACGTCGCTAGCAAAAAGTGA

>AGAP004383Delta10

ATGGAGCTGTACTACAACATTGTGTCGCCGCCGTGCCAGAGCGTGCTGCTCGTGGGCAAAAAGCTGGGCATCACGTTCGACCTGAAGGAAGTGAATCCTCATCTCCCGGAAGTGCGCGAACAGTTGCGGAAGTTCAATCCCCAACACACCATACCGACGTTCATTGAGGATGGACACGTCATTTGGGAGTCGTACGCGATCGCCATCTATCTGGTGGAGAAGTACGGCAATGGCGACGATGCGCTCTACCCGCGCGATCCGAAGGTACGGTCCGTCGTGAACCAACGGCTGTTCTTCGACAATGGCCTTATGTTCAAGAGTGCCATCGAGTACGTGGAGTGTATTTTGAAGAAAAAGCTCGAACCAACGGAAGAAATGCAGCAGCGGCTGAAGAAAGCGCTTGGATTGTTGGAGTCGTTCGTGAAGGAGCGAGCGTTCGTCGCCAGCGATCATCTAACGATCGCCGATATCTGTCTGCTGAGCAGTGTGACACTGCTAACCGGCATCAAGTACGATCTGGCCACCTTCCCGGGCATTACGGCCTGGGTCGCTCGTGTTACCGGTGAGCTTCCGGACTACGGCGAGTTCCACAAGGAGCTGTACGAGAAGAGCATGGAGTACATTAAAACGCTGTAG

>AGAP010404Sigma1

CTTAGCTCAAGTTCGATCAGCAGATCTTCACTTAAGTGCAACATCATGCCGGATTACAAGGTTTACTATTTCAACGTAAAAGCTCTGGGCGAACCACTGCGGTTCCTGCTGTCTTACGGCAACCTTCCGTTCGATGATGTGAGGATCACCCGCGAAGAATGGCCAGCACTCAAGCCAACAATGCCGATGGGTCAGATGCCGGTACTGGAGGTTGACGGCAAAAAAGTGCACCAGTCGGTCGCTATGTCTCGCTATCTGGCCAACCAGGTCGGTTTGGCCGGCGCTGATGATTGGGAGAACTTGATGATTGACACCGTGGTCGACACGGTGAACGATTTCCGTCTAAAAATTGCGATTGTCGCCTACGAACCGGACGATATGGTGAAGGAGAAAAAAATGGTCACCCTGAACAACGAGGTGATTCCGTTTTATCTGACTAAGCTGAACGTGATCGCCAAGGAGAACAATGGCCACCTGGTGCTTGGCAAGCCGACCTGGGCTGATGTTTATTTTGCTGGTATCCTAGACTATCTAAACTACCTGACCAAGACAAATCTGCTCGAGAACTTCCCGAACTTGCAGGAGGTGGTCCAGAAGGTGCTAGATAATGAGAATGTCAAAGCTTACATTGCTAAGAGACCCATCACCGAAGTTTAG

>AGAP009190E8U4

ATGATTCTGTACTACGACGAGGTCAGCCCACCGGTTCGGGGCGTCCTGCTAGCGATTGCAGCACTCGGTGTGAAGGACCGCATCAAGCTCGAGTACATCGATCTCTTTAAGGGTGGCCATTTAAGTAGCGATTATCTTAAGATTAATCCACTGCACACGGTCCCCGTCCTACGGCACGGTGAGTTAACGCTTACCGATAGTCACGCTATTTTAGTGTACCTGTGCGATACATTTGCCCCACCAGGGCACACGCTAGCCCTGCCCGACGCACTGACGCGCGCCAAAGTTTTCAACATGCTGTGCTTCAACAACGGCTGTTTGTTTCAGCGCGATGCGGAAGTTATGCGTAAAATCTTCAGCGGTGCCATTACCGACCCAACGCAGCATCTGAAACCGATCGAGGCAGCGATCGATGCGCTGGAGCAGTTTCTGCAGCGATCGCGCTACACCGCACACGATCAGCTTTCGGTGGCAGATTTCGCAATCGTCGCGACACTCAGCACGGTGGCCATTTTTGTGCCGCTCCCGGCGGATCGTTGGCCGCGGGTATGCGAGTGGTTCGCGGTGATGGAAGCGCTGCCATACTACAACGACCAGAACCGTGTTGGGTTGGACATGTTGCGCAAACATTTAGCGGGAAAGATTAAGCTGTAG

>AGAP009191Epsilon6

ATGTCGAGCAAGCCGGTCCTGTACACGCACACGATTAGTCCCGCCGGCCGTGCGGTCGAGCTGACCGTGAAGGCGTTAAACCTTGATGTCGATGTTCGCGAGATGAACGTCTTCAAGGGTCAGCATATGAGCGACGAGTTCAAGAAGTTAAACCCCGTCCAAACGATCCCAACACTGGACGACAACGGGTTCGTGCTGTGGGACAGCCACGCCATCATGATCTATCTGGCGCGCCGTTACGGTGCCGAGTCCGGCCTCTACACGGAAGAGTACGAGCAGCAGGCCCGCATCAATGCGGCCCTCTTCTTCGAGAGTTCGATCCTGTTCGCGCGGCTCCGCTTCTGCACGGACAATCTGACCGTGCTGGGCAAGAGTGCGATACCGGAGGAGAACCTACAGCGTGCGCTGGAAGGGCTGCAGCGGCTGGAGAGGATGCTGCAGTCGGAGTATGTGGCCGGCGATCAGCTGACCATTGCGGATCTGAGCTGCGTGAGCAGTGTGGCCACACTGCACCTGATGCTGAAACCGTCGGCGGAAGAGTTCCCCAAAACGTTCGCCTGGATGGAACGATTGTCGAAGTTGCCGTACTACGGGGAGGTGATGGGACGAGGACTTAAAGCGGCCGGAGAGCTGATGCAGACGCTTGGTAGCAAGAACAGTGGTGGTGGTGGTGATGGCAACTAG

>AGAP009192E5

ATGGCAACGAACCCCATCATCAAGCTGTACACGGCGAAACTAAGCCCACCGGGTCGTGCCGTAGAACTGACTGCCAAGCTGCTCGGCCTGTCCCTAGACATCGTCCCAATCAATCTGCTCGCCGGTGATCATCGGACGGACGAGTTTCTGCGCCTTAACCCGCAGCACACAATCCCGGTGATTGACGATGGTGGTGTGATCGTGCGGGACAGTCACGCCATCATCATCTATTTGGTGCAGAAGTACGGAAAGGATGGTCAAACCCTCTATCCGGAAGATCCAATCGCTCGGGCGAAGGTAAACGCTGGCCTGCACTTCGATTCGGGCGTACTGTTCTCTCGGCTGCGGTTCTATTTCGAACCGATACTGTACGAAGGATCGGCGGAAGTGCCGCAGGACAAAATCGACTATATGAAGAAAGGGTACGAGCTGCTGAACGACGCCCTGGTGGAGGATTACATTGCGGGAAGCTCACTTACGCTGGCCGATGTTAGCTGCATCGCAACGATTGCGACGATGGAGGAGTTCTTCCCGATGGATCGCTCCCGCTATCCGGCGCTGGTGGCGTGGATTGAACGGCTCAGTCGTACCTTGCCTGAGTACGACCAGCTCAACCAGGAAGGGGCGGTAGAGTTTGCAGAAATCTGTGAATCGCTGAGGCTTAAAAATGGGGCGTCGGTCGCTGCGAAGTAA

>AGAP009193E4

ATGCCAAACATTAAGCTGTACACGGCCAAACTCAGCCCACCGGGCCGGTCGGTCGAGCTGACAGCAAAGGCGCTCGGGCTGGAGCTCGACATCGTGCCGATCAATCTGCTCGCGCAGGAACATCTGACGGAAGCGTTCCGGAAGCTGAACCCGCAGCACACCATCCCGCTGATCGACGACAACGGGACGATCGTGTGGGACAGCCACGCCATCAATGTGTATCTGGTGAGCAAGTACGGCAAGCCCGAGGGCGACAGTTTGTATCCGTCGGATGTGGTGCAACGGGCGAAGGTTAACGCGGCGCTACACTTCGATTCGGGCGTTCTGTTTGCCCGGTTCCGGTTCTATTTGGAACCAATACTGTACTACGGAGCGACCGAGACACCGCAGGAAAAGATCGACAATCTGTACCGCGCGTACGAGCTGCTGAATGACACGCTGGTCGACGAGTACATCGTGGGCAACGAGATGACACTGGCCGATCTGAGCTGCATCGCCAGCATTGCTTCGATGCATGCGATTTTCCCGATCGATGCCGGCAAGTATCCGAGGCTGGCCGGTTGGGTCAAACGCCTTGCCAAGCTGCCGTACTACGAGGCAACGAATCGGGCCGGTGCGGAAGAGCTCGCTCAGCTGTACCGTGCCAAGTTGGAGCAAAACCGCACCAACGCCAAGTGA

>AGAP009194E2

ATGTCCAACCTTGTACTGTACACCCTGCACCTTAGCCCACCGTGCCGTGCCGTGGAGCTGACGGCCAAAGCATTGGGCTTGGAGCTGGAGCAGAAGACCATTAATCTGCTAACGGGTGACCATTTGAAGCCGGAATTTGTGAAGCTAAACCCGCAACATACGATCCCGGTGCTGGATGACAACGGTACGATCATCACCGAGAGCCACGCAATCATGATCTATCTGGTGACGAAGTATGGCAAAGATGATAGCCTCTATCCGAAAGACCCCGTCAAGCAGGCCCGTGTAAATTCGGCCCTGCACTTCGAGTCCGGCGTACTGTTCGCCCGGATGAGATTCATTTTCGAACGTATCCTGTTCTTCGGCAAATCGGACATCCCCGAGGATCGCGTTGAGTACGTGCAGAAATCGTACGAGCTGCTGGAGGACACACTGGTGGACGACTTTGTCGCCGGACCGACCATGACGATCGCCGACTTTAGCTGCATTTCCACGATCTCGAGCATTATGGGTGTGGTGCCGTTGGAGCAGTCGAAGCATCCCCGGATCTACGCGTGGATCGATCGGCTGAAGCAGCTGCCCTACTACGAGGAAGCGAACGGTGGCGGTGGCACCGATCTGGGCAAGTTTGTGCTAGCCAAAAAGGAGGAGAATGCTAAGGCTTAA

>AGAP009195E1

ATGCCGAAACCTGTGCTATACACGGTTCATCTGAGCCCACCGTGCCGTGCCGTGGAGCTGACGGCAAAAGCGCTTGGATTGGAGTTAGAGCGCAAGCTTGTGAACTTACTTGCTGGGGAAAATTTAACACCTGAGTTCTTGAAGCTCAATCCTAAGCATACGATCCCCGTGCTGGACGATAATGGGACGATCATCAGCGAAAGCCACGCGATCATGATCTATCTCGTGCGTAAGTACGGCCAGGGCGAAGGGAAGGATGCGCTGTACCCAACGGACATTGTCGAGCAGGCTCGGGTCAATGAGGCACTGCACTTCGAGTCCGGTGTGCTGTTTGCTCGGTTGCGATTCATTACCGAGCTGGTATTTTTCGCACGCAAACCAGAAATTCCGGAAGATCGCATCGAGTACGTTCGTACGGCGTACCGGTTGCTGGAAGACTCGCTGCAGAGCGATTACGTAGCCGGATCACGCATGACGATCGCTGACCTGAGCTGCATTTCCTCGGTCGCCTCTATGGTGGGCTTTATCCCGATGGAAAGGTCCGAGTTCCCGCGGGTGCACGGGTGGATCGAGCGGATGAAGCAGTTGCCGTACTATGAGGAGATCAACGGTGCCGGTGCCACAGAGCTGGCCGAGTTCATTGTGGATATGTTGGCGAAGAATGCAAAACTGTAA

>AGAP009196E7

ATGGAACCCAGCAGATTGGTACTGTACACGAACCGCAAGAGTCCACCGTGCCGAGCCGTGAAGCTGACTGCCCGTGCACTCGGCATCGAGCTAGTCGAAAAGGAGATGACGCTGCTGCGCGGTGACAAGCTGATGGAAGAGTTCCTCAAGGTGAATCCACAGCAAACCATACCCGTGCTGGACGATGGTGGCATCGTTATCACCGCAAGCCATGCCATCATGATTTATCTCGTGTGCAAGTACGGCCGCGACGACGGTCTCTATCCGAGCGAGCTGGTACGGCGGGCCCGGGTCCACACGGCACTACACCTCGAGGCGGGTGTAATCTTTTCGCGGTTAAGTTTTCTGTTTGAACCGGTCATTTATTCGGGCAAATCGTACTTCCACTCCGATCGGATCGAACACATCCGGAAGGCGTACCGCTTGTTGGAGGATTCGCTCGTCGATCAGTACATGGTGGGCGAGAGTCTCACGATTGCCGACTTTAGCTGCATCTCCAGCATTGCCACGCTGGTCGGTGTGGTACCGCTGGACGAGTCCAAGTTCCCGAAGAGCACGGCCTGGATGCGGCGCATGCAGGAGCTGCCGTACTACGAGGAAGCGAACGGTACCGGGGCGCTCGAGCTGGCCGAGTTTGTGCTGGGCAAGAAGGAAGCCAATGCTTCCCAGTTCCTGTGA

>AGAP009197E3

ATGGCACCGATTGTGTTGTACAGTACCCGCCGCACGCCGGCCGGCCGTGCCGTGGAGCTGACGGCAAAGATGATCGGTATCGAGCTGGATGTGCAGTACATTGATCTGGCGAAGAAGGAAAATATGACCGAAGAGTATTTGAAGATGAATCCGATGCACACCGTGCCGACGGTCAACGATAACGGTGTGCCGCTGTACGATAGCCATGCCATTATCAACTATTTGGTGCAGAAGTACGCAAAGGATGACACCCTTTACCCGGCGAAGGATCTAGTGAAGCAGGCCAACATCAACGCCCTGTTGCACTTCGAGTCGGGCGTACTGTTCGCCCGGTTGCGCTGGATCCTGGAGCCGGTGTTCTACTGGGGCCAGACGGAGGTGCCACAGGAGAAGATCGACTCGGTACATAAAGCGTACGATCTGCTTGAGGCTACGCTGAAGACATCTGGCACCGACTACCTCGTAGGCGGCACGATCACGCTTGCGGACATCTCCGTCAGCACATCGCTCTGCACGCTCAACGCGCTGTTCCCGGCCGATGCGAGCAAGTACCCGCTGGTGCTGGCCTACCTGAAGCGGCTGGAGCAAACGATGCCCCACTACCAGGAGATCAACACGGACCGGGCCAATGACGCGCTGCAGCTGTACAACCAGAAACTTGGCAAGGTTTAG

>AGAP009342U3

ATGGCTCCCCTGATTTTGTACCACTTTCCGGGCTCACCACCGTCCCGGTCGGCTCTGCTTGCATTGCGCAATTTGGATCTGGATGCCGAGGTGAAAATAGTGAACCTGTTCGCCGGTGAGCATCTGGCGGACGAGTTCGTTGCGATCAACCCGGACCACACCGTGCCGACGCTCGTCGACGAGGACTACATCCTGTGGGAGTCGAAAGCGATCGCCACGTACCTGGCGGAACAGTACAAGCCGGGCTGCACACTCTACCCGTCCCAGCCGAAAAAGCGCGGGCTCATCAACCACCGGCTCTACTTCGACTCCGGCACACTGTTTGTGGCGCTGCGCAACGTGCTGATGACGGTGCTGCGCTCCGGCGAGACGCGCATCCCGCAGGAAAAGAAGGACGCCGTGTACAAGGCGCTGGAAAAGCTCGACTCCTATCTGGACGGGTGCGATTGGATTGCGGGCGAGGAGTGTACGCTGGCCGATCTGTGCGCGTTGGCGAATGTGGCCACGCTGAAGGAAATCGGAGTGGGGATGGAGGGCTACGCCAACGTTAGCGGCTGGTACGAACGGTGCCGCGAGCTGCCCGGTTTCGACGAGAACGAGGAAGGCGCTAGCTTCCTTGGAAATGCGTTCAAATCGAAGCTCGAGGAACAGTTTTAG

>AGAP000761Theta1

ATGTCGAAAAATCTAAAATACTACTACGACCTAATGTCGCAGCCTTCGAGGGCGCTTTGGATTTTTCTGGAGAAAACCAAACTGCCCTACGAAAAGTGTTTGATCAATCTGGGCAAAGGAGAGCATCTGACCGAGGAGTTTAAGGCGATCAATCGCTTCCAAAAGGTGCCCTGCATTACGGACAGCCAGATCAAGCTGGCGGAAAGTGTGGCCATCTTCCGGTACCTGTGCCGGGAGTACCAGGTGCCGGACCACTGGTATCCGGCCGACTCGCGCCGCCAGGCCCTGGTGGACGAGTACCTCGAGTGGCAGCACCACAATACGCGCGCGACCTGCGCGATCTACTTCCAGTACGTCTGGCTGCGGCCGCGCATGTTCGGCACCAAGGTCGACCCGAAGCAGGCGGAAAAGTACCGGGGGCAGATGGAGGGCACGCTGGACTTTATCGAGCGCGAGTATCTCGGGTCGGGCGCCCGGTTCATCGCGGGGGACGAAATTACCGTGGCCGATCTGCTTGCGGCCTGCGAAATCGAGCAGCCGAGAATGGCTGGCTATGATCCGTGCGAGGGCCGCCCAAACCTAACCCAGTGGATGGCACGAGTCCGCGAATCGACCAATCCATACTACGACCAGGCGCATAAGTTAGTGAACAAATTTGCCCAGGACACGGCTAGCAAAGCTAAGCTGTAG

>AGAP000888Theta2

ATGTCCCGTAGTGTTAAACTTTACTATGACCTCATGTCGCAACCGTCGAGGGCGCTTTACATATTCTTATCGACCAACAAGATCCCCTTTGACCGGTGCCCGATAGCGCTGCGAAAAATGCAGCACAAGACGGACGAATACCGCCGGCAGGTGAATCGGTACGGCAAGGTGCCCTGCATCGTGGACGGCAGCTTCCGGCTAGCCGAGAGCGTCGCCATCTACCGGTACCTGTGCCGAGAGTTTCCGACCGACGGCCACTGGTACCCGAGCGACACGGTGCGCCAGGCACGCGTCGACGAGTACCTTTCCTGGCAGCACCTAAACCTGCGCGCCGACGTGTCGCTATACTTTTTCCACGTGTGGCTAAACCCGCTGCTGGGCAAGGAGCCGGACGCCGGCAAGACGGAACGGTTGCGCCGGCGGCTGGACGGTGTGCTGAACTTTTTCGACCAGGAGCTGCTGTCGGCCGGTAGCGGGCAGGCGTTTCTGGCGGGCGATCGCATCAGCATTGCCGACCTATCGGCCGCTTGCGAGATTGAGCAGGCGAAAATAGCCGGGTACGATCCGTGTGAGGGCCGGCCGGCACTGGCCAGCTGGCTGACGGCGGTGCGGGAGCGAACCAATCCGTACTACGACGAGGCGCACAAATACGTTTATCGGCTTTCGCCGGACCACATCGTAACGCCGGTCGTTGCGGAAGATGAGTGA

>AGAP000947U1

ATGAAGCTGTACGCTGTTTCCGATGGCCCACCGTCCCTGGCCGTCCGTATGGCCCTGGAAGCGCTCAACATACCGTACGAGCACGTCAGTGTCGATTACGGCAAGGCGGAACATCTTACCGCGGAGTACGAGAAGATGAATCCGCAAAAGGAAATTCCCGTGCTGGACGATGACGGGTTCTTCCTGAGCGAAAGTAATGCCATCCTGCAGTACCTGTGCGAAAAGTACGCACCGACCAGCGACCTCTACCCGAACGACCCGAAGGACCGTGCGCTCGTGAACCATCGTCTGTGCTTTAACCTGGCCTTTCTCTATCCGCAAATATCCGCCTACGTAATGGCGCCGATCTTCTTCGACTACGAGCGCACCGCGATCGGGCTGAAGAAGCTGCACCTTGCCCTGGCTGCCTTCGAAACGTATCTGCAACGCACCGGGACCAGGTACGCGGCGGGCAGCGGGCTGACGATTGCCGACTTTCCGCTCGTCAGCTCCGTGATGTGCCTGGAGGCGATCGGGTTCGGGCTGGGCGAACGGTACCCGAAGGTGCAGGCGTGGTACGACGGGTTCAAACAGGCCCACCCGTCGCTGTGGGCGATTGCGGCCAAGGGCATGGAGGAGATTGCCGAGTTCGAGAAGAACCCCCCGGACCTGACCGGCATGGTGCACCCGATCCATCCGATCCGGAAGCCGGCAGCGAAATAA

>AGAP012702DeltaPartial

GCGAAAATGGAGCTCTACAGCGATATCGTTTCACCGCCCTGTCAGAATGTGCTCCTGGTGGCTAAGAAGTTGGGCATTGCGCTTAACACCAAGAAGACAAACATTATGGATGCAGCGGACGTAGCTGAGTTGACAAAGGTCAATCCGCAACATTTGATCCCAACGTTCGTGGAGGACGACGGGCATGTGATCTGGGAGTCGTACGCAATTGCCATTTACCTGGTGGAAAAGTACGGCCAAGATGATGCCCTCTATCCAAAAGATCCCAAAGTGCGATCCATTGTAAATCAGCGTTTGTTTTTTGATATCGGAACGCTGTACAAAAACATTCTAGCCAACATTGACGTCCTGATCGAGAAGCAGCAACCTTCGGCCGAATTACGCGGGAAGCTGGAACAAGCGCTTGATCTTACGGAGAAGTTTGTTACCGAGTGTCGTTTTGTTGCTGCCGATCATCTTACGCTAGCTGACATCTTTATGCTTGGTAGCATTACTGCCCTGGAATGGTTTAGGTACGATTTGGAGCGTTATCCCGGCATTCGGGGATGGGTTGAGAGAGTAACGGCCCAATTCCCCGATTATTCCGACTTTCACAAGGAGATAAGGGAAGCCACTAAGCAGTACGTTGCAACGCACTGTCCGCATTTGGAATATTAG

>AGAP012838DeltaPartial

ATACACACGATGGATCTGTATTACCACATTCGCTCACCACCATGTCAGCCGGTTGTATTTCTGGCCCGCCATTTGGGTCTGGAGTTTAACCACATAGTGACGAGCATCTATGATCCGGCGGACTTTGAAGTGCTCAAGAAAGTGAATCCACAACACACCATCCCCACGCTGGTGGACAATGGCCATATTCTGTGGGAGTCGTACGCCATCCTGATCTATCTGGCGGAGAAGTACGCACTGGACGATAGTCTCTACCCAAAGGATGTTTGCGAACGATCGATCGTACATCAGCGGCTCTTCTTCGATAGTGGAATGTTCCAGAACACCACACTGCAGGCTTTATTGTCGCACTTGCGCAACAACCCAATCACGGACGAGCATCTGGCCAAGGTGAAGCGCGGCGTGGAGATAGTGGAGATGTATCTCACGGACAGCCCGTACGTTGCCGGCCAGAAGCTGACCATTGCCGATTTTTCGATCTTTGTAAGCTTCTGCTCGCTCGACATGATGAAGTACGACCTGACGGCGTACCCGAACGTGCAGCGATGGTTCGCAAAGATGGGCACGCACATTCCCGACCTGGAGCCAACG

>AGAP012839DeltaPartial

ATGCGGAACAGTCAGCCCAGCGAGAAGCAGGTGCAAAGGTTGCAGAAGGCAGTCGACGTGCTGGAAAGCTTCCTTTACGAACGATCGTACACGGCCGCAGATCAGCTGACGGTGGCGGACATTTGTTTGCTAGTTACGGTCAATGCGCTCACGCTTTGGTTGGGCTACGAGCTTGCACCTTATCCACGGATCAGAGATTGGCTCGGGAGAGTGGTGGCAGAAATTCCGGGTTGTGCTGAGTTTCAAAGGGAGGTAGAGGACGCCACCAGGGCGTACGTAGTCAATCGAAAGATTTGA
