# Supplementary material for: Molecular Cloning, Characterization and Positively Selected Sites of the Glutathione S-Transferase Family from Locusta migratoria
Source: PLoS One. 2014 Dec 8;9(12):e114776. doi: 10.1371/journal.pone.0114776 (PMC4259467; doi:10.1371/journal.pone.0114776)
Supplement: S4 Data — Sequences of microsomal GSTs used for phylogenetic analysis. (DOC) [file pone.0114776.s010.doc]

>LmGSTM1

MAAAELLLTTSNPVFKAYLLHVAVLGLKMLLMSPLTARQRFKNKIFASPEDTVSMKGAKVKYDHPDIERVRRGHLNDLENITVFFIVALAYLLTNPSPGLAINLFRAYTIARIGHTIVYCVIPLPQPSRFLFWIVGWGITVFMTGSVILKFM

>LmGSTM2

MSLESELRGAATAATTALVAANPVLRDYAYYSALLGLKVLAMGPLTARQRFAKKVFLNPEDAKFFSGSEKLDDPDVERVRRAHRNDLENIPVFMLIGGLYTLTNPDPKLALNLYRGYFAFRLAHTVVYAVYPVPQPARVLCHLGALTICVYMGVKVVKFFF

>LmGSTM3

MLLMSPLTARQRFKNKSFSNPEDTMFVKGSKVRYDHPDVERVRRAHLNDLENISVFFIVALAYVLTNPPPVLAINLFRAFTVARIGHTIVYCILPIPQPARFLFCFVGWLITIFMAGSVILYSL

>LmGSTM4

MITLQVPSQYGYCVLVAVGSIFVLMWKGIKVGVARKKFNIPYPTMYSKDNDQFNCIQRAHQNTLENYPQFLTLLLLGGLEHPVVSAAAGCVWLAGRIAYAKGYYTGNPAKRMQGGFAYLGLLVLLGTSVKFSLRLLGVV

>gi|27349249|dbj|BAC46264.1| bll0999 [Bradyrhizobium japonicum USDA 110]

MYHLTALVTLLAIAFYFFATINVSRARARTGIKVPATSGHPDFERAFRIQVNTLEWMPIFLPSLWLFAIYISDALAAGIGALWIVGRIVYFIGYSQAAAKRGPGFLIQAIAAIALWVGAIGAAVSRLV

>gi|16124382|ref|NP_418946.1| MAPEG family protein [Caulobacter crescentus CB15]

MQQSHALVAIVTLLSLLVYVWMIFRIGGARRRTGIDGPAMTGDPELERHLRVQANTVEWLVIYLPSLWLFALYWNDLFAAAAGVVWIIGRILYALGYAADARKRELGFIIQMLATAVLLFGALGKAIYVYAVIGA

>gi|33863296|ref|NP_894856.1| MAPEG family protein [Prochlorococcus marinus str. MIT 9313]

MTIPVLPAVVTLAAAIVYQGTMFAVAFARSQHKVKAPATSGPEEFERVLRVQQNTLEQMMFFLPVFWLAALSSNTSVACLIGFIWVGARIAYGIGYWKAAKLRGPGFAISLLASAVLLVMAIVGLFNS

>gi|37522626|ref|NP_926003.1| glutathione S-transferase [Gloeobacter violaceus PCC 7421]

MGLSALDTLVWPGLVTVAALVVYYGLSLNVGRARVRYGVAPPETNGNPDFERVLRVQENTTEQLVLFLPSLWLYALFVNPLWAAVLGSVWVVGRVLYALGYYEASERRTPGFAVSVVATLILLGGALVGLLRRLVLP

>gi|7388458|sp|P73795.1|Y1147_SYNY3 RecName: Full=Uncharacterized protein sll1147

MTKTELLWPALITALATMLYLVLVINVGRARAKYGVMPPATTGNEDFERVLRVQYNTLEQLAFFLPGLWLFAIYRDPTIAAILGAVWLLGRILYAWGYYQAAEKRMVGFALGSLSSMILVVGALLSILWQLRQLSQF

>gi|22961974|ref|ZP_00009580.1| hypothetical protein [Rhodopseudomonas palustris]

MFHYTTIATLLALMFYFYTSVQVARARMLYGVKAPAISGHPDFERVFRVQANTLEWLPIFLPSLWLFAYYLSDAFAAAAGAVWIIGRILYMLGYAEAPEKRGLGFAVQMVATAFLWGGSVYGVVHQMLGA

>gi|10946225|gb|AAG24803.1| PGE synthase [Rattus norvegicus]

MTSLGLVMENSQVLPAFLLCSTLLVIKMYAVAVITGQVRLRKKAFANPEDALKRGGLQYCRSDPDVERCLRAHRNDMETIYPFLFLGFVYSFLGPNPLIAWIHFLVVLTGRVVHTVAYLGKMNPRIRSGAYVLAQFACFSMALQILWEVAHHL

>gi|15529961|gb|AAK51127.1| prostaglandin E synthase [Bos taurus]

MPPSGLELMNGQVLPAFLLCSALLVIKMYVVAVITGQVRLRKKAFANPEDAQRHGGLQYCRNDPDVERCLRAHRNDMETIYPFLFLGFVYSFLGPNPFVARMHFLVFFLGRMVHTVAYLGKLRAPTRSLAYTLAQLPCASMALQIVWEAARHL

>gi|23428521|gb|AAL18255.1| prostaglandin E synthase [Equus caballus]

MPPPSLAMVSGQALPAFLLCSTLLVIKMYAVAVITGQVRLRKKAFANPEDALRHGGLQFHRDDQDVERCLRAHRNDMETIYPFLFLGLVYSFLGPDPFVAQMHFLVFFLGRMVHTVAYLGKLRAPTRSLAYTVAQLPCASMALQIVWEAARHL

>AL872105

MMDEVFASFVFYSTLLILKMYIIAVITGQIRLRKKAFANPEDAMRHGGIQYYRQDPDVERYRRAHNNDMENIYPFLFLGAMYSLLDPNPTIARIHFQIFFICRVLHTVAYVLPLKPPTRSVAYSIAQLPCFSMALQILYSVASSW

>AV669487

MIRNEVFSCFLFYAVLLVIKMYVIAIITGQVRLRKKAFANPEDALRHGGLQFHREDPYVERCRRAHINDMENILPFLFLGAIYSLTGPSLFVARLHFLVFSLSRLLHTVAYLLALRAPTRSVAYTIAQIPCVSMAVQILMSVMAYA

>AW147344

MMDNEVFASFVFYSTLLILKMYIIAVITGQIRLRKKAFANPEDALRHGGIQYYRQDPDVERYRRAHNNDMENIYPFLFLGAVYSMLSPNPTIARIHFQIFFICRVLHTVAYVLALKPPTRSMAYSIAQLPCFSMVLQILYSXASFW

>BG895996

MPPPSLEMVSGQVLPAFLLCSTLLVIKMYVVAIITGQVRLRKKAFANPEDAQRHGGLQYCRSDPDVERCLRAHRNDMETIYPFLFLGLVYSFLGPDPFVAWMHFLIFFLGRMVHTIAYLGKLRAPTRSLAYTLAQLPCASMALQIVWEAARHL

>BU415373

MMGNTVFLSFAFYSTILILKMYVVAIITGQVRLRKKAFANPEDALRNGGLQYYREDPDVERCRRAHRNDMENIFPFLFLGAVYSLLEPSPAVARVHFFIFCVGRIVHTVAYLLKLRAPTRSVAYSVAQLPCFSMALQILLAAAPYG

>BW302022

MIRNEVFSCFLFYAVLLVIKMYVIAIITGQVRLRKKAFANPEDALRHGGLQFHREDPYVERCRRAHINDMENILPFLFLGAIYSLMGPSLFVARLHFLVFSLSXLLHTLAYLLALRAPTRSVAYTIAQIPCVS

>CA844840

MIVSEVFSCFAFYGALLVIKMYIIAIITGQVRLRRKAFANPEDALRHGGLQYQRCDSYVERCRRAHVNDMENILPFLFLGAIYSMTGPSLAAARLHFLVFTFARGVHTIAYLCALRAPTRSVAYTLAQVPCVSMAVQILITVAAHA

>gi|7387730|sp|O14684.2|PTGES_HUMAN RecName: Full=Prostaglandin E synthase; AltName: Full=Microsomal glutathione S-transferase 1-like 1; Short=MGST1-L1; AltName: Full=Microsomal prostaglandin E synthase 1; Short=MPGES-1; AltName: Full=p53-induced gene 12 protein

MPAHSLVMSSPALPAFLLCSTLLVIKMYVVAIITGQVRLRKKAFANPEDALRHGGPQYCRSDPDVERCLRAHRNDMETIYPFLFLGFVYSFLGPNPFVAWMHFLVFLVGRVAHTVAYLGKLRAPIRSVTYTLAQLPCASMALQILWEAARHL

>gi|47117327|sp|Q9JM51.1|PTGES_MOUSE RecName: Full=Prostaglandin E synthase; Short=mPGES-1; AltName: Full=Microsomal prostaglandin E synthase 1

MPSPGLVMESGQVLPAFLLCSTLLVIKMYAVAVITGQMRLRKKAFANPEDALKRGGLQYYRSDPDVERCLRAHRNDMETIYPFLFLGFVYSFLGPNPLIAWIHFLVVLTGRVVHTVAYLGKLNPRLRSGAYVLAQFSCFSMALQILWEVAHHL

>gi|11595590|emb|CAC18210.1| related to MICROSOMAL GLUTATHIONE S-TRANSFERASE 3 [Neurospora crassa]

MAITLTLPDEYGYVLLATVSTFFANSFHSINTGRQRKAAGVKYPLAYAPQEVAEKDPKAFAFNCAQRAHANFTENLTPAIGAMLIAGLKYPVLAGALGGLWSLTRVLYTIGYTKKGPQGRTKFGIASSLSLLALKLMAAYTAVQIAFH

>gi|29840967|gb|AAP05968.1| similar to GenBank Accession Number AY050567 microsomal glutathione S-transferase in Oryctolagus cuniculus [Schistosoma japonicum]

MSLSKYLAPPLTSLPLCLPRYYGGVILVGVGAFGLNAYFVRRVMQARKEHNVELPIMYHPTDKLFNCIQRGHQNYLEVLPYFLMALFVGGLRYPRTYTACGVIFLLGRLIYFQGYSTGDPGKRYKGSISMIGGLPMILGLLSFRCSTPGSQCKMYRLYFTQIKETGLHGRIFINT

>gi|21617891|gb|AAM66941.1| glutathione-s-transferase, putative [Arabidopsis thaliana]

MAAITEFLPKEYGYVVLVLVFYCFLNLWMGAQVGRARKRYNVPYPTLYAIESENKDAKLFNCVQRGHQNSLEMMPMYFILMILGGMKHPCICTGLGLLYNVSRFFYFKGYATGDPMKRLTIGKYGFLGLLGLMICTISFGVTLILA

>gi|13702833|gb|AAK38509.1|AC087181_25 putative glutathione S-transferase [Oryza sativa]

MAVSIELTKEYGYVVLALVAYAFLNFWMSFQVGKARRKYKVFYPTMYAVESENKDAKLFNCVQRGHQNSLEMMPLFFVTLLVGGLQHPLVAAGLGVFYAVARFFYFKGYATGIPDNRLKIGGLNFLAIFGLIICTASFGINLVLRESI

>gi|29124627|gb|AAH49037.1| Zgc:56518 [Danio rerio]

MVVLSKEYGYVALTGAASFLLMVHLAHGVVKARKKYNVPYPTMYSDDPETGRIFNCIQRSHQNTIEILSPFLFHLSVGGIQHPRLASVLGMIWIVSRVLYAQGYSTGKPQKRHRGTFGMVALVGLFFCTVDTGRVMLGWGPGIKWPRCFK

>AL658418

MVTHLAINVGKARKQYKVEYPKMYSDDPENGNIFNCIQRAHQNTLESYPPFLFFLAVGGLTHPRATSALGVAWIVGRELYAHGYSTGDPSKRKRGAIGSFALLGLFGATVCSAFKILNWTLNPKTWC

>BX277286

MAVLSKEYGYVILTGAASFVLVTHLAVNVGKARKKYNVEYPTMYSTDAENGKIFNCIQRAHQNTLEVYPSFLFFLATGGIYHPRISTGLGIAWILGRLLYAHGYYTGEPKNRRRGALGSAALIGLVGTGVYSAFQHLGWICPD

>gi|16118439|gb|AAL12230.1| microsomal glutathione S-transferase [Oryctolagus cuniculus]

MAVLSKEYGFVLLTGAASFVMVLHLAINVNKARKKYKVEYPVMYSTDPENGHLFNCIQRAHQNTLEVYPPFLFFLAVGGVYHPRIVSGLGWVWIVGRVLYAYGYYTGDPSKRYRGAVSSLALFGLMGTTVCSAFQHLGWIKPRLGSGAKPCH

>gi|47116746|sp|Q9CPU4.1|MGST3_MOUSE RecName: Full=Microsomal glutathione S-transferase 3; Short=Microsomal GST-3; AltName: Full=Microsomal GST-III

MAVLSKEYGFVLLTGAASFVMVLHLAINVGKARKKYKVEYPVMYSTDPENGHMFNCIQRAHQNTLEVYPPFLFFLTVGGVYHPRIASGLGLAWIIGRVLYAYGYYTGDPSKRYRGAVGSLALFALMGTTVCSAFQHLGWIRPGLGYGSRSCHH

>BF282898

MAVLSKEYGFVLLTGAASFVMVLHLAINVGKARKKYKVEYPVMYSTDPENGHMFNCIQRAHQNTLEVYPPFLFFLTVGGVYHPRIASGLGVAWIIGRVLYAYGYYTGDPSKRYRGAVSSLALFALMGTTVCSAFQHLGWIKPGLGSGSRSCH

>BF076301

MAVLSKEYGFVILTGAASFLMVTHLAINVSKARKKYKVEYPTMYSTDPENGHIFNCIQRAHQNTLEVYPPFLFFLAVGGVYHPRIVSGLGLAWIVGRVLYAYGYYTGEPRKRQRGALSFIALIGLMGTTVCSAFQHLGWVRTGLNSGCKSCH

>BF199367

MTVLSKEYGFVVLTGAASFIMVGHLAINVSKARKKYKVEYPTMYSTDPENGHLFNCIQRAHQNTLEVYPPFLFFLAVGGIYHPRIVSGLGLAWIVGRVLYAYGYYTGEPSKRSRGALGSIALIGLMGTTVYSAFQHLGWVKTGLDCKSKCH

>CB550126

MAVLSKEYGFVLLTGAASFIMVAHLAINVSKARKKYKVEYPTMYSTDPENGHLFNCIQRAHQNTLEVYPPFLFFLAVGGVYHPRIASGLGLAWIVGRVLYAYGYYTGEPSKRSRGALGSIALLGLVGTTVCSAFQHLGWVKSGLGSGSKCCH

>CB296039

MAVLSKEYGFVLLTGAASFIMVAHLAINVSKARKKYKVEYPIMYSTDPENGHIFNCIQRAHQNTLEVYPPFLFFLAVGGVYHPRIASGLGLAWIVGRVLYAYGYYTGEPSKRSRGALGSIALLGLVGTTVCSAFQHLGWVKSGLGSGPKCH

>gi|7387731|sp|O14880.1|MGST3_HUMAN RecName: Full=Microsomal glutathione S-transferase 3; Short=Microsomal GST-3; AltName: Full=Microsomal GST-III

MAVLSKEYGFVLLTGAASFIMVAHLAINVSKARKKYKVEYPIMYSTDPENGHIFNCIQRAHQNTLEVYPPFLFFLAVGGVYHPRIASGLGLAWIVGRVLYAYGYYTGEPSKRSRGALGSIALLGLVGTTVCSAFQHLGWVKSGLGSGPKCCH

>AL921814

MTSDLPVLLAAVSVLSALHMGFQARRVGWSRMKFKIVPPIVTGPPEFERTFRAHQNSVEFYAIFVVLLWISGIFFNEVLAALGGLVYIVGREMYFTGYISESKRRLPGFYLVLFVLLFLAVTATIGIFQAFWTNISEDLIPVIY

>gi|18693189|dbj|BAB58882.2| leukotriene C4 synthase [Rattus norvegicus]

MKEETALLATVTLVGVLLQAYFSLQVISARRTFHVSPPLTSGPPEFERVFRAQVNCSEYFPLFLATLWVAGIFFHEGAAALCGLFYLFARLRYFQGYARSAQHRLDPLYASARALWLLVAMAALGLLVHFLPGTLRAALFRWLQVLLPMA

>BF042736

MAGNSILLAALSVLSACQQSYFAMQVGKARSKYKVTPPSVSGSPDFERIFRAQQNCVEFYPIFIITLWMAGWYFNQVFATCLGLVYIYSRHQYFWGYAEAAKKRVTGFRLSLGVLALLTVLGAVGILNSFLDEYLDIDIAKKLRHF

>BM780769

MPPAVSGSPEFDRIFRAQQNSVEFYPAFMITLWMAGWYFNQVFATCLGLLYIYARHQYFWGYSEAANKRMTGFRLGLGILALLAILGALGIANSFLDEYLDLNVAKKLRHF

>BX260243

MAGDLALLAAVSLLSVLQQSRFAQLVGKSRMKHKVMPPAVTGAPEFERTFRAQQNCAEFYPMFQTVLWIAGWFCNQELAALLGLLYMFARHKYFHGYAQAASERLTGFYLSLIVLACLTILGAAGIVNSFMDEYLGFNVGKKLHKLF

>CA038571

MTTEIPVLLAAVSLFSALQIGYLARRVGLARMTHKIIPPTVTGPPEFERTFRAHQNNVELYPVFLVVLWTSGLLCSEVLAVLGGVVYMVARHMYFNGYVMSTEKRLPGFYLTLGALFCLSVLSTIGILHGIFLEYFYKIVSMMVTH

>CA967794

MTSDLPVLLAAVSILSALHLALQAKRVGWSRMKHKIMPPTVTGPPEFERTFRAHQNSVEMYSVFLVVLWISGIFCSEVLASLGGLLYVVGREMYFTGYIRESNKRLPGFY

>CB496440

MTTEIPVLLAAVSLFSALQMVYLARQVGLARMTHKVMPPTVTGPPEFERTFRAHQNNVELYPVFLVVLWTSGLLFSEVLAVLGGVVYMVARHMYFSGYVMSTKKRLPGFYLTLGALFFLSVLSTIGILHGILLEYFYKIVSMMVTH

>>gi|2833283|sp|Q16873.1|LTC4S_HUMAN RecName: Full=Leukotriene C4 synthase; Short=LTC4 synthase; AltName: Full=Leukotriene-C(4) synthase

MKDEVALLAAVTLLGVLLQAYFSLQVISARRAFRVSPPLTTGPPEFERVYRAQVNCSEYFPLFLATLWVAGIFFHEGAAALCGLVYLFARLRYFQGYARSAQLRLAPLYASARALWLLVALAALGLLAHFLPAALRAALLGRLRTLLPWA

>gi|2842764|sp|Q99735.1|MGST2_HUMAN RecName: Full=Microsomal glutathione S-transferase 2; Short=Microsomal GST-2; AltName: Full=Microsomal GST-II

MAGNSILLAAVSILSACQQSYFALQVGKARLKYKVTPPAVTGSPEFERVFRAQQNCVEFYPIFIITLWMAGWYFNQVFATCLGLVYIYGRHLYFWGYSEAAKKRITGFRLSLGILALLTLLGALGIANSFLDEYLDLNIAKKLRRQF

>gi|20868706|ref|XP_130878.1| hypothetical protein MGC41409 [Mus musculus]

MAGDSSLLAAVSLLSACQQSYFAWRVGRARLKHKIAPPAVTGPLEFERIFRAQQNSLEFYPVFIVMLWMAGWYFNQVFAACLGLLYIYARHKYFWGYAEAAEKRITGFRLSLGILTLLPVLAVLGVASRFLNEYLDFHVAKKLRKPF

>gi|157823501|ref|NP_001099900.1| microsomal glutathione S-transferase 2 [Rattus norvegicus]

MAGDSSLLAAVSLLSACQQSYFALQVGRVRLKYKIAPPAVTGSLEFERIFRAQQNSLEFYSVFIISLWMAGWYFNQVFATCLGLLYIYARHKYFWGYAEAAEKRIIGFRLSLGILALLTVLAVLGVASRFLDEYLDFHVAKKLKRPF

>BU134937

MAKSTQLIDNEVFRAYATYAAIVLLKMMLMSLVTAYFRITRKAFANPEDTASFGKGDSAKKFLRTDADVERVRRGHLNDLENIVPFFGIGLLYALCGPDLSTALLHFRIFAGARILHTFAYLIPLPQPSRGLSWAVGYAVTISMAYKVLSKALYL

>BU905244

MANLSSLMDSEVLRAYATYSTIVLLKMMLMSIATAYFRLTKKVFANPEDARVHAKGGDTKKLLKTDEDVERVRRCHLNDIENIVPFVAIGLIYALTNPNLASALLHFRIFTGSRILHTIAYLLPLPQPSRGLTWVVGYLVTISMAVGILRGVLYL

>BX306987

MAELTHMIDSEVFLAFSTYATIVVLKMMLMSPMTGYFRFTRKAFANQEDTSLASSTEDKKKLVRVDPDVERVRRCHQNDLENIIPFVVIGLLYTLTGPDLSTALLHFRVFVGSRLFHTVAYVLHLPQPSRAVAFLIGLVTTSSMASRVLITALYL

>CA966335

MADLMNNDVFLAFSTYATIVVLKMMFMSPLTGYFRITRKAFSNWEDTAMGKKNPEDRKKMLQTNPDVERVRRCHQNDLENIIPFVVIGLLYAFTGPDLSTALLHFRVFVGSRFIHTVSYVLALPQPSKGLSWVVGMITTFSMAYRVLTTALLL

>CB228792

MVDLTQIMDDEVFMAFASYATIILSKMMLMGATTAFYXMTRKVFANPEDCVTFGKGENAKKYLRTDDRVERVRRAHLNDLENIVPFLGIGLLYSLSGPDLSTAILHFRLFVGARIYHTISYLTPLPHPNKGLSFFIGYGVTSFHGLRVA

>CB446056

MANLSQLMENEVFMAFASYTTIVLSKMMFMSTATAFYRLTRKVFANPEDCAGFGKGENAKKYLRTDDRVERVRRAHLNDLENIVPFLGIGLLYSLSGPDLSTAILHFRLFVGARIYHTIAYLTPLPQPNRALAFFIGYGVTLSMAYRLLKSKLYL

>CF377037

MANLSSLMDSEVLRAYATYATIVLLKMMLMSVATAYFRITKKVFANPEDARAAAKGGDARKLLKTDEDVERVRRCHLNDIENVVPFVAIGLIYTLTNPDLASALLHFRIFTGSRLLHTVAYLLPLPQPSRGLMWIIGYFATISMAVSILRGVLYL

>gi|121741|sp|P08011.3|MGST1_RAT RecName: Full=Microsomal glutathione S-transferase 1; Short=Microsomal GST-1; AltName: Full=Microsomal GST-I

MADLKQLMDNEVLMAFTSYATIILAKMMFLSSATAFQRLTNKVFANPEDCAGFGKGENAKKFLRTDEKVERVRRAHLNDLENIVPFLGIGLLYSLSGPDLSTALIHFRIFVGARIYHTIAYLTPLPQPNRGLAFFVGYGVTLSMAYRLLRSRLYL

>gi|121740|sp|P10620.1|MGST1_HUMAN RecName: Full=Microsomal glutathione S-transferase 1; Short=Microsomal GST-1; AltName: Full=Microsomal GST-I

MVDLTQVMDDEVFMAFASYATIILSKMMLMSTATAFYRLTRKVFANPEDCVAFGKGENAKKYLRTDDRVERVRRAHLNDLENIIPFLGIGLLYSLSGPDPSTAILHFRLFVGARIYHTIAYLTPLPQPNRALSFFVGYGVTLSMAYRLLKSKLYL

>gi|3023900|sp|P79382.3|MGST1_PIG RecName: Full=Microsomal glutathione S-transferase 1; Short=Microsomal GST-1; AltName: Full=Microsomal GST-I

MADLTELMKNEVFMAFASYATIVLSKMMFMSTATAFYRLTRKVFANPEDCSSFGKGENAKKYLRTDERVERVRRAHLNDLENIVPFLGIGLLYSLSGPDLSTAILHFRLFVGARIYHTIAYLTPLPQPNRGLAFFLGYGVTLSMAYRLLKSRLYL

>gi|47116030|sp|Q91VS7.3|MGST1_MOUSE RecName: Full=Microsomal glutathione S-transferase 1; Short=Microsomal GST-1; AltName: Full=Microsomal GST-I

MADLRQLMDNEVLMAFTSYATIILTKMMFMSSATAFQRITNKVFANPEDCAGFGKGENAKKFVRTDEKVERVRRAHLNDLENIVPFLGIGLLYSLSGPDLSTALMHFRIFVGARIYHTIAYLTPLPQPNRGLAFFVGYGVTLSMAYRLLRSRLYL

>BG383543

MKDEVALLATVTLLGVLLQAYFSLQVISARRAFRVSPPLTTGPPEFERVYRAQVNCSEYFPLLLATLWVAGIYFHEGAAALCGLIYLYARFRYFQGYARSAQQRLAPMYASACALWLLLALAALGLLAHFLPAALRTELQELLPRA

>BU439419

MLEQIHLLAAVTVLGVLEQAYFFLQVIHARRKFGISPPNISGPPEFERIFRAQVNSSEYFPIFVALLWQAGLFFHQGLAAALGLLYLYSRYCYFMGYRASSSDRLTPIYFSTGVLWVLIAAATLGLLHFFLSHYVGLNVLRLIAA

>gi|2842634|sp|Q60860.1|LTC4S_MOUSE RecName: Full=Leukotriene C4 synthase; Short=LTC4 synthase; AltName: Full=Leukotriene-C(4) synthase

MKDEVALLATVTLVGVLLQAYFSLQVISARRAFHVSPPLTSGPPEFERVFRAQVNCSEYFPLFLATLWVAGIFFHEGAAALCGLFYLFARLRYFQGYARSAQLRLTPLYASARALWLLVAMAALGLLVHFLPGTLRTALFRWLQMLLPMA

>gi|4028648|gb|AAC98692.1| microsomal glutathione S-transferase-like protein [Drosophila melanogaster]

MASPVELLSLSNPVFKSFTFWVGVLVIKMLLMSLLTAIQRFNTKTFANPEDLMSPKLKVKFDDPNVERVRRAHRNDLENILPFFAIGLLYVLTDPAAFLAINLFRAVGIARIVHTLVYAVVVVPQPSRALAFFVALGATVYMALQVIASAAF

>gi|28571189|ref|NP_788903.1| CG33177 [Drosophila melanogaster]

MDNGPMDATPTAAAFRLILLSKSNPVMGCYMFWTSLLVLKMLVMSLLTARQRMKTKTYANPEDLRLSRSTEVRFGDPNVERVRRAHRNDLENILPFLLMSLAYVASGPNPLTARLLIRIGASARLIHTVVYAIIPVPQPARALAFFTTFAITCFEAGYVLVCCIKYI

>gi|28571191|ref|NP_788904.1| CG33178 [Drosophila melanogaster]

MSAAASNSSKMMTSPGDMFTLENPVFCCYLFWSTVLVVKMLLMSLLTAVQRFRYKIFPNQEDLFFKNLEVQFDDPHVERVRRAHRNDMENILPYFIMSLIYISTNPNADVACILFRVASVARIIHTLVYAVYPVPQPSRILAFATMLLITFYMAAVVALRTLSFI

>gi|22832630|gb|AAN09527.1| microsomal glutathione S-transferase-like, isoform B [Drosophila melanogaster]

MLNPELMSLENQVFRCYLGWSAILILKIFAAGIYTGLMRFFTATFANPEDLMSPKLKVKFDDPNVERVRRAHRNDLENILPFFAIGLLYVLTDPAAFLAINLFRAVGIARIVHTLVYAVVVVPQPSRALAFFVALGATVYMALQVIASAAF

>gi|30908840|gb|AAP37003.1| microsomal glutathione transferase GSTMIC1 [Anopheles gambiae]

MTTLLQNVNEEVFRTYVFWTAVLVVKMLAMSVLTGRQRFRKKVFANPEDIQPSKKGAQPKFDDPDVERVRRAHRNDLENILPFFAIGLLYMLTNPEPFIAINLFRAVAIARIVHTLVYAVVVIPQPARGLSWAIAYFATAYMAVKTALFFL

>gi|30908842|gb|AAP37004.1| microsomal glutathione transferase GSTMIC2 [Anopheles gambiae]

MASPFDSINSEAYKAYVFWSAVLVAKMLLMALLTAIQRFKNKAFASPEDTRVISKKLVPKYDDPDVERVRRAHQNDLENILPFFVIGFLYLLTNPAPWLAINLYRLVAASRILHTIVYAVVVIPQPARFLAFVGAMMPTAYMTLQTILYFML

>gi|30908848|gb|AAP37005.1| microsomal glutathione transferase GSTMIC3 [Anopheles gambiae]

MSLVFGQVEPAIFKAYAFWAAVLGLKMLLMSVLTGLKRGSKKVFSNPEDVKPGGKVAYDDPDVERVRRAHRNDMENILPYFIIGFLYMFTNPSVTVATNLFRLVAVVRISHTVFHVLVPVHKFRGMSWAIGFFTTAFMGVQIVLHFL

>gi|31419261|gb|AAH53181.1| Zgc:63982 [Danio rerio]

MYASVMDNIFLLVLVTLLSVVQNVFFALKVEKECTGHQSKRSAAFERLSCAKRNCMDTYPTFLAVLWCAGICLSQAPAAFAGILYLVVRQKYFVGYLGETSQSTPGFLFGKRILFFLSLMCVVGIINHLMLTYGGSDYKEYIQTITKAASTLLLLP

>CF250881

MDQETLGSVVLLAIVTLISVIQNAFFASKLEHESKHCNGKGIQRPGSSAFERVYTANQNCGHTYPTFLAVLWCAGLLCSQAPAAFAGLMYLFVRQKYFVGYLGERTQSTPGYLFGKRIILFLFLMSVAGILNYYLIYFFGSDFEIHIKTITSAISPLLLIP

>gi|146345364|sp|P20291.2|AL5AP_RAT RecName: Full=Arachidonate 5-lipoxygenase-activating protein; AltName: Full=FLAP; AltName: Full=MK-886-binding protein

MDQEAVGNVVLLAIVTLISVVQNAFFAHKVELESKAQSGRSFQRTGTLAFERVYTANQNCVDAYPTFLVVLWTAGLLCSQVPAAFAGLMYLFVRQKYFVGYLGERTQSTPGYIFGKRIILFLFLMSLAGILNHYLIFFFGSDFENYIRTITTTISPLLLIP

>gi|120267|sp|P20292.2|AL5AP_HUMAN RecName: Full=Arachidonate 5-lipoxygenase-activating protein; AltName: Full=FLAP; AltName: Full=MK-886-binding protein

MDQETVGNVVLLAIVTLISVVQNGFFAHKVEHESRTQNGRSFQRTGTLAFERVYTANQNCVDAYPTFLAVLWSAGLLCSQVPAAFAGLMYLFVRQKYFVGYLGERTQSTPGYIFGKRIILFLFLMSVAGIFNYYLIFFFGSDFENYIKTISTTISPLLLIP

>gi|232099|sp|P30353.1|AL5AP_HORSE RecName: Full=Arachidonate 5-lipoxygenase-activating protein; AltName: Full=FLAP; AltName: Full=MK-886-binding protein

MDQETVGNVVLLAIVTLISVIQNGFFAHKVEHESKTQNGRSFQRTGTLAFERVYTANQNCVDAYPTFLVMLWSAGLLCSQVPAAFAGLMYLFVRQKYFVGYLGERRQSTPGYIFGKRIILFLFLMSLAGIFNYYLILFFGSDFENYIKTITTT

>gi|232100|sp|P30354.1|AL5AP_MACMU RecName: Full=Arachidonate 5-lipoxygenase-activating protein; AltName: Full=FLAP; AltName: Full=MK-886-binding protein

MDQETVGNVVLLAIVTLISVVQNGFFAHKVEHESRTQNGRSFQRTGTLAFERVYTANQNCVDAYPTFLAVLWSAGLLCSQVPAAFAGLMYLLVRQKYFVGYLGERTQSTPGYIFGKRIILFLFLMSVAGIFNYYLIFFFGSDFENYIKTVTTT

>gi|77416859|sp|P30355.2|AL5AP_MOUSE RecName: Full=Arachidonate 5-lipoxygenase-activating protein; AltName: Full=FLAP; AltName: Full=MK-886-binding protein

MDQEAVGNVVLLALVTLISVVQNAFFAHKVEHESKAHNGRSFQRTGTLAFERVYTANQNCVDAYPTFLVVLWTAGLLCSQVPAAFAGLMYLFVRQKYFVGYLGERTQSTPGYIFGKRIILFLFLMSFAGILNHYLIFFFGSDFENYIRTVSTTISPLLLIP

>gi|232102|sp|P30356.1|AL5AP_PIG RecName: Full=Arachidonate 5-lipoxygenase-activating protein; AltName: Full=FLAP; AltName: Full=MK-886-binding protein

MDQEAMGNIVLLAIVTLISVVQNAFFAHKVEHESKTHNGRSFQRTGTPAFERVYTANQNCVDAYPTFLVVLWSAGLFCSQVPAAFAGLMYLFVRQKYFVGYLGERTQSTPGYIFGKRIILFLFLMSLAGIFNYFLILFFGSDFENYIKTITTT

>gi|232103|sp|P30357.1|AL5AP_RABIT RecName: Full=Arachidonate 5-lipoxygenase-activating protein; AltName: Full=FLAP; AltName: Full=MK-886-binding protein

MDQEAVGNVVLLAIVTLISVVQNGFFAHKVEHESRNQNGRSFQRTGTLAFERVYTANQNCVDAYPTFLAVLWTAGLLCSQVPAAFAGLMYLFVRQKYFVGYLGERTQSTPGYIFGKRIILFLFLMSLAGILNYCLILLFGSDFENYIKTISTT

>gi|232104|sp|P30358.1|AL5AP_SHEEP RecName: Full=Arachidonate 5-lipoxygenase-activating protein; AltName: Full=FLAP; AltName: Full=MK-886-binding protein

MDQETVGNIVLLAIVTLISVVQNGFFAHKVEHESKTHNGRSFQRTGPLAFERVYTANQNCVDAYPTFLVMLWSAGLLCSQVPAAFAGLMYLFVRQKYFVGYLGERTQSTPGYIFGKRIILFLFAMSLAGILNYFLIAFFGSDFENYIKTVTTT

>gi|15600793|ref|NP_232423.1| glutathione S-transferase [Vibrio cholerae O1 biovar El Tor str. N16961]

MVTTGYAVVLCGWLIYLAVQVIRQRRKHQVLFADGGVDALVRARSAQSNATEYIPIFLILLGLAEMNGVNVWWIHALGVAFVVGRVLHADSMFKATIPNRVRGMQLTFGCLAVLMVLNLWVLPYSKFFYPLPT

>gi|16122290|ref|NP_405603.1| hypothetical protein YPO2051 [Yersinia pestis CO92]

MVSSLYVVLGALLLIKLSFDVVKLRNQYRVAYGDGGFYELQTAIRVHGNAVEYIPIAVILLIMMEMNGALTWMIHICGLMLIVGRLLHYYGLRHREIRWRRSGMSATYVSLVLMIIANIYYLPWDQIFSFT

>gi|16124565|ref|NP_419129.1| hypothetical protein CC_0310 [Caulobacter crescentus CB15]

MDTIVSGHAAALWAGLNLFLLLILSLLVVRLRQKHKVALGDEGIPELARAIRAFGNASEYIPSGIAALAVLAVAGAAPLAIHVVGFILFAGRVVHAIGLSNSGGASIPRAVGMVATWLAYIFAGVALLLSAIG

>gi|24371907|ref|NP_715949.1| membrane protein MAPEG superfamily [Shewanella oneidensis MR-1]

MPLIVTGFYASLTGLLIVALAYRVVKIRKSQKIGIGDGGNNALVLADRVHANLIENAPIVLILMLVAEAGGLAHFYLHCFGTVWIVARLLHAIGLTQGKGGYHFGRFWGVLLTWLVTLSLALVNLVHFAQSM

>gi|28867610|ref|NP_790229.1| hypothetical protein PSPTO_0380 [Pseudomonas syringae pv. tomato str. DC3000]

MTVAFWCVLVAILLPIICAGIAKFGSGKFGSGHNHDPRAFLDKLEGFPRRAHAAQLNSFEVTPAFAAAVIIAHIAGNAQLVTIDVLAVLFITSRLLYIIFYLADLAALRSVVWLAGMGLIIALFGVSAFPAVS

>gi|28900607|ref|NP_800262.1| glutathione S-transfersae-like protein [Vibrio parahaemolyticus RIMD 2210633]

MITALYASILALLLVWLAFQVIKQRRLNKVAYADGGVEALQIARSAQSNASEYIPITLILMALLEFNGAAPIWIHLTGIIFVIGRIIHARGILQESFKGRVKGMQLTFLVIVSLVVLNMFYFPYGKLW

>gi|37526022|ref|NP_929366.1| hypothetical protein plu2106 [Photorhabdus luminescens subsp. laumondii TTO1]

MVSSLYVVLGALLLIKLSFDVVKLRTQYRVSYGDGGFYELQTAIRIHGNAVEYIPISMLLLVMMEMNGSNVWMIHVCGLILLTGRVLHYYGLYHREFYWRRSGMVATYLSIVLMVITNIYYLPWEQIFSLY

>gi|13476897|ref|NP_108466.1| hypothetical protein mlr8352 [Mesorhizobium loti MAFF303099]

MNQTTIFWPVLAHVALIYIVYVQMGRRRYFAVKSGEARVGQYKVRSTEPASSLAVANNLINQFELPVLFYTLCLTLHVTNGVNYLTLALAWIFVLTRYFHAWVHLTSNNLRLRSRSFFAGAVVLALAWIWFALHLLGIV

>gi|15595737|ref|NP_249231.1| hypothetical protein PA0540 [Pseudomonas aeruginosa PAO1]

MTIAFWCVLIAIFLPYVCTSIAKFGGEGYGGRANADPRAFLGSLEGFRRRANNAQLNAFEVTPAFAAAVIIAHLVGNASQATLDSLAIAWITSRLLYVICYLADWGPLRSLVWFVGMLLIAAFFVVSA

>gi|15676871|ref|NP_274016.1| hypothetical protein NMB0979 [Neisseria meningitidis MC58]

MTFAYWCILIACLLPLFCAAYAKKAGGFRFKDNHNPRGFLAHTQGAAARAHAAQQNGFEAFAPFAAAVLTAHATGNAAQSTINTLACLFILFRLAFIWCYIADKAAMRSLMWAGGFACTVGLFVAAA

>gi|15887966|ref|NP_353647.1| Conserved hypothetical protein [Agrobacterium fabrum str. C58]

MSPTTAMFWPMIAHAFLVFILYALLLHRRKNHTLTSREAVTQYRERGEEGQASYLVNRNIANQFELPVLFHAICLLLYITDADNVVTVVLAWLFVISRYAHSYVHVTSNRLRYRAPLFGIGFALLVCLWGWLAIWLALE

>gi|159185303|ref|NP_355570.2| conserved hypothetical protein [Agrobacterium fabrum str. C58]

MQPTFLAASPFLPLIGLSVFLLVAHVLLQGMTATRELGREWNAGPRDGELKPQGKLSGRASRASANFRETYPAFLALAFGVIMAGDPAGIALIGAWLWLICRIIYIPLYLAGVPYIRSFVWLGSMLGLALMFVVLMF

>gi|16125865|ref|NP_420429.1| hypothetical protein CC_1618 [Caulobacter crescentus CB15]

MRMAFELQMIAVAVAIGILNLLWASAAAQPQRGLKWNVGPRDETIELTGMAGRLMRAFANFRETFPFFVALVIVDYLGGRLGDLTVYGAALYVAARAAYLPLYAFGVPYVRSLVWLASMVGILMLLAALVV

>gi|16760854|ref|NP_456471.1| hypothetical protein STY2112 [Salmonella enterica subsp. enterica serovar Typhi str. CT18]

MVSALYAVLGALLLMKFSFNVVRLRMQYRVAYGDGGFSELQSAIRIHGNAVEYIPVALVLLLFMEMNGAETWMVHICGIILIAGRLMHYYGFHHRLFRWRRAGMSATWCALLLMVLANLWYMPWELVFSLY

>gi|17232155|ref|NP_488703.1| hypothetical protein all4663 [Nostoc sp. PCC 7120]

MIIFLYSIAAAAVLIYLPFLVVGYARARAGYDVSAPRAMFDKLPPYGQRATWAHQNSFEAFMVFAAAALMAYVTGVNSPTAAWAAIAFLVARLLYSIFYILNIPLLRSLMYAIGSLGSGTLFVLSIIQAQG

>gi|17545507|ref|NP_518909.1| hypothetical protein RSc0788 [Ralstonia solanacearum GMI1000]

MPIALWCVLIAALLPYVCIQIARFTGPRRDNRQPRQWAAGLTGVAQRANGAQQNHFEVFPFFAVAVLVAILGGSPVDRVNGLAVAFIAVRVLYTVCYLADWATARSLVWTVGLVLTIALFVQPAFVH

>gi|17986426|ref|NP_539060.1| hypothetical protein BMEI0142 [Brucella melitensis bv. 1 str. 16M]

MEPGVLSASPFLPLIGWSVVLLVVHILLQSMMATQELGSRWNAGPRDESLKPSGRLAGRAERASANFRETYPAFIALALALVLKGDPSGWGILGAWLWFFSRIVYIPLYLAGIPYLRSFVWLISLVGLGIMLLALVL

>gi|21232079|ref|NP_637996.1| hypothetical protein XCC2648 [Xanthomonas campestris pv. campestris str. ATCC 33913]

MQLSIELQMLGWAMVLGLVQLLAASTSMTAQRGTKWNASARDGETKPLTGVAARLDRAFRNFLETFAIFAAAVLAVTVAGRTNAETALGVQLYLWARVAYVPVYALGIPYLRSAIWVVSFWGIVKLVRALLGW

>gi|21243545|ref|NP_643127.1| hypothetical protein XAC2818 [Xanthomonas axonopodis pv. citri str. 306]

MQLTIELQMLGWAMVLGLVQLLAASAGMTAQRGTKWNASARDGEAKPLTGVAARLDRAFRNYLETFPIFAAAVLAVSVAGRTSADTALAVQLYLWARVAYVPVYAAGIAYLRSAIWVVSFWGIIKLVRALLGI

>gi|22298638|ref|NP_681885.1| hypothetical protein tll1094 [Thermosynechococcus elongatus BP-1]

MMLSNIPWLLAASLLTAMLLIYFPYIFVVVGRLQVGFDMAAPRALFEKLPPFAQRAVWAHENSFETFMPFAAAVLLTLFAGVNNVTVAIASLSFVVARFLYSICYIANFPLGRSLMFGVGTAATLTLFWQSLTALAA

>gi|56479987|ref|NP_707757.2| hypothetical protein SF1910 [Shigella flexneri 2a str. 301]

MVSALYAVLSALLLMKFSFDVVRLRMQYRVAYGDGGFSELQSAICIHGNAVEYIPIAIVLMLFMEMNGAETWMVHICGIVLLAGRLMHYYGFHHRLFRWRRSGMSATWCALLLMVLANLWYMPWELVFSLR

>gi|24216198|ref|NP_713679.1| hypothetical protein LA_3499 [Leptospira interrogans serovar Lai str. 56601]

MNPAIIALLGFIFWTLFLGLCVVSVRSFKVLTGSNKSNEFPAGIKHGSEFYWRLNRAHINCIENLPIFGILVLIGVFAGVFDHRFELATQIILGARIFQTLAHLSSGSVFAVNARFTGFMIQYGCFLYLLWHILHNWQLRSF

>gi|24375205|ref|NP_719248.1| inner membrane MAPEG superfamily protein [Shewanella oneidensis MR-1]

MNTLLTCLFIAMLLPYLAKGPVAWAMAKAGGYDNHHPRSQQAQLTGFGARALAGHQNAFESLLIFGLAVLTVIATGKVTPTAEWLAIVHIVARFAYQILYLLNKGTLRSLSWFVAIFSAFGIFFQAF

>gi|26989373|ref|NP_744798.1| eicosanoid and glutathione metabolism membrane protein [Pseudomonas putida KT2440]

MSSALQAYALCVVVLFLKTFLVSCYQGYHRLRFVAFTNPEDAAVFRRIAQAVERPQVIRAAKVWANDLENIPMFFALGGLAVALEAATLPVLWLSVVFTVARVLHTLAYLRGLQPWRTLFYGIGVICLLGFCLLITARIGG

>gi|27379470|ref|NP_770999.1| hypothetical protein bll4359 [Bradyrhizobium japonicum USDA 110]

MTRELFWLTLTVIFTGILWVPYILDRCQVRGLSGAMANPSRSDKPQSPWATRLMFAHDNAVENLVIFAPLVLILNAIDYSSKWTVLACAVYFWSRVAHLIVYAMGIPVFRTLAFTVGFLAQAVLALAIFKVL

>gi|27382864|ref|NP_774393.1| hypothetical protein blr7753 [Bradyrhizobium japonicum USDA 110]

MTLAEWCVFGALLLYLATIASIKWIRFRGFDNSRPRDPAFYEDAIAQRALGAHQNGIETFPFFAFAVLLAEFRDSPQRLIDELAVLFLIVRIAYVLTYLGNRPTLRSILWSIGFAINLVIFFMPMLKRFLPV

>gi|33593626|ref|NP_881270.1| hypothetical protein BP2656 [Bordetella pertussis Tohama I]

MNATAAFLLAAALLPLVPAIAAKAGGRRYDNNDPRPWLAGQAGWRARANSAQANTFEALPFFFAAVLYALHTQAPAATLHVLMGAWLVARLVYVALYIGGRGNLRSLIWLAALLVNVAILLAGS

>gi|33602048|ref|NP_889608.1| hypothetical protein BB3072 [Bordetella bronchiseptica RB50]

MAPQRTSMNATAAFLLAAALLPLVPAIAAKAGGRRYDNNDPRPWLAGQAGWRARANSAQANTFEALPFFFAAVLYALHSQAPAATLHVLMGAWLVARLVYVALYIGGRGNLRSLIWLAALLVNVAILLAGS

>gi|33861571|ref|NP_893132.1| hypothetical protein PMM1015 [Prochlorococcus marinus subsp. pastoris str. CCMP1986]

MPIVFAWSLCLSVVVVLLSTIPLTLGRIKAGYSVENMSAPRALFDKLPDFGKRAVWCHQNCWESISIHAPACILCLITLPDSNLSLIAAWMHPLLRFLYIGAYVLNIPIARGLIWASGIFTTLVLYKEGISQFM

>gi|34495724|ref|NP_899939.1| hypothetical protein CV_0269 [Chromobacterium violaceum ATCC 12472]

MIYPMFALVLLTIFVGVRLGAARFANARSGKVKGSYYRLMQGDAPPDGELKLARNFSNLFETPVLFHVFGAIVIAKNLAAPLPLALAWSYVALRAVHTAIHLSYNHPIHRFLAFLASNILLLAMWVWLAVVL

>gi|34497045|ref|NP_901260.1| transmembrane protein [Chromobacterium violaceum ATCC 12472]

MPFVLWSLLLAALLPLIWAGAAKAGARYDNHRPREWAARLDGYRQRANWAQQNAWEALPTYLAAVAAAWWMKVPVAEMNAAAGVFALARVAHGLLYVADKAALRSLAWLVGLLAVLYLFLRAGGVIAL

>gi|37523841|ref|NP_927218.1| hypothetical protein glr4272 [Gloeobacter violaceus PCC 7421]

MSGPLLLYGCLAAAAALIYVPFVLVALGRLQVGYDYAAPRATFDKLPPYAQRATWAHQNAFEGFALYTAAVLMVLVSGQTGELANTLAVAYLAFRAGHGLFYIANLPWLRSGMWALAMTCIAGLMAIALGMLG
